# HIV drug resistance in HIV positive individuals under antiretroviral treatment in Shandong Province, China

**DOI:** 10.1371/journal.pone.0181997

**Published:** 2017-07-27

**Authors:** Bin Lin, Xiaoguang Sun, Shengli Su, Cuixia Lv, Xiaofei Zhang, Lin Lin, Rui Wang, Jihua Fu, Dianmin Kang

**Affiliations:** 1 Shandong Center for AIDS Control and Prevention, Shandong Center for Disease Control and Prevention, Jinan, Shandong Province, China; 2 Qilu Hospital of Shandong University, Jinan, Shandong Province, China; National and Kapodistrian University of Athens, GREECE

## Abstract

The efficacy of antiretroviral drugs is limited by the development of drug resistance. Therefore, it is important to examine HIV drug resistance following the nationwide implementation of drug resistance testing in China since 2009. We conducted drug resistance testing in patients who were already on or new to HIV antiretroviral therapy (ART) in Shandong Province, China, from 2011 to 2013, and grouped them based on the presence or absence of drug resistance to determine the effects of age, gender, ethnicity, marital status, educational level, route of transmission and treatment status on drug resistance. We then examined levels of drug resistance the following year. The drug resistance rates of HIV patients on ART in Shandong from 2011 to 2013 were 3.45% (21/608), 3.38% (31/916), and 4.29% (54/1259), per year, respectively. *M184V* was the most frequently found point mutation, conferring resistance to the nucleoside reverse transcriptase inhibitor, while *Y181C*, *G190A*, *K103N* and *V179D/E/F* were the most frequent point mutations conferring resistance to the non-nucleoside reverse transcriptase inhibitor. In addition, the protease inhibitor drug resistance mutations *I54V* and *V82A* were identified for the first time in Shandong Province. Primary resistance accounts for 20% of the impact factors for drug resistance. Furthermore, it was found that educational level and treatment regimen were high-risk factors for drug resistance in 2011 (P<0.05), while treatment regimen was a high risk factor for drug resistance in 2012 and 2013 (P<0.05). Among the 106 drug-resistant patients, 77 received immediate adjustment of treatment regimen following testing, and 69 (89.6%) showed a reduction in drug resistance the following year. HIV drug resistance has a low prevalence in Shandong Province. However, patients on second line ART regimens and those with low educational level need continuous monitoring. Active drug resistance testing can effectively prevent the development of drug resistance.

## Introduction

Zidovudine (AZT) was the first antiretroviral drug approved by the Food and Drug Administration to be used as a treatment for AIDS in 1987, and it was subsequently approved as a preventative treatment in 1990. The introduction of highly active antiretroviral therapy (HAART, or cocktail therapy) in 1996 was the turning point in the history of AIDS therapy, as it transformed AIDS from a death penalty to a manageable disease [1]. However, over the course of antiretroviral therapy(ART), its efficacy becomes increasingly limited because of the generation of drug-resistant viruses [2].

HIV drug resistance is a combined result of the high replication and mutation rates of HIV and long-term drug use in patients. It has been estimated that there is a total of 10^7^~10^8^ infected cells [3] in the lymphoid tissues of most untreated HIV-positive individuals. During chronic infection, the number of infected cells remains relatively stable because the viruses keep replicating and infecting new cells while the infected cells die (half-life as short as 1–2 days). Owing to the lack of a proof-reading function in the HIV reverse transcriptase, one mismatched base pair can be generated for an average of 2000–5000 bases during replication [4], resulting in the generation of many virus variants in infected individuals and a high degree of heterogeneity in the viral population [5]. Meanwhile, the possibility of gene recombination also increases the heterogeneity of the population. As a result, a “quasispecies” is formed by all the HIV mutants present in a HIV-infected person, with the most adaptable virus being the dominant species. Although gene mutation occurs at random, the viral strain(s) that can continue to replicate and proliferate under the selective pressure of drug treatment will quickly become the dominant species in the patient, leading to the development of secondary resistance [6]. On the other hand, primary resistance, which refers to the presence of drug resistance prior to ART, including natural resistance [7, 8] and the presence of drug-resistant infectious strains, is also often observed in HIV-infected individuals. Many studies on HIV drug resistance have focused on the virus itself, and a multifactorial study on transmission, mutation, treatment and testing is still currently lacking.

Among the eight antiretroviral drugs freely provided by the Chinese government presently, AZT, lamivudine (3TC), stavudine (d4T), abacavir (ABC) and tenofovir (TDF) are members of the nucleoside reverse transcriptase inhibitor (NRTIs) family; efavirenz (EFV) and nevirapine (NVP) are members of the non-nucleoside reverse transcriptase inhibitor (NNRTIs) family; and lopinavir/r (LPV/r) is a protease inhibitor (PIs). The first line ART regimens are usually composed of two NRTIs (usually two of TDF, AZT and 3TC) and one NNRTIs (NVP or EFV). The second line ART regimens are usually composed of two NRTIs (usually two of TDF, AZT and 3TC) and LPV/r [9]. TDF used to be in the second line in the early years then changed into the first line in 2011. The use of d4T in the first line ART regimens has gradually stopped since 2015 because of its side effects [9].

Free ART for AIDS began in Shandong Province in 2003, and the number of patients on ART had already surpassed 7,000 by the end of 2016. In addition, ever since the implementation of drug resistance testing in 2009, it was found that the development of resistance is also increasing yearly. Therefore, it is of great importance to fully examine the current status and impact factors of HIV drug resistance, as well as the effect of drug resistance testing. In order to reduce bias and to observe the effect of drug resistance testing, we have collected and analyzed over 3 years of data in the present study.

## Patients and methods

### Ethics statement

All participants were granted with written informed consent to participate in the study. The informed consent was explained to all participants by the stuff and only those who agreed to sign the informed consent were enrolled. All of the signed informed consents were locked in a cabinet in our laboratory with the only access by the study staff. The study protocol and the informed consent procedures were approved by the Ethics Committee of Shandong Center for Disease Control and Prevention (Approval number:2017–20).

### Patients

Data were collected using the full sampling method. Patients who were already on or new to ART in Shandong Province from 2011 to 2013 were included in this study. Qualified patients must have been on ART for >6 months, were aged ≥18 years, could provide a signed written informed consent, and had no obvious physiological or mental illness. Blood samples were collected in accordance with standard procedures by laboratory personnel of the local Center for Disease Control and Prevention (CDC), and were delivered to our laboratory for testing in compliance with relevant bio-safety requirements. Other information on the patients was downloaded directly from the online reporting system.

### Sample processing

After arrival at the laboratory, blood samples were centrifuged at 1000 × *g* for 15 minutes to obtain the plasma, which was then collected into three tubes and stored at −70°C. Of the three tubes, two were used for viral load testing and drug resistance testing, and the remaining one was reserved as a backup. Viral load testing was performed using bioMérieux instruments and corresponding NucliSENS 2.0 Reagents. Drug resistance testing was only conducted on samples with a greater than 1000 cp/ml viral load.

### Samples for primary resistance testing

We carried out primary resistance testing for patients with drug resistance. The blood samples for the testing were collected in the past. The blood samples were collected by county laboratories for HIV screening test. Afterward, the samples were delivered to city laboratories for recheck, and then delivered to our laboratories for HIV confirmatory test. The samples were stored in -70°C refrigerators in the sample library of our laboratory. The average storage period was 3.21 years. The longest preserved samples were kept for 8.62 years.

### Drug resistance testing

The pol region was amplified using an in-house method (Table 1, https://dx.doi.org/10.17504/protocols.io.h6yb9fw) to obtain a target amplicon of about 1300bp. Sequences were spliced and processed using the ChromasPro 1.41 software, and the processed sequences were compared with those in the HIV Drug Resistance Database by Stanford University (https://hivdb.standford.edu). Sequences with a low degree of resistance or above were considered as showing drug resistance. The Stanford database can also identify the HIV subtypes through these sequences.

**Table 1:** Primers for detecting drug resistance.

Primer name	Sequence	Location (HXB2)	Direction
MAW-26^★^	5′-TGGAAATGTGGA AAGGAAGGA C-3′	2027–2050	Upstream of flanking region
RT-21^★^	5′-CTGTATTTCTGCTATTAAGTCTTTTGA -3′	3509–3539	Downstream of flanking region
PRO-1^※^^Δ^	5’-CAGAGCCAACAGCCCCACCA-3’	2147–2166	Upstream of sequence (forward)
RT-20^※^^Δ^	5′-CTGCCAGTTCTAGCTCTGCTTC -3′	3441–3462	Downstream of sequence (reverse)
RT4R (backup) ^※^	5’-CTTCTGTATATCATTGACAGTCCAGCT-3’	3300–3326	Downstream of sequence (reverse)
RT1^※^	5′-CCAAAAGTTAAACAATGGCCATTGACAGA-3′	2604–2632	Forward
PROC1^※^	5’-GCTGGGTGTGGTATTCC-3’	2826–2842	Reverse

^★^indicates first round primer.

^Δ^indicates second round primer.

^※^indicates sequencing primers.

### Data analysis and quality control

Statistical analysis of the data was performed using SPSS 17.0 statistical software. Sequence splicing, processing and inputting were operated separately by two technicians to ensure the quality of the result.

## Results

608 patients were recruited in 2011. The age range was from 18 to 77 years old with a mean age of 39.82 years old. 572 patients were on the first line treatment, and 36 patients were on the second line treatment.

916 patients were recruited in 2012, and 572 of them were from 2011. The age range was from 19 to 78 years old with a mean age of 39.05 years old. 863 patients were on the first line treatment, and 53 patients were on the second line treatment.

1259 patients were recruited in 2013, and 854 of them were from 2012. The age range was from 19 to 81 years old with a mean age of 38.89 years old. 1175 patients were on the first line treatment, and 84 patients were on the second line treatment. 556 patients remained in the study for all three years.

### 1. Drug resistance

**Fig 1:**
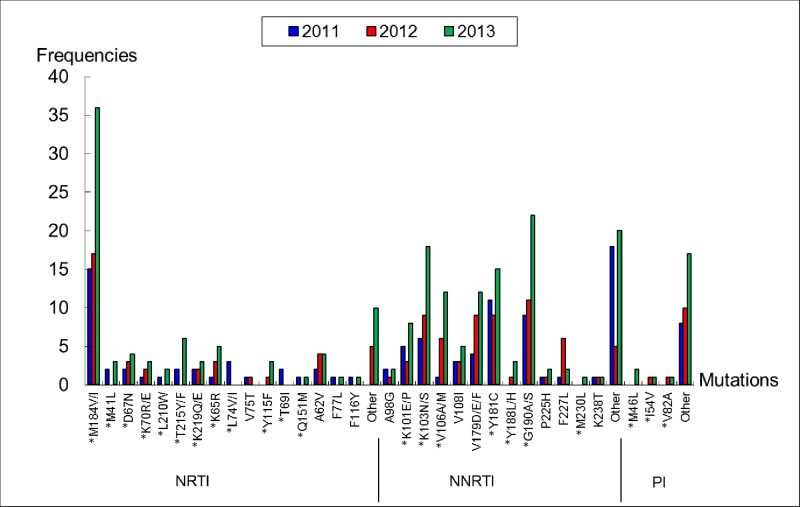
Drug resistance mutations and frequencies among HIV patients in 2011–2013. Note: *indicates second round primer. The drug resistance mutations labeled ‘Other’ indicate that they are related to drug resistance, but the mutations alone do not result in drug resistance.

The HIV drug resistance rates among 608 HIV patients from 2011, 916 patients from 2012, and 1259 patients from 2013 were 3.45% (21/608), 3.38% (31/916), and 4.29% (54/1259), respectively. Drug resistance mutations and frequencies are shown in Fig 1. We found that *M184V* was the most frequent point mutation conferring resistance against NRTIs, whereas *Y181C*, *G190A*, *K103N* and *V179D/E/F* were the most frequent point mutations conferring resistance to NNRTIs. Additionally, the PIs drug resistance mutations *I54V* and *V82A* were identified for the first time in Shandong Province. The two mutations can reduce susceptibility of PIs, including LPV/r, which is the only PIs in second-line regimen.

The rates of HIV drug resistance in patients with second line ART regimens were 16.67% (6/36) in 2011, 5.66% (3/53) in 2012, and 13.10% (11/84) in 2013. We found that M184V and Y181C were also the most frequent point mutations in these patients. No major or minor mutation conferring resistance against PIs was found in patients with second line ART regimens. No patient was resistant to LPV/r.

B and 01-AE were the major HIV subtypes among drug resistant patients. Among the 21 drug resistant patients in 2011, 10 were subtype B, 10 were subtype CRF01-AE, and 1 was subtype C. Among the 31 drug resistant patients in 2012, 14 were subtype B, 15 were subtype CRF01-AE, and 2 were subtype C. Of the 54 drug resistant patients in 2013, 16 were subtype B, 35 were subtype CRF01-AE, and 3 were subtype C. All the results of HIV subtypes above were obtained from the Stanford database, using the drug resistance sequences. We did not detected HIV subtypes separately to all patients.

### 2. Impact factors for drug resistance

#### 2.1 Primary resistance

The samples for HIV confirmatory test of drug-resistant patients were used as early samples (prior to ART) to test for the presence of drug-resistant HIV strains, i.e., primary resistance. However, out of the 106 drug-resistant patients from 2011 to 2013, we were only able to acquire the drug resistance sequences from 10 patients. Table 2 shows the changes in drug resistance sites. In particular, samples #2 and #9 already showed an emergence of resistance mutations at the time of confirmed diagnosis, which indicated that the patients were infected with drug-resistant strains and thereby had primary resistance.

**Table 2:** Detection of primary resistance in 10 HIV drug-resistant patients.

Sample no.	Mutations identified at time of diagnosis			Mutations identified at time of this study		
	NRTI	NNTTI	PI	NRTI	NNTTI	PI
1	*None*	*None*	*A71T*	*M184V*	*V108I*,*Y181C*	*None*
2	*None*	*V179D*	*None*	*None*	*V179D*	*None*
3	*None*	*None*	*A71T*	*M184V*	*V106I*, *Y188FL*	*A71T*
4	*Y115NY*, *Q151PQ*	*V106EIKV*	*None*	*D67N*, *K70KR*, *F116FY*,*Q151M*, *M184V*	*Y181C*, *G190A*, *H221Y*	*None*
5	*None*	*None*	*None*	*M184V*	*K101E*, *G190A*	*None*
6	*None*	*V106I*	*None*	*M184V*, *T215F*	*V106I*, *Y181C*, *H221Y*	*None*
7	*V118I*	*V106I*	*None*	*T69NT*, *V118I*, *M184V*	*V106I*, *Y181C*	*None*
8	*Y115CDGY*, *F116FI*	*V108AV*	*A71AT*	*M184V*, *T215F*	*K103N*, *V108IV*, *M230LM*	*A71AT*
9	*None*	*None*	*L10I*, *A71AV*	*None*	*None*	*L10IL*, *A71AV*
10	*None*	*None*	*None*	*M41L*, *T69N*, *M184V*,*L210W*, *T215Y*	*K103N*, *Y181C*	*None*

In the mutations identified at time of diagnosis, A71T, A71AT, A71AV, V179D, V106I, V106EIKV are polymorphic mutations. V108A is a highly unusual mutation at the position of 108, where usually is V108I, a non-polymorphic accessory mutation. A71T, A71AT, A71AV, L10I are PI minor resistance mutations.

We also observed the development of mutation and drug resistance in one patient after 6 months of ART, the shortest time in which drug resistance had been recorded as developing. The patient was samples #5, male, 29 years old, Han nationality and unmarried. The transmission route was male-male homosexual intercourse. The treatment regimen for him was AZT+3TC+NVP.

#### 2.2 Risk factors for drug resistance

Patients were divided based on the presence or absence of drug resistance, and the effects of age, gender, ethnicity, marital status, educational level, route of transmission and status of treatment on drug resistance were examined. We found (Tables 3–5) that both educational level and treatment regimen were high risk factors for drug resistance in 2011 (P<0.05). A multivariate logistic regression showed that the odds ratio (OR) values of educational level and treatment regimen were 0.55 and 3.07, respectively. Furthermore, treatment regimen was a high risk factor for drug resistance in both 2012 and 2013 (P<0.05), but no significant effects from other factors were observed.

**Table 3:** Analysis of impact factors for drug resistance in 2011.

Variables	Drug resistance		OR value	OR value 95% CI	χ^2^ value	P value
	Yes	No				
**Age (years)**						
<30	6	90	1.000		4.421	0.219
30-	5	219	0.342	0.102–1.151		
40-	5	191	0.393	0.117–1.321		
50-	5	87	0.862	0.254–2.928		
**Gender**						
Male	15	381	1.000		0.38	0.538
Female	6	206	0.740	0.283–1.936		
**Ethnicity**						
Han	20	531	1.000		0.128	0.721
Other	1	56	0.474	0.062–3.600		
**Marital status**						
Single	4	100	1.000		3.265	0.353
Married	11	380	0.724	0.226–2.321		
Divorced or widowed	4	64	1.064	0.259–4.376		
Other	2	13	3.846	0.64–23.107		
**Educational level**						
Illiterate	8	123	1.000		11.247	0.024
Elementary	6	108	0.854	0.287–2.540		
Junior high school	6	182	0.507	0.172–1.497		
Senior high school and secondary school	1	89	0.173	0.021–1.406		
College and above	0	85	0.000	0		
**Route of infection**						
Blood transfusion	3	53	1.000		3.05	0.692
Plasmapheresis	5	103	0.858	0.197–3.727		
Illegal drug injection	1	12	1.472	0.141–15.411		
Homosexual transmission	3	124	0.427	0.084–2.187		
Heterosexual transmission	7	262	0.472	0.118–1.884		
Unknown	2	33	1.071	0.170–6.750		
**Duration of treatment (year)**						
<1	4	117	1.000		2.4	0.494
1-	10	236	1.239	0.381–4.036		
3-	2	128	0.457	0.082–2.542		
5 and above	5	106	1.380	0.361–5.273		
**Treatment regimen**						
AZT/d4T+3TC+NVP/EFV	15	540	1.000	2.196–16.39	10.115	0.006
AZT/d4T/ TDF +3TC+LPV/r	6	36	6.000	3		
TDF+3TC+EFV/NVP	0	9	0.000	0		

OR: odds ratio; CI: confidence interval

**Table 4:** Analysis of impact factors for drug resistance in 2012.

Variables	Drug resistance		OR value	OR value 95% CI	χ^2^value	P value
	Yes	No				
**Age (years)**						
<30	6	170	1.000		1.65	0.648
30-	10	316	0.897	0.320–2.509		
40-	8	274	0.827	0.282–2.425		
50-	7	125	1.587	0.520–4.837		
**Gender**						
Male	18	616	1.000		1.872	0.171
Female	13	269	1.654	0.799–3.424		
**Ethnicity**						
Han	28	722	1.000		1.542	0.214
Other	3	163	0.475	0.143–1.580		
**Marital status**						
Single	3	210	1.000		4.418	0.22
Married	22	516	2.984	0.884–10.078		
Divorced or widowed	6	154	2.727	0.672–11.075		
Other	0	5	0.000	0		
**Educational level**						
Illiterate	5	152	1.000		1.04	0.904
Elementary	6	164	1.112	0.333–3.719		
Junior high school	11	275	1.216	0.415–3.565		
Senior high school and secondary school	6	156	1.169	0.349–3.912		
College and above	3	138	0.661	0.155–2.817		
**Route of infection**						
Blood transfusion	1	60	1.000		6.831	2.234
Plasmapheresis	5	108	2.778	0.317–24.331		
Illegal drug injection	1	24	2.500	0.150–41.605		
Homosexual transmission	5	258	1.163	0.133–10.137		
Heterosexual transmission	19	403	2.829	0.372–21.519		
Unknown	0	32	0.000			
**Duration of treatment (year)**						
<1	9	230	1.000		2.751	0.432
1-	11	276	1.019	0.415–2.501		
3-	9	220	1.045	0.407–2.682		
5 and above	2	159	0.321	0.069–1.508		
**Treatment regimen**						
AZT/d4T+3TC+NVP/EFV	24	810	1.000		7.808	0.02
AZT/d4T/ TDF +3TC+LPV/r	3	53	1.910	0.557–6.549		
TDF+3TC+EFV/NVP	4	22	6.429	2.048–20.176		

OR: odds ratio; CI: confidence interval

**Table 5:** Analysis of impact factors for drug resistance in 2013.

Variables	Drug resistance		OR value	OR value 95% CI	χ^2^ value	P value
	Yes	No				
**Age (years)**						
<30	13	241	1.000		1.672	0.643
30-	19	427	0.852	0.4–1.7		
40-	12	353	0.630	0.283–1.405		
50-	10	184	1.008	0.432–2.349		
**Gender**						
Male	38	890	1.000		0.325	0.569
Female	16	315	1.190	0.645–2.163		
**Ethnicity**						
Han	47	1077	1.000		0.296	0.587
Other	7	128	1.253	0.555–2.831		
**Marital status**						
Single	16	326	1.000		0.308	0.875
Married	31	695	0.909	0.490–1.685		
Divorced or widowed	7	184	0.775	0.313–1.919		
**Educational level**						
Illiterate	10	147	1.000		3.768	0.438
Elementary	10	206	0.714	0.290–1.758		
Junior high school	19	378	0.739	0.336–1.627		
Senior high school and secondary school	8	239	0.492	0.190–1.275		
College and above	7	235	0.438	0.163–1.176		
**Route of infection**						
Blood transfusion	1	71	1.000		6.481	0.262
Plasmapheresis	4	112	2.536	0.278–23.147		
Illegal drug injection	1	26	2.731	0.165–45.266		
Homosexual transmission	19	428	3.152	0.415–23.915		
Heterosexual transmission	29	531	3.878	0.520–23.905		
Unknown	0	37	0.000	0		
**Duration of treatment (year)**						
<1	13	211	1.000		2.488	0.477
1-	21	438	0.778	0.382–1.584		
3-	13	330	0.639	0.291–1.406		
5 and above	7	226	0.503	0.197–1.284		
**Treatment regimen**						
AZT/d4T+3TC+NVP/EFV	35	948	1.000		9.896	0.007
AZT/d4T/ TDF +3TC+LPV/r	11	84	3.547	1.738–7.238		
TDF+3TC+EFV/NVP	8	172	1.260	0.575–2.762		

OR: odds ratio; CI: confidence interval

### 3. Effect of drug resistance testing

The treatment regimens for 77 patients in the 106 drug-resistant patients from 2011 to 2013 were immediately adjusted based on their drug resistance testing results. Meanwhile, treatment remained unchanged for the other 29 patients because of death (2 patients), less than 1 year of first line treatment (8 patients), untimely adjustment of treatment regimen (15 patients), and use of second line drugs (4 patients). Drug resistance testing in the next year showed that the improvement rate of the 77 patients who received treatment adjustments was up to 89.6% (69/77), and only 8 patients retained drug resistance. On the other hand, among the 27 patients without treatment adjustments (excluding the 2 patients who died), the drug resistance improvement rate was 7.4% (2/27), where the 2 patients who showed improvements had only undergone less than a year of first line treatment. Statistical analysis on the adjusted and the unadjusted groups showed *χ*^*2*^ = 62.4, P<0.05, indicating that drug resistance can be significantly improved if treatment regimen is adjusted in a timely manner following drug resistance testing.

## Discussion

### 1. Method of drug resistance detection

Genotyping and phenotyping are the two major methods for drug resistance detection. Only genotyping was used in the present study. According to previous studies, the two methods could generate highly consistent results. Comparison of drug resistance genotyping vs. Pheno Sense TM phenotyping by Dunne et al. [10] demonstrated that the consistency of resistance detection between the two methods was up to 81% for NRTIs, up to 91% for NNRTIs, and up to 90% for PIs. Moreover, the results were found to be completely consistent between the two methods in 17% of the patients. The presence of antagonistic and complex mutations may lead to inconsistent results where resistance is confirmed by genotyping, but not by phenotyping. In addition, a limitation of genotyping is the presence of mutations that has not been mentioned or explained in the HIV Drug Resistance Database by Stanford University.

### 2. Subjects

Drug-resistant patients who were included in this study accounted for 99.67% (608/610), 99.56% (916/920), and 99.52% (1259/1265) of the total patients in Shandong Province per year from 2011 to 2013, respectively, who met the criteria of this study. Although there was a continuity among the patients during the 3-year period of examination, 36 patients from 2011 (5 died, 18 discontinued ART and others were transferred) did not continue ART in 2012, and 62 patients from 2012 (7 died, 30 discontinued ART and others were transferred) did not continue ART in 2013. It was important to note that death, withdrawal, and transfer have resulted in certain biases in the current study.

### 3. Drug resistance

The rates of HIV drug resistance in Shandong per year from 2011 to 2013 were 3.45% (21/608), 3.38% (31/916), and 4.29% (54/1259), respectively, which are below the 5% of low level HIV drug resistance threshold set by the World Health Organization [11]. Therefore this indicates that levels of drug resistance were low in the province. These findings are similar or lower than those reported in other provinces of China (4.7% in Gansu, 5.16% in Hunan, and 7.7% in Zhejiang) [12–14]. *M184V* was identified to be the most frequent mutation conferring resistance to NRTIs. Previous studies have shown that *M184V* could result in high level resistance to 3TC and emtricitabine (FTC) [15,16]and low level resistance to didanosine (ddI) and ABC *in vitro* [17–19]. Interestingly, *M184V*could reduce pathogenicity of HIV, and increase susceptibility to other NRTIs [17, 18, 20]. So patients with the *M184V* mutation could remain 3TC in therapeutic regimen. In addition, the reason for keeping 3TC in the second line drug regimen is that the drug can be taken one time a day and reduce body load [9]. Similarly, *Y181C*, *G190A*, *K103N* and *V179D/E/F* were the most frequent mutations conferring resistance to NNRTIs, and also the most frequent mutations found among patients on ART[21–23]. *In vitro* studies have demonstrated that while *Y181C/I/V* could result in high level resistance to NVP and low level resistance to EFV [24], *G190A* led to high level resistance to NVP, and intermediate resistance to EFV [25]. In addition, *K103N* was shown to cause high level resistance to NVP and EFV [25], whereas *V179D/E* led to low level reductions in susceptibility to NVP and EFV [26]. Therefore, the presence of the above mutations could directly elevate the resistance rates to NVP and EFV in patients on ART. Among all the patients on ART, 106 were found drug-resistant during the 3-year period, and 104 of them were resistant to both EFV and NPV. The finding of high NVP- and EFV-resistance rates is consistent with those observed in other countries as well as other regions in China [23, 27]. Currently, NVP and EFV are the only NNRTIs in China’s large-scale ART program, so the prevention of the transmission of NNRTI-resistant HIV strains is a top priority in prevention and control of AIDS in Shandong Province. The present study was the first to identify the PI-resistance mutations *I54V* and *V82A* in Shandong, and hence PIs should be carefully selected when we develop treatment regimens for these patients.

There were two interesting points. First, there was no PIs mutation on patients with second line treatment. *M184V* and *Y181C* were also the most frequent point mutations conferring resistance to NRTIs and NNRTIs in those patients. Second, the *I54V* and *V82A* mutations about PIs discovered in the study were not found in patients on second line treatment, but found in two patients who remained on the first line treatment. The two patients need continuous study, especially investigate the source of their infection.

We also found that subtypes B and CRF 01-AE were the major HIV subtypes among drug-resistant patients, which is consistent with the molecular epidemiological finding that B, CRF01_AE, CRF07_BC and CRF08_BC are the major subtypes among individuals with AIDS in China [28, 29].

### 4. Risk factors for drug resistance

Drug resistance testing results could only be generated in 10 of the early samples from drug-resistant patients, possibly because of the degradation of nucleic acids after long-term storage. Most of the mutations of the 10 samples were polymorphisms or minor mutations. It is possible that these mutations occurred earlier than other mutations [30], or these mutations did not cause strong drug resistance and facilitated their transmission. These need to increase the number of samples in further research. A drug-resistant strain was identified in 2 of 10 patients (20%), which to some extent reflects the proportion of primary resistance in all resistance. Among all the current drug resistance related studies, the proportion of primary resistance in patients on ART has been rarely reported [31, 32]. We could only obtain a few references from the rate of drug resistance in drug naïve population. It was found that the prevalence of HIV drug resistance in some African regions and among untreated individuals with AIDS in China were 19.4% [33] and 3.8% [34], respectively. In addition, the prevalence of HIV drug resistance among drug naïve population in Shandong, Guizhou, Jilin and the Liangshanzhou region of Sichuan in 2009 were 3.5%, 3.6% [35], 2.7% [36], and 5.43% [37], respectively, with the latest having the highest prevalence of HIV drug resistance.

Among the five demographic parameters examined in 2011, which were age, gender, ethnicity, marital status and educational level, only educational level showed a low degree of correlation with drug resistance, and the other parameters had no effect on drug resistance. The emergence of drug resistance in patients with a low educational level might be caused by their poor adherence to medication. However, this finding was not observed in the 2012 and 2013 data, suggesting that it was possibly a result of statistical bias, and hence requires further analysis. Although age was not found to be a risk factor for drug resistance in this study, Yaxelis Mendoza et al. have shown that age may be a risk factor for drug resistance [27]. Moreover, despite the absence of a correlation between the route of transmission and drug resistance in the current study, drug resistance testing is especially important for male-male homosexual transmission as this population is active in high risk behaviors that accelerate the transmission of viral strains [38]. Once the resistant strains reach a certain level, they may be rapidly spread to other populations.

Antiretroviral (ARV) drug toxicity, discontinued or intermittent access to ARV drugs, suboptimal dosage and a long duration of virological failure in individuals on first line treatment are all factors associated with ARV drug resistance over time [39, 40]. Our findings showed a significantly high rate of drug resistance among patients with LPV/r. Firstly, it could be associated with poor ART adherence of patients. Since patients who failed on the first line treatment normally had poor ART adherence, they may remain poor ART adherence during second line treatment. Secondly, it could be due to the short duration of second line treatment, the effects of second line treatment may have not appeared on some drug resistant patients. It also explains why use of TDF is associated with higher rates of drug resistance though it has changed into first line in 2011.

It has been reported that ART adherence and subtype, which were not examined in this study, are associated with drug resistance. In particular, low ART adherence was highly correlated with the development of drug resistance [41]. A similar study we conducted in 2009 showed that ART adherence among AIDS patients in Shandong was as high as 94.75% [42], which was consistent with that among AIDS patients in central regions of China (94.75%, calculated from 1 week of ART) [35]. Sui H and Cabello M et al. have also demonstrated that HIV subtype was highly associated with the development and spread of drug resistance [43–48].

### 5. Positive role of drug resistance testing

The implementation of HIV drug resistance testing and the timely adjustment of ART regimen increased the treatment improvement rate up to 89.6% (69/77) in the current study. Among the 8 patients who retained drug resistance, the ART regimen of 4 patients had only been changed for less than 6 months, which was not long enough for the drugs to achieve full suppression of the resistant strains. We found that the introduction of drug resistance testing has improved the development of drug resistance and reduced the number of drug-resistant patients, and was thereby the key to maintain low HIV drug resistance in Shandong. A small number of drug-resistant patients and a low viral load lead to reduced transmission of HIV and drug-resistant strains, forming a positive feedback loop that greatly facilitates the prevention of AIDS. Furthermore, the maintenance of low level HIV drug resistance through drug resistance testing could also make the effects of various risk factors insignificant.

### 6. Limitations

Findings in this study may have some limitations. First, the study subjects were under the national free treatment, which has limited drug types. And there is no monitoring on drug resistance carried out for patients before ART. Second, our study subjects are patients who had relatively high ART adherence. We were not able to bring those undetected AIDS patients as well as patients who had worse ART adherence into the study. These two limitations may have some impacts on the conclusion.

## Conclusions

In summary, the prevalence of HIV drug resistance is low in Shandong Province. Among the risk factors for drug resistance, primary resistance accounted for 20% of the total cases of resistance, while age, gender, ethnicity, marital status, route of transmission and duration of treatment had no significant effect on drug resistance. However, patients with a low educational level and those undergoing second line treatment should be continuously monitored, and active participation in drug resistance testing could effectively prevent the development and spread of drug resistance.

## Supporting information

S1 File 2011 nucleic acid sequences. (PDF)

S2 File 2012 nucleic acid sequences. (PDF)

S3 File 2013 nucleic acid sequences. (PDF)
