# Supplementary material for: HIV drug resistance in HIV positive individuals under antiretroviral treatment in Shandong Province, China
Source: PLoS One. 2017 Jul 27;12(7):e0181997. doi: 10.1371/journal.pone.0181997 (PMC5531464; doi:10.1371/journal.pone.0181997)
Supplement: S1 File — (PDF) [file pone.0181997.s001.pdf]

>13
CCTCAAATCACTCTTTGGCAACGACCCCTCGTCACAATAAAGATAGGrGGGCAAkTAAAG
GAAGCYCTATTAGATACAGGAGCAGATGATACAGTATTAGAAGACATGAATTTrCCAGGA
AGATGGAGACCAAAAATGATAGGGGGAATTGGAGGTTTTATCAAAGTAARACAGTATGAT
CAGATACCCATAGAAATCTGTGGACACAAGGCTGTAGGTACAGTATTAATAGGACCyACA
CCTrTCAACATAATTGGGAGAAAyyTGTTGACTCArCTTGGTTGyACTTTAAATTTTCCT
ATTAGTCCTATTGAAACTGTACCAGTAAAATTAAAGCCAGGAATGGATGGCCCAAAAGTT
AArCAATGGCCATTGACAGAAGAAAAAATAAAAGCmTTAGTAGAAATTTGTACAGAAATG
GAAAAGGAAGGrAAAATTTCAAAAATTGGGCCTGAAAATCCATAyAATACTCCAGTATTT
GCCATAAAGAAAAAAGACAGTACTAAATGGAGAAAATTAGTAGATTTCAGGGAACTTAAT
AAAAGAACTCAAGACTTCTGGGAAGTyCAATTAGGAATACCACATCCyGCAGGGTTAAAA
AAGAAAAAATCTGTAACAGTCCTGGATGTGGGTGATGCATAYTTCTCAGTYCCTTTAGAT
AAAGAmTTCAGGAAGTAyACTGCATTTACCATACCTAGTGTAAACAATGAGACACCAGGG
ATCAGATATCAGTACAATGTrCTTCCACAGGGATGGAAAGGATCACCAGCAATATTCCAA
TGTAGTATGACAAAAATCTTAGAGCCTTTTAGrmAACAAmATCCAGACATrRTyATCTAT
CAATAyATGGATGATTTGTATGTAGGATCTGACTTAGAAATAGGGCAGCATAGAGCAAAA
ATAGAGGAACTGAGAGAACATCTGTTGAGGTGGGGATTTACCACACCAGACAAAAAACAT
CAGAAAGAACCTCCATTCCTTTGGATGGGTTATGAACTCCATCCTGATAAATGGACAGTA
CAGCCTATAGTGCTGCCAGAAAAGGACAGCTGGACTGTCAATGACATACAGAAGTTAGTG
GGAAAGTTGAATTGGGCAAGTCAAATTTATsCAGGGATyAAArTAAGGGAATTATGTAAA
CTCATTAGGGGAACCAAAGCACTAACAGAAGTAATACCACTAACAGAAGAAGCAGAG

>16
CCTCAAATCACTCTTTGGCAACGACCCTTAGTCACGATAAAAATAGGGGGGCAATTAAAG
GAAGCTCTATTAGATACAGGAGCAGATGATACAGTAATAGAAGACATGAATTTGCCAGGA
AGATGGAAACCAAAAATGATAGGGGGAATTGGAGGTTTATCAAAGTAAGACAGTATGATC
AGATACCCATAGAAATCTGCGGACACAAGACTGTAGGTACAGTATTAATAGGACCTACAC
CTGTCAACATAATTGGAAGAAATCTGTTGACTCAACTTGGTTGCACCCTCAATTTTCCCA
TTAGTCCTATTGAAACTGTACCAGTAAAATTAAAGCCAGGAATGGATGGCCCAAAAGTTA
AACAATGGCCATTGACAGAAGAAAAAATAAAAGCCTTAGTAGAAATTTGTACAGAACTGG
AAAAGGAAGGAAAAATTTCAAAAATTGGGCCTGAAAATCCATATAATACTCCAGTATTTG
CCATAAAGAAAAAAGACAGTACTAAATGGAGAAAATTAGTAGATTTCAGGGAACTTAATA
AAAGAACTCAAGACTTTTGGGAAATTCAATTAGGAATACCACATCCCGCAGGATTACCAA
AGAACAAATCTGTAACAGTCCTGGACATTGGTGATGCATATTTCTCAGTCCCTTTAGATA
AAGACTTCAGGAAGTATACTGCATTTACCATACCTAGTATAAACAATGAGACACCAGGAA
TTAGATATCAGTACAATGTGCTTCCACAGGGATGGAAAGGATCACCAGCAATATTCCAAA
GTAGCATGACAAAAATCTTAGAGCCTTTTAGAAAACAAAATCCAGATATAGTTATCTATC
AATACGTGGATGATTTGTATGTAGGATCTGACTTAGAAATAGGGCAGCATAGAGCAAAAA
TAGAGGAACTGAGACAACATCTGTTGAGGTGGGGGTTTTTTACACCAGATAAAAAACATC

AGAAAGAACCTCCATTCCTTTGGATGGGTTATGAACTCCATCCTGATAAATGGACAGTAC
AGCCTATAGTGCTGCCAGAAAAGGACAGCTGGACTGTCAATGACATACAGAAGTTAGTGG
GAAAGTTAAATTGGGCAAGTCAGATTTATGCAGGGATTAAGACAAGGGAATTATGTAAAC
TCCTTAGGGGAACCAAAGCACTAACAGAAGTAATACCACTAACAGAAGAAGCAGAGC

>25
CCTCAAATCACTCTTTGGCAACGACCCCTCGTCCCAATAAGGATAGGGGGGCAATTAAAG
GAAGCTCTATTAGATACAGGAGCAGATGATACAGTATTAGAAGAAATGAATTTGCCAGGA
AGATGGAAACCAAAAATGATAGGGGGAATTGGAGGTTTTATCAAAGTAAGACAATATGAT
CAGATACCCATAGAAATCTGTGGACACAAGACTGTTGGTACAGTGTTAATAGGACCCACA
CCTGTCAACATAATTGGAAGAAATCTGTTGACTCAGCTTGGTTGCACCyTAAATTTTCCC
ATTAGTCCTATTGAAACTGTACCAGTAAAATTAAAGCCAGGAATGGATGGCCCAAAAGTT
AAACAATGGCCATTGACAGAAGAAAAAATAAAAGCATTAGTAGAAATTTGTACAGAAATG
GAAAAGGAAGGGAAAATTTCAAAAATCGGGCCTGAAAATCCATACAATACTCCAGTATTT
GCAATAAAGAArAAAAACAGTACTAGGTGGAGAAAATTAGTAGATTTCAGGGAACTTAAT
AAAAGAACTCAAGACTTCTGGGAAGTTCAATTAGGAATACCACATCCCGCrGGGTTAGAA
AAGAAAAAATCkGTAACAGTGCTGGATGTGGGTGATGCATATTTCTCAGTTCCTCTAGAT
AAAGATTTYAGRAAATATACTGCATTTACCATACCTAGTACAAACAATACGACACCAGGA
ATCAGATATCAGTACAATGTGCTTCCGCAGGGATGGAAAGGATCACCAGCAATATTCCAA
AGTAGCATGACAAAAATCTTAGAGCCTTTTAGAAAACAAAATCCAGACATAGTTATCTGT
CAATAyGTGGATGATTTGTATGTAGGATCTGACTTAGAAATAGGGCAGCATAGAGTAAAA
GTAGAGGrACTAAGACAACATCTGTTGAGGTGGGGATTTACCACACCAGACCAAAAATAT
CAGAAAGAACCTCCATTCCTTTGGATGGGTTATGAACTCCATCCAGATAAATGGACAGTA
CAGCCTATAGTGCTGCCAGAAAAGGACAGCTGGACTGTCAATGACATACAGAAGTTAGTG
GGAAAGTTGAATTGGGCAAGCCAGATTTATGCAGGGATTAAGGTAAAGGAGTTATGTAAA
CTCCTTAGGGGAACCAAAGCACTAACAGAAGTAATACCACTAACAGAAGAAGCAGAG

>28
CCTCAAATCACTCTTTGGCAACGACCCCTCGTCACAATAAArATAGGGGGGCAAyTAAAG
GAAGCTCTATTAGATACAGGrGCAGATGATACAGTATTAGAAGAAATGAATTTGCCAGGA
AAATGGAAACCAAAAATGATAGGGGGAATTGGAGGTTTTATCAAAGTAAGACAGTATGAT
CAGrTATCyATAGAAATyTGTGGACACAAGACTGTAGGTACAGTATTAATAGGACCTACA
CCTGTCAACATAATTGGGAGAAATCTGTTGACACAGCTTGGTTGCACkTTAAATTTTCCC
ATTAGTCCTATTGAAATTGTACCAGTAAAATTAAArCCAGGAATGGATGGCCCAAAAGTT
AAACAATGGCCATTGACAGAAGAAAAAATAAAAGCATTAGTAGAAATTTGTACAGAACTG
GAAAAGGAAGGGAAAATTTCAAAAATTGGGCCTGAAAATCCATACAATACTCCAGTATTT

GCCATAAAGAAAAAAGACAGTACTAAATGGAGAAAATTAGTAGATTTCAGGGAACTTAAT
AAAAGAACTCAGGACTTCTGGGAAGTTCAATTAGGAATACCACATCCyGCAGGATTAAAA
AAGAAAAArTCTGCAACAGTCCTGGATGTGGGTGATGCATATTTCTCAGTTCCTTTrGAT
rAAGACTTCAGGAAATATACTGCATTTACCATACCTAGkGTAAACAATGAGACACCAGGG
ATCAGATATCAGTACAATGTGCTTCCACAGGGATGGAAAGGATCACCAGCAATATTCCAA
TGTAGTATGACAAAAATCTTAGAGCCTTTTAGAAAACAAAATCCAGACATAGTTATCTAT
CAATACGTGGATGATTTGTATGTAGCATCTGACTTAGAAATAGGGCAGCATAGAGCAAAA
ATAGAGGAACTGAGACAGCATTTrTGGAGGTGGGGATTTTACACACCAGACAAAAAACAT
CAGAAAGATCCTCCACTTCGTTGGATGGGTTATGArCTCCATCCTGATAAATGGACAGTA
CAGCCTATAGTGCTGCCAGAAAAGGACAGCTGGACTGTCAATGACATACAGAAGTTAGTG
GGAAAATTAAATTGGGCAAGyCAGATTTATGCAGGGATTAAGGTAAAGGAATTATGTAAA
CTCATTAGGGGAACCAAAGCACTAACAGAGGTAGTACCACTAACAGAAGAAGCAGAG

>49
CCTCAAATCACTCTTTGGCAACGACCCCTCGTCACAATAAGGATAGGGGGGCAATTAAAG
GAAGCTyTATTAGAyACAGGAGCAGATGATACAGTATTAGAAGACATGAATTTGCCAGGA
ARATGGAAACCAAAAATGATAGGGGGAATTGGAGGTTTTATCAAAGTAAGACAGTATGAb
sAGrTrCCCnTAGAAATyTGCGGACACAAGGCTGTAGGTACAGTATTAATAGGACCTACA
CCTGTCAACATAATTGGGAGAAATyTGTTGACTCAGCTTGGrTGyACTTTAAATTTTCCC
ATTAGTCCTATTGAAACTGTrCCAGTAAAATTAAAGCCAGGAATGGATGGCCCAAAAGTT
AAACAATGGCCATTGACAGAAGAAAAAATAAAAGCATTAGTAGAAATTTGyrCAGAAATG
GAAAAGGAAGGGAAAATTTCAArAATTGGGCCTGAAAATCCATACAATACyCCAGTATTT
GCCATAAAGAAAAArGACAGTACTAAATGGAGAAAATTAGTAGATTTCAGrGAACTkAAT
AAAAGAACTCAAGAyTTCTGGGAAGTTCAATTAGGAATACCACATCCCGCrGGGTTAAAA
AAGAAAAAATCyGTAACmRTyCTGGATGTGGGTGATGCATAYTTCTCAGTyCCTTTAGAT
AAAAACTTCAGGAAGTATACTGCATTTACCATACCTAGTGTAAACAATGArACACCAGGr
ATCAGrTAyCAGTACAATGTGCTTCCACAGGGATGGAAAGGATCACCAGCAATATTCCAA
TGTAGTATGACAAAAATCTTAGAGCCTTTTAGrAAACAAAATCCAGAAATAGTTATCTAT
CAATACATGGATGATTTGTATGTAGGATCTGACTTAGAAATAGGGCAGCATAGAGCAAAA
ATAGAGGAACTrAGACAACATTTGTTGAGsTGGGGATTCACCACACCAGACAAAAAACAT
CAGAAAGAACCTCCATTCCTTTGGATGGGTTATGAACTCCATCCTGATAAATGGACAGTA
CAGCCTATAGTGCTGCCAGAAAAGGACAGCTGGACTGTCAATGACATACAGAAGTTAGTG
GGAAAGTTGAATTGGGCAAGTCAGATTTATGCAGGGATTAAGGTAAGGGAATTATGTAAr
CTCCTTAGGGGAACCAAAGCACTAACAGAAGTAGTACCACTAACAGAAGAAGCAGAG

>57

CCTCAAATCACTCTTTGGCAACGACCCmTCGTCACArTAAAGATAGGGGGGCAATTAAAG
GAAGCTCTATTAGATACAGGAGCAGATGATACAGTATTAGAAGACATGAATTTGCCAGGA
AGATGGAAACCAAAAATGATAGGGGGAATTGGAGGTTTTATCAAAGTAAGACAGTATGAT
CAGrTRTCCWTrGAmATCTGyGGACACAArGyTGTAGGTACAGTATTAATAGGACCTACA
CCTrTCAACATAATTGGAAGrAATCTGTTGACTCArMTTGGTTGCACTTTAAATTTkCCC
ATTAGTCCTATTGAAACTGTACCAGTAAAATTAAAGCCAGGrATGGATGGCCCAAAAGTT
AAACAATGGCCATTGACAGAAGAAAAAATAAAAGCATTAGTAGAAATTTGTACAGAAATG
GAAAAGGAAGGrAAAATyTCAAAAATyGGGCCTGAAAAYCCATACAATACTCCAGTATTT
GCCATAAAGAAAAAAGACAGTACAAAATGGAGAAArTTAGTAGATTTCAGGGAACTTAAT
AAAAGAACTCAAGACTTCTGGGAAGTTCAATTAGGAATACCACATCCCGCAGGGTTAAAA
AAGAAAAAATCAGTrACAGTCCTGGATGTGGGTGATGCATATTTyTCAGTyCCTTTAGAT
RAAGAYTTCAGGAAATATACTGCATTTACCATACCTAGTGTAAACAATGAGACwCCAGGG
ATCAGATATCAGTACAATGTGCTTCCACAGGGATGGAAAGGATCACCAGCAATATTCCAA
TGTAGCATGACAAArATCTTAGAGCCTTTTAGAAAACAAAATCCAGAyATAGTTATCTAT
CAATACATGGATGATTTGTATGTAGGATCTGACTTAGAAATAGrACAGCATAGAGCAAAA
ATAGAGGAACTrAGACAACATyTGTTGGGGTGGGGATTTACCACACCAGACAAAAAACAT
CAGAAAGAACCTCCATTCCTTTGGATGGGTTATGAACTCCATCCTGATAAATGGACAGTA
CAGCCTATAGTGCTGCCAGAAAAGGACAGCTGGACTGTCAATGACATACAGAAGTTAGTG
GGrAAATTGAATTGGGCAAGTCAGATTTATCCAGGGATTAGAGTAAGGGAATTATGTAAA
CTCCTTAGGGGAACCAAAGCACTAACAGAAGTAATACCACTAACAGAAGAAGCAGAG

>62
CCTCAGATCACTCTTTGGCAACGACCCCTCGTCACAATAAAGATAGGGGGGCAATTAAAG
GAAGCTCTATTAGATACAGGAGCAGATGATACAGTATTAGAAGACATGAATTTGCCAGGA
AGATGGAAACCAAAAATGATAGGGGGAATTGGAGGTTTTATCAAAGTAAGACAGTATGAT
CAAGTAGCTATAGAAATCTGTGGCCACAAGGCTGTAGGTACAGTATTAATAGGACCTACA
CCTGTCAACATAATTGGAAGGAATCTGTTGACTCAGATTGGGTGCACTTTAAATTTTCCC
ATTAGTCCTATTGAAACTGTACCTGTAAAGTTAAAGCCAGGAATGGATGGCCCAAAAGTT
AAACAATGGCCACTGACAGAAGAAAAAATAAAAGCATTAGTAGAAATTTGTACAGAAATG
GAAAAAGAGGGGAAAATTTCAAAAATCGGGCCTGAAAATCCATACAATACTCCAGTATTT
GCCATAAAGAAAAAAGACAGTACTAAATGGAGAAAATTAGTAGATTTCAGGGAACTTAAT
AAAAGAACGCAGGACTTCTGGGAAGTTCAATTAGGAATACCACATCCCGCAGGGTTAAAA
CAGAAAAAATCAGTAACAGTCCTGGATGTGGGTGATGCATATTTCTCAGTCCCTTTAGAT
AAGGACTTCAGGAAGTATACTGCATTCACCATACCTAGTGTAAACAATGAGACACCAGGG
ATCAGATATCAGTACAATGTGCTTCCACAGGGATGGAAAGGATCACCAGCAATATTCCAA
TGTAGCATGACAAAAATCTTAGAGTCTTTTAGAAAACAAAATCCAGACATAGTTATCTAC
CAATACGTGGATGATTTGTATGTAGGATCTGACTTAGAAATAGGGCAACATAGAGAAAAA
ATAGAGGAACTGAGACAACATTTGTTGAGGTGGGGATTTACCACACCAGACAAAAAACAT
CAGAAAGAACCTCCATTCCTTTGGATGGGTTATGAACTCCATCCTGATAAATGGACAGTA
CAGCCTATAGTGCTGCCAGAAAAGGACAGCTGGACAGTCAATGACATACAGAAGTTAGTG

GGAAAGTTGAACTGGGCAAGTCAGATTTATGCAGGAATTAAGATAAAGGAATTATGTAAA
CTCCTTAGGGGAACCAAAGCACTAACAGAAGTAATACCACTAACAGAAGAAGCAGAG

>83
CCTCAAATCACTCTTTGGCAACGACCCCTTGTTACCATAAAAGTAGGAGGACAGCTGAGA
GAAGCTCTATTAGATACAGGAGCAGATGATACAGTATTAGAAGACATAAATTTGCCAGGA
AAATGGAAACCAAAAATGATAGGGGGAATTGGAGGTTTTATCAGGGTAAGGCAATATGAT
CAGATACTTATAGAAATTTGTGGAAAAAAGGCTATAGGTACAGTGTTAGTAGGACCTACA
CCTGTCAACATAATTGGACGAAATATGTTGACTCAGCTTGGTTGTACTTTAAATTTTCCA
ATCAGTCCTATTGACACTGTACCAGTAAAATTAAAACCAGGAATGGATGGACCAAAGGTT
AAACAATGGCCATTGACAGAAGAAAAAATAAAAGCATTAACAGAAATTTGTAAAGAGATG
GAAGAGGAAGGAAAAATTTCAAAAATTGGGCCTGAAAACCCATACAATACTCCAGTATTT
GCTATAAAGAAAAAGGATGGCACCAAATGGAGGAAAkTAGTAGATTTCAGAGAGCTTAAT
AAAAGGACACAGGATTTTTGGGAAATTCAATTAGGAATACCGCATCCAGCAGGyTTAsAA
AAGAAAAAATCAGTAACAGTACTAGATGTGGGAGATGCATATTTTTCAGTyCCATTAGAT
AAAGACTTTAGAAAGTATACTGCATTCACCATACCTAGTATAAACAATGAGACACCAGGA
ATCAGATATCAGTACAATGTGCTACCACAGGGATGGAAAGGATCACCAGCAATATTCCAG
AGTAGCATGACAAGAATCTTAGAGCCCTTTAGAATAAAAAATCCAGAATTAACTATCTGT
CAATACATGGATGACTTGTATGTATCATCTGATTTAGAAATAAAACAGCATAGAGCAAAA
ATAGAGGAGCTAAGAGCTCATCTATTGAACTGGGGATTTACTACACCAGACAAAAAGCAT
CAGAAGGAACCGCCTTTCCTTTGGATGGGATATGAACTCCATCCTGACAAATGGACAGTC
CAGCCTATACAACTGCCAGAAAAAGACAGCTGGACTGTCAATGATATACAGAAATTAGTG
GGAAAACTAAATTGGGCAAGTCAGATTTATGCAGGGATTAAGATAAAACAACTGTGTAAA
CTTCTCAGGGGAACTAAAACACTAACAGAAATAGTACCACTGACTGCAGAAGCAGAG

>86
CCTCAAATCACTCTTTGGCAACGACCCCTTGTCACAATAAAGATAGGGGGGCAATTAAAG
rAAGCTCTATTAGATACAGGAGCAGATGATACAGTATTAGAAGACATGAATTTGCCAGGA
ArATGGAAACCAAAAATGATAGGGGGAATTGGAGGTTTTATCAAAGTAAGACAGTATGAT
CAGATACCCATAGAAATCTGTGGACACAAGGCTGTAGGTACAGTATTAATAGGACCTACA
CCTGTCAACATAATTGGrAGAAATCTGTTGACTCAACTTGGGTGyACTTTAAATTTTCCC
ATTAGTCCTATTGAAACTGTACCAGTAAAATTAAAGCCAGGAATGGATGGCCCAAAAGTy
AAACAATGGCCATTGACAGAAGAAAAAATAAAAGCATTAGTAGAAATTTGTACAGAAATG
GAAAAGGAAGGGAAAATTTCAAAAATCGGGTCTGAAAATCCATACAATACTCCAGTATTT
GCCATAAAGAAAAAAGACAGTACTAAGTGGAGAAAGTTAGTAGATTTCAGGGAACTTAAT
AAAAGAACTCAAGACTTCTGGGAAGTTCAATTAGGAATACCACATCCCGCAGGGTTAAAA

AAGAAAAAAATCTGTAACAGTCCTGGATGTGGGTGATGCATACTTCTCAGTCCCTTTAGAT
AAAGACTTCAGGAAGTATACTGCATTTACCATACCTAGTGTAAACAATGAGACACCAGGG
ACCAGATACCAGTACAATGTGCTTCCACAGGGATGGAAAGGATCACCAGCAATATTCCAA
TGTAGCATGACAAAAATCTTAGAGCCTTTyAGAAAACAAAATCCAGACATAGTTATCTAT
CAATACATGGATGATTTGTAyGTAGGATCTGACTTAGAAATAGGGCAGCATAGAGCAAAA
ATAGAGGAACTsAGACAGCATCTGTTGAAGTGGGGATTTACCACACCAGATAAAAAACAT
CAGAAAGAACCTCCATTCCTTTGGATGGGGTATGAACTCCATCCTGAyAAATGGACAGTA
CAGCCTATAGTGCTGCCAGAAAAGGACAGCTGGACTGTCAATGACATACAGAAGTTAGTG
GGAAAGTTGAATTGGGCAAGyCAGATTTATGCAGGGATYAGGGTAAAGGAATTATGTAAA
CTCCTTAGGGGAACCAAAGCACTAACAGAAGTAGTACCACTAACAGAAGAAGCAGAG

>114
CCTCAAATCACTCTTTGGGAACGACCCCTCGTCACAATAAAGATAGGGGGGCAATTAAAA
GAAGCTCTATTAGATACAGGAGCAGATGATACAGTATTAGAAGAAATGAATTTGCCAGGA
AGATGGAAACCAAAAATGATAGGGGGAATTGGAGGTTTTATCAAAGTAAAACAGTATGAT
CAGATACCCATAGAAATCTGTGGACACAAGACTGAAGGTACAGTATTAATAGGACCTACA
CCTGTCAACATAATTGGGAGAAATCTGTTGACTCAGCTTGGTTGCACTTTAAATTTTCCC
ATTAGTCCTATTGAAACTGTACCAGTAAAATTAAAGCCAGGAATGGATGGCCCGAGAGTT
AAACAATGGCCATTGACAGAAGAAAAAATAAAGGCCTTAGTAGAAATTTGTACAGAAATG
GAAAArGAAGGGAAAATTTCAAAAATTGGGCCTGAAAATCCATACAATACTCCAGTMTTT
GCyATAAAGAAAAArGACAGTACTAAATGGAGAAAATTAGTAGATTTTAGGGAACTTAAT
AAAAGAACTCAAGACTTCTGGGAAGTTCAATTAGGAATACCACATCCCGCAGGGTTAAAA
AAGAGAAAATCAGTAACArTCCTGGATGTGGGTGATGCATATTTCTCAGTTCCTTTAGAT
AAAGACTTCAGGAAGTATACTGCATTTACCATACCTAGTGTAAACAATGAGACACCAGGG
ATyAGATATCAGTACAATGTACTCCCACAGGGGTGGAAAGGATCACCAGCAATATTCCAA
TGTAGCATGACAAAAATCTTAGAGCCTTTTAGAAAACAAAATCCAGATATAGACATCTGT
CAATACGTGGATGATTTGTATGTAGGATCTGACTTAGAAATAGGGCAGCATAGAGCAAAA
ATAGAGGAACTGAGACAACATCTGTTGAGGTGGGGATTTACCACACCAGACAAAAAAyAT
CAGAAAGAACCTCCATTTCTTTGGATGGGTCATGAACTCCATCCTGATAAATGGACAGTA
CAGCCTATAGAGCTACCAGAAAAGGACAGCTGGACTGTCAATGACATACAGAAGTTAGTG
GGAAAGTTGAATTGGGCAAGTCAAATTTATGCAGGGATTAAGGTAAGAGAATTATGTAAA
CTCATTAGGGGAACCAAAGCACTAACAGAAGTAGTACCACTAACAGAAGAAGCAGAG

>142
CCTCAAATCACTCTTTGGCAACGACCCCTCGTCACAATAAAGATAGGGGGGCAATTAAAG
GAAGCTCTATTAGATACAGGAGCAGATGATACAGTATTAGAAGACATGAATTTGCCAGGA

AGATGGAAACCAAAAATGATAGGGGGAATTGGAGGTTTTATCAAAGTAAGACAGTATGAT
CAGATACCCATAGAAATTTGTGGGCACAAGACTGTAGGTACAGTATTAGTAGGACCTACA
CCTGTCAACATAATTGGAAGAAATCTGTTGACTCAGATTGGTTGCACTTTAAATTTTCCC
ATTAGTCCTATTGAAACTGTATCAGTAAAATTAAAGCCAGGAATGGATGGCCCAAAAGTT
AAACAATGGCCATTAACAGAAGAAAAAATAAAAGCATTATCAGAAATTTGTACAGAAATG
GAAAAGGAAGGAAAAATTTCAAAAATCGGGCCTGAAAATCCATACAATACTCCAGTATTT
GCCATAAAGAAAAAAGACAGTACTAAATGGAGAAAATTAGTAGATTTCAGAGAACTTAAT
AAAAGAACTCAAGACTTCTGGGAAGTTCAATTAGGAATACCACATCCAGCAGGGTTAAAG
AAGAAAAAATCAGTGACAATACTGGATGTAGGTGATGCATATTTCTCAGTCCCTTTAGAT
AAAGACTTCAGGAAGTATACTGCATTTACCATACCTAGTGTAAACAATGAGACACCAGGG
ATCAGATATCAGTACAATGTGCTGCCACAGGGATGGAAAGGATCACCAGCAATATTCCAA
TGTAGCATGACAAAAATCTTAGAGCCTTTTAGAAAGCAAAATCCAGATATAGTTATCTGT
CAATACGTAGATGATTTGTATGTAGGATCTGACTTAGAAATAGGGCAACATAGAGCAAAA
ATAGAGGAACTAAGACAGCATCTGTTGAGGTGGGGATTTACCACACCAGACAAAAAATAT
CAGAAAGAACCTCCATTCCGTTGGATGGGTTATGAACTCCATCCTGATAAATGGACAGTA
CAGCCTATAGTGCTGCCAGAAAAGGACAGCTGGACTGTCAATGACATACAGAAGTTAGTG
GGAAAGTTGAATTGGGCAAGTCAGATTTATTCAGGGATTAAGGTAAGGGAATTATGTAAA
CTCATTAGGGGAACCAAAGCATTAACAGAAATAGTACCACTAACAGAAGAAGCAGAG

>181
ACAATGGCCATTGACAGAAGAAAAAATAAAAGCATTAGTAGAAATTTGTACAGAAATGGA
AAAAGAAGGGAAAATTTCAAAAATCGGGCCTGAAAATCCATACAATACTCCAGTATTTGT
CATAAAGAGAAAAGACAGTACTAAATGGAGAAAATTAACAGATTTCAGGGAACTTAATAA
AAGAACTCAAGACTTTTGGGAAGTTCAATTAGGAATACCACATCCAGCAGGGTTAGAAAA
GAAAAAATCTGTAACAGTCCTGGATGTGGGTGATGCATATTTCTCGGTCCCTTTAGATAA
AGACTTCAGGAAGTATACTGCATTTACCATACCTAGTATAAACAATGAGACACCAGGGAT
CAGATATCAGTACAATGTGCTTCCACAGGGATGGAAGGGATCACCAGCAATATTTCAGAG
TAGCATGACAAAAATCTTAGAGCCTTTTAGAAAACAAAATCCAGACATAGTTATCTGTCA
ATATATGGATGATTTGTATGTAAGCTCTGACTTAGAAATAGGGCAGCATAGAGCAAAAAT
AGAGGAACTGAGAAATCATCTGTTGAAGTGGGGATTTACCACTCCAGACAAAAAATATCA
GAAAGAACCTCCATTTCTTTGGATGGGTTATGAACTCCATCCTGATAAATGGACAGTACA
GCCTATACAGCTGCCAGAAAAGGACAGCTGGACTGTCAATGACATACAGAAGTTAGTGGG
AAAATTGAAGTGGGCAAGCCAGATCTATGCAGGGATTAAGGTAAGGGAATTATGTAAACT
CCTTAGGGGAACCAAAGCACTAACAGAAGTAGTACCACTAACAAAGGAAGCAGAG

>194
CCTCAAATCACTCTTTGGCArCGACCCCTCGTCACArTAAAAATAGGAGGGCAGCTAAAA
GAAGCTcTATTAGATACAGGAGCTGATGATACAGTATTAGAAGATATAAATTTGCCAGGA

AAATGGAAACCAAAAATGATAGGGGGAATTGGAGGTTTTATCAAAGTAAGACAGTATGAT
CAGGTAyCCATAGArATCTGTGGACACAAAGCTATrGGTACAGTATTAGTAGGACCTACA
CCTGTCAACATAATTGGACGAAATATGTTGACTCAAATTGGTTGTACwTTAAATTTTCCC
ATTAGTCCTATTGAAACTGTACCAGTAAAATTAAAGCCAGGAATGGATGGTCCAAAGGTT
AAACAGTGGCCATTGACAGAAGAAAAAATAAAAGCATTAACAGAAATTTGTAAAGArATG
GAArArGAAGGAAAAATyTCAAAAATyGGGCCTGAAAATCCATACAATACTCCAGTATTT
GCAATAAAGAAAAArGACAGTACyAAATGGAGAAAATTAGTAgATTTTAgAGAACTTAAT
AAAAGAACTCAAGACTTCTGGGAAGTTCAATTAAGAATACCACATCCCGCAGGGTTAAAA
AAGAAAAAATCTGTAACAGTCTTGGATATAGGTGATGCATATTTCTCAGTTCCTTTAGAT
GAAGAATTCAGGAAGTATACTGCATTTACCATACCTAGTGTAAACAATGAAACACCAGGG
GTTAGATATCAGTACAATGTGCTTCCACARGGATGGAAAGGATCACCAGCAATATTCCAA
TATAGCATGACAAAAATCTTAgAkCCTTTTAGAAAACAAAATCCAGACATArTTATCTAT
CAATACATGGATGATTTGTATGTAGGATCTGACTTAGAAATAGGRCAACaTAGAACAAAA
ATAGAgGAACTGAGACaACATCTGTTrAAATGGGgGyTkACCaCACCAGACAAAAAACAT
CAGAAAGArCCTCCATTCCTTTGGATGGGTTATGAGCTCCATCCTGATAAATGGACAGTA
CAGCCTATAGTGCTGCCAGAAAAAGACAGCTGGACTGTCAATGACATmCAGAAGTTAGTG
GGAAAGTTrAATTGGGCAAGTCAGATTTATGCAGGGATTAAAGTAAGrGAGTTATGTAAA
CTCCTTAGGGGAACCAAAGCACTAACAGAAGTAGTACCACTAACAGAAGAAGCAGAG

>236
CCTCAAATCACTCTTTGGCAACGACCCGTTGTCACAGTAAAAATAGGGGGACAGCTGAGA
GAGGCTCTATTAGATACAGGAGCAGATGATACAGTATTAGAAGAAATAGATTTGCCAGGr
AAATGGAAACCAAAAATGATAGGGGGAATTGGAGGTTTTATCAArGTwAGGCAATATGAT
CAGATACTTATAGAAATTTGTGGAAAAArrGCTATAGGTACAGTGTTAGTwGGACCTACA
CCTGTCAACATAATTGGRCGAAACATGTTGACTCAGATTGGTTGTACTTTAAATTTCCCA
ATTAGTCCTATTGACACTGTACCAGTAAAATTAAAGCCAGGAATGGATGGGCCAAAGrTT
AAACAGTGGCCmTTGACAGAAGAAAAAATAAAAGCATTAACAGAAATTTGTAArGAAATG
GAAGAGGAAGGAAAAATCTCAAAAATTGGGCCTGAAAATCCATATAATACTCCAGTATTT
GCTATAAAGAAAAAGGACAGTACCAAATGGAGGAAATTAGTAGACTTCAGAGArCTCAAT
AAAAGAACTCAGGAyTTTTGGGAAGTTCAATTAGGAATACCGCATCCAGCAGGTyTAmAA
AAGAAAAAATCAGTAACAGTACTAGATGTGGGAGATGCATATTTTTCAGTTCCATTAGAT
AAAGACTTTAGAAAGTATACTGCATTCACCATACCTAGTATAAACAAyGAGACACCAGGA
ATTAGATATCAGTACAATGTrCTGCCACAGGGATGGAAAGGATCACCAGCAATATTCCAG
TGTAGCATGACAAAAATCTTAGAACCCTTTAGAAGAAAAAATCCAGAAATAATTATCTAT
CAATACGTGGATGACTTGTATGTAGCATCTGATTTAGAAATAGGGCAGCACAGAGCAAAA
ATAGAGGAGCTAAGAGCTCATCTATTGAGCTGGGGATTTACTACACCAGACAAAAAGCAT
CAGAAGGAACCTCCATTCCTTTGGATGGGATATGAACTCCATCCTGACAGATGGACAGTC
CAGCCTATAGAACTGCCAGAAAArGACAGCTGGACTGTCAATGATATACAGAAATTAGTG
GGAAAACTAAATTGGGCAAGCCAGATCTATCCAGGGATTAGAATAAAGCAACTGTGTAAA
CTCCTCAGGGGAGCTAAAGCACTAACAGACATAGTACCACTAACTGAAGAAGCAGAG

>261

CCTCAAATCACTCTTTGGCAACGACCCCTTGTCACAGTAAAGATAGGGGGGCAACTAAAA
GAAGCyCTATTAGATACAGGAGCAGATGATACAGTATTAGAAGACATGAGTTTGCCAGGA
AGATGGAAACCAAAAATGATAGGGGGAATTGGAGGTTTTATCAAAGTAAGACAGTATGAT
CAGATACCCATAGAAATATGTGGACATAAAGCTATAGGCACAGTATTAATAGGACCTACA
CCTGTCAACATAATTGGAAGAAATCTGTTGACTCAGATTGGCTGTACCTTAAATTTTCCC
ATTAGTCCTATTAAAACTGTACCAGTAAAATTrAAACCAGGCATGGATGGCCCAAAAGTT
AAACAATGGCCrTTGACAGAAGAAAAAATAAAAGCATTAGTAGAAATTTGTACAGAAATG
GAAAAGGAAGGAAAAATTTCAAAAATTGGGCCTGAAAATCCATACAATACTCCAGTATTT
GCTATCAAGAAAAAAGACAGCACTAAATGGAGAAAATTAGTAGATTTCAGAGAACTTAAT
AAAAGAACTCAAGACTTCTGGGAGGTTCAATTAGGAATACCACATCCAGCAGGGTTAAAr
AAGAAAAAATCAGTAACAGTACTGGATGTGGGTGATGCATATTTTTCAGTTCCCTTAGAT
GAAGACTTCAGGAAGTATACTGCATTTACCATACCTAGTACAAACAATGAGACACCAGGG
ATTAGGTATCAGTATAATGTGCTTCCACAGGGATGGAAAGGATCACCAGCAATATTCCAA
AGyAGCATGACAAAAATCTTAGATCCTTTTAGAAAACAAAACCCAGACATAGTGATCTAT
CAATACATGGATGATTTGTATGTAGGATCTGACCTAGAAATAGGGCAACATAGAACAAAA
GTAGAGGAACTGAGACAACATCTGCTGAAGTGGGGGTTAACCACACCAGACAAAAAACAT
CAGAAAGAACCTCCATTCCTTTGGATGGGTTATGAACTCCATCCTGATAAATGGACAGTA
CAGCCTATAGTGCTACCAGAAAAGGACAGCTGGACTGTCAATGACATACAGAAGTTAATA
GGAAAATTGAATTGGGCAAGTCAAATTTATGCAGGGATTAAArTAAAGCATTTATGTAAA
CTCCTTAGGGGAACCAAAGCACTAACAGAAGTAGTACCACTAACAGAAGAAGCAGAG

>293

CCTCAGATCACTCTTTGGCAGCGACCCCTTGTCACAATAAGAGTAGGGGGCCAAATAAAA
GAGGCTCTCTTAGACACAGGAGCAGATGATACAGTATTAGAGGAAATAAATTTGCCAGGA
AAATGGAAACCAAAAATGATAGGrGGAATTGGAGGTTTTATCAAAGTAAGACAATATGAT
CAAATACCTATAGAAATTTGTGGGAAAAAAGCTATAGGTACAGTATTAGTGGGACCTACA
CCTGTCAACATAATTGGAAGAAATCTGTTGACTCAGCTTGGsTGCACTTTAAATTTTCCA
ATCAGTCCCATTGAAACTGTACCAGTAAAATTAAAGCCAGGAATGGATGGCCCAAAGGTT
AAACAATGGCCATTGACAGAAGAAAAAATAAAAGCATTAACAGmAATTTGTGAGGAAATG
GArAAGGAAGGAAAAATTACAAArATTGGGCCTGAAAATCCATATAACACTCCAATATTT
GCCATAAAAAAGAAGGACAGTACTAAGTGGAGAAAGTTAGTAGATTTCAGGGAACTCAAT
AAAAGGACTCArGATTTTTGGGAAGTTCAATTAGGAATACCACACCCAGCAGGGyTGAAA
ArGAArAAATCAGTGACAGTACTrGATGTGGGGGATGCATATTTTTCAGTTCCTTTACAT
GAAGACTTCAGGAAGTATACTGCATTCACCATACCTAGTACRAACAATGAAACACCAGGA
ATTAGGTATCAATATAATGTGCTTCCACAGGGATGGAAAGGATCACCAGCAATATTCCAA
TCTAGCATGATAAAAATCTTAGAGCCTTTTAGAAATCAAAATCCAGACATAGTTATCTAT

CAATATATGGATGACTTATATGTAGGATCwGACTTAGArATAGGGCAACATAGAGCAAAA
ATAGAGGAGTTAAGAGAACATCTGTTAAAGTGGGGATTTACCACACCAGACAAAAAACAT
CAGAAAGAACCTCCrTTTCTTTGGATGGGRTATGAACTCCATCCTGACAAATGGACAGTA
CAGCCTATACAGCTGCCAGAAAAGGATAGCTGGACTGTAAATGACATACAAAAGTTAGTG
GGAAAATTAAACTGGGCAAGTCAGATTTACCCAGGAATTAAAATAAAGCAACTTTGTAAA
CTCATTAGGGGAGCCAAAGCACTAACAGACATAGTACCACTAACTGrAGAAGCAGAG

>294
CCTCAAATCACTCTTTGGCAACGACCCCTTGTCACAATAAAAATAGAAGGACAGCTGAAA
GAAGCTCTATTAGATACAGGAGCAGATGATACAGTATTAGAAGATATAAATTTGCCAGGA
AAATGGAAACCAAAAATGATAGGGGGAATTGGAGGTTTTATCAAAGTAAGGCAATATGAT
CAGATACTTATAGAAATTTGTGGAAAAAGGGCTATAGGTACAGTATTAGTAGGACCTACA
CCTGTCAACATAATTGGACGAAATATGTTGACTCAGATTGGTTGTACTTTAAATTTCCCA
ATTAGTCCTATTGACACTGTACCAGTAAAATTAAAGCCAGGAATGGATGGGCCAAGGGTC
AAACAATGGCCATTGACAGAAGAAAAAATAAAAGCATTAATAGAAATTTGTAAACAGATG
GAAGAGGAAGGAAAAATCTCAAGAATTGGGCCTGAAAATCCATACAATACTCCAGTGTTT
GTTATAAAGAAAAAGAACAGCACCAAGTGGAGGAAATTAATAGATCTCAGAGAGCTCAAT
AAAAGAACTCAGGATTTTTGGGAAGTTCAATTAGGAATACCGCATCCAGCAGGCTTAAAA
AAGAAAAAATCAGTAACAATACTAGATGTGGGTGATGCATATTACTCAGTACCTTTAGAT
AAAGAGTTTAGAAAGTATACTGCATTCACCATACCTAGTATAAACAATGAGACACCAGGA
ATCAGATATCAGTACAATGTGCTGCCAATGGGATGGAAAGGATCACCAGCAATATTCCAG
AGTAGCATGACAAAAATCTTAGAGCCCTTTAGAACAAAAAATCCAGAAATAATTATCTGT
CAATACGTGGATGACTTGTATGTGGCATCTGATTTAGAAATAGGGCAGCACAGAACAAAA
GTAGAGGAGCTAAGAGCTCATCTATTGAGCTGGGGACTTACTACCCCAGACGAAAAGCAT
CAGAAGGAACCTCCATTCCTTTGGATGGGATATGAACTCCATCCTGACACATGGACAGTC
CAGCCTATAGAACTGCCAGAAAAGGACAGCTGGACTGTCAATGATATACAGAAATTAGTG
GGAAAACTAAATTGGGCAAGTCAAATTTATGCAGGGGTTAAGGTAAAGCAACTGTGTAAA
CTCCTCAGGGGAACTAAAGCATTAACAGATGTAGTACCACTGACTAAAGAAGCAGAG

>302
CCTCAAATCACTCTTTGGCAACGACCCCTTGTCACAGTAAAAGTAGGAGGACAGATGArA
GAAGCTCTATTAGATACAGGAGCAGATGACACAGTATTAGAAGATATAAATTTGCCAGGA
AAATGGAAACCAAAAATGATAGGGGGAATTGGAGGTTTTATCAAGGTAAAGCAATATGAT
CAGATACTTATAGAAATTTGTGGAAAAAAGGCTATAGGTACAGTGTTAGTAGGACCTACA
CCTGTCAACATAATTGGACGAAATATGTTGACTCAGATTGGkTGTACTTTAAATTTCCCA
ATTAGTCCTATTGACACTGTACCAGTAACATTAAArCCAGGAATGGATGGACCAAAAGTT

AAACAGTGGCCATTAACAGAAGAAAAAATAAAAGCATTAACAGAAATTTGTAAAGAGATG
GAAGAGGAAGGAAAAATyTCAAAAATTGGGCCTGAAAATCCATACAATACTCCAGTATTT
GCTATAAAGAAAAAGGACAGCACCAAATGGAGGAAATTAGTAGATTTCAGAGAGCTCAAT
AAAAGAACTCAGGACTTTTGGGAAGTTCAATTAGGAATACCACATCCAGCAGGTTTAArG
AAAAGGAAATCAGTAACAGTACTAGATGTGGGAGATGCATATTTTTCAGTGCCTTTAGAT
GAAGGCTTTAGAAAGTATACTGCATTTACCATACCTAGTACAAACAATGAGACACCAGGA
ATTAGATATCAGTACAATGTGCTGCCACAGGGATGGAAAGGATCACCGGCAATATTCCAG
AGTAGCATGACAAAGATCTTAGAGCCCTTTAGAATAAAAAATCCAGAAATAGATATCTGT
CAATACGTGGATGACTTGTATGTAGGCTCTGATTTAGAAATAGGGCAGCACAGAATAAAA
ATAGAGGAGCTAAGAGCTCATCTATTGAGCTGGGGACTTACTACCCCAGACAAAAAGCAT
CAGAAGGAACCTCCATTCCTTTGGATGGGATATGAACTCCATCCTGACAAATGGACAGTC
CAGCCTATAGAACTGCCAGAAAAAGACAGCTGGACTGTCAATGATATACAGAAATTAGTG
GGAAAACTTAATTGGGCAAGTCAAATATATGCAGGAATTAAGGTAAAGCAACTGTGCAAA
CTCCTCAGAGGAGCTAAAGCACTAACAGACATAATACCACTGACTGAAGAAGCAGAG

>309
CCTCAAATCACTCTTTGGCAACGACCCCTCGTCACAATAAAGATAGGGGGACAGTTAAAG
GAAGCTCTATTAGATACAGGAGCAGATGATACAGTATTAGAAGACATGAATTTGCCAGGA
AGATGGAAACCAAAAATGATAGGGGGAATTGGAGGTTTTATCAAAGTAAGACAGTATGAT
CAGATACCCGTAGAAATyTGCGGACACAAGGCAGTAGGTACAGTATTAGTAGGACCTACA
CCTGTCAACATAATTGGGAGAAATCTGTTGACTCAGATTGGTTGCACTTTAAATTTTCCC
ATTAGTCCTATTGAAACTGTACCAGTAAAATTAAArCCAGGAATGGATGGCCCAAAArTT
AAACAATGGCCATTGACAGAGGAAAAAATAAAAGCACTAGTAGAAATTTGTGCAGAAATG
GAAAAAGAAGGAAAAATTTCAAAAATCGGGCCTGAAAAyCCATACAATACTCCAGTATTT
GCCATAAAGAAAAAAGACAGTACTAAATGGAGAAAATTAGTAGATTTCAGGGAACTTAAT
AAAAGAACTCAAGACTTCTGGGAAGTTCAATTAGGAATACCACATCCCGCAGGGTTAAAA
AAGAAAAAATCTGTAACAGTsCTGGATGTAGGTGATGCATATTTCTCAGTCCCTTTAGAy
AAAGACTTCAGGAAGTATACTGCATTTACCATACCTAGTGTAAACAATGAGACACCAGGG
ATCAGATATCAGTACAATGTGCTTCCACAGGGATGGAAAGGATCACCAGCAATATTCCAG
TGTAGCATGACAAAAATCTTAGrGCCTTTTAGAAAACAAAATCCAGACATAGTTATCTAT
CAATACATGGATGATTTGTATGTAGGATCTGACTTAGAAATAGGGCAGCATAGAGCAAAA
ATAGAGGAACTGAGACAACATTTATTGAGGTGGGGrTTTACCACACCAGACAAAAAGCAT
CAGAAAGAACCTCCATTCCTTTGGATGGGATATGAACTCCATCCTGATAAATGGACAGTA
CAGCCTATAGTGCTGCCAGAAAAGGACAGCTGGACTGTCAATGACATACAGAAGTTAGTG
GGAAAGTTrAATTGGGCAAGTCAGATTTATGCAGGGrTTAAGACAAAGGAATTATGTAAA
CTCATTAGGGGAACCAAGGCATTAACAGAAGTAGTACCACTAACAGAAGAAGCAGAG

>333
CCTCAAATCACTCTTTGGCAACGACCCCTCGTCCCAATAAGGATAGGGGGGCAATTAAAG
GAAGCTCTATTAGATACAGGAGCAGATGATACAGTATTAGAAGACATGAATTTGCCAGGG
AAATGGAAACCAAAAATGATAGGGGGAATTGGAGGTTTTATCAAAGTAAGACAGTATGAC
CAGATAGCCATAGAAATCTGTGGACACAAGGCTGTAGGTACAGTATTAATAGGACCTACA
CCTGTCAACATAATTGGGAGAAATCTGTTGACTCAGCTTGGTTGTACTTTAAATTTTCCC
ATTAGTCCTATTGAAACTGTACCAGTAAAATTAAAGCCAGGAATGGATGGCCCAAAAGTT
AAACAATGGCCATTGACAGAAGAAAAAATAAAAGCATTAGTAGAAATTTGTACAGAAATG
GAAAAGGAAGGAAAAATTTCAAAAATTGGGCCTGAAAATCCATACAATACTCCAGTATTT
GCCATAAAGAAAAAAGACAGTACTAAATGGAGAAAATTAGTAGATTTCAGGGAACTTAAT
AAAAGAACTCAAGACTTCTGGGAAGTTCAATTAGGAATACCACATCCCGCAGGGTTAAAA
AAGAAAAAATCAGTAACAGTCCTGGATGTGGGTGATGCATAyTTyTCAGTCCCTTTAGAT
AAAGACTTCAGGAAGTATACTGCATTTACyATACCTAGTGTAAACAATGAGACACCAGGG
ATCAGATATCAGTACAATGTGCTTCCACArGGATGGAAAGGATCACCAGCAATATTCCAG
TGTAGCATGACAAAAATCTTAGAGCCTTTTAGAAAACAAAATCCAGACATAGTTATCTAT
CAATACATGGATGATTTGTATGTAGGATCTGAyTTAGAAATAGGGCAGCATAGAGmAAAA
ATAGAGGAACTGAGACAACATCTGTTGAGGTGGGGATTTACCACACCAGAyAAAAAACAT
CAGAAAGAACCyCCATTCCTTTGGATGGGTTATGAACTCCATCCTGATAAATGGACAGTA
CAGCCTATAGTGCTGCCAGAAAAGGACAGCTGGACTGTCAATGACATACAGAAGTTAGTG
GGAAAGTTRAATTGGGCAAGTCAGATTTATGCAGGrATTAAGGTAArGGARTTATGTAAA
CTCCTTAGGGGAACCAAAGCACTAACAGAAGTAATACCACTAACAGAAGAAGCAGAG

>336
CCTCAAATCACTCTTTGGCAACGACCCCTTGTCACAATAAAAATAGGAGGACAGCTAAAA
GAAGCTCTATTAGATACAGGAGCAGATGATACAGTATTAGAAGATATAAATTTGCCAGGA
AAATGGAAACCAAAAATGATAGGGGGAATTGGAGGTTTTATCAAGGTAAGGCAATATGAT
CAGATACTTATAGAAATTTGTGGAAAAAGGGCTATAGGTACAGTGTTAGTAGGACCTACA
CCCGTCAACATAATTGGACGAAATATGTTGACTCAGCTTGGTTGTACTTTAAATTTCCCA
ATTAGTCCTATTGACACTGTACCAGTAACATTAAAGCCAGGAATGGATGGACCAAAGrTT
AAACAGTGGCCATTGACAGAAGAAAAAATAAAAGCACTAACAGAAATTTGTAGAGAAATG
GAAGAGGAAGGAAAAATCTCAAAAATTGGGCCTGAAAATCCATATAATACTCCAGTATTT
GCTATAAAGAAAAAGGACAGCACCAAATGGAGGAAATTAGTAGATTTCAGAGAGCTCAAT
AAAAGAACTCAGGACTTTTGGGAAGTTCAATTAGGAATACCGCATCCAGCAGGATTAAAA
AGGAAAAAATCAGTGACrGTACTAGATGTGGGAGATGCATATTTTTCAGTTCCTTTAGAT
GAAAGCTTTAGAAAGTATACTGCATTCACCATACCTAGTAyAAACAATGAGACACCAGGA
ATCAGATATCAGTACAATGTGCTACCACAGGGATGGAAAGGATCTCCGGCAATATTCCAG
AGTAGCATGACAAAAATCTTAGAGCCCTTTAGAAGAAAAAATCCAGAGATGGAGATCTGT
CAATACGTGGATGACTTGTATGTAGGATCTGATTTAGAAATAGGGCAGCATAGAACAAAA
ATAGAGGAGCTAAGAGCTCATCTATTGAGCTGGGGATTTACTACACCAGACAAAAAGCAT
CAGAAGGAACCTCCATTTCTTTGGATGGGATATGAACTCCATCCGGACAGATGGACAGTC
CAGCCTATAGAACTACCAGAAAAAGACAGCTGGACTGTCAATGATATACAGAAATTAGTG

GGAAAACTAAATTGGGCAAGTCAAATTTATGCAGGGATTAAGGTAAAGCAACTGTGTAGA
CTCCTCAGGGGAGCTAAAGCACTAACAGAGATAGTACCACTGACTGAAGAAGCAGAG

>355
CCTCAGATCACTCTTTGGCAGCGACCCCTTGTCTCAATAAAAGTAGGGGGCCAAATAAAA
GAGGCTCTCTTAGACACAGGAGCAGATGATACAGTATTGGAAGAAGTAAATTTGCCAGGT
AAATGGAAACCTAAAATGATAGGAGGAATTGGAGGTTTTATCAAAGTAAGACAATATGAG
CAAATACCTATAGAAATTTGTGGAAAAAAGGCTATAGGTACAGTATTAGTGGGACCCACA
CCTGTCAATATAATTGGAAGAAATATGTTGACTCAGCTTGGATGCACACTAAATTTTCCA
ATCAGTCCCATTGAAACTGTACCAGTAAAATTAAAGCCAGGmATGGATGGCCCAAAGGTT
AAACAATGGCCATTGACAGAAGArAAAATAAAAGCATTAACAGCAATTTGTGATGAAATG
GAAAAGGAAGGAAAAATTACAAAAATTGGGCCTGACAATCCATATAACACTCCAATATTT
GCCATAAAAAAGAAGGACAGTACTAAGTGGAGAAAATTAGTAGATTTyAGGGAACTCAAT
AAAAGAACTCAAGATTTTTGGGAAGTTCAATTAGGAATACCACACCCAGCAGGATTAAAA
AAGAAAAAATCAGTAACAGTCCTGGATGTGGGTGATGCATATTTCTCAGTTCCTTTAGAT
AAAGACTTCAGGAAGTATACTGCATTTACCATACCTAGTrTAAACAATGCGATACCAGGG
ATTAGATATCAGTACAATGTGCTCCCACAGGGATGGAAGGGATCACCAGCAATATTCCAA
TCTAGTATGACAAAAATCTTAGAGCCTTTTAGAAAACAAAATCCAGACATAGTTATCTAT
CAATACATGGATGACTTGTATGTAGGATCTGACTTAGAAATAGGGCAACATAGAACAAAA
ATAGAAGAACTGAGAGAACATCTGTTAAAGTGGGGATTTACCACACCAGACAAGAAACAT
CAGAAAGAACCTCCATTTCTTTGGATGGGGTATGAACTACATCCTGACAAATGGACAGTA
CAGCCTATACAACTGCCAGAAAAGGATAGCTGGACTGTCAATGATATACAAAAGTTAGTG
GGAAAATTAAACTGGGCAAGTCAGATTTACCCAGGAATTAAAGTAAGACAACTCTGTAAA
CTCCTTAGGGGGACCAAAGCACTAACAGAAGTAGTACCACTAACTGAAGAAGCAGAG

>365
CCTCAGATCACTCTTTGGCAACGACCCATCGTCACAATAAAGATAGGGGGGCAATTAAAG
GAAGCTCTATTAGATACAGGAGCAGATGATACAGTATTAGAAGACATGAATTTGCCAGGA
AGATGGAAACCAAAAATGATAGGGGGAATTGGAGGTTTTATCAAAGTAAGACAGTATGAT
CAGGTACCCATAGAAATTTGCGGACACAAGACTGAAGGTACAGTCTTAGTAGGACCTACA
CCTGTCAACATAATTGGGAGAAATCTGTTGACTCAGCTTGGTTGTACTTTAAATTTTCCC
ATTAGTCCTATTGAAACTGTACCAGTAAAATTGAAGCCAGGAATGGATGGCCCAAAAGTT
AAACAATGGCCATTGACAGAAGAAAAAATAAAAGCATTAGTAGAAATTTGTACAGAAATG
GAAAAGGAAGGAAAAATTTCAAAAATTGGGCCTGAAAATCCATACAATACTCCAGTATTT
GCCATAAAGAAAAAAGACAGTACTAAATGGAGAAAATTAGTAGATTTCAGGGAACTTAAT
AAAAGAACTCAAGACTTCTGGGAAGTTCAATTAGGAATACCACATCCTGCAGGGTTAAAA
AAGAAAAAATCTGTAACAGTCCTGGATGTGGGTGATGCATACTTCTCAGTTCCTTTACAT

GAAGATTTCAGGAAGTATACTGCATTTACCATACCTAGTATAAACAATGAGACTCCAGGG
ATAAGATATCAGTACAATGTACTTCCACAGGGATGGAAAGGATCACCAGCAATATTTCAA
TGTAGCATGACCAAAATCTTAGAGCCTTTTAGAAAAGAAAATCCAGACATAGAAATATAT
CAATACATGGATGATTTGTATGTAGGGTCTGACTTAGAAATAGGGCAGCATAGAACAAAA
ATAGAGGAACTGAGGGAACATCTGTTGAGGTGGGGATTTACCACACCAGACAAAAAACAT
CAGAAAGAACCCCCATTCCTTTGGATGGGTTATGAACTCCATCCTGATAAATGGACAGTA
CAGCCTATAGTGCTGCCAGAAAAAGACAGCTGGACTGTCAATGACATACAGAAGTTAGTG
GGAAAGTTGAATTGGGCAAGCCAGATTTATCCAGGGATTAAGGTAAAAGAATTATGTAAA
CTCCTTAGGGGAACCAAAGCACTAACAGAAGTAATACCATTAACAGAAGAAGCAGAG

>366
CCTCAAATCACTCTTTGGCAACGACCCCTTGTCACAGTAAAAATAGGAGGGCAGCTGAGA
GAAGCTCTATTAGATACAGGAGCAGATGATACAGTATTAGAAGATATAAATTTGCCAGGA
AAATGGAAGCCAAAAATGATAGGGGGAATTGGGGGTTTTATCAAGGTAAGACAATATGAT
CAGATACTTATAGAAATCTGTGGAAAAAAGGCTATAGGTACAGTATTAGTAGGACCTACA
CCTGTCAACATAATTGGACGAAATATGTTGACTCAGATTGGTTGTACTTTAAATTTCCCA
ATTAGCCCTATTAGCACTGTACCAGTAACATTAAAGCCAGGAATGGATGGGCCAAAGGTT
AAACAATGGCCATTGACAGAAGAAAAAATAAAAGCCTTAACAGAAATTTGTAAAGAGATG
GAAGAGGAAGGAAAAATCTCAAAAATTGGGCCTGAAAATCCACACAATACTCCAGTATTT
GCTATAAAGAAAAAAGACAGCACCAAATGGAGGAAATTAGTAGATTTCAGAGAACTCAAT
AAAAGGACTCAGGACTTTTGGGAAGTTCAATTAGGAATACCGCATCCAGCAGGTTTAAAA
AAGAACAAATCAGTAACAGTACTAGATGTGGGAGATGCATATTTTTCAGTTCCTTTAGAT
GAAAGCTTTAGAAAGTATACAGCATTCACCATACCTAGTATAAACAATGAGACACCAGGA
ATCAGATATCAGTACAATGTGCTGCCACAGGGATGGAAAGGATCACCAGCAATATTCCAG
CATAGCATGACAAAAATTTTAGAGCCCTTTAGAACAAAAAATCCAGAAGTGGTTATCTGT
CAATACGTGGATGACCTGTATGTAGGATCTGATTTAGAAATAGGACAGCACAGAAAAGTA
ATAGAAGAGCTAAGAGCTCATCTATTGAGCTGGGGATTTACTACACCAGACAAAAAGCAT
CAGAAGGAACCTCCATTTCTTTGGATGGGTTATGAACTCCATCCTGATAAATGGACAGTA
CAGCCTATAGTGCTGCCAGAAAAAGACAGCTGGACTGTCAATGACATACAGAAGTTAGTA
GGAAAGTTGAATTGGGCAAGTCAAATTTATGCAGGGATTAAGGTAAAGCAATTGTGTAAA
CTCCTCAAGGGAGCTAAGGCACTAACAGCTATAGTGCCACTGACTGAAGAAGCAGAG

>367
CCTCAAATCACTCTTTGGCAACGACCCCTCGTCACAGTAAAAATAGGrGGGCAGCTGAAA
GAAGCTCTATTAGATACAGGAGCAGATGATACAGTATTAGAGGATATAAATTTGCCAGGA
AAATGGAAACCAAAAATGATAGGGGGAATTGGGGGTTTTATCAAGGTAAGGCAATATGAT

CAGATACTGATAGAAATTTGTGGAAAAAAGGCTATAGGTACAGTATTAGTAGGACCTACA
CCTGTCAACATAATTGGACGAAATATGTTGACTCAGATTGGTTGTACTTTAAATTTCCCA
ATTAGTCCTATTAGCACTGTACCAGTAAmATTAAAGCCAGGAATGGATGGACCAAAGGTT
AAACAGTGGCCATTGACAGAAGAAAAAATAAAAGCCTTAACArAAATTTGTAAAGAGATG
GAAGAGGAGGGAAAAATCTCAAAAATTGGGCCTGAAAATCCATACAATACTCCAGTATTT
GCTATAAAGAAAAAGGACAGCACCAAATGGAGGAAATTAGTAGATTTCAGAGAGCTCAAT
AAAAGGACTCAGGACTTTTGGGAAGTTCAATTAGGAATACCTCATCCAGCAGGTTTAAAA
AAGAAAAAATCAGTAACAGTACTAGATGTGGGAGATGCATATTTTTCAGTTCCTTTAGAT
GAAAGCTTTAGAAAGTATACTGCATTCACCATACCTAGTATAAATAATGAGACACCAGGA
ATCAGATATCAGTACAATGTGCTGCCACAGGGATGGAAAGGATCACCGGCAATATTCCAG
TCTAGCATGACAAAAATCTTAGAGCCCTTTAAAATAAAAAATCCAGAAGTGGTTATCTAT
CAATACATGGATGACCTGTATGTAGGATCTGATTTAGAAATAGGACAGCACAGAAAAGTA
ATAGAAGAGCTAAGAGCTCATCTATTGAGCTGGGGATTTACTACACCAGACAAAAAGCAT
CAGAAGGAACCTCCATTTCTTTGGATGGGTTATGAACTCCATCCTGATAAATGGACAGTA
CAGCCAATAGAGCTGCCAGAAAAAGACAGCTGGACTGTCAATGACATACAGAAGTTAGTG
GGAAAGTTGAATTGGGCAAGTCAAATTTATGCAGGGATTAAGGTAAAGCAATTGTGTAAA
CTCCTCAGGGGAGCTAAGGCACTAACAGACATAGTwCCACTGACTGAAGAAGCAGAG

>378
CCTCAGATCACTCTTTGGCAACGACCCCTTGTCTCAATAAGAGTAGGGGGCCAGACAAAA
GAGGCTCTCCTAGATACAGGAGCAGATGATACAGTATTAGAAGAAGTAAATTTGCCAGGC
AAATGGAAACCAAAAATGATAGGGGGAATTGGAGGTTTTATCAAAGTAAGACAGTATGAT
CAGGTACCCATAGAAATTTGCGGrCACAAAGCTATAGGTACAGTATTArTAGGACCTACA
CCTGTCAACATAATTGGAAGAAATCTGTTGACTCAGCTkGGTTGCACTTTAAATTTTCCC
ATTAGTCCTATTGAAACGGTACCAGTAAAATTAAAGCCAGGAATGGATGGCCCAAAGGTT
AAACAGTGGCCATTGACAGAAGAAAAAATAAAAGCATTAACAGAAATTTGCAAAGAAATG
GAAGAGGAAGGGAAAATCTCAAAAATTGGGCCTGAAAATCCATACAATACTCCAGTATTT
GCTATAAAGAAAAAGGACAGCACCAAATGGAGGAAACTAGTAGATTTCAGAGAGCTCAAT
AAAAGAACTCAGGATTTTTGGGAAGTTCAATTAGGrATACCACATCCAGCAGGATTAAAA
AAGAAAAAATCAGTAACAGTACTAGATGTGGGAGATGCATATTTTTCAGTTCCTTTAGAT
GAAAGCTTTAGAAArTATACTGCATTCACCATACCTAGTATAAACAATGAGACACCAGGA
ATCAGATATCAGTACAATGTGCTGCCACAGGGATGGAAAGGATCACCAGCAATATTCCAG
AGTAGCATGACAAAAATCTTAGAGCCCTTTAGAGyAAAAAATCCAGAAATAATTATCTAT
CAATACATGGATGACTTGTATGTAGGATCTGATTTAGAAATAGGGCAGCATAGAACAAAA
ATAGAGGAGCTAAGAGCTCATCTATTGAGCTGGGGrTTTACTACACCAGACAAAAAGCAT
CAGAAGGAACCCCCATTCCTTTGGATGGGATATGAACTCCATCCTGATAGATGGACAGTy
CAGCCTATAAAACTGCCAGAAAAAGACAGCTGGACTGTCAATGATATACAGAAATTAGTG
GGAAAACTAAATTGGGCAAGTCArATTTATGCAGGrATTAAGGTAAArCAACTGTGTAAA
CTCCTTAGrGGGACTAAAGCACTAACAGACATAGTACCAyTGACTGAAGAAGCAGAG

>380
CCTCAGATCACTCTTTGGCAACGACCCCTTGTCTCAATAAAAGTAGGGGGCCARATAAAA
GArGCTCTCTTAGACACAGGAGCAGATGATACAGTATTAGAAGAAATAAATTTGCCAGGA
AAATGGAAACCAAAAATGATAGGAGGAATTGGAGGTTTTATCAAAGTAAGACAATATGAG
CAAATACCTATAGAAATTTGTGGGAAAAAAGCTATAGGTACAGTATTAGTGGGACCCACA
CCTGTCAACATAATTGGAAGAAATATGTTGACCCAGCTTGGATGCACATTAAATTTTCCA
ATCAGTCCCATTGAAACTGTACCAGTAAAATTAAAGCCAGGAATGGATGGCCCAAAGGTT
AAACAATGGCCATTGACAGAAGArAAAATAAAAGCATTAACAGAAATTTGTGATGAAATG
GAAAAGGAAGGAAAAATTACAAAAATTGGGCCTGAyAATCCATATAACACTCCAATATTT
GCCATAAAAAAGAAGGACAGTACTAAGTGGAGAAAATTAGTAGATTTCAGGGAACTCAAT
AAAAGAACTCAAGAyTTTTGGGArGTTCAATTAGGAATACCACACCCAGCAGGGTTAAAA
AAGAAAAAATCAGTmACAGTCCTGGATGTGGGTGATGCATATTTCTCAGTTCCTTTAGAT
GAAGAATTCAGGAAGTACACTGCATTCACCATACCTAGTATmAACAATGAGACACCAGGG
ATAAGATATCAGTACAATGTGCTTCCACAGGGATGGAAAGGATCACCAGCAATwTTCCAA
AGTAGCATGACAAAAATyTTAGAGCCTTTTAGAAAACAAAATCCAGACATAGTGATCTAT
CAATACATGGATGACTTGTATGTAGGATCTGACTTAGAAATAGGrCAGCATAGAACAAAA
ATAGAGGAACTGAGAGAACATCTGTTAAAGTGGGGATTTACCACmCCAGAyAAGAAACAT
CAGAAAGAACCTCCATTTCTTTGGATGGGGTATGAACTCCATCCTGACAAATGGACAGTA
CAGCCTATACAGCTGCCAGAAAAGGATAGCTGGACTGTCAATGATATACARAAGTTAGTG
GGAAAATTAAACTGGGCAAGTCAGATyTACCCAGGAATTAAGGTAAAGCAACTKTGTAAA
CTyCTTAAGGGGACCAAGGCACTAACAGACATAGTACCACTAACTGAGGAAGCAGAG

>382
CCTCAAATCACTCTTTGGCAACGACCCCTCGTCACAATAAAGATAGGGGGRCAATTAAAG
GAAGCTCTATTAGATACAGGAGCAGATGATACAGTATTAGAAGACATGAATTTGCCAGGA
AGATGGAAACCAAAAATGATAGGGGGAATTGGAGGTTTTATCAAAGTAAGACAGTATGAT
AAGATACCCATAGAAATCTGTGGACACAAGGCTATAGGTACAGTATTAATAGGACCTACA
CCTRTCAACATAATTGGAAGAAATCTGTTGACTCAGCTTGGTTGCACTTTAAATTTTCCC
ATTAGTCCTATTGAAACTGTACCAGTAAAATTAAAGCCAGGAATGGATGGCCCAAAAGTT
AAACAATGGCCATTGACAGAAGArAAAATAAAAGCATTAGTAGAAATTTGTACAGAAATG
GAAAAGGAAGGGAAAATTTCAAAAATyGGGCCTGAAAATCCATAyAATACTCCAGTATTT
GCCATAAAGAAAAAAGACAGTACTAAATGGAGAAAATTAGTAGATTTCAGGGAACTTAAT
AAAAGAACTCAGGACTTCTGGGAAGTTCAATTAGGAATACCACATCCCGCAGGGTTAAAr
AAGAAAAAATCTGTAACAGTCCTGGATGTGGGTGATGCATAyTTCTCAGTyCCTTTAGAT
AAAGACTTCAGGAAGTATACTGCATTTACCATACCTAGTGTAAACAATGAGACACCAGGG
ATCAGATATCAGTACAATGTGCTTCCACAGGGATGGAAAGGrTCACCAGCAATATTCCAA

TGTAGCATGACAAAAATyTTAGAGCCTTTTAGAAArCAGAATCCAGACATAGTTATCTAT
CAATACATGGATGATyTGTATGTAGGATCTGATTTAGAAATAGGrCAGCATAGAGCAAAA
ATAGAGGAAyTGAGACAACATyTGTTGAGGTGGGGATTTACCACACCAGACAAAAAACAT
CAGAAAGAACCYCCATTCCTTTGGATGGGTTATGAACTCCATCCTGATAAATGGACAGTA
CAGCCTATAGAGCTGCCAGAAAAGGACAGCTGGACTGTCAATGACATACAGAAGTTAGTr
GGAAAGTTGAATTGGGCAAGyCAGATTTATGCAGGGATyArGGTAAAGGAATTATGTAAA
CTCCTTAGGGGArCCAAAGCACTAACAGAGGTAATACCACTAACAGAAGAAGCAGAG

>385
CCTCAGATCACTCTTTGGCAGCGACCCCTTGTCTCAATAArAGTAGGGGGCCAGATAAAA
GAGGCTCTCTTAGACACAGGAGCAGATGATACAGTATTAGAAGrAkTAArTTTGCmAGGA
ArwTGGAAACCAAAAATGATAGGAGGAATTGGAGGTTTTATCAAAGTAAGACAATATGAT
CAAATACCTATAGAAATTTGTGGAAAAAAGGCTATAGGTACAGTATTAGTGGGACCCACA
CCTGTCAACATAATTGGAAGAAAymTGTTGACTCAGCTTGGATGCACAYTAAATTTTCCA
ATyAGTCCCATTGAAACTGTACCAGTAAAATTrAAGCCAGGAATGGATGGCCCAAAGGTT
AAACAATGGCCATTGACAGAAGArAAAATAAAAGCATTAACAGArATTTGTACAGAAATG
GAAAAGGAAGGrAAAATTTCAAAAATTGGGCCTGAAAATCCmTACAATACTCCAGTATTT
GCCATAAAGAAAAAAGACAGTACTAAATGGAGAAAATTAGTAGATTTCAGrGAACTTAAT
AAAAGrACTCArGAyTTTTGGGArGTTCArTTAGGAATACCACACCCAGCAGGGTTAAAr
AAGAArAAATCAGTGACAGTACTrGATGTGGGGGATGCATATTTTTCAGTTCCTTTrTAT
GAAGACTTCAGGAArTATACTGCATTCACCATACCTAGTATAAACAATGAGACACCAGGG
ATTAGGTATCAATATAATGTGCTTCCACAGGGATGGAAAGGATCACCAGCAATATTCCAG
GCTAGCATGACAArAATCTTAGAGCCCTTTAGGGCACAAAATCCAGAAATAGTCATCTAT
CAATATATGGATGACTTGTATGTAGGATCTGACTTAGAAATAGGGmAACATAGAGCAAAA
ATAGAGGAGCTAAGAGGACATCTGTTAAAGTGGGGATTTACCACmCCAGACAAGAAACAT
CAGAAAGAACCyCCATTTCTTTGGATGGGGTATGAACTCCATCCTGACAAATGGACAGTr
CAGCCTATACAGYTGCCAGAAAAAGATAGCTGGACTGTCAATGATATACAAAAGTTAGTG
GGAAAATTAAACTGGGCAAGTCAGATTTACCCAGGAATTAAAGTAAAGCAGCTTTGTAAA
CTCCTTAAGGGGGCCAAAGCACTAACAGACATAGTACCACTAACTGAAGAAGCAGAG

>388
CCTCAAATCACTCTTTGGCAACGACCCCTTGTCACAGTAAAAATAGGAGGACArCTGAAA
GAAGCTCTTTTAGATACAGGAGCAGATGATACAGTATTAGAAGAyATAAATTTGCCAGGA
AAATGGAAACCAAAAATGATAGGGGGAATTGGAGGTTTTATCAArGTAAGACAATATGAT
CAGATACTTATAGAAATTTGTGGAAAAAAGGCTATAGGTACAGTATTAGTAGGACCTACA
CCTGTCAACATAATTGGACGAAATATGTTGACTCAGATTGGTTGTACTTTAAATTTCCCA

ATTAGTCCTATTGAAACTGTACCAGTAACATTAAAGCCAGGrATGGATGGrCCAAAGGTT
AArCAGTGGCCATTGACAGAAGAAAAAATAAArGCATTAACAGAAATTTGTAAAGArATG
GAAGAGGAAGGAAArATCTCAAAAATTGGGCCTGAAAATCCATACAATACCCCAGTATTC
GCTATAAAGAAAAAAGACAGCACCAAATGGAGGAAGCTAGTAGATTTCAGAGAGCTCAAT
AAAAGAACTCAGGAyTTTTGGGAAGTTCAATTAGGAATACCACACCCAGCAGGTTTAAAA
AAGAAAAAATCArTAACAGTACTAGATGTGGGAGATGCATATTTTTCAGTTCCTTTAGAT
GAAAGCTTTAGAAAGTAYACTGCATTCACCATACCTAGTATAAACAATGAGACACCAGGA
ATCAGATATCAGTACAATGTGCTGCCACAGGGATGGAAAGGATCACCAGCAATATTCCAG
TGTAGCATGACAAAAATCTTAGAGCCCTTTAGAATAAAAAATCCAGAAATAACTATCTAC
CAATATATGGATGATTTATATGTAGCATCTGATTTAGAAATAGGACAGCATAGArCAAAA
ATAGAGGAGCTAAGAGCTCATCTATTGAGCTGGGGrTTTACTACACCAGACAAAAAGCAT
CAGAAGGAACCTCCATTCCTTTGGATGGGGTATGArCTCCATCCTGACAGATGGACAGTC
CAGCCTATAGAACTACCAGAAAAAGAyAGCTGGACTGTCAATGATATACAGAAATTAGTG
GGAAAACTAAATTGGGCAAGTCAAATTTATSCAGGGATTAAGrTAAAACAATTGTGTAAA
CTCCTCAGGGGAACTAAAGCACTAACAGACATAGTGCCACTGACTGAGGAAGCAGAG

>427
CCTCAAATCACTCTTTGGCAACGACCCCTCGTCmCAATAAGGATAGGGGGGCAATTAAAr
GAAGCTCTATTAGATACAGGAGCAGATGATACAGTGTTAGAAGAAATGAATTTRCCAGGA
AGATGGAAACCAAAAATGATAGGGGGAATTGGAGGTTTTATCAAAGTAAGACAGTATGAT
CAGGTACCCATAGAAATyTGTGGACACAAGGCTGTAGGTACAGTATTAATAGGACCCACA
CCTGTCAACATAATTGGRAGAAATCTGTTGACTCAGCTTGGTTGCACTTTAAATTTTCCT
ATyAGTCCTATTGAAACTGTACCAGTAAAATTAAAGCCAGGAATGGATGGCCCAAGAGTT
AAACAATGGCCATTAACAGAAGAAAAAATAAAAGCATTAGTAGAAATTTGTACAGAAATG
GAAAAGGAAGGrAAAATTTCAAAAATAGGGCCTGAAAACCCATACAATACTCCAGTATTT
GCCATAAAGAAAAAAGACAGTACTAAATGGAGAAAATTAGTAGATTTCAGGGAACTTAAT
AAAAGAACTCAAGACTTCTGGGAAGTyCAATTAGGAATACCACATCCAGCAGGGTTAAAA
AAGAGAAAATCAGTAACAGTCCTGGATGTGGGTGATGCATATTTCTCAGTCCCTTTAGAT
GAAGACTTCAGGAAGTATACTGCATTTACCATACCTAGTGTAAACAATGArACACCAGGG
ATCAGATATCAGTACAATGTGCTTCCACAGGGATGGAAAGGATCACCAGCAATATTCCAA
TGTAGCATGACAAAAATCTTAGAGCCTTTTAGAGAAmrCAATCCAGACATAGTTATCTAT
CAGTACATGGATGATTTGTATGTAGGmTCTGACTTAGAAATAGGGCAGCATAGAGCAAAA
ATAGAAGAACTrAGACAACATCTGTTGGGGTGGGGATTTACCACACCAGACAAAAAACAT
CAGAAAGAACCTCCATTCCTTTGGATGGGGTATGAACTCCATCCTGATAAATGGACAGTC
CAGCCCATAGTGCTGCCAGAAAAGGACAGCTGGACTGTCAATGACATACAGAAGTTAGTG
GGAAArTTrAATTGGGCAAGTCAAATTTATGCAGGGATTAAGGTAArGGAATTATGTAAA
CTCATTAGGGGAACCAAAGCACTAACAGAAGTAATACCACTAACAGAAGAAGCAGAG

>430
CCTCAAATCACTCTTTGGCArCGACCCCTTGTCTCAATAAAAGTAGGGGGCCAGATAAAA
GArGCTCTYTTAGAYACAGGAGCAGATGATACAGTATTAGAAGAAGTAAATTTGCCAGGr
AAATGGAAACCAAAAATGATAGGAGGAATTGGAGGATTTATCAAAGTAAGrCAATATGAT
CAAATACCTATAGAAATTTrTGGrAAAAAGGCTATAGGTACAGTATTAGTAGGACCTACA
CCKGTCAACATAATTGGAAGAAATCTGTTGACTCAGCTTGGTTGCACTTTAAATTTTCCC
ATTAGTCCTATTGAAACTGTACCAGTAAAATTAAAGCCAGGAATGGATGGCCCAAAAGTT
AAACAATGGCCATTAACAGAAGAAAAAATAAAAGCATTArTAGAAATTTGTACAGAAATG
GAAAAGGAAGGGAAAATTTCAAAAATTGGGCCTGAAAATCCATAYAATACTCCAGTATTT
GCCATAAAGAAAAAAGACAGTACTAAATGGAGAAAATTAGTAGATTTCAGGGAACTCAAT
AAAAGAACTCAGGATTTTTGGGAAGTTCArTTAGGAATACCACACCCAGCAGGGTTAAAA
AAGAAAAAATCAGTGTCAGTACTGGATGTGGGGGATGCATATTTTTCAGTTCCTTTAYAT
GArGACTTCAGGAAATATACTGCATTCACCATACCyAGTATAAACAATGAAACACCAGGG
ATTAGGTATCAATAyAATGTACTTCCACAGGGATGGAAAGGATCACCAGCAATATTCCAA
TGTAGCATGACAAAAATCTTAGAGCCCTTTAGAAAACAAAATCCAGAAATAGwCATCTAT
CAATATATGGATGACTTGTATGTAGGATCTGACTTAGAAATAGGGCAACATAGAGCAAAA
ATAGArGAGTTAAGAGAACATCTGTTAAAGTGGGGrTTTACCACACCAGAyAAGAAACAT
CAGAAAGAACCTCCATTTCTTTGGATGGGGTATGAACTCCATCCTGACAAATGGACAGTA
CAGCCTATACAGCTGCCAGAAAAGGATAGCTGGACTGTCAATGATATACAGAAGTTAGTG
GGAAAATTAAACTGGGCAAGTCAGATTTACCCAGGAATTAAAGTAArGCAACTTTGTARA
CTCCTTAGGGGGrCCAAAGCACTAACAGACATAGTACCACTAACTGAAGAAGCAGAG

>448
CCTCAAATCACTCTTTGGCAACGACCCCTCGTTACAATAAAGATAGGGGGGCAATTAAAG
GAAGCTCTATTAGATACAGGAGCAGATGATACAGTATTAGAAGAAATGAATTTGCCAGGA
AGATGGAAACCAAAAATGATAGGAGGAATTGGAGGTTTTATCAAAGTAAGACAGTATGAT
CAGGTATCCATAGAAATCTGyGGACACAAGGyTGTGGGTACAGTATTAATAGGACCTACA
CCyGTCAACATAATTGGGAGAAATCTGTTGACTCAGCTTGGTTGyACTTTAAATTTTCCT
ATTAGTCCTATTGAAACTGTACCAGTAAAATTAAAGCCAGGAATGGATGGGCCAAAAGTT
AAACAATGGCCATTrACAGAAGAAAAAATmAAAGCATTAGTAGAAATTTGTACAGAAATG
GAAAAGGAAGGGAAAATTTCAAAAATyGGGCCTGAAAATCCATACAATACTCCAGTATTT
GCCATAAAGAAAAAAGACAGTACTAAATGGAGAAAATTAGTAGATTTCAGGGAACTTAAT
AAAAGAACTCAAGACTTCTGGGAAGTbCAATTAGGAATACCACATCCCGCAGGGTTAAAA
AAGAAAAAATCyrTAACAGTCCTGGATGTGGGTGATGCATAYTTCTCAGTCCCTTTAGAT
AAAGACTTCAGGAAGTATACTGCATTTACCATACCTAGTGTAAACAATGAGACACCAGGG
ATCAGATATCAGTACAATGTGCTTCCACAGGGATGGAAAGGATCACCAGCAATATTCCAA
TGTAGCATGACAAAAATCTTAGAkCCTTTTAGArAACAAAATCCAGACATAGTTATCTAT
CAATACATGGATGATTTrTATGTAGGATCTGACTTAGAAATAGGGCAGCATAGArCAAAA

GTAGAGGAACTAAGACAACATTTGTTGGGGTGGGGATTTACCACACCAGACAAAAArCAy
CAGAAAGArCCTCCATTCCTTTGGATGGGTTATGAACTCCATCCTGATAAATGGACAGTA
CAGCCTATAGTGCTGCCAGAAAAGGACAGCTGGACTGTCAATGACATACAGAAGTTAGTG
GGAAAGTTrAATTGGGCAAGTCArATTTATGCAGGrATTAAGGTAAGGGAATTATGTAAA
CTCCTTAGGGGAGCCAAAGCACTAACAGAAGTAATACCACTAACAGAAGAAGCAGAG

>456
CCTCAGATCACTCTTTGGCAACGACCCCTCGTCACAATAAAGATAGGGGGGCAATTAAAG
GAAGCTCTATTAGATACAGGAGCAGATGATACAGTATTAGAAGACATGAATTTGCCAGGA
AGATGGAAACCAAAAATGATAGGGGGAATTGGAGGTTTTATCAAAGTAAGACAGTATGAT
CAGGTAGCCATAGAAATCTGTGGACACAAAGTTGTAGGTACAGTATTAATAGGAGATACA
CCTGTCAACATAATTGGGCGGAATCTGTTGACTCAGCTTGGTTGCACCTTAAATTTTCCC
ATTAGTCCTATTGAAACTGTACCAGTAAAATTAAAGCCAGGGATGGATGGCCCAAAAGTT
AAACAATGGCCATTGACAGAAGAAAAAATAAAAGCATTAGTAGAAATTTGTACAGAAATG
GAAAAAGAAGGGAAAATCTCAAAAATTGGGCCTGAAAATCCATACAATACTCCAGTATTT
GCCATAAAGAAAAAGGACAGTACTAAATGGAGAAAATTAGTAGATTTCAGGGAACTTAAT
AAAAGAACTCAAGACTTCTGGGAAGTTCAATTAGGAATACCACATCCCGCAGGGTTAAAA
AAGAAAAAATCAATTACAGTCCTGGATGTGGGTGATGCATATTTCTCAGTCCCTTTAGAT
AAAGACTTCAGGAAGTACACTGCATTTACCATACCTAGTATAAATAATGAGACACCAGGG
ATCAGATATCAGTACAATGTGCTTCCACAGGGATGGAAAGGATCACCAGCAATATTTCAA
TGTAGCATGACAAAAATCTTAGAGCCTTTTAGAAAACAAAATCCAGACCTAGTTATCTAT
CAATACATGGATGACTTGTATGTAGGATCTGATTTAGAAATAGGGCAACATAGAACAAAA
ATAGAGGAACTGAGACAACATTTGTTAGGGTGGGGATTTACCACACCAGACAAAAAACAT
CAGAAAGAACCTCCATTCCTTTGGATGGGGTATGAACTCCATCCTGATAAATGGACAGTA
CAGCCTATAGTGCTGCCAGAAAAGGACAGCTGGACTGTCAATGACATACAGAAGTTAGTG
GGAAAGCTGAATTGGGCAAGTCAGATTTATCCAGGGATCAAGGTAAGGGAATTATGTAAA
CTCCTTAGAGGAACCAAAGCACTAACAGAAGTAGTACCACTAACAGAAGAAGCAGAG

>534
CCTCAAATCACTCTTTGGCAACGACCCCTTGTTACAGTAAAAATAGGAGGACAGCTGAAA
GAAGCTCTATTAGATACAGGAGCAGATGATACAGTATTAGAAGATATAAATTTGCCAGGA
AAATGGAAACCAAAAATGATAGGGGGAATTGGAGGTTTTATCAAGGTAAGGCAATATGAT
CAAATACTTATAGAAATTTGTGGAAAAAAGGCTATAGGTACAGTATTAGTAGGACCTACA
CCTGTCAACATAATTGGAAGAAATATGTTGACTCAGATTGGTTGTACTTTAAATTTCCCC
ATTAGTCCTATTGACACTGTACCAGTGAAATTAAAGCCAGGAATGGATGGACCAAAAGTT
AAACAGTGGCCATTGACAGAAGAGAAAATAAAAGCATTAACAGAAATTTGTAAAGAGATG
GAAGCGGAAGGAAAAATCTCAAAAATTGGGCCTGAAAATCCATACAATACTCCAGTATTT

GCTATAAAGAAAAAGGACAGCACCAAATGGAGGAAATTGGTAGATTTCAGAGAGCTTAAT
AAAAGAACTCAAGACTTTTGGGAAGTTCAATTAGGAATACCGCATCCAGCAGGTTTAGAA
AAGAAAAAATCAGTAACAGTACTAGATGTGGGAGATGCATATTTTTCAGTTCCATTAGAT
AAAGACTTTAGAAAGTATACTGCATTCACCATACCTAGTACAAACAATGArACACCAGGA
ATCAGATATCAGTACAATGTGCTGCCACAGGGATGGAAAGGATCACCAGCAATATTCCAG
AGTAGCATGACAAAAATCTTAGAGCCCTTTAGAAAACAAAATCCAGAAATAATTATCTAT
CAATACGTGGATGACTTGTATGTAGCATCTGATTTAGAAATAGGGCAACACAGAACAAAA
GTAGmGGAGCTAAGAGATCATCTATTAAACTGGGGATTTACTACACCAGACAAAAAGCAT
CAGAAGGAACCGCCATTCCTTTGGATGGGATATGAACTCCATCCTGACAGATGGACAGTC
CAGCCTATAGAACTGCCAGAAAAGGACAGCTGGACTGTCAATGATATACAGAAATTAGTG
GGAAAACTAAATTGGGCAAGTCAAATTTATGCAGGGATTAAGATAAAGCAACTGTGTAAA
CTCCTCAGGGGAACTAAAGCACTAACAGACGTAGTACCCCTGACTGAAGAAGCAGAG

>536
CCTCAAATCACTCTTTGGCAACGACCCATTGTCACAGTAAAAATAGGGGGACAGCTAAAA
GAAGCTCTATTAGATACAGGAGCAGATGATACAGTATTAGAAGATATAAATTTGCCAGGA
AAATGGAAACCAAAAATGATAGGGGGAATTGGAGGTTTTATCAAGGTAAGACAATATGAT
CAGATAACTATAGAAATTTGTGGACAAAAGGCTATAGGTACAGTGTTAGTAGGACCTACG
CCTGTCAACATAATTGGGCGAAATATGTTGACTCAGATTGGCTGTACTTTAAATTTCCCA
ATTAGTCCTATTAACACTGTACCAGTAACATTAAAGCCAGGAATGGATGGACCAAAAGTT
AAACAATGGCCATTAACAGAAGAAAAAATAAAAGCATTAACAGAAATTTGTAAAGAGATG
GAGGCAGAAGGAAAAATCTCAAAAATTGGGCCTGAAAATCCATACAATACTCCAATATTT
GCTATAAAGAAAAAGGATGGCACCAAATGGAGAAAATTAGTAGACTTCAGAGAGCTCAAT
AAAAGAACTCAGGACTTTTGGGAAGTTCAATTAGGAATACCACATCCAGCAGGTTTAAAA
AAGAAAAAATCAGTAACAGTACTAGATGTGGGAGATGCATATTTTTCAGTTCCTTTAGAT
GAAAGCTTTAGAAAGTATACTGCATTCACCATACCTAGTATAAACAATGAGACACCAGGA
ATCAGATATCAGTACAATGTGCTGCCACAAGGATGGAAAGGATCACCGGCAATATTCCAA
AGTAGCATGACAAAAATCTTAGAGCCCTTTAGAATAAAAAATCCAGACATAGTAATCTAT
CAATATATGGATGACTTGTATGTAGGATCTGATTTAGAAATAGAGCAGCACAGAGCAAAA
ATAGAGGAGCTGAGAGCTCATCTATTGAGCTGGGGATTTACTACACCAGACAAAAAGCAT
CAGAAGGAACCyCCATTyCTTTGGATGGGATATGAACTCCATCCTGACAGATGGACAGTC
CAGCCTATAGAACTGCCAGAAAAAGACAGCTGGACTGTTAATGATATACAGAAATTAGTG
GGAAAGCTAAATTGGGCAAGTCAGATTTATGCAGGGATTAAGATAAAGCAACTGTGTAAA
CTCCTCAGGGGAGCTAAAGCACTAACAGACATAGTACAACTGACTGAAGAAGCAGAG

>537

CCTCAGATCACTCTTTGGCAACGACCCCTTGTCTCAATAAAAGTAGGGGGyCAGATAAAA
GAGGCTCTCTTAGACACAGGAGCAGATGATACAGTATTAGAAGAAGTAAATTTGCCAGGA
AAATGGAAACCrAAAATGATAGGrGGAATTGGAGGTTTTATCAAAGTAAGACAATATGAG
CAAATACCTATAGAAATTTGTGGAAAAAAGGCTATAGGTACAGTATTAGTGGGACCCACA
CCTGTCAACATAATTGGAAGAAATATGTTGACCCAGCTTGGATGCACACTAAATTTTCCA
ATCAGTCCCATTGAAACTGTACCAGTGAAATTAAAGCCAGGAATGGATGGCCCAAGGGTT
AAACAATGGCCATTGACAGAAGAGAAAATAAAAGCATTAACAGCAATTTGTGAGGAAATG
GAGAAGGAAGGAAAAATTACAAAAATTGGGCCTGACAATCCATATAATACTCCAATATTT
GCCATAAAAAAGAAGGACAGTACTAAGTGGAGAAAATTAGTAGATTTCAGGGAACTCAAT
AAAAGAACTCAAGATTTTTGGGAAGTTCAGTTAGGAATACCACACCCwGCAGGGTTAAGA
AAGAAAAAATCAGTAACAGTCCTGGATGTGGGTGATGCATATTTCTCAGTTCCTTTAGAT
AAAGATTTCAGGAAGTATACTGCATTTACCATACCTAGTGTAAACAATGAGACACCAGGG
ATTAGATATCAGTACAATGTGCTCCCACAGGGATGGAAAGGATCACCAGCAATATTCCAA
AGTAGCATGACAAAAATCTTAGAACCTTTTAGAAAACAAAATCCAGACATAGTTATCTAT
CAATACATGGATGACTTGTATGTGGGATCTGACTTAGAAATAGGGmAGCATAGAGCAAAA
ATAGAGGAACTGAGAGAACATCTGTTAAAGTGGGGrTTTACCACACCAGACAAGAAACAT
CAGAAAGAACCCCCATTTCTCTGGATGGGGTATGAACTCCATCCTGACAAATGGACAGTA
CAGCCTATAGAGCTGCCAGAAAAGGATAGCTGGACTGTCAATGATATACAGAAGTTAGTG
GGAAAATTAAACTGGGCAAGYCAGATTTACCCAGGArTTAAAGTAAGGCAACTTTGTAAA
CTCCTTAGGGGGGCCAAAGCACTAACAGACATAGTACCACTAACTGAAGAAGCAGAG

>541
CCTCAAATCACTCTTTGGCAACGACCCCTTGTCACAATAAAAATAGGGGGACAACTAAAA
GAAGCTCTATTAGATACAGGAGCAGATGATACAGTATTAGAAGATATAAATTTGCCAGGA
AAATGGAAACCAAAAATGATAGGGGGAATTGGAGGTTTTATCAAGGTAAGGCAATATGAT
CAGATACTTATAGAAATTTGTGGAAAAAAGGCTATAGGTACAGTATTAGTAGGACCTACA
CCGGTCAACATAATTGGACGAAATATGTTGACTCAAATTGGTTGTACTTTAAATTTCCCC
ATTAGTCCTATTGACACTGTACCAGTAAAATTAAAGCCAGGAATGGATGGACCAAAGGTT
AAACAGTGGCCATTGACAGArGAAAAAATAAAAGCATTAACAGAAATTTGTAAAGAGATG
GAAGAGGAAGGAAAAATCTCAAAAATTGGGCCTGAAAATCCCTATAATACTCCAGTATTT
GCTATAAAGAAAAAGGACAGCAmCAAATGGAGGAAATTAGTAGATTTCAGAGAGCTCAAT
AAAAGAACTCArGACTTTTGGGAAGTTCAATTAGGAATACCkCATCCAGCAGGATTAAAA
AAGAAAAAATCAGTGACAGTACTrGATGTGGGAGATGCATATTTTTCAGTTCCTTTAGAT
GAAAGCTTTAGAAAATATACTGCATTCACCATACCTAGTATAAACAATGAGACACCAGGA
ATCAGATATCAGTACAATGTGCTrCCACAGGGATGGAAAGGATCTCCGGCAATATTCCAG
TGTAGCATGACAAAAATCTTAGAkCCCTTTAGAAArAAmAATCCAGAGATGGATATCTAT
CAATACATGGATGACTTGTATGTAGGATCTGATTTAGAAATAGGGCAGCACAGArCAAAA
ATAGAGGAGCTAAGAGCTCATCTATTGAGCTGGGGATTTACTACACCAGACAAAAAGCAT
CAGAAGGAACCTCCATTTCTTTGGATGGGATAyGAACTCCATCCGGACAAATGGACAGTC
CAGCCTATArAACTACCAGAAAAAGACAGCTGGACTGTCAATGATATACAGAAATTAGTG

GGAAAGCTAAATTGGGCAAGTCAAATTTATGCAGGGATTAAGGTAAAGCAACTGTGTAAA
CTCCTCAGGGGAGCTAAAGCACTAACAGAAGTAGTACCACTGACTGAAGAAGCAGAG

>554
CCTCAAATCACTCTTTGGCAACGACCCGTTGTCACAGTAAAAATAGGAGGGCAGCTGAAA
GAAGCCCTATTAGATACAGGAGCAGATGATACAGTATTAGAAGATATAAATCTGCCAGGA
AAATGGAAACCAAAAATGATAGGGGGAATTGGAGGTTTTATCAAGGTAAAGCAATATGAT
CAGATACTTATAGAAATTTGTGGAAAAAGGGCTATAGGTACAGTrTTAGTAGGACCTACA
CCTGTCAACATAATTGGACGAAATATGTTGACTCAGATTGGTTGTACTTTAAATTTCCCA
ATTAGTCCTATTGACACTGTACCAGTAAAATTAAAGCCAGGAATGGATGGACCAAArGTT
AAACAGTGGCCATTGACAGAAGAAAAAATAAAAGCATTAACAGATATTTGTAAAGAAATG
GAACAGGAAGGAAAAATCTCAAAAATTGGGCCTGAAAATCCATACAATACTCCAGTATTT
GCTATAAAGAAAAAGGACAGCACCAAATGGAGAAAATTGGTAGATTTCAGAGArCTTAAT
AAAAGAACTCAGGACTTTTGGGAAGTTCAATTAGGAATACCGCATCCAGCAGGTTTAAAA
AAGArAAAATCCATAACAGTACTAGATGTGGGAGATGCATATTTTTCAGTTCCACTAGAT
AAAGACTTTAGAAAGTATACTGCATTCACCATACCTAGTATAAACAATGAGACACCAGGA
ATCAGATATCAGTACAATGTGCTTCCACAGGGATGGAAGGGATCACCAGCAATATTCCAA
AGTAGCATGACAAAAATCTTAGAGCCTTTTAGAAAACAAAATCCAGAAATAGATATCTAT
CAATACrTGGATGATTTGTATGTAGCATCTGACTTAGAAATAGGGCAGCATAGAGCAAAA
ATAGAGGAACTGAGGCAACATCTGTTArGGTGGGGACTTACCACACCAGACAAAAAACAT
CAGAAGGAACCCCCATTCCTTTGGATGGGATATGAGCTCCATCCTGACAAATGGACAGTC
CAGCCTATAGAACTGCCAGAAAAGGACAGTTGGACTGTCAATGATATACAGAAATTAGTA
GGAAAACTAAATTGGGCAAGCCAGATTTATGCAGGGATTAAGGTAAAACAACTGTGTAAG
CTCCTCAGGGGAGCAAAAGCACTAACAGACATAGTACCACTGACTGCAGAAGCAGAG

>620
CCTCAGATCACTCTTTGGCAACGACCCCTTGTCACAATAAGAATAGGAGGACAGCTGAAA
GAAGCTCTATTAGATACAGGAGCAGATGATACAGTATTAGAAGATATAAATTTGCCAGGA
AAATGGAAACCAAAAATGATAGGGGGAATTGGAGGTTTTATCAAAGTAAGGCAATATGAT
CAGATACTTATAGAAATTTGTGGAAAAAAGGCTATAGGTACAGTGTTAGTGGGACCTACA
CCTGTCAACATAATTGGACGGAATATGCTGACTCAGATTGGTTGTACTTTAAATTTTCCA
ATTAGTCCTATTGACACTGTACCAGTAACATTAAAGCCAGGAATGGATGGACCAAAGGTT
AAACAGTGGCCATTGACAGAAGAAAAAATAAAAGCATTAACAGAAATTTGTAGGGAAATG
GAAGAGGAAGGAAAAATCTCAAAAATTGGGCCTGAAAATCCATATAATACTCCAGTATTT
GCTATAAAGAAAAAGGATGGCACCAAATGGAGGAAATTAGTAGATTTCAGAGAGCTCAAT
AAAAGAACTCAGGACTTTTGGGAAGTACAATTAGGAATACCGCATCCAGCAGGATTAAAA

AAGAACAAATCAGTGACAGTATTAGATGTGGGAGATGCATATTTTTCAGTCCCTTTAGAT
GAAAGCTTTAGAAAGTATACTGCATTCACCATACCTAGTACAAACAATGAGACACCAGGA
ATCAGATATCAGTACAATGTGCTGCCACAGGGATGGAAAGGATCTCCGGCAATATTCCAG
TGTAGCATGACAAAAATCTTAGAGCCCTTTAGAAGAAAAAATCCAGAGATAGTTATCTAT
CAATACGTGGATGACTTGTATGTAGGATCTGATTTAGAAATAGGGCAGCACAGAACAAAA
ATAGATGAGCTAAGAGCTCATCTATTGAGATGGGGATTTACTACTCCAGACAAAAAGCAT
CAAAAAGAACCGCCATTTCTTTGGATGGGATATGAACTCCATCCGGACAGATGGACAGTC
CAGCCTATAGAACTGCCAGAAAAAGACAGCTGGACTGTCAATGATCTACAGAAATTAGTG
GGAAAACTAAATTGGGCAAGTCAAATTTATGCAGGGATTAAGGTAAAGCAACTGTGTAAA
CTCCTCAGGGGAACTAAAGCATTAACArAAGTCGTACCACTGACTGAAGAAGCAGAG

>624
CCTCAAATCACTCTTTGGCAGCGACCCCTCGTCTCAATAAAGATAGGGGGGCAACAAAAG
GAAGCTCTATTAGATACAGGAGCAGATGATACAGTATTAGAAGAAATGCATTTACCAGGA
ArATGGAAACCAAAAATGATAGGGGGAATTGGAGGyTTTATCAAAGTAAGACAGTATGAT
CArrTACCCATAGAAATTTACGGACATAAAGCTATAGGTACAGTATTAATAGGACCTACA
CCTGTCAACATAATTGGAAGAAATCTGTTGACTCAGATTGGCTGCACTTTAAATTTTCCC
ATTAGTCCTATTrAAACTGTACCAGTAAAATTAAAGCCAGGTATGGATGGCCCAAAAGTT
AAACAATGGCCATTGACAGAAGAAAAGATAAAAGCATTAGTAGAAATTTGTACAGAAATG
GAAAAGGAAGGAAAAATTTCAAAAATAGGGCCTGAAAATCCATACAATACTCCAGTATTT
GCAATAAAGAAAAAAGACAGTAATAAATGGAGAAAATTAGTAGATTTCAGAGAACTCAAT
AAAAGAACTCAAGACTTCTGGGAGGTTCAATTAGGAATACCACATCCCGGCGGGTTAAAA
AAGAAAAAATCAATAACAGTACTGGATGTGGGTGATGCATATTTTTCAATTCCCTTAyAT
GAGGACTTTAGGAArTATACTGCATTTACCATACCTAGTACAAAYAATGAGACACCAGGG
ATTAGGTATCAGTACAATGTGCTTCCACAGGGATGGAAAGGATCACCAGCAATATTCCAA
AGTAGCATGACAAAAATCTTAGATCCTTTTAGAAAACAAAATCCAGACATAGTGATTTGT
CAGTACATGGATGATTTrTATGTAGGATCTGACTTAGAAATAGGGCAACATAGAACAAAA
GTAGAGGAACTGAGACAACATCTGTTGAGGTGGGGATTAACCACACCAGACAAAAAACAT
CAGAAAGAACCTCCATTCCTTTGGATGGGTTATGAACTCCATCCTGATAAATGGACAGTA
CAACCTATACTGCTGCCAGAAAAGGACAGCTGGACTGTCAATGACATACAGAAGTTAATA
GGAAAACTGAATTGGGCAAGTCAAATTTATGCAGGGATTAAAGTAAAGCAATTATGTAAA
CTCCTTAGGGGAGCCAAATCACTAACAGAAGTAGTACCACTAACACATGAAGCAGAG

>657
CCTCAAATCACTCTTTGGCAACGACCCCTCGTCAAAATAAAGATAGGGGGGCAATTAAAG
GAAGCTCTrTTAGATACAGGAGCAGATGATACAGTATTAGAAGACATGGATTTGCCTGGG
AAATGGAAACCAAAAATGATAGGGGGAATTGGAGGTTTTATCAAAGTAAAACAGTATGAA

CAGATACCCATAGArATCTGTGGACACAAAGTTATAGGTACAGTATTAGTAGGACCTACA
CCTGTCAACATAATTGGAAGAAATCTrTTGACCCAGCTTGGTTGCACTTTAAATTTTCCA
ATTAGTCCCATTGAAACTGTACCAGTAAAATTAAAGCCAGGAATGGATGGTCCAAAAGTT
AAACAATGGCCATTGACAGAAGAGAAAATAAAAGCATTAACAGCAATTTGTGATGAAATG
GAGAAGGAAGGAAAAATTACAAAAATTGGGCCTGAAAATCCATATAACACTCCAATATTT
GCTATAAAAAAGAAGGACAGTACTAAGTGGAGAAAATTAGTAGATTTCAGGGAACTCAAT
AAAAGAACTCAAGATTTTTGGGAAGTTCAATTAGGAATACCACACCCAGCAGGGTTAAAA
AAGAAAAAATCAATGACAGTACTGGATGTGGGGGATGCATACTTCTCAGTTCCTTTATAT
GAAGACTTCAGGAAATATACTGCATTCACCATACCTAGTATAAACAATGAAACACCAGGG
ATTAGGTATCAGTACAATGTACTTCCACAGGGATGGAAAGGATCACCAGCAATATTCCAA
AGTAGCATGACAAAAATCTTAGAGCCTTTTAGAAAACAAAATCCAGACATAGTTATCTAC
CAATACATGGATGATTTATATGTAGGATCTGACTTAGAGATAGGGCAGCATAGAACAAAA
GTAGAGGAACTGAGACAACATTTGTTGGGGTGGGGATTTACCACACCAGACAAGAAACAT
CAGAAGGAACCTCCATTTCTTTGGATGGGGTATGAACTCCATCCTGACAAATGGACAGTA
CAGCCTATACAGCTrCCAGAAAARGATAGCTGGACTGTCAACGATATACAAAAGTTAGTG
GGAAAATTAAACTGGGCAAGTCAGATTTATCCTGGAATTAAArTAAGGCAACTTTGCAAA
CTyCTTAGGGGGGCCAAAGCACTAACAGACATAGTACCACTAACTGAAGAAGCAGAG

>665
CCTCAAATCACTCTTTGGCAACGACCCCTCGTCACAATAAGGATAGGGGGGCAATTAAAG
GAGGCTCTATTAGATACAGGAGCAGATGATACAGTATTAGArGACATGGATTTACCAGGA
AGATGGAAACCAAAAATGATAGGGGGAATTGGAGGTTTTATCAAAGTAAGACAGTATGAT
CAGATACCCATAGAAATCTGTGGACACAAGGTTGTAGGTACAGTGTTAAyAGGACCCACA
CCTrTCAACATAATTGGGAGAAATTTGTTGACTCAGCTTGGTTGCACTTTAAATTTTCCC
ATTAGTCCTATTGAAACTGTACCAGTAAAATTAAAGCCAGGAATGGATGGCCCAAAAGTT
AAACAATGGCCATTGACAGAAGAAAAAwTAAAAGCATTAGTAGAAATTTGTACAGAAATG
GAAAAGGAAGGAAAAATTTCAAAAATTGGGCCTGAAAATCCATACAATACTCCAGTATTT
GCCATAArGAAAAAAGACAGTGCTAAATGGAGAAAATTAATAGATTTCAGGGAACTTAAT
AAAAGAACTCAAGACTTCTGGGAAGTTCAATTAGGAATACCACATCCCGCAGGGTTAAAG
AAGAATAAATCAGTAACAGTCCTGGATGTGGGTGATGCATATTTCTCAGTCCCTTTAGAT
AAAGACTTCAGGAAGTATACTGCATTTACCCTACCTAGTTTAAACAATGAAACACCAGGG
ATCAGATATCAGTACAATGTGCTTCCACAGGGATGGAAAGGATCACCAGCAATATTCCAA
TGTAGCATGACAAAAATCTTAGAACCTTTTAGAAAACAAAATCCAGACATAGTTATCTAT
CAATACGTAGATGATTTGTATGTAGGATCTGACTTAGAAATAGGGCAGCATAGAGCAAAA
ATAGAAGAACTGAGACAACATTTGTTGAAGTGGGGAyTCACCACACCAGACAAAAAGCAT
CAGAAAGAACATCCATTCCTTTGGATGGGTTATGAACTCCATCCTGATAAATGGACAGTA
CAGCCTATAGTGCTGCCAGAAAAGGACAGCTGGACTGTCAATGACATACAGAAGTTAGTG
GGAAAGTTAAATTGGGCAAGTCAGATTTATGCAGGGATTAAGACAAGGGAATTATGTAAA
CTCATTAGGGGAACCAGAGCACTAACAGAAGTAGTACCACTAACAGAAGAAGCAGAG

>682
CCTCAAATCACTCTTTGGCAACGACCCCTCGTCACAATAAAGATAGGGGGGCAATTAAAG
GAAGCYCTATTAGATACAGGAGCAGATGATACAGTATTAGAAGACATAAATTTGCCAGGA
AGATGGAAACCAAAAATGATAGGGGGAATTGGAGGTTTTATCAAAGTAAGACAGTATGAT
CAGATACCCATAGAAATCTGTGGACACMAGGyTGWGGGTACAGTATTAATAGGACCTACA
CCTGTCAACATAATTGGAAGAAATCTGTTGACTCAGCTTGGTTGCACTTTAAATTTTCCy
ATTAGTCCTATTGAAACTGTACCAGTAAAATTAAAGCCAGGAATGGATGGCCCAAAAGTT
AAACAGTGGCCATTGACAGAAGAAAAAATAAAAGCATTAGTAGAAATTTGTACAGAAATG
GAAAAGGAAGGGAAAATTTCAAAAATTGGGCCTGAGAATCCATACAATACTCCAGTATTT
GCAATAAAGAAGAAAGACAGTACTAAATGGAGAAAATTAGTAGATTTCAGrGAACTTAAT
AAAAGAACTCAAGACTTCTGGGAGGTTCAATTAGGAATACCACATCCCGCAGGGTTAAAG
AAGAAAAAATCTGTAACAGTCTTGGATGTGGGTGATGCATATTTCTCAGTTCCTTTAGAT
AArGACTTCAGGAAGTATACTGCATTTACCATACCTAGTGTAAACAATGAGACACCAGGr
ATTAGATATCAGTACAATGTGCTTCCACAGGGATGGAAAGGATCACCAGCAATATTCCAA
TGTAGTATGACAAAAATCTTAGAGCCTTTTAGAAAACAAAATCCAGACATAGTTATCTAT
CAATAyATGGATGACTTrTATGTAGGATCTGACTTAGAAATAGGGCAGCATAGAGCAAAA
ATAGAGGAACTGAGACAACATCTGTTGAGGTGGGGGTTTACCACACCAGACAAAAAACAT
CAGAAAGAACCTCCATTCCTTTGGATGGGGTATGAACTCCATCCTGATAAATGGACAGTA
CAGCCTATAGArCTGCCAGAAAAGGACAGCTGGACTGTCAATGACATACAGAAGTTAGTG
GGAAAGTTGAATTGGGCAAGCCAGATTTATGCAGGGATTAAGGTAArAGAATTATGTAAA
CTCCTTAGGGGAACCAAAGCACTAACAGAAGTAATACCACTAACAGAAGAAGCAGAG

>689
CCTCAAATCACTCTTTGGCAGCGACCCCTTGTCACAATAAGAATAGGAGGACAGCTGAAA
GAAGCTCTATTAGATACAGGAGCAGATGATACAGTATTAGAAGATATAAATTTGCCAGGA
AAATGGAAACCAAAAATGATAGGGGGAATTGGAGGTTTTATCAAAGTAAGGCAATATGAT
CAGATACTTATAGAAATTTGTGGGAAAAAGGCTATAGGTACAGTGTTAGTAGGACCTACA
CCTGTCAACATAATTGGACGAAATATGTTGACTCAGATTGGTTGTACTTTAAATTTTCCA
ATTAGTCCTATTGACACTGTACCAGTAACATTAAAGCCAGGAATGGATGGACCAAAGGTT
AAACAGTGGCCATTGACAGAAGAAAAAATAAAAGCATTAACAGAAATTTGTAGGGAAATG
GAAGAGGAAGGAAAAATCTCAAAAATTGGGCCTGAAAATCCATATAATACTCCAGTATTT
GCTATAAAGAAAAAGGATAGCACCAAATGGAGAAAATTAGTAGATTTCAGAGAGCTCAAT
AAAAGAACTCAGGACTTTTGGGAAGTACAATTAGGAATACCGCATCCAGCGGGATTAAAA
AAGAAAAAATCAGTGACAGTACTAGATGTGGGAGATGCATATTTTTCAGTCCCTTTAGAT
GAAAGCTTTAGAAAGTATACTGCATTCACCATACCTAGTACAAACAATGAGACACCAGGA
ATCAGATATCAGTACAATGTGCTGCCACAGGGATGGAAAGGATCTCCGGCAATATTCCAG

TGTAGCATGACAAAAATCTTAGAGCCCTTTAGAAGAAAAAATCCAGAGATGGTTATCTAT
CAATACATGGATGACTTGTATGTAGGATCTGATTTAGAAATAGGGCAGCACAGAACAAAA
ATAGATGAGCTAAGAGCTCATCTATTrAGCTGGGGATTTACTACTCCAGACAArAAGCAT
CAAAAAGAACCrCCATTTCTTTGGATGGGATATGAACTCCATCCGGACAGATGGACAGTC
CAGCCTATAGAACTGCCAGAAAAAGACAGCTGGACTGTCAATGATATACAGAAATTAGTG
GGAAAACTAAATTGGGCAAGTCAAATTTATGCAGGGATTAAGGTAAAGCAACTGTGTAAA
CTCCTCAGGGGAGCTAAAGCATTAACAGAAGTAGTACCACTGACTGAAGAAGCAGAG

>696
CCTCAGATCACTCTTTGGCAGCGACCCCTCGTCTCAATAAAGATAGGGGGGCAACAAAAG
GAAGCTCTATTAGATACAGGAGCAGATGATACAGTATTAGAAGAmATGYrTTTACCAGGA
AGATGGAAACCAAAAATGATAGGGGGAATTGGAGGTTTTATCAAAGTAAGACAGTATGAT
CAkATACyCATAGAAATTTGyGGAyATAAAGCTATAGGTACAGTATTAATAGGACCYACA
CCTGTCAACATAATTGGAAGAAATCTATTGACTCAGATTGGCTGyACTTTAAATTTTCCC
ATTAGyCCTATTGAAACTGTACCAGTAAAATTAAArCCAGGTATGGATGGACCAAAAGTT
AAACArTGGCCATTGACAGAAGAAAAAATAAAAGCATTAGTAGAAATTTGTACAGArATG
GAAAAGGAAGGAAAAATTTCAAAAATAGGGCCTGAAAATCCATACAATACTCCAGTATTT
GCAATAAAGAAAAAAGACAGTACTAAATGGAGAAAATTAGTAGATTTyAGAGAACTCAAT
AAAAGAACTCAAGACTTCTGGGAGGTTCAATTAGGAATACCACAyCCyGCAGGGTTAAAA
AAGAAAAAATCAATAACAGTACTGGATGTGGGTGATGCATATTTTTCArTTCCCTTATrT
GAGGACTTTAGGAAGTATACTGCATTTACCATACCTAGTACAAACAATGAGACACCAGGG
ATTAGRTATCAGTACAATGTGCTTCCACAGGGATGGAAGGGATCACCAGCAATATTCCAA
AGTAGCATGACAArAATCTTAGATCCyTTTAGAAAACAAAATCCAGACATAGTGATCTAT
CAGTACATGGATGATTTGTATGTAGGATCTGACTTAGAAATAGGGCAACATAGAACAAAA
GTAGAGsArCTsAGACAACATCTGTTGAAGTGGGGATTAACCACACCAGACAAAAAACAT
CAGAAAGAACCTCCATTCCTYTGGATGGGTTATGAACTCCATCCTGATAAATGGACAGTA
CAACCTATArTACTGCCAGAAAAGGACAGCTGGACTGTCAATGACATACAGAAGTTAATA
GGAAArCTrAATTGGGCAAGTCAAATTTATGCAGGGATTAAAGTAArGCAATTATGTAAA
CTCCTTAGGGGAGCCAAATCACTAACAGAAGTAGTACCACTAACACATGAAGCAGAG

>717
CCTCAAATCACTCTTTGGCAACGACCCCTTGTCACAATAAAAATAGGAGGACAGCTGAAA
GAAGCTCTATTAGATACAGGAGCAGATGATACAGTATTAGAAGATATAAATTTGCCAGGA
AArTGGAAACCAAAAATGATAGGGGGAATTGGGGGTTTTATCAAAGTAAGGCAATATGAT
CAGATACCTATAGAAATTTGTGGAAAACAGGCTATAGGTACAGTGTTAGTAGGACCTACA
CCTGTCAACATAATTGGACGAAATATGTTGACTCAGCTTGGTTGTACTTTAAATTTTCCA
ATTAGTCCTATTGAmACTGTACCAGTAACATTAAAGCCAGGAATGGATGGACCAAAGGTT

AAACAGTGGCCATTGACAGAAGAAAAAATAAAAGCATTAAyAGAmATTTGTAAGGArATG
GAAGmrGAAGGAAAAATCTCAAAAATTGGGCCTGAAAATCCATATAATACTCCAGTATTT
GCTATAAAGAAAAAGGACAGCACCAAATGGAGGAAATTAGTAGATTTCAGrGAGCTCAAT
AAAAGAACTCAGGACTTTTGGGAAGTTCAATTAGGAATACCACATCCAGCAGGATTAAAA
AAGAAAAAATCAGTGACAGTACTAGATGTGGGAGATGCATATTTTTCAGTCCCTTTAGAT
rAAAACTTTAGAAAGTATACTGCATTCACCATACCTAGTAyAAACAATGAGACACCAGGA
ATYAGATATCAGTACAATGTGCTACCACAGGGATGGAAAGGATCTCCGGCAATATTCCAG
TGTAGCATGACAAAAATATTAGAGCCCTTTAGAAGAAAAAATCCAGAGATGATTATCTAT
CAATATrTGGATGACTTGTATGTAGCATCTGATTTAGAAATAGGGCAGCACAGAACAAAA
ATAGATGAGCTGAGAGCTCATCTATTGAGCTGGGGATTTACTACACCAGACAAAAAGCAT
CAGAAGGAACCGCCATTTCTTTGGATGGGATATGAACTCCATCCGGACAGATGGACAGTC
CAGCCTATAGAACTGCCAGAAAAAGACAGCTGGACTGTCAATGATATACAGAAATTAGTG
GGAAAACTAAATTGGGCAAGTCAAATTTATGCAGGGATTAAGGTAAAGCAACTGTGTAGA
CTCCTCAGGGGAGCTAAAGCATTAACAGAAGTAGTACCACTGACTGAAGAAGCAGAG

>732
CCTCAAATCACTCTTTGGCAGCGACCCCTCGTCTCAATAAAGATAGGGGGGCAACAAAAG
GAAGCTCTATTAGATACAGGAGCAGATGATACAGTATTAGAAGAAATGCATTTACCAGGA
AAATGGAAACCAAAAATGATAGGGGGAATTGGAGGTTTTATCAAAGTAAGACAGTATGAT
CAGATACTCATAGAAATTTGTGGCyATAAAGCTATAGGTACAGTATTAATAGGACCTACA
CCTGTCAACATAATTGGAAGAAATCTGTTGACTCAGATTGGCTGCACTTTAAATTTTCCT
ATTAGTACTGTTGAAACTGTACCAGTAAAATTAAAGCCAGGTATGGATGGCCCAAAAGTT
AAACAATGGCCAyTGACAGAAGAAAAAATAAAAGCATTAGTAGAAATTTGTACAGAAATG
GAGAAGGAAGGAAAAATTTCAAAAATAGGGCCTGAAAATCCATACAATACTCCAGTATTT
GCAATAAAGAAAAAAGACAGyACTAAATGGAGAAAATTAGTAGATTTCAGAGAACTCAAT
AAAAGAACTCAAGACTTCTGGGArGTTCAATTAGGAATACCACATCCCGsAGGGTTAAAA
AAGAAmAAATCAATAACAGTACTGGATGTGGGTGATGCATATTTTTCAGTTCCCTTATAT
GArGACTTTAGGAAGTATACTGCATTTACCATACCyAGTACAAACAATGAGACACCAGGG
ATTAGGTATCAGTACAATGTGCTTCCACArGGATGGAArGGATCACCAGCAATATTyCAA
AGTAGCATGACAAAAATCTTAGATCCTTTTAGAAAACAAAATCCAGACATAGTGATCTrT
CAGTACATGGATGAyTTGTATGTAGsATCTGACTTAGAAATAGGGCAACATAGAACAAAA
GTAGAGGAACTGAGACAGCATCTGTTGArGTGGGGATTAACCACACCAGACAAAAAAyAT
CAGAAAGAACCTCCATTCCTTTGGATGGGTTATGAACTCCATCCTGATAAATGGACAGTA
CAGCCTATAGTGyTGCCAGAAAAGGACAGCTGGACTGTCAATGACATACAGAArTTAGTA
GGAAAACTGAATTGGGCAAGTCAAATTTATGCAGGGATTAAAGTAAAGCAATTATGTAAA
CTCCTTAGGGGAGCCAAATCACTAACAGAAGTAGTACCACTAACACATGAAGCAGAG

>734
CCTCAAATCACTCTTTGGCAACGACCCCTTGTCACAATAAAAATAGGAGGACAGCTGAAA
GAAGCTCTATTAGATACAGGAGCAGATGATACAGTATTAGAAGATATAAATTTGCCAGGA
AAATGGAAACCAAAAATGATAGGGGGAATTGGAGGTTTTATCAAAGTAAGGCAATATGAT
CAGATACTTATAGAAATTTGTGGAAAAAAGGCTATAGGTACAGTGTTAGTAGGACCTACA
CCTGTCAACATAATTGGACGAAATATGTTGACTCAGATTGGTTGTACTTTAAACTTTCCA
ATTAGTCCTATTGACACTGTACCAGTAACATTAAAGCCAGGAATGGATGGACCAAAGGTT
AAACAGTGGCCATTGACAGAAGAAAAAATAAAAGCATTAACAGAAATTTGTAGGGAAATG
GAAGAGGAAGGAAAAATTTCAAAAATTGGGCCTGAAAATCCATATAATACTCCAGTATTT
GCTATAAAGAAAAAGGACAGCACCAAATGGAGGAAATTAGTAGATTTCAGAGAGCTCAAT
AAAAGAACTCAGGACTTTTGGGAAGTCCAATTAGGGATACCGCATCCAGCAGGATTAAAA
AAGAAAAAATCAGTGACAGTACTAGATGTGGGAGATGCATATTTTTCAGTCCCTTTAGAT
GAAAGCTTTAGAAAGTATACTGCATTCACCATACCTAGTATAAACAATGAGACACCAGGA
ATYAGATATCAGTACAATGTGCTACCACArGGATGGAAAGGATCTCCGGCAATATTCCAG
TGTAGCATGACAAAAATCTTAGAGCCCTTTAGAAAACAAAATCCAGAGATAGTTATCTAT
CAATACATGGATGACTTGTATGTAGGATCTGATTTAGAAATAGGGCAGCACAGAACAAAA
ATAGATGAGCTAAGAGCTCATCTATTGAGCTGGGGATTTACTACACCAGACAAAAAGCAT
CArAAGGAACCGCCATTTCTTTGGATGGGATATGAACTCCATCCGGACAGrTGGACAGTC
CAGCCTATAGAACTGCCAGAAAAAGACAGCTGGACTGTCAATGATATACAGAAATTAGTG
GGAAAACTAAATTGGGCAAGTCAAATATATGCAGGGATyAAGATAArGCAACTGTGTAAA
CTCCTyAGGGGAGCTAAAGCATTAACAGATGTAGTACCACTGACTGAAGAAGCAGAG

>735
CCTCAAATCACTCTTTGGCAACGACCCCTTGTCACAATAAAAATAGGAGGACAGYTAAGA
GAAGCTCTATTAGATACAGGAGCAGATGATACAGTATTAGAAGATATAAATTTGCCAGGA
AAATGGAAGCCAAAAATGATAGGGGGAATTGGAGGTTTTATCAAGGTAAGGCAATATGAT
CAGATACTTATAGAAATTTGTGGAAAAAAGGCTATAGGTACAGTrTTAGTAGGACCrACA
CCTGTCAACATAATTGGACGAAATATGTTGACTCAGCTTGGTTGTACTTTAAATTTCCCA
ATTAGTCCTATTGACACTGTACCAGTAACATTAAAGCCAGGAATGGATGGACCAAAGGTT
AAACAGTGGCCATTGACAGAAGAAAAAATAAAAGCATTAACAGAAATTTGTAAAGAGATG
GAAGAGGAAGGAAAGATCTCAAAAATTGGGCCTGAGAATCCATATAATACTCCAGTATTT
GCTATAAAGAAAAAGGACAGCACCAAATGGAGGAAATTAGTAGATTTCAGAGAGCTCAAT
AAAAGAACTCAGGATTTTTGGGAAGTTCAATTAGGGATACCGCATCCAGCAGGATTAAAA
AAGAAAAAATCAGTrACAGTACTAGATGTGGGAGATGCCTATTTTTCArTTCCTTTAGAT
AAAAGCTTTAGAAAGTATACTGCATTCACCATACCTAGTATAAAyAATGAGACACCAGGA
ATCAGATATCAGTATAATGTGCTACCACAAGGATGGAAAGGATCTCCGGCAATATTCCAG
TGTAGCATGACAAAAATCTTAGAGCCCTTTAGAAGAAAAAATCCAGAGATGATTATCTAT
CAATACATGGATGACTTGTATGTAGGATCTGATTTAGAAATAGGGCAGCACAGArCAAAA
ATAGAGGAGCTAAGAACTCATCTATTGAGCTGGGGATTyACTACACCAGACAAAAAACAT

CAGAAGGAACCTCCATTTCTTTGGATGGGATATGAACTCCATCCAGATAGATGGACAGTC
CAGCCTATAGAACTGCCAGAAAAAGACAGCTGGACTGTCAATGATATACAGAAATTAGTG
GGAAAATTAAATTGGGCAAGTCAAATTTATGCAGGGATTAAGGTAAAGCAACTGTGTAGA
CTCCTCAGGGGAGCTAAAGCACTAACAGACATAGTACCACTGACTGAAGAAGCAGAG

>739
GCTCTCTTAGACACAGGAGCAGATGATACAGTATTAGAAGAAGTAAATyTGCCAGGAAAA
TGGAAACCAAGAATGATAGGAGGAATTGGGGGTTTTATCAAAGTAAGrCAATATGAGCAA
ATACCTATAGAAATTTGTGGAAAAAAGGCTATAGGTACAGTATTAGTGGGACCCACACCT
GTCAACATAATTGGAAGAAATATGTTGACCCAACTTGGATGCACACTAAATTTTCCAATC
AGTCCCATTGAAACTGTACCAGTAAAATTAAAGCCAGGAATGGATGGCCCAAATTGTGAT
GAAATGGAgAAAGAAGGAAAAATTACAAAAATTGGGCCTGACAATCCATATAATACTCCA
ATATTTGCTATAAAAAAGAAGGAyAGCACTAAgTGGAGAAAATTAGTAgAyTTCAGGGAA
CTCAATAAAAGrACTCAAGATTTTTGGGAArTTCAATTAGGAATACCACACCCAGCAGGr
TTAAAAmAGAAmAAATCAGTAACAGTTCTGGATGTrGGTGATGCATATTTCTCAGTTCCT
TTAGATAAAGACTTCAGGAAGTATACTGCATTTACCATACCTAGTATAAACAATGAGACw
CCAGGGATTAGATATCAGTAyAATGTGCTTCCACAGGGATGGAAAGGATCACCAGCAATA
TTCCAAAGTAGCATGACAAAAATCTTAGAGCCTTTTAGAAArCAAAATCCAGACATAGTT
ATCTATCAATAyATGGATGACTTGTATGTAGGATCTGACTTAGAAATAGGGCAGCATAGA
ACAAAAATAGAGGAACTGAGAGmACAyCTGTTAAAGTGGGGGTTTACTACACCAGACAAG
AAACATCAGAAAGAACCTCCATTTCTTTGGATGGGGTATGAACTCCATCCTGACAAATGG
ACAGTACAGCCTATACAGCTGCCAGAAAAGGATAGCTGGACTGTCAATGATATACAGAAG
TTAGTGGGAAArTTAAAyTGGGCAAGTCAGATTTACCCAGGAATTAAAGTAAAGCAACTT
TGTAAACTCCTTAGGGGrACCAAAGCACTAACAGACATAGTACCACTAACTGAAGAAGCA
GAG

>751
CCTCAAATCACTCTTTGGCAACGACCCCTTGTCACAATAAAAATAGGAGGACAGCTGAAA
GAAGCTCTATTAGATACAGGRGCAGATGATACAGTATTAGAAGAyATAAATTTGCCAGGA
AAATGGAAACCAAAAATGATAGGGGGAATTGGAGGTTTTATCAAGGTAAGGCAATATGAT
CAGATACTTATAGAAATTTGTGGAAAAAAGGCTATAGGTACAGTATTAGTAGGACCTACA
CCTGTCAACATAATTGGACGAAATATGTTGACTCAGATTGGTTGTACTTTAAATTTCCCA
ATTAGTCmTATTGAmACTGTACCAGTGAmATTrAAGCCAGGrATGGATGGACCAAAAGTT
AAACAGTGGCCATTAACAGAAGAAAAAATAAAAGCATTAACAGAAATTTGTAAAGAGATG
GAAGAGGAAGGAAAAATCTCAAAAATTGGGCCTGAAAATCCATATAATACTCCrGTATTT
GCyATAAAGAAAAAGGACAGCACCAAATGGAGGAAGTTAGTAGATTTyAGAGAGCTCAAT

AAAAGAACTCAGGACTTTTGGGAAGTTCAATTAGGAATACCACATCCAGCAGGATTAAAA
AAGAAAAAATCAgTGACAGTACTAgATGTGGGAgATGCAtATTTTTCAgTTCCTTTAgAT
RAAAATTTTAGAAAATATACTGCATTCACCATACCTAGTATAAAyAATGAGrCACCAGGA
ATCAGATATCAGTACAATGTGCTACCACAGGGATGGAAAGGGTCTCCGGCAATATTCCAG
TGTAGCATGACAAAAATCTTAGAGCCCTTTAGAAAGAAAAATCCAGAGATGGTTATCTAT
CAATACATGGATGACTTATATGTAGGATCTGATTTAGAAATAGGGCAGCACAGAACAAAA
ATAGAGGAGCTAAGAGCTCATCTATTGAGCTGGGGATTTACTACACCAGACAAAAAGCAT
CAGAAGGAACCTCCATTTCTTTGGATGGGATATGAACTCCATCCGGACAGATGGACAGTC
CAGCCTATAGAACTGCCAGAAAAAGACAGCTGGACTGTCAATGATATACAGAAATTAGTG
GGAAAACTAAATTGGGCAAGTCAAATTTATGGAGGGATTAAGGTAAAGCAACTGTGTAAA
CTCCTCAGGGGAACCAAAGCACTAACAGACATAGTACCACTGACTGAAGAAGCAGAG

>762
CCTCAAATCACTCTTTGGCAACGACCCCTTGTTACAATAAAGATAGGGGGGCAATTAAAG
GAAGCTCTATTAGATACAGGAGCAGATGATACAGTATTAGAAGACATGAATTTGCCAGGG
AAATGGAAACCAAAAATGATAGGGGGAATTGGAGGTTTTATCAAAGTAAGACAGTATGAA
GAGATACCCATAGAAATCTGTGGACATAAAGTTATAGGTACAGTATTAATAGGACCTACA
CCTGTCAACATAATTGGAAGAAATCTGTTGACTCATCTTGGTTGTACTTTAAATTTTCCA
ATCAGTCCTATTGAAACTGTACCAGTAAAACTAAAGCCAGGAATGGATGGCCCAAAGGTT
AAACAATGGCCATTGACAAAAGAAAAAATAGAAGCATTAACAGCAATCTGTGATGAAATG
GAAAAGGAAGGAAAAATTACAAAAATTGGGCCTGAAAATCCATACAAyACTCCAATATTT
GCCATAAAAAAGAAAGACAGTACTAAGTGGAGAAAATTAGTAGATTTCAGGGAACTCAAT
AAAAGAACTCAAGATTTTTGGGAAGTTCAATTAGGAATACCACACCCAGCAGGATTAAAA
AAGAAAAAATCAGTGACAGTGCTGGATGTGGGsGATGCATATTTTTCAGTTCCTTTATAT
GAAGACTTCAGGAAATATACTGCATTCACCATACCTAGTATAAACAATGAAACACCAGGG
ATTAGGTATCAGTACAATGTACTTCCACAGGGATGGAAAGGATCACCAGCAATATTTCAA
AGTAGCATGACAAAAATCTTAGAGCCTTTTAGAAAACAAAATCCAGACATAGwCATCTAT
CAATACATGGATGATTTGTATGTAGGATCTGACTTAGArATAGGGCAGCATAGAACAAAA
ATAGAGGAACTGAGACAACATTTGTTGAGGTGGGGATTTACCACACCAGACAAGAAACAT
CAGAAAGAACCTCCATTTCTTTGGATGGGGTATGAACTCCATCCTGACAAATGGACAGTA
CAGCCTATACAGCTGCCAGTGCAAGATAGCTGGACTGTCAATGATATACAAAAGTTAGTG
GGAAAATTAAACTGGGCAAGTCAGATTTATCCTGGAATTAArGTAAGGCAACTTTGTAAA
CTCCTTAGGGGGACCAAAGCACTAACAGACATAGTACCACTAACTGAAGAAGCAGAG
