# Supplementary material for: HIV drug resistance in HIV positive individuals under antiretroviral treatment in Shandong Province, China
Source: PLoS One. 2017 Jul 27;12(7):e0181997. doi: 10.1371/journal.pone.0181997 (PMC5531464; doi:10.1371/journal.pone.0181997)
Supplement: S2 File — (PDF) [file pone.0181997.s002.pdf]

>6

CCTCAAATCACTCTTTGGCAACGACCCCTCGTCACAATAAAGATAGGGGGGCAATTAAAG
GAAGCTCTATTAGATACAGGAGCAGATGATACAGTATTAGAAGACATGAATTTGCCAGGA
AAATGGAAACCAAAAATGATAGGGGGAATTGGAGGTTTTATCAAAGTAAGACAGTATGAT
CAGATACCCATAGAAATCTGCGGACACAAGGCTGTAGGTACAGTATTAATAGGACCTACA
CCTGTCAACATAATTGGGAGAAATCTGTTGACTCAGCTTGGTTGCACTTTAAATTTTCCC
ATTAGTCCTATTGAAACTGTACCAGTAAAATTAAAGCCAGGAAyGGATGGCCCAAAAGTT
AAACAATGGCCATTGACAGAAGAAAAAATAAAAGCATTAGTAGAAATTTGTACAGAAATG
GAAAAGGAAGGGAAAATTTCAAAAATCGGGCCTGAAAATCCATACAATACTCCAGTATTT
GCCATAAAGAAAAAAGACAGTACTAAATGGAGAAAATTAGTAGATTTCAGGGAACTTAAT
AAGAGAACTCAAGACTTCTGGGAAGTTCAATTAGGAATACCACATCCCGCAGGGTTAAAA
AAGAAAAAATCCGTAACAGTCCTGGATGTGGGTGATGCATATTTCTCAGTTCCTTTAGAT
AAAGACTTCAGGAAGTATACTGCATTTACCATACCTAGTGTAAACAATGAGACACCAGGG
ATCAGATATCAATACAATGTGCTTCCACAGGGATGGAAAGGATCACCAGCAATATTCCAA
TGTAGCATGACAAAAATTTTAGAGCCTTTTAGAAAACAAAATCCAGACATAGTTATCTAT
CAATATATGGATGATTTGTATGTAGGATCTGATTTAGAAATAGGGCAGCATAGAGCAAAA
ATAGAGGAACTGAGACAACATCTGTTGAGGTGGGGATTTACCACACCAGACAAAAAACAT
CAGAAAGAACCTCCATTCCTTTGGATGGGTTATGAACTCCATCCTGATAAATGGACAGTA
CAGCCTATAGTGCTGCCAGAGAAGGACAGCTGGACTGTCAATGACATACAAAAGTTAGTG
GGAAAATTGAATTGGGCAAGTCAGATTTATGCAGGAATTAAGGTAAGGGCATTATGTAAA
CTCCTTAGGGGGACCAAAGCACTAACAGAAGTAATACCACTAACAGAAGAAGCAGAG

>9

CCTCAAATCACTCTTTGGCAACGACCCCTCGTCACAGTAAAGATAGGGGGGCAATTAAAG
GAAGCTCTATTAGATACAGGAGCAGATGATACAGTATTAGAAGACATGAATTTGCCAGGG
AAATGGAArCCAAAAATGATAGGGGGAATTGGAGGTTTTATCAAAGTAAGACAGTATGAA
CAGRTACCCrTAGAAATTTGCGGACACAAAGCTATAGGTACAGTATTAGTCGGrCCTACA
CCTGTCAACATAATTGGAAGAAATCTGTTGACTCAGATTGGCTGCACkTTAAATTTTCCr
ATCAGTCCCATTGAAACTGTACCAGTAmAATTAAAGCCAGGAATGGATGGCCCCAAGGTT
AAACAATGGCCATTGACAGAAGAGAAAATAAAAGCATTAACArCAATTTGTGAGGAAATG
GAGAAAGAAGGAAAAATTACAArAATTGGGCCTGAAAATCCATATAACACTCCAATATTT
GCCATAAAAAAGAAAGATAGyACTAAATGGAGrAAGCTAGTAGATTTCAGGGAACTCAAT
AAAAGAACCCAAGATTTTTGGGAAGTTCAATTAGGGATACCACACCCAGCAGGGTTAAAA
AAGAAAAAATCAGTGACAGTACTGGATGTGGGGGATGCATATTTTTCAGTTCCTTTATAT
GAGGACTTCAGGAARTATACTGCATTCACCATACCTAGTACAAACAATGAAACACCAGGG
ATTAGGTATCAGTACAATGTACTTCCACAGGGATGGAAAGGATCACCAGCAATATTCCAA
AATAGCATGACAAAAATCTTAGAGCCTTTTAGAAAACAAAATCCAGACATAGTTATyTAT
CAATACATGGATGATTTGTATGTAGGATCTGACTTAGAGATAGGGCAGCATAGAAyAAAA
ATAGAGGAACTGAGAsAACATTTGTTGAGGTGGGGATTTACCACACCAGACAAGAAACAT

CAGAAAGAACCTCCATTTCTTTGGATGGGGTATGAACTCCATCCCGACAAATGGACAGTG
CAGCCTATACAGCTGCCAGArArAGAyAGCTGGACTGTCAATGATATACAAAAGTTAGTG
GGAAAATTAAACTGGGCAAGTCAGATTTACCCTGGAATTAAAGTAAAGCAACTTTGCAAA
CTCCTTAGGGGGACCAAAGCACTAACAGACATAGTACCACTAACTGAAGAAGCAGAG

>14
CCTCAGATCACTCTTTGGCAACGACCCCTyGTCTCAATAAGAGTAGGGGGCCAGACAAAA
GAGGCTCTCCTAGATACAGGAGCAGATGATACAGTATTAGAAGAAGTAAATTTGCCAGGC
AAATGGAAACCAAAAATGATAGGGGGAATTGGAGGTTTTATCAAAGTAAGACAGTATGAT
CAGGTACCCATAGAAATTTGCGGACACAAAGCTATAGGTACAGTATTAGTAGGACCTACA
CCTGTCAACATAATTGGAAGAAATCTGTTGACTCAGCTkGGTTGCACTTTAAATTTTCCC
ATTAGTCCTATTGAAACGGTACCAGTAAAATTAAAGCCAGGAATGGATGGCCCAAAGGTT
AAACAGTGGCCATTGACAGAAGAAAAAATAAAAGCATTAACAGAAATTTGCAAAGAAATG
GAAGAGGAAGGGAAAATCTCAAAAATTGGGCCTGAAAATCCATACAATACTCCAGTATTT
GCTATAAAGAAAAAGGACAGCACCAAATGGAGGAAACTAGTAGATTTCAGAGAGCTCAAT
AAAAGAACTCAGGATTTTTGGGAAGTTCAATTAGGAATACCACATCCAGCAGGATTAAAA
AAGAAAAAATCAGTAACAGTACTAGATGTGGGAGATGCATAyTTTTCAGTTCCTTTAGAT
GAAArCTTTAGAAAGTAyACTGCATTCACCATACCTAGTATAAACAATGAGACACCAGGA
ATCAGATATCArTACAATGTGCTGCCACAGGGATGGAAAGGATCACCAGCAATATTCCAG
ArTAGCATGACAAAAATCTTAGAGCCCTTTAGAGyAAAAAATCCAGAAATAATTATCTAT
CAATACATGGATGACTTGTATGTAGGATCTGATTTAGAAATAGGGCAGCATAGAACAAAA
ATAGAGGAGCTAAGAGCTCATCTATTGAGCTGGGGGTTTACTACACCAGACAAAAAGCAT
CAGAAGGAACCCCCATTCCTTTGGATGGGATAyGAACTCCATCCTGATAGATGGACAGTY
CAGCCTATAAAACTGCCAGAAAAAGACAGCTGGACTGTCAATGATATACAGAAATTAGTG
GGAAAAyTAAATTGGGCAAGTCArATTTATGCAGGrATTAAGGTAAAGCAACTGTGTAAA
CTyCTTAGGGGGACTAAAGCACTAACAGACATAGTACCAyTGACTGAAGAAGCAGAG

>15
CCTCAAATCACTCTTTGGCAACGACCCryTGTYGCAATAAGAGTAGGAGGCCAGATAAAA
GAGGCTCTATTAGACACAGGAGCAGATGATACAGTATTAGAArAAATAAATTTGCCAGGA
AAATGGAAACCAAAAATGATAGGGGGAATTGGAGGATTTATCAAAGTAAGACAGTATGAT
CAAATACCTATAGAAATTTGTGGAAAAAAGGCTATAGGTACAGTATTAGTAGGACCTACA
CCTGTCAACATAATTGGAAGAAATCTGTTGACTCAGCTTGGTTGCACTTTAAATTTTCCC
ATTAGTCCTATTGAAACTrTACCAGTAAAATTAAAGCCAGGmATGrATGGCCCAAAAGTT
AAACAATGGCCATTGACAGAAGAAAAAATAAAAGCATTAACAGAAATTTGTATGGAAATG
GAAAAGGAGGGAAAAATTTCAAAAATTGGGCCTGAAAATCCATACAATACTCCAGTATTT

GCCATAAAGAAGAAAGATAGTACTAAGTGGAGAAAATTAGTAGATTTCAGGGAACTCAAT
AAGAGAACTCAAGATTTTTGGGAAGTCCAATTAGGAATACCACACCCGGCAGGGTTAAAA
AAGAAAAAATCAGTGACAGTACTGGATGTGGGGGATGCATATTTTTCAGTTCCTTTATAT
GAAGACTTCAGGAAATATACTGCATTCACCATACCTAGTAGAAACAATGAAACACCAGGG
ATTAGGTATCAGTACAATGTGCTTCCACAAGGATGGAAAGGATCACCAGCAATATTCCAG
TGTAGCATGACAAAGATTTTAGAGCCTTTTAGAAAACAAAATCCAGACATAGTTATCTAT
CAATACATGGATGATTTGTATGTAGGATCTGACTTAGAAATAGGGCAACATAGAGCAAAA
ATAGAAGAGTTAAGAGAACATCTGTTAAAGTGGGGGTTTACCACACCAGACAAGAAACAT
CAGAAAGAACCTCCATTTCTTTGGATGGGGTATGAACTCCATCCTGACAAATGGACAGTA
CAGCCTATACAGCTGCCAGAACGGGATAGCTGGACTGTCAATGATATACAGAAGTTArTG
GGAAAATTAAACTGGGCAAGTCAGATATATCCTGGAATTAAAATAAGGCAACTTTGTAAA
CTCCTTAGGGGGGCCAAAGCACTAACAGACATAGTACCACTAACTGAAGAAGCAGAG

>16
CCTCAGATCACTCTTTGGCAACGACCCCTTGTCTCAATAAGAGTAGGGGGCCAGACAAAA
GAGGCTCTCCTAGATACAGGAGCAGATGATACAGTATTAGAAGAAGTAAATTTGCCAGGC
AAATGGAAACCAAAAATGATmGGGGGAATTGGAGGTTTTATCAAAGTAAGACAGTATGAT
CArGTACCCATAGAAATTTGCGGrCACAAAGCTATAGGTACAGTATTArTAGGACCTACA
CCTGTCAACATAATTGGAAGAAATCTGTTGACTCAGCTTGGTTGCACTTTAAATTTTCCC
ATTAGTCCTATTGAAACGGTACCAGTAAAATTAAAGCCAGGAATGGATGGCCCAAAGGTT
AAACAGTGGCCATTGACAGAAGAAAAAATAAAAGCATTAACAGAAATTTGCAAAGAAATG
GAAGAGGAAGGGAAAATCTCAAAAATTGGGCCTGAAAATCCATACAATACTCCAGTATTT
GCTATAAAGAAAAAGGACAGCACCAAATGGAGGAAACTAGTAGATTTCAGAGAGCTCAAT
AAAAGAACTCAGGATTTTTGGGAAGTTCAATTAGGAATACCACATCCAGCAGGATTAAAA
AAGAAAAAATCAGTAACAGTACTAGATGTGGGAGATGCATAyTTTTCAGTTCCTTTAGAT
GAAArCTTTAGAAAGTATACTGCATTCACCATACCTAGTATAAACAATGAGACACCAGGA
ATCAGATATCAGTACAATGTGCTGCCACAGGGATGGAAAGGATCACCAGCAATATTCCAG
AGTAGCATGACAAAAATCTTAGAGCCCTTTAGAGyAAAAAATCCAGAAATAATTATCTAT
CAATACATGGATGACTTGTATGTAGGATCTGATTTAGAAATAGGGCAGCATAGAACAAAA
ATAGAGGAGCTAAGAGCTCATCTATTGAGCTGGGGGTTTACTACACCAGACAAAAAGCAT
CAGAAGGAACCyCCATTCCTTTGGATGGGATAyGAACTCCATCCTGATAGATGGACAGTC
CAGCCTATAAAACTGCCAGAAAAAGACAGCTGGACTGTCAATGATATACAGAAATTAGTG
GGAAAAyTAAATTGGGCAAGTCArATTTATGCAGGrATTAAGGTAAAGCAACTGTGTAAA
CTyCTTAGGGGGACTAAAGCACTAACAGACATAGTACCAyTGACTGAAGAAGCAGAG

>17

CCTCAAATCACTCTTTGGCAACGACCCCTCGTCACAATAAAGATAGGGGGGCAATTAAAG
GAAGCTCTATTAGAyACAGGAGCAGATGATACAGTATTAGAAGACATGAATTTGCCAGGA
ArATGGAAACCAAAAATGATAGGGGGAATTGGAGGTTTTATCAAAGTAAGACArTATGAk
mAGrTACCCATAGAAATCTGyGGACAyAAGGCyATAGGTACAGTATTAATAGGACCTACA
CCTrTCAACATAATTGGAAGAAATCTGTTGACTCAGCTTGGTTGCACTTTAAATTTTCCC
ATTAGTCCTATTGAAACTGTACCAGTAAAATTAAAGCCAGGAATGGATGGCCCAAAAGTT
AAACAATGGCCATTGACAGAAGArAAAATAAAAGCmTTAGTAGAAATTTGTACAGArATG
GAAAAGGAAGGGAAAATTTCAAAAATCGGGCCTGAAAATCCATAyAATACTCCAGTATTT
GCCATAAAGAAAAAAGAyAGTACTAAATGGAGAAArTTAGTAGATTTCAGrGAACTTAAT
AAAAGAACyCArGACTTCTGGGAAGTTCAATTAGGAATACCACATCChGCAGGGTTAAAr
AAGAAAAAATCTGTAACAGTCCTGGATGTGGGTGATGCATATTTCTCAGTYCCTTTAGAT
AAAGACTTCAGGAAGTATACTGCATTyACCATACCTAGTGTAAACAATGAGACACCAGGG
ATCAGATATCAGTACAATGTGCTTCCACAGGGwTGGAAAGGrTCACCAGCAATATTCCAA
TGTAGCATGACAAArATyTTAGAGCCTTTTAGAAAACArAATCCAGAyATAGTTATCTAT
CAATACATGGATGATyTGTATGTAGGATCTGAyTTAGAAATAGGrCAGCATAGAGCAAAA
ATAGAGGAACTGAGACAACATTTGTTGAGGTGGGGATTTACCACACCAGACAAAAAACAT
CAGAAAGAACCTCCATTCCTTTGGATGGGTTATGAACTCCATCCTGATAAATGGACAGTA
CAGCCTATAGWGCTGCCAGArAAGGACmKCTGGACTGTCAATGACATACAGAAGTTAGTG
GGAAAGTTrAATTGGGCAAGyCArATTTATGCAGGGATyAAGGTAAAGGAATTATGTAAA
CTCCTTAGGGGArCCAAAGCACTAACAGArGTAATACCACTAACAGAAGAAGCAGAG

>18
CCTCAAATCACTCTTTGGCAACGACCCmTCGTCCCAATAAGGGTAGGGGGGCAATTAAAG
GAAGCTCTATTAGATACAGGAGCAGATGATACAGTvTTAGAAGACATGAATTTGCCAGGA
AAATGGAGACCAArAATGATAGGGGGAATTGGAGGyTTTATCAAAGTAAGACAGTAyGAT
CAGATACCCATAGAAATyTGyGGACACAAAGCTATAGGTACAGTATTAGTAGGACCTACA
CCTGTCAACATAATTGGAAGrAATTTGTTGACTCAGCTTGGTTGYACTTTAAATTTTCCC
ATTAGTCCTATTGAwACTGTACCAGTAAAAyTAAArCCAGGAATGGATGGCCCkAAAGTy
AAACAATGGCCATTGACAGAAGArAAAATAAAAGCATTAryAGAAATTTGTACAGAAATG
GAAAAGGAAGGGAAAATTTCAAAAATyGGGCCTGAAAATCCATACAATACTCCAGTATTT
GCyATAAAGAAAAAAGACAGTACCAAATGGAGAAAATTAGTAGATTTCAGrGAACTTAAT
AAAAGAACTCAAGACTTCTGGGAAGTTCAATTAGGAATACCACAyCCyGCAGGryTAAAA
AAGAAmArATCyrTAACAGTCCTAGATGTGGGTGATGCATATTTCTCmGTyCCTTTAGAT
GAAGACTTCAGGAAGTATACTGCATTTACCATACCTAGTkTAAACAATGAGACACCAGGG
GTTAGATATCAGTACAATGTGCTTCCACAGGGATGGAAAGGvTCACCAGCAATATTCCAA
TsTAGCATGACAAAAATCTTAGAkCCyTTTAGAAAACrAAATCCAGATmTArTTATCTrT
CAATACATGGATGATTTGTATGTAGGrTCTGACTTAGAAATAGGrCAGCATAGAACAAAA
ATAGAGGAACTGAGAvAACATCTGwTGryGTGGGGATTTACCACACCAGACAAMAAAYAT
CAGAAAGAAmCTCCAyTCCkTTGGATGGGTTATGAACTCCATCCAGATAAATGGACAGTA
CAGCCTATAATACTGCCAGAAAArGACAGCTGGACTGTCAATGACATACArAAGTTAGTG
GGrAArTTRAATTGGGCAAGTCAGATTTATGCAGGRATTAAGGTAAArGAATTATGTAAA

CTCCTTAGGGGAACCAAAGCAyTAACAGAAGTAGTACCACTrACAGAAGAAGCAGAG

>19
CCTCAAATCACTCTTTGGCAACGACCCmTCGTCACAATAAAGATAGGGGGGCAATTAAAG
GAAGCTCTrTTAGATACAGGAGCAGATGATACAGTATTAGAAGACATGAATTTGCCAGGA
AGATGGAAACCAAAAATGATAGGGGGAATTGGAGGTTTTATCAAAGTAArACAGTATGAT
CAGATACCCATAGAAATCTGTGGrCACAArRCTGwrGGTACAGTATTAATAGGACCTACA
CCTrTCAACATAATwGGrAGrAATCTGTTGACWCAGCTTGGGTGCACTTTAAATTTTCCC
ATTAGTCCTATTGAAACTGTACCAGTAAAATTAAAGCCAGGrATGGATGGCCCAAArGTT
AAACAATGGCCATTGACAGAAGAAAAAATAAAAGCATTArTAGAAATTTGTACAGAAATG
GAAAAGGAAGGrAArATTTCAAAAATTGGGCCTGArAATCCATACAATACTCCAGTATTT
GCmATAAAGAAAAAAGAyAGTACTAAATGGAGAAAATTAGTAGATTTCAGrGAACTTAAT
AAAAGAACTCAAGACTTCTGGGAAGTTCAATTAGGAATACCACATCCmGCAGGGTTrAAA
AAGAAAAAATCTGTAACAGTmyTGGATGTGGGTGATGCATAyTTCTCAGTyCCTTTAGAy
AAAGAyTTCAGGAAGTAyACTGCATTTACCATACCTAGTGTAAAyAATGAGACACCAGGG
AThAGATATCAGTACAATGTrCTTCCACAGGGATGGAAAGGATCACCAGCAATATTyCAA
TGTAGTATGACAAArATCTTAGArCCTTTTAGAAAACAAAATCCAGACATAGTTATCTAT
CAATACATGGATGATTTGTATGTAGGATCTGAyTTAGAAATAGGGCAGCAyAGAGCAAAA
ATAGAGGAACTGAGACArCATCTGTTrAGGTGGGGATTTACCACACCAGACAAAAAACAT
CAGAAAGAACCTCCATTyCTTTGGATGGGTTATGAACTCCATCCTGATAAATGGACAGTr
CAGCCTATAGAGCTGCCAGAAAAGGACAGCTGGACTGTCAATGACATACAGAAGTTAGTG
GGAAAGTTrAATTGGGCAAGTCAGATTTATGCAGGrATTAGGrTAmrrGAATTATGTAAA
CTCmTTAGGGGArCCAAAGCACTAACAGAAGTAGTACCACTAACAGAAGAAGCAGAG

>20
CCTCAAATCACTCTTTGGCAACGACCCCTAGTCACAATAAAAATAGGAGGACAGCTGAGA
GAAGCTCTATTAGATACAGGAGCAGATGATACAGTATTAGAAGATATAAATTTGCCAGGA
AAATGGAAACCAAAAATGATAGGGGGAATTGGAGGTTTTATCAAAGTAAGGCAATATGAT
CAGATACTTATAGAAATTTGTGGAAAACAGGCTATAGGTACAGTGTTAATAGGACCTACA
CCTGTCAACATAATTGGACGAAATATGTTGACTCAGATTGGTTGTACTTTAAATTTTCCA
ATTAGTCCTATTGAkACTGTACCAGTAACATTAAAACCAGGAATGGATGGACCAAAGGTT
AAACAATGGCCATTGACAGAAGAAAAAATAAAAGCATTAACAGAAATTTGTAGGGAGATG
GAAGAGGAAGGAAAAATCTCAAAGATTGGGCCTGAAAATCCATATAATACTCCAGTATTT
GCTATAAAGAAAAAGAACAGCACCGAATGGAGGAAATTAGTAGATTTCAGAGAGCTCAAT
AAAAGAACTCAGGATTTTTGGGAAGTTCAATTAGGAATACCGCATCCAGCAGGATTAAAA
AAGAGAAAATCAATGACAGTACTAGATGTGGGAGATGCATATTTTTCAGTCCCTTTAGAT

GAGAACTTTAGAAAGTATACTGCATTCACCATACCTAGTATAAATAATGAGACACCAGGA
ATCAGATATCAGTACAATGTGCTACCACAGGGATGGAAAGGATCTCCAGCAATATTCCAG
TGTAGCATGACAAAAATCTTAGAGCCCTTTAGAAGCAAAAATCCAGAGATAGATATCTAT
CAATATGTGGATGACTTGTATGTAGGATCTGATTTAGAAATAGGGCAGCACAGArCAAAA
ATAGAGGAGCTAAGAGCTCATCTATTAAGCTGGGGATTTACTACACCAGAyAAAAAGCAT
CAGAAGGAACCGCCACTTCTTTGGATGGGATATGAACTCCATCCGGACAGATGGACAGTC
CAGCCTATACAACTGCCAGAAAAAGACAGCTGGACTGTCAATGATATACAGAAATTAGTG
GGAAAACTAAATTGGGCAAGTCAAATTTATCCAGGGrTTAGGATAAAGCAATTGTGTAAA
CTCCTCAGGGGAACTAAAGCATTAACAGACGTAGTACCATTGACTGAAGAAGCAGAG

>21
CCTCAAATCACTCTTTGGCAACGACCCCTTGTCACAATAARAATAGGAGGACAGCTGAAA
GAAGCTCTATTAGATACAGGAGCAGATGATACAGTATTAGAAGATATAAATTTGCCAGGA
AArTGGAAACCAAAAATGATAGGGGGAATTGGGGGTTTTATCAAAGTAAGGCAATATGAT
CAGATACCTATAGAAATTTGTGGAAAACAGGCTATAGGTACAGTGTTAGTAGGACCTACA
CCTGTCAACATAATTGGACGAAATATGTTGACTCAGCTTGGTTGTACTTTAAAyTTTCCA
ATTAGTCCTATTGAAACTGTACCAGTAACATTAAAGCCAGGAATGGATGGACCAAAGGTT
AAACAGTGGCCATTGACArAAGAAAAAATAAAAGCATTAAyAGAmATTTGTAAGGArATG
GAAGCrGAAGGAAAAATCTCAAAAATTGGGCCTGAAAATCCATATAATACTCCAGTATTT
GCTATAAAGAAAAAGGACAGCACCAAATGGAGGAAATTAGTAGATTTCAGAGAGCTCAAT
AAAAGAACTCAGGACTTTTGGGAAGTTCAATTAGGAATACCACATCCAGCAGGATTAvmA
AAGAAAAAATCAGTrACAGTACTAGATGTGGGAGATGCATATTTTTCAGTCCCTTTAGAT
rAAAACTTTAGAAAGTATACTGCATTCACCATACCTAGTACAAACAATGAGACACCAGGA
ATyAGATATCAGTACAATGTGCTACCACAGGGATGGAAAGGATCTCCGGCAATATTCCAG
TGTAGCATGACAAAAATATTAGAGCCCTTTAGAAGAAAAAATCCAGAGATrATTATCTAT
CAATATGTGGATGACTTGTATGTAGCATCTGATTTAGAAATAGGGCAGCACAGAACAAAA
ATAGATGAGCTGAGAGCTCATCTATTGAGCTGGGGATTTACTACACCAGACAAAAAGCAT
CAGAAGGAACCGCCATTTCTTTGGATGGGATATGAACTCCATCCGGACAGATGGACAGTC
CAGCCTATAGAACTGCCAGAAAArGACAGCTGGACTGTCAATGATATACAGAAATTAGTG
GGAAAACTAAATTGGGCAAGTCAAATTTATGCAGGGATTAAGGTAAAGCAACTGTGTAGA
CTCCTCAGGGGAGCTAAAGCATTAACAGAAGTAGTACCACTGACTGAAGAAGCAGAG

>22
CCTCAGATCACTCTTTGGCAACGACCCCTTGTCTCAGTAAAAGTAGGGGGCCAGATAAAA
GAGGCTCTCTTAGACACAGGAGCAGATGATACAGTmTTAGAAGAAATAAATTTGCCAGGA
AAATGGAAACCAAAAATGATAGGAGGAATTGGAGGTTTTATCAAAGTAAGACAATATGAT

CAAATACCTATAGAAATTTGTGGAAAAAAGGCTATAGGTACAGTATTAGTGGGACCCACA
CCTGTCAACATAATTGGAAGAAATATGTTGACTCAACTTGGATGCACACTAAATTTTCCA
ATCAGTCCCATTGAAACTGTACCAGTAAArTTAAAGCCAGGrATGGATGGCCCAAAGGTT
AAACArTGGCCATTGACAGAAGAGAAAATAAAAGCATTAACAGCAATTTGTGATGArATG
GArAAGGAAGGAAAAATTTCAAAAATTGGGCCTGAAAAyCCATATAACACTCCAATATTT
GCCATAAAAAAGAAGGACAGTACTAAGTGGAGAAAATTAGTAGATTTCAGGGAACTyAAT
AAGAGAACyCAAGATTTTTGGGAAGTTCAATTAGGAATACCACACCCAGCAGGGTTAAAA
AAGAAAAAGTCAGTGACAGTACTGGATATAGGGGATGCATATTTTTCAGTACCTTTAGAT
GAAAACTTCAGGAAATATACTGCATTCACCATACCyAGTGTAAACAATGAAACACCAGGG
ATTAGrTAyCAATATAATGTGCThCCACArGGATGGAAAGGATCACCAGCAATATTCCAG
AGTAGCATGACAAGAATCTTAGAGCCCTTTAGrATAAAmAATCCAGAAATAGTCATCTAT
CAATATATGGATGACTTGTATGTAGGATCTGACTTAGAAATAGGGCAACATAGAGCmAAA
ATAGArGAGCTAAGAGAACATCTGTTArrGTGGGGAyTkACyACACCAGACAAAAAACAT
CArAAAGAACCCCCATTTCTTTGGATGGGGTATGAACTCCATCCTGACAAATGGACAGTA
CAGCCTATACAGCTGCCAGAAAAGGATAGCTGGACTGTCAATGATATACAGAAGTTAGTG
GGAAAATTAAACTGGGCAAGTCAGATTTACCCAGGAATTAAAGTAAGrCAACTyTGTAAA
CTCCTTAGGGgGrCyAAAGCACTAACAGATATAGTGCCACTAACTGAAGAAGCAGAG

>23
CCTCAAATCACTCTTTGGCAACGACCCCTCGTCACAATAAAGATAGGGGGGCAGTTAAAG
GAAGCTCTATTAGATACAGGAGCAGATGATACAGTATTAGAAGACATGGAkTTACCAGGA
AAATGGAAACCAAAAATGATAGGGGGAATTGGAGGTTTTATCAAAGTwArACAATATGAG
GAGGTACCCATAGACATCTGTGGACACAAAGCTATAGGTACAGTATTAGTAGGACCTACA
CCTGTCAACATAATTGGAAGAAATCTGTTGACTCAGCTTGGGTGTACyCTAAATTTTCCC
ATTAGTCCTATTGAAACTGTACCAGTAAAATTAAAGCCAGGAATGGATGGCCCAAAAGTT
AAACAATGGCCATTGACAGAAGAAAAAATAAAAGCATTAGTAGAAATTTGTrCAGAAATG
GAAAAGGAAGGrAAAATTTCAAAAATyGGGCCTGAAAATCCATACAATACTCCAGTATTT
GCCATAAAGAAAAAAGACAGTACTAAATGGAGAAAATTAGTAGATTTCAGGGAACTTAAT
AAAAGAACTCAAGACTTCTGGGArGTTCAATTAGGAATACCACATCCCTCAGGGTTAAAA
AAGAAAAAATCTGTAACAGTCCTGGATGTAGGTGATGCATATTTCTCAATTCCTTTAGAT
AAGGArTTCAGGAAGTATACTGCATTTACCATACCTAGTATAAACAATGAGACACCAGGA
ATTAGATATCAGTACAATGTGCTTCCACAGGGATGGAAAGGATCACCAGCAATATTCCAA
AGTAGCATGACAAAGATCTTAGAGCCTTTTAGAAAACAAAATCCAGATATAGTTATCTAT
CAATACATGGATGACTTGTATGTAGGATCTGATTTAGAAATAGAGCAGCATAGAGyAAAA
GTAGAGGAACTrAGAGAACATCTGTTGGGGTGGGGACTTACCACACCAGACAAAAAACAT
CAGAAAGAACCyCCATTCCTTTGGATGGGTTATGAACTCCATCCTGATAAATGGACAGTC
CAGCCTATAGAATTACCAGAAAAGGACAGCTGGACTGTCAATGATATACAGAAATTAGTG
GGAAAACTAAATTGGGCAAGTCAAATTTATGCAGGGATCAAGGTAArGCAATTGTGTArA
CTCCTCAGGGGAGCTAAAGCATTAACAGAAATAGTAmCATTAACTAAAGAAGCAGAG

>24
CCTCAAATCACTCTTTGGCAACGACCCCTTGTTACAGTAAAAATAGGAGGACAGyTAAAA
GAAGCTCTATTAGAyACAGGAGCAGATGATACAGTrTTAGAAGAGATAAATTTGCCAGGA
AAATGGAAACCAAAAATGATAGGGGGAATTGGAGGTTTTATCAArGTAAGRCAATATGAT
CAGATACTTATAGAAATTTGTGGAAAAAAGGCTATAGGTACAGTrTTAGTAGGACCCACA
CCTGTCAACATAATTGGACGAAATATGTTGACTCAGATTGGTTGTACTTTAAATTTCCCA
ATTAGTCCTATTGACACTGTACCAGTAACATTAAAGCCAGGAATGGATGGACCAAArGTT
AAGCAATGGCCATTAACCGAAGAAAAAATAAAAGCCTTAACAGAAATTTGTAATGAGATG
GArAAGGAAGGAAAAATCTCAAArATTGGGCCTGAAAAyCCATACAATACTCCAGTATTT
GCyATAAAGAAAAAGGACAGCACTAAATGGAGrAAATTAGTGGACTTCAGAGAGCTyAAT
AAAAGAACTCAGGAyTTTTGGGAAGTTCAATTrGGAATACCrCATCCAGCAGGyTTAAAA
AAGAAAAAATCAGTAACAGTACTAGATGTGGGAGATGCATATTTyTCAGTTCCTTTAGAT
GAAAGCTTTAGAAArTATACTGCATTTACCATACCyAGTACAAACAATGAGACACCRGGA
ATCAGrTATCAGTACAATGTGCTGCCACAGGGATGGAAAGGrTCACCAGCAATATTyCAG
TGTAGCATGACAAAAATCTTAGAGCCCTTTAGAATAAAAAATCCAGArATAGTTATCTAT
CAATACATGGATGACTTGTATGTAGGATCTGATTTAGAAATAGGGCAGCACAGAATAAAA
ATAGAAGAGCTAAGAAGTCATCTATTGAGCTGGGGATTTACTACACCAGATAAAAArCAT
CAGAAGGAACCTCCmTTCCTwTGGATGGGATATGAACTCCATCCTGACAGATGGACAGTC
CAGCCTGTAGAAyTGCCAGAAAAAGAYAGCTGGACTGTCAATGATATACAGAAATTAGTG
GGAAAACTAAATTGGGCAAGTCArATTTATGCAGGAATTAAAGTRAAGCAACTGTGTAAA
CTCCTCAGGGGAGCTAAAGCATTAACAGACATAGTACCACTGACTGAAGAAGCAGAG

>25
CCTCAAATCACTCTTTGGCAACGACCCCTTGTCACAATAAAAATAGGAGGACAGCTAAGA
GAAGCTCTATTAGATACAGGAGCAGATGATACAGTATTAGAAGATATAAATTTGCCAGGA
AAATGGAAGCCAAAAATGATAGGGGGAATTGGAGGTTTTATCAAGGTAAGGCAATATGAT
CAGATACCTATAGAAATTTGTGGAAAAAAGGCTATAGGTACAATATTAGTAGGACCTACA
CCTGTCAACATAATTGGACGAAATATGTTGACTCAGCTTGGTTGTACTTTAAATTTCCCA
ATTAGTTCTATTGACACTGTACCAGTAACATTAAAGCCAGGAATGGATGGACCGAAGGTT
AAACAGTGGCCATTAACAGAAGAAAAAATAAAAGCATTAACAGAAATTTGTArAGAGATG
GAAGAGGAAGGAAAAATCTCAAAAATTGGGCCTGAGAATCCATATAACACTCCAGTATTT
GCTATAAAGAAAAAGGACrGCACCAAATGGAGGAAATTAGTAGATTTCAGAGAGCTCAAT
AAAAGAACTCAGGACTTTTGGGAAGTTCAACTAGGGATACCGCATCCAGCAGGATTAAAA
AAGAAAAAATCAGTGACAGTACTAGATGTGGGAGATGCATATTTTTCAGTyCCTTTAGAT
AAAAGCTTTAGAAAGTATACTGCATTCACCATACCTAGTATAAACAATGAGACACCAGGA
ATCAGATATCAGTATAATGTGCTACCACAAGGATGGAAAGGATCTCCGGCAATATTCCAG

TGTAGCATGACAAAAATTTTAGAACCCTTTAGAAGAAAAAATCCAGAGATAGATATCTAT
CAATACATGGATGACTTGTATGTAGGATCTGATTTAGAAATAGGGCAGCACAGAAyAAAA
ATAGAsGAGCTAAGAGCTCATCTATTGAACTGGGGATTTACTACACCAGACAAAAAACAT
CAGAAGGAACCTCCATTTCTTTGGATGGGATATGAACTCCATCCGGATAGATGGACAGTC
CAGCCTATAGAACTGCCAGAAAAAGACAGCTGGACTGTCAATGATATACAGAAATTAGTG
GGAAAATTAAATTGGGCAAGTCAAATTTATGCAGGGATTAAGGTAAAGCAACTGTGTAGA
CTCCTCAGGGGAGCTAAAGCACTAACAGACATAGTACCACTGACTGAAGAAGCAGAG

>26
CCTCAAATCACTCTTTGGCAACGACCCGTTGTCACAGTAAAAATAGGrGGACAGCTGAGA
GArGCTCTATTAGATACAGGAGCAGATGATACAGTrTTAGAAGAAATAGATTTGCCAGGA
AAATGGAAACCrAAAATGATAGGGGGAATTGGAGGTTTTATCAArGTwAGGCAATATGAT
CAGATACTTATAGAAATTTGTGGAAAAArrGCTATAGGTACAGTGTTAGTTGGACCTACA
CCTGTCAACATAATTGGGCGAAACATGTTGACTCAGATTGGTTGTACTTTAAATTTCCCA
ATTAGTCCTATTGACACTGTACCAGTAAAATTAAAGCCAGGAATGGATGGGCCAAAGrTT
AAACAGTGGCCATTGACAGAAGAAAAAATAAAAGCATTAACAGAAATTTGTAArGAAATG
GAAGAGGAAGGAAAAATyTCAAAAATTGGGCCTGAAAATCCATATAATACTCCAGTATTT
GCTATAAAGAAAAAGGACAGTACCAAATGGAGGAAATTAGTAGACTTCAGAGAACTCAAT
AAAAGAACTCAGGACTTTTGGGAAGTTCAATTAGGAATACCGCATCCAGCAGGTTTACAC
AAGAAAAAATCAGTAACAGTACTAGATGTGGGAGATGCATATTTTTCAGTTCCATTAGAT
AAAGACTTTAGAAAGTATACTGCATTCACCATACCTAGTATAAACAATGAGACACCAGGA
ATTAGATATCAGTACAATGTACTGCCACAGGGATGGAAAGGATCACCAGCAATATTCCAG
TGTAGCATGACAAAAATCTTAGAACCCTTTAGAAGrAAAAATCCAGAAATAATTATCTAT
CAATACGTGGATGACTTGTATGTAGCATCTGATTTAGAAATAGGGCAGCACAGAGCAAAA
ATAGAGGAGCTAAGAGCTCATCTATTGAGCTGGGGATTTACTACACCAGACAAAAAGCAT
CAGAAGGAACCTCCATTCCTTTGGATGGGATATGAACTCCATCCTGACAGATGGACAGTC
CAGCCTATAGAACTGCCAGAAAArGAYAGCTGGACTGTCAATGATATACAGAAATTAGTG
GGAAAACTAAATTGGGCAAGCCAGATCTATCCAGGGATTAGArTAAAGCAACTGTGTAAA
CTCCTCAGGGGAGCTAAAGCACTAACAGACATAGTACCACTrACTGAAGAAGCAGAG

>27
CCTCAAATCACTCTTTGGCAACGACCCCTTGTCACAATAAAAATAGCAGGACAGCTGAGA
GAAGCTCTATTAGATACAGGAGCAGATGATACAGTATTAGAAGATATAAATTTGCCAGGA
AAATGGAAGCCAAAAATGATAGGGGGAATTGGAGGTTTTATCAAGGTCAGGCAATATGAT
CAGATACTTATAGAAATTTGTGGAAAAAAGGCTATAGGTACAGTGTTAGTAGGACCTACA
CCTGTCAACATAATTGGACGAAATATGTTGACTCAGCTTGGTTGTACTCTAAATTTCCCA

ATTAGTCCTATTGACACTGTACCAGTAAAATTAAAGCCAGGAATGGATGGGCCAAAGGTT
AAACAATGGCCATTGACAGAAGAAAAAATAAAAGCATTAACAGAAATTTGTAAAGAAATG
GAAGAGGAAGGAAAAATCTCAArAATTGGGCCTGAAAATCCATACAATwCTCCAGTATTT
GyTATAAAGAAAAAAGACAGCACCAAATGGAGGAAATTAGTrGACTTCAGAGAGCTCAAT
AAAAGAACTCrGGACTTTTGGGAAGTTCAGTTAGGAATACCGCATCCAGCAGGTTTAAAA
AAGAAAAAATCAGCAACAGTACTAGATGTAGGAGATGCATATTTTTCAGTTCCTTTAGAT
GAAAGCTTTAGAAAGTATACTGCATTCACCATACCTAGTAGAAACAATGAGACACCAGGA
ATTAGATATCAGTACAATGTGCTTCCACAGGGATGGAAAGGATCACCAGCAATATTCCAA
AGTAGCATGACAAAAATCTTAGAGCCTTTTAGAAAACAAAATCCAGACATAGAGATCTAT
CAATACGTGGATGATTTGTATGTAGGATCTGACTTAGAAATAGGGCAGCATAGAACAAAA
ATAGAGGAGCTGAGACAACATTTATTGAAGTGGGGACTTACCACACCAGACAAGAAACAT
CAGAAAGAACCCCCGyTCCGTTGGATGGGTTATGAACTCCATCCTGATAAATGGACAGTA
CAGCCTATAGyGCTGCCAGATAAGGATAGCTGGACTGTCAATGACATACAAAAGTTAGTG
GGAAAATTAAATTGGGCAAGTCAAATTTATGCAGGGATCAAGGTGAAGCAACTGTGCAAG
CTCCTCAGGGGAGCTAAGGCGCTAACAGACATAGTGCCACTAACTGAAGAAGCAGAG

>28
CCTCAGATCACTCTTTGGCAGCGACCCCTTGTCACAATAArAGTAGGGGGCCAAATAAAA
GAGGCTCTCTTAGACACAGGAGCAGATGATACAGTATTAGAGGAAATAAATTTGCCAGGA
AAATGGAAACCAAAAATGATAGGGGGAATTGGAGGTTTTATCAAAGTAAGACAATATGAT
CAMATACCTATAGAAATTTGTGGGAAAAAAGCTATAGGTACAGTATTAGTGGGACCTACA
CCTGTCAACATAATTGGAAGAAATCTGTTGACTCAGCTTGGGTGCACTTTAAATTTTCCA
ATCAGTCCCATTGAAACTGTACCAGTAAAATTAAAGCCAGGAATGGATGGCCCAAAGGTT
AAACAATGGCCATTGACAGAAGAAAAAATAAAAGCATTAACAGmAATTTGTGAGGAAATG
GArAArGAAGGAAAAATTACAAAAATTGGGCCTGAAAATCCATATAACACTCCAATATTT
GCCATAAAAAAGAAGGACAGTACTAAGTGGAGAAAGTTAGTAGATTTCAGGGAACTCAAy
AAAAGGACTCAGGATTTTTGGGAAGTTCAATTAGGAATACCACACCCAGCAGGGTTGAAA
AAGAAAAAATCAGTGACAGTACTrGATGTGGsrGATGCATATTTTTCAGTTCCTTTAyAT
GAAGAmTTCAGGAAGTATACTGCATTCACCATACCTAGTACAAACAATGArACACCAGGA
ATTAGGTATCAATATAATGTGCTTCCACAGGGATGGAAAGGATCACCAGCAATATTCCAA
TsTAGCATGAyAAAAATCTTAGAGCCTTTTAGAAATCAAAATCCAGACATAGTyATCTAT
CAATATATGGATGACTTATATGTAGGATCwGACTTAGArATAGGGCAACAyAGAGCAAAA
ATAGArGAGTTAAGrGAACATCTGTTAAAGTGGGGATTTACCACACCAGACAAAAAACAT
CAGAAAGAACCTCCATTTCTTTGGATGGGrTATGAACTCCATCCTGACAAATGGACAGTA
CAGCCTATACAGCTGCCAGAAAAGGATAGCTGGACTGTAAATGACATACAAAAGTTAGTG
GGAAAATTAAAyTGGGCAAGTCAGATTTACCCAGGAATTAAArTAArGCAACTTTGTAAA
CTCATTAGGGGAGCCAAAGCACTAACAGACATAGTACmACTAACTGrAGAAGCAGAG

>29
CCTCAAATCACTCTTTGGCAACGACCCCTTGTyACAATAAAGATAGGGGGACArTTAAAG
GAAGCTCTATTAGATACAGGAGCAGATGATACAGTATTAGAAGAyATGAATTTGCCAGGA
AGATGGAAACCAAAAATGATAGGGGGAATTGGAGGTTTTATCAAAGTAAGACAGTATGAG
AATATACCCATAGAAATCTGCGGACACAAGGCTGTAGGwACAGTATTAATAGGACCTACA
CCTGTCAACATAATTGGAAGAAATCTGTTGACTCAGCTTGGTTGyACTTTAAATTTTCCC
ATTAGTCCTATTGAAACAGTACCAGTAAAATTAAAGCCAGGAATGGATGGCCCAAAAGTT
AAACAATGGCCATTGACAGAAGAAAAAATAAAAGCATTAGTAGAAATTTGTACAGAAATG
GAAAAGGAAGGGAAAATTTCAAAAATyGGGCCTGAAAATCCATAyAATACTCCAGTATTT
GCCATAAAGAArAAAGACAGyACTAAATGGAGAAAATTAGTAGATTTCAGGGAACTTAAT
AAAAGAACTCAAGACTTCTGGGAAGTTCAATTAGGAATACCACATCCCGCAGGGTTAAAA
AAGAACAAATCCGTAACAGTCCTGGATGTGGGwGATGCATATTTCTCAGTyCCTTTAGAT
AAAGACTTCAGGAAGTATACTGCATTTACCATACCTAGTGTAAAyAATGAGACACCAGGG
ATCAGATATCAGTACAATGTGCTTCCACAGGGATGGAAAGGATCACCAGCmATATTCCAG
TGTAGCATGACAAAAATCTTAGAGCCTTTTAGAAAACAAAATCCAGACATAGTTATCTAT
CAATACATGGATGATTTGTATGTAGGATCTGACTTAGArATAGGGCAGCATAGAGCAAAG
ATAGAGGAAyTGAGACAACATCTGTTGArGTGGGGATTTACCACACCAGACAAAAARCAT
CAGAAAGAACCTCCATTCCTTTGGATGGGTTATGAACTCCATCCTGATAAATGGACAGTA
CAGCCTATAGTGCTGCCAGAAAARGACAGCTGGACTGTCAATGACATACArAAGTTAGTG
GGAAAGTTAAAyTGGGCAAGTCAGATTTATGCAGGRATTAAGGTAARGGAATTATGTARA
CTCATTAGGGGAAyCAAAGCACTAACAGAAGTAGTACCACTAACAGAAGAAGCAGAG

>30
CCTCAAATCACTCTTTGGCAACGACCCCTCGTCACAATAArGATAGGGGGGCAAkTAAAG
GAAGCTCTATTAGATACAGGAGCAGATGATACAGTATTAGAAGACATGAATTTGCCAGGA
AGATGGAAACCAAAAATGATAGGGGGAATTGGAGGTTTTATCAAAGTAAGACAGTATGAT
CAGATACCCATAGAAATCTGCGGrCACAAGGyTGTAGGTACmGTATTAwTAGGACCTACA
CCTGTCAACATAATTGGAAGAAATCTGTTGACTCAGCTTGGTTGCACCTTAAATTTTCCC
ATTAGTCCyATTGAAACTGTACCAGTAAAATTAAAGCCAGGAATGGATGGCCCAAAAGTT
AArCAATGGCCATTGACAGAAGAAAAAATAAAAGCATTAGTAGAAATTTGTACAGAAATG
GAAAAGGAAGGrAAAATTTCAAAAATyGGGCCTGAAAATCCATACAATACTCCAGTATTT
GCCATAAAGAAAAArGACAGTACTAAATGGAGAAAATTAGTAGATTTCAGGGAACTyAAT
AAAAGAACTCAAGACTTCTGGGAAGTTCAATTAGGAATACCACATCCCGCAGGGTTAAAA
AAGAAAAAATCCGTAACAGTCCTGGATGTGGGwGATGCATATTTCTCAGTCCCTTTAGAT
AAAGACTTCAGGAAGTATACkGCATTTACCATACCTAGTGTAAACAATGAGACACCAGGG
ATCAGATATCAGTACAATGTGCTTCCACAGGGATGGAAAGGATCACCAGCAATATTCCAA
TGTAGCATGACAArAATCTTAGAGCCTTTTAGAAAACAAAATCCAGACATAGTTATCTAT
CAATACATGGATGATTTGTATGTAGGATCTGACTTAGAAATAGGGCAGCATAGAGCAAAA

ATAGAGGAACTGAGACAACATCTGTTrrGGTGGGGATTTACCACACCAGACAAAAAACAT
CAGAAAGAACCTCCATTCCTTTGGATGGGTTATGAACTCCATCCTGATAAATGGACAGTA
CAGCCTATAGTGCTGCCAGAAAAGGACAGCTGGACTGTCAATGACATACAGAAGTTAGTG
GGAAAGTTGAATTGGGCAAGTCAGATTTATGCAGGGATTAAGGTwAGGGAATTATGTAAA
CTCCTTAGGGGAACCAAAGCACTAACAGAAGTAATACCACTAACAGAAGAAGCAGAG

>31
CCTCAAATCACTCTTTGGCAACGACCCCTCGTCACAATAAAGATAGGrGGGCAATTAAAG
GAAGCTCTATTAGATACAGGAGCAGATGATACAGTATTAGAAGACATGAATTTGCCAGGA
AGATGGAGACCAAAAATGATAGGGGGAATTGGAGGTTTTATCAAAGTAAAACAGTATGAT
CAGATACCCATAGAAATCTGTGGACACAAGGCTGTAGGTACAGTATTAATAGGACCyACA
CCTrTCAACATAATTGGGAGAAAyYTGTTGACTCAACTTGGTTGyACTTTAAATTTTCCT
ATTAGTCCTATTGAAACTGTACCAGTAAAATTAAAGCCAGGAATGGATGGCCCAAAAGTT
AAACAATGGCCATTGACAGAAGAAAAAATAAAAGCmTTAGTAGArATTTGTACAGAAATG
GAAAAGGAAGGrAAAATTTCAAAAATTGGGCCTGAAAAyCCATAyAATACTCCAGTATTT
GCCATAAAGAAAAAAGACAGTACTAAATGGAGAAAATTAGTAGATTTCAGGGAACTTAAT
AArAGAACTCAAGACTTCTGGGAAGTyCAATTAGGrATACCACATCCyGCAGGGTTAAAA
AAGAAAAAATCTGTAACAGTCCTGGATGTGGGTGATGCATAyTTCTCAGTyCCTTTAGAT
AAAGAmTTCAGGAAGTAyACTGCATTTACCATACCTAGTGTAAACAATGAGACACCAGGG
ATCAGATATCAGTACAATGTrCTTCCACAGGGATGGAAAGGATCwCCAGCAATATTCCAA
TGTAGTATGACAAAAATCTTAGrGCCTTTTAGArwACAAAATCCAGACATrrTyATCTAT
CAATACATGGATGATTTGTATGTAGGATCTGACTTAGAAATAGGGCAGCATAGAGCAAAA
ATAGAGGAACTGAGAGAACATCTGTTGAGGTGGGGATTTACCACACCAGACAAAAAACAT
CAGAAAGAACCTCCATTCCTyTGGATGGGTTATGAACTCCATCCTGATAAATGGACAGTA
CAGCCTATAGTGCTGCCAGAAAAGGACAGCTGGACTGTCAATGACATACAGAAGTTAGTG
GGAAAGTTGAATTGGGCAAGTCAAATTTATsCAGGGATyAAArTAAGGGAATTATGTAAA
CTCATTAGGGGAACCAAAGCAyTrACAGAAGTAATACCACTAACAGAAGAAGCAGAG

>33
CCTCAAATCACTCTTTGGCAACGACCCCTCGTCACAATAAAGATAGGGGGGCAATTAArG
GAAGCTCTATTAGATACAGGAGCAGATGATACAGTATTAGAAGACATGAATTTGCCAGGA
AGATGGAAACCAAAAATGATAGGGGGAATTGGAGGTTTTATCAAAGTAAAACAGTATGAT
CAAATAyCCATAGAAATCTGCGGACACAAGGTTGAAGGTACAGTGTTAATAGGACCTACG
CCTGTCAACATAATTGGrAGAAATCTGTTGACTCAGCTTGGTTGCACTTTAAATTTTCCT
ATTAGTCCTATTGAAACTGTACCAGTAAAATTAAAGCCAGGAATGGATGGCCCAAAAGTT
AAACAATGGCCATTGACAGAAGAAAAAATAAAAGCATTAGTAGAAATTTGTACAGAAATG

GAAAAGGAAGGGAAAATTTCAAAAATTGGACCTGAAAATCCATACAATACTCCAGTATTT
GCCATAAAGAAAAAGGACAGTACTAGGTGGAGAAAATTAGTAGATTTCAGGGAACTTAAT
AAACGAACTCAAGACTTCTGGGAAGTTCAATTAGGAATACCACATCCTGCAGGGTTAAAA
AAGAACAAATCyGTAACAATCCTGGAyGTGGGTGATGCATATTTCTCAGTCCCTTTAGAT
AAAGACTTCAGGAAGTATACTGCATTTACCATACCTAGCATAAACAATGAGACACCAGGG
ATCAGATATCAGTACAATGTGCTTCCACAGGGATGGAAAGGCTCACCArCAATATTCCAA
AGTAGCATGACAAAAATCTTAGAGCCTTTTAGAAAACAAAATCCAGATATAGTGATCTGT
CAATACGTGGATGATTTGTATGTAGGATCTGACTTAGAAATAGGACAGCATAGAGCAAAA
ATAAAGGAACTGArAAATCATCTGTTAAGGTGGGGATTTACCACACCAGACAAAAAACAT
CAGAAAGAACCTCCATTCCGTTGGATGGGTTATGAACTCCATCCTGATAAATGGACAGTA
CAGCCTATAGTGCTGCCAGAAAAGGACAGCTGGACTGTCAATGATATACAGAAGCTAGTG
GGAAAGTTAAATTGGGCAAGTCAGATTTATGCAGGGATTAAAGTAAGGGAATTATGTAAA
CTCCTyAGGGGAACCAAAGCACTAACAGAAGTAATAyCACTAACAAAAGAAGCAGAG

>34
CCTCAAATCACTCTTTGGCAACGACCCmTCGTCACArTAArGATAGGGGGGCAATTAAAG
GAAGCyCTATTAGATACAGGAGCAGATGATACAGTATTAGAAGACATGAATTTGCCAGGA
ArATGGAAACCAAAAATGATAGGGGGAATTGGAGGTTTTATCAAAGTAAGACAGTATGAT
CAGrTrTCCwTrGAAATCTGTGGACAyAArGyTGTAGGTACAGTATTAATAGGACCTACA
CCTGTCAACATAATTGGAAGrAATCTGTTGACTCArATTGGTTGCACTTTAAATTTTCCC
ATTAGTCCTATTGAAACTGTACCAGTAAAATTAAAGCCAGGAATGGATGGCCCAAAAGTT
AAACAATGGCCATTrACAGAAGAAAAAATAAAAGCATTAGTAGAAATTTGTACAGAAATG
GAAAAGGAAGGrAAAATTTCAAAAATyGGGCCTGAAAAyCCATACAATACTCCAGTrTTT
GCCATAAAGAAAAAAGAyAGTACAAAATGGAGAAAATTAGTAGATTTCAGGGAACTTAAT
AAAAGAACTCAAGACTTCTGGGAAGTTCAATTAGGAATACCACATCCyGCAGGGTTAAAA
AAGAAAAAATCArTAACAGTCCTGGATGTGGGTGATGCATATTTCTCAGTTCCTTTAGAT
GAAGATTTCAGGAAATATACTGCATTTACCATACCTAGTGTAAACAATGAGACwCCAGGG
ATCAGrTATCArTACAATGTGCTTCCACAGGGATGGAAAGGATCACCAGCAATATTCCAA
TGTAGCATGACAAAAATCTTAGAGCCTTTTAGAAAACAAAATCCAGAyATAGTTATCTAT
CAATACATGGATGATTTGTATGTAGGATCTGAyTTAGAAATAGrACAGCATAGAGCAAAA
ATAGAGGAACTGAGACAACATTTGTTrGGGTGGGGvTTTACCACACCAGACAAAAAACAT
CAGAAAGAACCyCCATTTCTTTGGATGGGTTATGAACTCCATCCTGATAAATGGACAGTA
CAGCCTATAGTGCTrCCAGAAAAGGACAGCTGGACTGTCAATGAyATACAGAAGTTAGTG
GGAAARTTrAATTGGGCAAGTCArATTTATCCAGGGATTARAGTAArrGAATTATGTAAA
CTCmTTAGGGGAACCAAAGCAyTAACAGAAGTAATACCACTAACAGAAGAAGCAGAG

>35
CCTCAGATCACTCTTTGGCAACGACCCCTCGTCACAATAAAGATAGGGGGGCAATTAAAG
GAAGCTCTATTAGATACAGGAGCAGATGATACAGTATTAGAAGACATGAATTTGCCAGGr
AGATGGAAACCAAAAATGATAGGGGGAATTGGAGGTTTTATCAAAGTAAGACAGTATGAT
CAARTASyyrTAGAAATCTGTGGCCACAAGGCTGTAGGTACAGTATTAATAGGACCTACA
CCTGTCAACATAATTGGrAGrAATyTGTTGACTCAGATTGGKTGCACTTTAAATTTTCCC
ATTAGTCCTATTGAAACTGTACCwGTAAAGTTAAAGCCAGGAATGGATGGCCCAArAGTT
AAACAATGGCCACTGACAGAAGAAAArATAAAAGCATTAGTAGAAATTTGTACAGAAATG
GAAAAAGAGGGGAAAATTTCAAAAATyGGGCCTGAAAATCCATACAATACTCCAGTATTT
GCCATAAAGAAAAAAGACAGTACTAAATGGAGAAAATTAGTAGATTTCAGGGAACTTAAT
AAAAGAACACAGGACTTCTGGGAAGTTCAATTAGGAATACCACATCCyGCAGGGTTrAAA
CAGAAAAAATCAGTAACAGTCCTGGATGTGGGTGATGCATATTTyTCAGTCCCTyTAGAT
AAGGACTTCAGGAAGTATACTGCATTTACCATACCTAGTGTAAACAATGAGACACCAGGG
ATyAGATATCAGTACAATGTGCTTCCACAGGGATGGAAAGGATCACCAGCAATATTCCAA
TGTAGCATGACAAAAATCTTAGAGyCTTTTAGAAAACAAAATCCAGACATAGTTATCTAT
CAATACATGGATGATTTGTATGTAGGATCTGACTTAGAAATAGGGmAACATAGAGAAAAA
ATAGAGGAACTGAGACAACATTTGyTGAGGTGGGGATTTACCACACCAGACAAAAAACAT
CAGAAAGAACCTCCATTCCTTTGGATGGGTTATGAACTCCATCCTGATAAATGGACAGTA
CAGCCTATAGTGyTGCCAGAAAAGGACMrCTGGACTGTCAATGACATACARAAGTTAGTG
GGAAAGTTGAATTGGGCAAGTCAGATTTATGCAGGAATTAAGRTAAAGGAATTATGTAAA
CTCATTAGGGGAACCAAAGCACTAACAGAAGTAATACCACTAACAGAAGAAGCAGAG

>36
CCTCAAATCACTCTTTGGCAACGACCCCTCGTyACAATAAArRTAGGGGGGCAATTAAAG
GAAGCTCTATTAGATACAGGAGCAGATGATACAGTATTAGAAGACATGAATTTGCCAGGr
ArrTGGArACCAArAATGATAGGGGGAATTGGAGGTTTTATCAAAGTAArACAGTATGAT
mAbrTAmCCATAGAAATCTrYGGACACAAGGTTGTAGGTACAGTATTAATAGGACCyACm
CCTGTCAACATAATTGGrAGAAATyTGTTGACTCAGCTTGGTTGCACCTTAAATTTTCCC
ATTAGTCCTATTGAAACTGTACCAGTAAAATTAAAGCCAGGAATGGATGGCCCAAAAGTT
AAACAATGGCCATTGACAGAAGAAAAAATAAAAGCATTArTAGAAATTTGTACAGAaATG
GAAAAGGAAGGrAARATTTCAAAAATTGGGCCTGAAAATCCATAyAATACTCCAGTATTT
GCmATAAAGAAAAAAGACAGTACTAAATGGAGAAAATTAGTAgATTTCAGGGAACTTAAT
AAAAGAACTCAAGACTTCTGGGAAGTTCAATTAGGAATACCACATCCTGCAGGATTAAAA
AAGAAAAAATCwGTAACAGTCCTGGATGTGGGTGATGCATAYTTCTCAGTYCCTTTAGAT
rAAGAmTTCAGGAAGTAyACTGCATTTACCATACCTAGTGTAAACAATGAGACACCAGGG
ATTAGATATCAGTACAATGTGCTTCCACAGGGATGGAAAGGMTCACCAGCAATATTCCAA
TGTAGCATGACAAAAATCTTAGAGCCTTTTAGAArAMAmAATCCAGAMATrGTTATCTAT
CAATACATGGATGATTTGTATGTAGGATCTGACTTAGAAATAGGGCAGCATAGAGCAAAA
ATAGAGGAACTAAGAGACCATCTGTTGAAGTGGGGrTTTACCACACCAGACAAAAAACAT
CAGAAAGAACCyCCATTCCTTTGGATGGGTTATGAACTCCATCCTGATAArTGGACAGTA

CAGCCTrTAGTGCTGCCAGAAAAGGACAGCTGGACTGTCAATGACATACAGAAGTTAGTG
GGAAAGTTGAATTGGGCAAGTCAGATTTATCCAGGGATTAAGGTAAAGGAATTATGTAAA
CTCCTTAGrGGAACCAAAGCACTAACAGAAGTArTACCACTAACAGAAGAAGCAGAG

>37
CCTCAAATCACTCTTTGGCAACGACCCCTyGTCACArTAAGGATAGGGGGGCAATTAAAG
GAAGCTCTATTAGATACAGGAGCAGATGATACAGTATTAGAAGACATGAATTTGCCAGGr
ArATGGAAACCAAAAATGATAGGGGGAATTGGAGGTTTTATCAAAGTAAGACAGTATGAT
CAGATACCCATAGAAATCTGTGGACACAAGACTGTAGGTACAGTATTAATAGGACCTACA
CCTGTCAACATAATTGGAAGAAATCTGTTGACTCAGCTTGGTTGCACTTTAAATTTTCCT
ATTAGTCCTATTGAAACTGTACCAGTAAAATTAAAGCCAGGAATGGATGGCCCAAAAGTT
AArCAATGGCCATTrACAGAAGAAAAAATAAAAGCATTAGTAGAAATTTGTACAGAAATG
GAAAAGGAAGGrAAAATTTCAAAAATyGGGCCTGAAAATCCATACAATACTCCAGTATTT
GCCATAAAGAAAAAAGACAGTACTAArTGGAGAAAATTAGTAGATTTCAGGGArCTTAAT
AAAAGAACTCAAGACTTCTGGGAAGTTCArTTAGGAATACCACATCCyGCrGGryTAAAA
AAGAAAAAATCCGTAACAGTyCTGGATGTGGGTGATGCATATTTCTCAGTCCCTTTAGAT
AAAGACTTCAGrAAGTATACTGCATTTACCATACCTAGTsTAAAyAATGAGACACCAGGG
mTCAGATATCAGTACAATGTGCTTCCACARGGATGGAAAGGATCACCAGCAATATTyCAA
TrTAGCATGACAAAAATCTTAGAGCCTTTTAGAAAACAAAATCCAGACATAATTATCTAT
CAATACATGGATGATTTGTATGTAGGATCAGACTTAGAAATAGGGCAGCATAGAGCAAAA
ATAGArGAACTGAGACAACATyTGTTGAGGTGGGGrTTyACCACACCAGACAAAAAACAT
CAGAAAGAACCTCCATTCCTTTGGATGGGkTATGAACTCCATCCTGATAAATGGACAGTA
CAGCCTATAGTGCTGCCAGAAAAGGACAGCTGGACTGTCAATGACATACAGAAGTTAGTG
GGAAAGTTrAATTGGGCAAGTCAGATTTATGCAGGGATTAAGGTAAGrGAATTATGTAAG
CTCCTTAGGGGAACCAAAGCACTAACAGAAGTAATACCACTAACAGAAGAAGCAGAG

>38
CCTCAAATCACTCTTTGGCAACGACCCCTCGTCACAATAAAGATAGGGGGGCAATTAAAG
GAAGCTCTATTAGATACAGGAGCAGATGATACAGTATTAGAAGACATGAATTTGCCAGGA
AAATGGAAACCAAAAATGATAGGGGGAATTGGAGGTTTTATCAAAGTAAGACAGTATGAT
CAGGTACCCATAGAAATCTGyGGACACAAGGCAGTAGGTACAGTrTTAATAGGACCTACA
CCTGTCAACATAATTGGAAGAAATCTGTTGACTCAGCTTGGTTGCACTTTAAATTTTCCy
ATTAGTCCTATTGAAACTGTACCAGTAAAATTAAAGCCAGGAATGGATGGCCCAAArGTT
AAACAATGGCCATTGACAGAAGAAAAAATAAAAGCATTAGTAGAGATTTGTACAGAAATG
GArAAGGAAGGrAAAATTTCAAAAATTGGGCCTGAAAATCCATACAATACTCCAGTATTT
GCCATAAAGAAAAArGACAGTACTAAATGGAGAAAATTAGTAGATTTCAGGGAACTTAAT
AAAAGAACTCAAGACTTCTGGGAAGTTCAATTAGGAATACCACAyCCCGCAGGATTAAAA

AAGAAAAAAATCTGTAACAGTCCTGGATGTGGGTGATGCATATTTCTCAGTTCCTTTAGAT
AAAGAATTCAGGAAGTATACTGCATTTACCATACCTAGTGTAAACAATGAGACACCAGGG
ATyAGATATCAGTACAATGTGCTTCCACAGGGATGGAAAGGATCACCAGCAATATTyCAA
TGTAGCATGACAAAAATCTTAGAGCCCTTTAGAAArCAAAATCCAGAyATrGTwATCTAT
CAATACATGGATGATTTGTATGTAGGATCTGACTTAGAAATAGGGCAGCATAGAGAAAAA
ATAGAGGAACTGAGACAACATCTGTTGArrTGGGGrTTTACCACACCAGAyAAAAAACAT
CAGAAAGAACCTCCATTCCTwTGGATGGGTTATGAACTCCATCCTGATAAGTGGACAGTA
CAGCCTATAGTGCTGCCAGAAAAGGACAGCTGGACTGTCAATGACATACAGAAGTTAGTG
GGAAArTTAAATTGGGCAAGTCAGATTTATGCAGGGATTAAGATAAGGGAATTATGCAAA
CTCATTAGGGGAACCAAAGCACTAACAGAAATAGTACCACTAACAGAAGAAGCAGAG

>39
CCTCAAATCACTCTTTGGCAACGACCTGTTGTCAYAGTrAAAATAGrAGrrCwrYTGAAA
GAAGCTCTGTTAGATACAGGAGCAGATGATACAGTATTAGAGGATATAAATTTGCCAGGG
AAATGGAAGCCAAAAATGATAGGGGGAATTGGAGGTTTTATCAAAGTAArACArTATGAT
CARRTAyyTATAGAAATTTGTGGAAAAAAGGCTrTAGGTACAGTrTTAGTAGGACCTACA
CCTGTCAACATAATTGGACGAAATATGTTGACTCAGATTGGyTGTACTTTAAATTTyCCr
ATCAGThCTATTGAnACTrTACCAGTAACATTAAAGCCAGGAATGGATGGACCAArGGTT
AAACArTGGCCATTGACAGAAGAAAAAATAAAAGCATTAACAGAAATTTGTAAAGAGATG
GAAGAGGAAGGAAAAATTTCAAAAATTGGGCCTGAAAAyCCATACAATACTCCAATATTT
GCTATAAAGAAAAAGGATGGCACCAAATGGAGAAAATTAGTAGATTTCAGrGAGCTCAAT
AAAAGAACTCAGGATTTTTGGGAAGTTCAATTAGGAATACCACATCCAGCAGGTTTAAAA
AAGAAAAAATCArTAACAGTACTTGATGTGGGAGATGCATATTTTTCArTTCCATTAGAT
GAAAACTTTAGAAAGTATACTGCATTCACCATACCTAGTATAAACAATGAGACACCAGGA
ATCAGATATCAGTACAATGTGCTGCCACAGGGATGGAAAGGATCACCAGCAATATTCCAG
AGTAGCATGACAAAAATCTTAGAACCmTTTAGAGCAAAAAATCCAGAAATAATTATCTAT
CAATACATGGATGACTTGTATGTAGGATCTGATyTAGAAATAGGGCAGCATAGAATGAAA
ATAGArGAGCTrAGAGCTCATCTATTrAAATGGGGATTTACyACACCAGACAAAAAGCAT
CAGAAAGAACCyCCATTCCTTTGGATGGGATATGAACTCCATCCTGACAGATGGACAGTC
CAGCCTATAGAATTGCCAGAAAAGGACAGCTGGACTGTCAATGATATACAGAAATTAGTG
GGAAAACTAAATTGGGCAAGTCAAATTTATCCAGGGATTAAAGTAAAGCAATTGTGyAAA
CTCCTCAGrGGAGCTAAAGCACTAACAGACATAGTACCACTGACTGCAGAAGCAGAG

>40
CCTCAAATCACTCTTTGGCAACGACCCCTTGTCACAGTAAAAATAGGAGGACAGATrAAA
GAAGCTCTATTAGATACAGGGGCAGATGATACAGTATTAGAAGATATAAATTTACCAGGA

AAATGGArACCAAAAATGATAGGGGGAATTGGAGGTTTTATCAAGGTAAAACAATATGAT
CAGATACCTATAGAAATTTGTGGAAAAAAGGCTATAGGTACAGTATTAGTAGGACCTACA
CCTGTCAACATAATTGGACGAAATATGTTGACTCAGATTGGTTGTACTTTAAATTTyCCA
ATTAGTCCTATTGACACTATACCAGTAGCATTAAAGCCAGGAATGGATGGACCAAAAGTT
AAACAGTGGCCATTAACAGAAGAAAAAATAAArGCATTAACAGAAATTTGTAAAGAGATG
GAAGAGGAAGGAAAAATTTCAAAAATTGGGCCTGAAAATCCATACAATACTCCGGTATTT
GCTATAAAGAAAAAGGACAGCACCAAATGGAGAAAATTAGTAGATTTCAGAGAGCTCAAT
AAAAGrACTCAGGACTTTTGGGAAGTTCAATTAGGAATACCGCATCCAGCGGGCTTAAAG
AAAAAGAAATCAGTAACAGTACTAGATGTGGGAGATGCATATTTTTCAGTTCCTTTAGAT
GAAArCTTTAGAAAGTATACTGCATTTACAATACCTAGTACAAACAATGAGACACCAGGA
ATCAGATATCAGTACAATGTGCTGCCACAAGGATGGAAAGGATCACCGGCAATATTCCAG
AGTAGCATGACAAAGATCTTAGAGCCCTTTAGAATAAAAAATCCAGAGATGGTTATCTAT
CAATACATGGATGACTTGTATGTAGGCTCTGATTTAGAAATAGGGCAACACAGAATAAAA
rTAGAGGAGCTGAGAGCTCATCTATTGAGCTGGGGATTTACTACCCCAGACAAAAAGCAT
CAGAAGGAACCyCCATTCCTTTGGATGGGATATGAACTCCATCCTGACAAATGGACAGTC
CAGCCTATAGAACTGCCAGAAAAGGACAGCTGGACTGTCAATGATATACAGAAATTAGTG
GGAAAACTCAATTGGGCAAGTCAAATTTATGCAGGAATTAAGGTAAAGCAACTATGTAGA
CTCCTCAGGGGAACTAAAGCACTAACAGACATAGTGCCACTGACTGAAGAAGCAGAG

>41
CCTCAAATCACTCTTTGGCAACGACCCCTTGTCACAATAArAATAGGAGGACAGCTAAAA
GAAGCTCTATTAGATACAGGAGCAGATGATACAGTATTAGAAGATATAAATTTGCCAGGA
AGATGGAAACCAAAAATGATAGGGGGAATTGGAGGwTTTATCAAAGTAAGGCAATATGAT
CAGATACCTATAGAAATTTGTGGAAAAAGGGCTATAGGTACAGTATTAGTAGGACCTACA
CCTGTCAACATAATTGGACGAAATATGTTGACTCAGCTTGGyTGTACTTTAAATTTCCCA
ATTAGTCCTATTGATACTGTACCAGTAACATTAAAGCCAGGAATGGATGGACCAAAGGTT
AAACAGTGGCCATTGACAGAAGAAAAAATAAAAGCATTAACAGAAATTTGTAAAGAGATG
GAAGAGGAAGGAAAAATCTCAAAAATTGGGCCTGAAAATCCATATAATACTCCTGTATTT
GCTATAAAGAAAAAGGACAGCACCAAATGGAGGAAATTAGTAGATTTCAGAGAGCTCAAT
AAAAGAACTCAGGACTTTTGGGAAGTTCAATTAGGAATACCGCATCCAGCAGGATTAAAA
AGGAAAAAATCAGTGACAGTACTAGATGTGGGAGATGCATATTTTTCAGTTCCTTTAGAT
GAAAGCTTTAGAAAGTATACTGCATTCACCATACCTAGTATAAACAATGAGACACCAGGA
ATCAGATATCAGTACAATGTACTACCACAGGGATGGAAAGGATCTCCAGCTATATTCCAG
TGTAGCATGACAAAAATCTTAGAGCCCTTTAGAAGAAAAAATCCAGACATGGTTATCTAT
CAATATATGGATGACTTGTATGTAGGATCTGATTTAGAAATAGGGCAGCACAGAACAAAA
ATAGAGGAGCTAAGAGATCATCTATTGAGCTGGGGATTTACTACACCAGACAAAAAGCAT
CAGAAGGAGCCTCCATTTCTATGGATGGGATATGAACTCCATCCAGACAGATGGACAGTC
CAGCCTATAGAACTACCAGAAAAAGACAGCTGGACTGTCAATGATATACAGAAATTAGTG
GGAAAACTAAATTGGGCAAGTCAAATTTATGCAGGGATTAAGGTAAAGCAACTGTGTAAR
CTCCTCAGGGGAACTAAAGCACTAACAGAAATAGTACCACTAACTGAAGAAGCAGAG

>42
CCTCAAATCACTCTTTGGCAACGACCCCTTGTCACAGTAARAATAGGAGGACAATTGAAG
GAAGCTCTATTAGATACAGGAGCAGATGATACAGTATTAGAAGATATArATTTGCCAGGr
AAATGGAAACCAAGAATGATAGGGGGrATTGGAGGTTTTATCAAGGTAAAGCAATATGAA
CAGATACCTATAGAAATTTGTGGAAAAAAGGCTATAGGTACAGTRTTAGTAGGACCTACA
CCTGTCAACATAATTGGACGAAATATGTTGACTCAGGTTGGTTGTACTTTAAATTTCCCA
ATTAGTCCTATTGACACTGTACCAGTAAAATTAAAGCCAGGAATGGATGGACCAAAGGTT
AAACAGTGGCCATTGACAGAAGAAAAAATAAAAGCATTAACrGAAATTTGTAAAGAAATG
GAAGAGGAAGGAAAAATCTCAAAAATTGGGCCTGAAAATCCATACAATACyCCAGTATTT
GCTATAAAGAAAAAGGACAGTACCAAATGGAGRAAATTAGTAGATTTCAGAGAGCTTAAT
AAAAGAACTCAGGAyTTCTGGGAAGTTCAATTAGGAATACCGCATCCAGCAGGTTTAAAA
AAGAAAAAATCAGTAACAGTACTrGATGTGGGAGATGCATATTTCTCAGTTCCATTGGAT
AAAGACTTTAGAAAGTATACTGCATTCACCATACCTAGTwyAAACAATGAGACACCAGGA
ATCAGATATCAGTATAATGTGyTGCCACAGGGATGGAArGGATCACCAGCAATATTCCAG
AGTAGCATGACAAAAATCTTAGAGCCCTTTAGAATAAAAAATCCAGAAGTAATTATCTAT
CAATACATGGATGACTTGTATGTAGGATCAGATTTAGAAATAGGACAGCATAGAACAAAA
ATAGAGGAGCTAAGAGCTCATCTATTGAGCTGGGGATTmACTACACCAGACAAAAAGCAy
CAGAAGGAACCTCCATTCCTkTGGATGGGATATGAACTCCATCCTGACArATGGACAGTC
CAGCCTATAGAACTGCCAGAAAAAGACAGCTGGACTGTCAATGATATACAAAAATTAGTG
GGAAAACTAAATTGGGCAAGCCAGATTTATGCAGGrATTAArATAAAGCAACTGTGTAAA
CTCCTCAGGGGAGCTAAAGCATTAACAGAyATAGTrCCAyTGACTACAGAAGCAGAG

>43
CCTCAGATCACTCTTTGGCAACGACCCCTTGTCGCAATAAGArTrGGGGGACArAyAAAA
GAGGCCCTCTTAGACACAGGAGCAGATGATACAGTATTAGAAGAAATAAATTTGCCAGGC
AAGTGGAAACCAAAAATGATAGGAGGAATTGGAGGTTTTATCAAAGTAAGACAATATGAT
CAAATAGCTrTAGAAATTTGTGGAAAAAAGGCTATAGGTACAGTATTAGTGGGACCCACr
CCTrTyAACATAATTGGAAGAAATCTGTTGACTCAGATTGGATGCACACTAAATTTTCCG
ATCAGTCCCATTGAAACTGTACCAGTAAAATTAAAGCCAGGAATGGATGGCCCAAAAGTy
AAACAATGGCCATTGACAGAAGAAAAAATAAAAGCATTAGTAGAAATTTGTACAGAAATG
GAAAAGGAAGGAAArATTTCAAAAATyGGACCTGAAAATCCATACAATACCCCAGTATTT
GCCATAAAGAAAAArGACAGTACTAAATGGAGAAAATTAGTAGATTTCAGGGAACTTAAT
AAAAGAACTCAAGACTTyTGGGAAGTTCAATTAGGAATACCACATCCkGCAGGGTTAAAA
AAGAAAAAATCTGTAACAGTCCTGGATGTGGGTGATGCATATTTTTCAGTTCCTTTAGAT
AAAGACTTTAGGAArTATACTGCATTTACCATACCTAGTGTAAACAATGAGACACCAGGA

ATTAGATACCAGTACAATGTGCTTCCACAAGGATGGAAAGGATCACCAGCAATATTTCAA
TGTAGCATGACAAAAATCTTAGAACCTTTTAGAAAACAAAATCCAGAyATAGwTATCTAT
CAATACATGGATGATTTGTACGTAGGATCTGATTTAGAAATAGrACAGCATAGAACAAAA
ATAGAGGAACTGAGrCAACATCTGTTGAGGTGGGGATTTACCACACCAGACAAAAAACAT
CAGAAAGAACCTCCATTCCAATGGATGGGTTATGAACTCCATCCTGATAAATGGACAGTA
CAGCCTATAATGCTGCCAGAGAAAGACAGCTGGACTGTCAATGACATACAGAAGTTAGTG
GGAAAGTTAAATTGGGCAAGTCAGATCTATCCAGGGATTArGrTAAAGGAATTATGTARA
CTCATTAGGGGAACTAAArCATTAACAGAAGTAATACCACTAACAAAAGAAGCAGAG

>44
CCTCAAATCACTCTTTGGCAACGACCCCTTGTTACCATAAAGATAGGGGGGCAATyAAAG
GAAGCTCTATTAGATACAGGAGCAGATGATACAGTATTAGAAGACATrAATTTGCCAGGG
AAATGGAAACCAAAAATGATAGGGGGAATTGGAGGTTTTATCAAAGTAAGACAGTATGAm
CAGATACCCATAGAAATCTGTGGACATAAAGCTGTAGGTACAGTATTAATAGGACCTACA
CCTGTCAACATAATTGGAAGAAATCTGTTGACTCAGCTTGGTTGTACTTTAAATTTTCCA
ATCAGTCCTATTGAAACTGTACCAGTAAAACTAAAGCCAGGAATGGATGGCCCAAAGGTT
AAACAATGGCCATTGACAAAAGAGAAAATAGAAGCATTAACAGCAATTTGTGAGGAAATG
GAAAAGGAAGGAAAAATTACAAAAATTGGGCCTGAAAATCCATACAACACTCCAATATTT
GCCATAAAAAAGAAAGACAGTACTAAGTGGAGAAAATTAGTAGATTTTAGGGAACTCAAT
AAAAGAACTCAAGATTTTTGGGAAGTTCAATTAGGAATACCACACCCAGCAGGATTAAAA
AAGAAAAAATCAGTGACAGTGCTGGATGTGGGGGATGCATATTTTTCAGTTCCTTTATAT
GAAGACTTCAGGAAATATACTGCATTCACCATACCTAGTACAAACAATGAAACACCAGGG
ATTAGGTATCAGTACAATGTACTTCCACAGGGGTGGAAAGGATCACCAGCAATATTTCAA
AGTAGCATGACAAAAATCTTAGAGCCTTTCAGAAAACAAAATCCAGACATAGTCATCTAT
CAATACATGGATGATCTGTATGTAGGATCTGACTTAGAGATAGGGCAGCATAGAACAAAA
ATAGAGGAACTGAGACAACATTTATTGAGGTGGGGATTyACCACACCAGACAAGAAACAT
CAGAAAGAACCTCCATTTCTTTGGATGGGGTATGAACTCCATCCTGACAAATGGACAGTA
CAGCCTATACAGCTGCCAGTACAAGATAGCTGGACTGTCAATGATATACAAAAGTTAGTG
GGAAAACTAAACTGGGCAAGTCAGATCTATCCTGGAATTAAAGTAAGGCAACTGTGTAAA
CTTCTTAGGGGGGCCAAAGCACTAACAGACGTAGTACCACTAACTGAAGAAGCAGAG

>45
CCTCAAATCACTCTTTGGCAACGACCCCTCGTTACAATAAAGATAGGGGGGCAATTAAAG
GAAGCTCTATTAGATACAGGAGCAGATGATACAGTATTAGAAGAAATGAATTTGCCAGGA
AGATGGAAACCAAAAATGATAGGAGGAATTGGAGGTTTTATCAAAGTAAGACAGTATGAT
CAGGTATCCATAGAAATCTGTGGACACAAGGCTGTGGGTACAGTATTAATAGGACCTACA

CCCGTCAACATAATTGGGAGAAATCTGTTGACTCAGCTTGGTTGTACTTTAAATTTTCCT
ATTAGTCCTATTGAAACTGTACCAGTAAAATTAAAGCCAGGAATGGATGGGCCAAAAGTT
AAACAATGGCCATTAACAGAAGAAAAAATAAAAGCATTAGTAGAAATTTGTACAGAAATG
GAAAAGGAAGGGAAAATTTCAAAAATTGGGCCTGAAAATCCATACAATwCTCCAGTATTT
GCCATAAAGAAAAAAGACAGTACTAAATGGAGAAAATTAGTAGATTTCAGGGAACTTAAT
AAAAGAACTCAAGACTTCTGGGAAGTGCAATTAGGAATACCACATCCCGCAGGGTTAAAA
AAGAAAAAATCTGCAACAGTCCTGGATGTGGGTGATGCATACTTCTCAGTCCCTTTAGAT
AAAGACTTCAGGAAGTATACTGCATTTACCATACCTAGTGTAAACAATGAGACACCAGGG
ATCAGATATCAGTACAATGTGCTTCCACAGGGATGGAAAGGATCACCAGCAATATTCCAA
TGTAGCATGACAAAAATCTTAGATCCTTTTAGAAAACAAAATCCAGACATAGTTATCTAT
CAATACGTGGATGATTTGTATGTAGGATCTGACTTAGAAATAGGGCAGCATAGAGCAAAA
GTAGAGGAACTAAGACAACATTTGTTGGGGTGGGGATTTACCACACCAGACAAAAAACAC
CAGAAAGAGCCTCCACTCCTTTGGATGGGTTATGAACTCCATCCTGATAAATGGACAGTA
CAGCCTATAGTGCTGCCAGAAAAGGACAGCTGGACTGTCAATGACATACAGAAGTTAGTG
GGAAAGTTAAATTGGGCAAGTCAGATTTATGCAGGAATTAAGGTAAGGGAATTATGTAAA
CTCCTTAGGGGAGCCAAAGCACTAACAGAAGTAATACCACTAACAGAAGAAGCAGAG

>46
CCTCAAATCACTCTTTGGCAACGACCCATTGTCACAGTAAAAGTAGAAGGACAGCTGAAA
GAAGCTCTATTGGATACAGGAGCAGATGATACAGTTTTAGAAGATATAAATTTGCCAGGA
AAATGGAAACCAAAAATGATAGGGGGrATwGGAGGTTTTATCAArGTAAGGCAATATGAT
CAGATATCTATAGArATTTGTGGrAAAAAGGCyATAGGTACAGTATTAGTAGGACCTACA
CCTGTCAACATAATTGGrCGAAATATGTTGACTCAGGTTGGTTGyACTTTAAATTTCCCA
ATTAGTCCTATTGATACTGTACCAGTArCATTAAAGCCAGGAATGGATGGACCAAAAGTT
AAACAGTGGCCATTGACAGAAGAAAAAATAAAAGCATTAACAGAGATTTGTAAAGAGATG
GAAGAGGAAGGAAAAATCTCAAAAATTGGGCCTGAAAATCCATACAATACTCCAATATTT
GCTATAAAGAAAAAGGAyGGCACCAAATGGAGAAAATTAGTAGACTTTAGAGAGCTCAAT
AAAAGAACTCAGGACTTTTGGGAAGTTCAATTAGGAATACCACATCCAGCAGGTTTAAAA
AAGAAmAAATCAGTAACAGTACTAGATGTGGGAGATGCATATTTTTCAGTTCCTTTACAT
GAAGACTTTAGAAARTATACTGCATTCACCATACCTAGTATAAACAATGAGACACCAGGA
ATCAGATATCAGTACAATGTGCTGCCACAAGGATGGAAAGGATCACCrGCAATATTyCAA
AGTAGCATGACAAAAATCTTAGAGCCCTTTAGAATAAAAAATCCAGAAATAGTGATCTAT
CAATACATGGATGACTTGTATGTAGGATCTGATTTAGAAATAGAGCAGCACAGAACAAAA
ATAGAGGAGCTAAGAGCTCATCTATTGAGCTGGGGATTTACTACACCAGACAAAAArCAT
CAGAAGGAACCCCCATTCCTTTGGATGGGATATGAACTCCATCCTGACAAATGGACAGTC
CAGCCTATAGAACTGCCAGAAAGAGACAGCTGGACTGTTAATGATATACAGAAATTAGTG
GGAAAGCTAAATTGGGCAAGTCAGATTTATGCAGGGATTAAGATAAAGCAACTGTGTAAA
CTCATCAGGGGAGCTAAAGCACTAACAGACGTAGTACCACTGACTGAAGAAGCAGAG

>47
CCTCAAATCACTCTTTGGCAACGACCCCTTGTCACAATAAAAATAGGAGGACAGCTAAAA
GAAGCTCTGTTAGATACAGGAGCAGATGATACAGTATTAGAAGATATAAATTTGCCAGGA
AAATGGAAACCAAAAATGATAGGGGGAATTGGAGGTTTTATCAAGGTAAGGCAATATGAT
CAGATACTTATAGAAATTTGTGGAAAAAAGGCTATAGGTACAGTAyTAGTAGGACCTACA
CCTGTCAACATAATTGGACGAAATATGTTGACTCAGATTGGTTGTACTTTAAAyTTyCCA
ATTAGTCCTATTGACACTGTACCAGTAACATTAAAGCCAGGAATGGATGGACCAAAGGTT
AAACAGTGGCCATTGACAGAAGAAAAAATAAAAGCATTAACAGAAATTTGTAAAGAGATG
GAAGAGGAAGGAAAAATCTCAAAAATTGGGCCTGAAAATCCATATAATACCCCAGTATTT
GCTATAArGAAAAAGGACAGCACCAAATGGAGGAAATTAGTAGATTTCAGAGAGCTCAAT
AAAAGAACTCAGGACTTTTGGGAAGTTCAATTAGGAATACCGCATCCAGCAGGATTAAAA
AAGAAAAAATCAGTGACAGTATTAGATGTGGGAGATGCATATTTTTCAGTTCCTTTAGAT
GAAAGCTTTAGAAArTATACTGCATTCACCATACCTAGTATAAACAATGAGACACCAGGA
ATCAGATATCArTACAATGTGCTACCACAGGGATGGAAAGGATCTCCGGCAATATTCCAG
TGTAGCATGACAAAAATCTTAGAGCCCTTTAGAACAAAAAATCCAGAGATkGTTATCTAT
CAATACATGGATGACTTGTATGTAGGATCTGATTTAGAAATAGGGCAGCACAGAATAAAA
ATAGAGGAGCTAAGAGCTCATCTATTGAGCTGGGGATTTACTACACCAGACAAAAAGCAT
CAGAAGGAACCTCCATTTCTTTGGATGGGATATGAACTCCATCCGGACAGATGGACAGTC
CAGCCTATACAACTGCCAGAAAAAGACAGCTGGACTGTCAATGATATACAGAArTTAGTG
GGAAAACTAAATTGGGCAAGTCAAATTTATGCAGGGATTMrGGTAAAGCAACTGTGTAAA
CTCCTCAGGGGAGCTAAAGCACTAACrGACATAGTACCACTGACTGAAGAAGCAGAG

>48
CCTCAAATCACTCTTTGGCAACGACCCCTTGTCACAATAAAAATAGGGGGACAACTAAAA
GAAGCTCTATTAGATACAGGAGCAGATGATACAGTATTAGAAGATATAAATTTGyCAGGA
AAATGGAAACCAAAAATGATAGGGGGAATTGGAGGTTTTATCAArGTAAGGCAATATGAT
CAGATACTTATAGAAATTTGTGGAAAAAAGGCTATAGGTACAGTrTTAGTAGGACCTACA
CCGGTCAACATAATTGGmCGAAATATGTTGACTCAAATTGGTTGTACTTTAAATTTCCCC
ATTAGTCCTATTGACACTGTACCAGTAAAATTAAAGCCAGGAATGGATGGACCAAAGGTT
AAACAGTGGCCATTGACAGArGAAAAAATAAAAGCATTAACAGAAATTTGTAArGAGATG
GAAGAGGAAGGvAAAATCTCAAAAATTGGrCCTGAAAATCCCTATAATACTCCAGTATTT
GCTATAAAGAAAAAGGACAGCAsCAAATGGAGGAAATTAGTAGATTTCAGAGAGCTCAAT
AAAAGAACTCAGGACTTTTGGGAAGTTCAATTAGGAATACCkCATCCAGCAGGATTrAAA
AAGAAAAAATCAGTGACAGTACTrGATGTGGGAGATGCATATTTTTCAGTTCCTTTAGAT
GAAAGCTTTAGAAAATATACTGCATTCACCATACCTAGTATAAACAATGAGACACCAGGA
ATCAGATATCAGTACAATGTGCTRCCACAGGGATGGAAAGGATCTCCGGCAATATTCCAG
TGTAGCATGACAAAAATCTTAGAGCCCTTTAGAArAAACAATCCAGAGATGGwwATCTAT

CAATACATGGATGACTTGTATGTAGGATCTGATTTAGAAATAGGGCAGCACAGArCAAAA
ATAGAGGAGCTrAGAGCTCATCTATTGAGCTGGGGATTTACTACACCAGACAAAAAGCAT
CAGAArGAACCTCCATTTCTTTGGATGGGmTAyGAACTCCATCCGGACAAATGGACAGTC
CAGCCTATAGAACTACCAGAAAAAGACAGCTGGACTGTCAATGATATACAGAAATTAGTG
GGAAARCTAAATTGGGCAAGTCAAATTTATGSAGGrATTAAGGTAAAGCAACTGTGTAAA
CTCCTCAGGGGAGCyAAAGCACTAACAGAAGTAGTACCACTGACTGAAGAAGCAGAG

>49
CCTCAAATCACTCTTTGGCAACGACCCATTATCCCAGTAAGAATAGGAGGACAGCTAAGA
GAAGCTCTATTAGATACAGGAGCAGATGATACAGTATTAGAAGACATAGATTTACCAGGA
AAATGGAAACCAAAAATGATAGGGGGAATTGGAGGTTTTATCAAAGTAArGCAATATGAT
CAGATACCTATAGAAATTTGTGGwAAAAAGGCTATAGGAACAGTGTTAGTAGGACCTACA
CCTGTCAACATAATTGGACGAAATATGTTGACTCAGATTGGTTGTACTTTAAATTTCCCA
ATTAGTCCTATTGACACTGTACCAGTAAAATTAAAACCAGGAATGGATGGACCAAAGGTT
AAGCAGTGGCCATTGACAGAAGArAAAATAAAAGCATTAACAGAAATTTGTAAAGAGATG
GAAGAGGAAGGAAAAATTTCAAAAATTGGGCCTGAAAATCCATAyAATACTCCAGTGTTT
GCTATAAAGAAAAAAGACAGCAACAAATGGAGGAAATTAGTAGATTTTAGAGAGCTCAAT
AAGAGAACTCAAGACTTTTGGGAAGTTCAATTAGGAATACCGCATCCAGCAGGTTTAAGA
AAGAAAAAATCAGTAACAGTACTAGATGTGGGAGATGCATATTTTTCAGTTCCATTAGAT
AAAGAATTTAGAAAGTATACTGCATTCACCATACCTAGTATAAACAATGAGACACCAGGA
ATCAGATATCAATACAATGTGCTACCACAGGGATGGAAAGGATCACCAGCAATATTCCAG
AGTAGCATGACAAAAATCTTAGAGCCCTTTAGAATAAAAAATCCAGAAATAACTATCTAT
CAATACATGGATGACTTGTATGTAGGGTCTGATTTAGAAATAGGACAACATAGAACAAAA
GTAGAGGAGCTGAGAGCTCATCTATTGAGCTGGGGGTTTACTACACCAGACAAAAAGCAT
CAGAAGGAACCTCCATTyCTTTGGATGGGATATGAACTCCATCCTGACAAATGGACAGTC
CAGCCTATAGAACTGCCAGAAAAAGACAGCTGGACTGTCAATGATATACAGAAGTTAGTG
GGAAAACTAAATTGGGCAAGTCAAATTTATGCAGGAATTAAGGTAAGGCAACTGTGTAAA
CTCCTCAGGGGAACTAAAGCACTAACAGACATAGTACCACTAACTGAAGAAGCAGAG

>50
CCTCAAATCACTCTTTGGCAACGACCCATTGTCCCAGTAAGAATAGGAGGGCAGCTAAAA
GAAGCTCTATTAGATACAGGAGCAGATGATACAGTATTAGAAGACATAGATTTACCAGGA
AAATGGAAACCAAAAATGATAGGGGGAATTGGAGGTTTTATCAAAGTAAAGCAATATGAT
CAGATACCTATAGAAATTTGGGGAAAAAAGGCTATAGGAACAGTGCTAGTAGGACCTACA
CCTGTCAACATAATTGGACGAAATATGTTGACTCAGATTGGTTGTACTTTAAATTTCCCA
ATTAGTCCTATTGACACTGTACCAGTAAAATTAAAACCAGGAATGGATGGACCAAAGGTT

AAGCAGTGGCCATTGACAGAAGAAAAAATAAAAGCATTAACAGAAATTTGTAAAGAGATG
GAAGAGGAAGGAAAAATCTCAAAAATTGGGCCTGAAAATCCATATAATACCCCAGTGTTT
GCTATAAAGAAAAAAGACAGCACCAAATGGAGGAAATTAGTAGATTTCAGAGAGCTCAAT
AAAAGAACTCAAGACTTTTGGGAAGTTCAATTAGGAATACCACATCCAGCAGGTTTAAGA
AAGAAAAAATCAGTAACAGTACTAGATGTGGGAGATGCATACTTTTCAGTTCCATTAGAT
AAAGAATTTAGAAAGTATACTGCATTCACCATACCTAGTATAAACAATGAGACACCAGGA
ATCAGATATCAATACAATGTGCTGCCACAGGGATGGAAAGGATCACCAGCAATATTCCAG
AGTAGCATGACAAAAATCTTAGAGCCCTTTAGAATAAAGAATCCAGAAATAACTATCTAT
CAATACGTGGATGACTTGTATGTAGCGTCTGATTTAGAAATAGGACAACATAGAACAAAA
GTAGAGGAGCTAAGAGCTCATCTATTGAGCTGGGGATTTACTACACCAGACAAAAAGCAT
CAGAAAGAACCTCCATTCCTTTGGATGGGATATGAACTCCATCCTGACAAATGGACAGTC
CAGCCTATAGAACTGCCAGAAAAAGACAGCTGGACTGTCAATGATATACAGAAATTAGTG
GGAAAATTAAATTGGGCAAGTCAAATTTATGCAGGGATTAAGATAAAGCAACTGTGTAAA
CTCATTAGGGGAACTAAAGCACTAACAGAAATAGTACCACTAACTGAAGAAGCAGAG

>51
CCTCAAATCACTCTTTGGCAACGACCCCTTGTCACAGTAAAAATAGAAGGACAATTrAAA
GAAGCTCTRTTAGATACAGGAGCAGATGATACAGTATTAGAAGATATAAATTTGCCAGGA
AArTGGAAACCAAAAATGATAGGGGGAATTGGAGGTTTTATCAArGTAAGGCAATATGAT
CAGATACATATAGAAATTTGTGGAAAAAGrGCTATAGGTACAGTrTTAGTAGGACCTACA
CCTGTCAACATAATTGGACGAAATATGTTGACTCAGATTGGTTGTACTTTAAATTTCCCA
ATAAGTCCTATTGACACTGTACCrGTAAAATTAAAGCCAGGAATGGATGGACCAAAGGTT
AAACAGTGGCCATTGACAGAAGAAAArATAAAAGCATTAACAGAAATTTGTAArGArATG
GAAsAGGAAGGAAAAATCTCAAAAATTGGRCCTGAAAATCCATACAATACTCCAATATTT
GCTATAAAGAAAAAGGACGGyACCAAATGGAGAAAATTAGTAGATTTCAGAGAGCTCAAT
AAAAGAACTCAGGACTTTTGGGAAGTTCAATTAGGAATACCACATCCAGCAGGTyTAAAA
AAGAAwAAATCAGTAACyGTAyTrGATGTGGGAGATGCATATTTTTCAGTTCCTCTAGAT
GAAAGCTTTAGAAAGTATACTGCATTCACCATMCCTAGTACAAACAATGAGACACCAGGA
ATCAGATATCAGTACAATGTGCTACCACAGGGATGGAAAGGATCACCAGCAATATTCCAA
AGTAGCATGAyAAAAATCTTAGAGCCCTTTAGAGCAAAAAATCCAGAAmTAACTATCTAT
CAATACATGGATGACTTATATGTAGsATCTGATTTAGAAATAGGACAACATAGAACAAAA
ATArArGAGTTAAGArAACATCTrTTACAATGGGGATTTACCACACCAGACAAGAAACAT
CAGAAAGAACCTCCATTTCTTTGGATGGGGTATGAACTCCAyCCTGACAAATGGACAGTA
CAGCCTATACArCTGCCAGAAAAAGATAGCTGGACTGTCAATGATATACAGAAGTTAGTG
GGTAAATTAAACTGGGCAAGTCAGATTTACCCAGGAATTArAATAAAGCAACTTTGTAAA
CTCmTwAGGGGGGCCAAGGCACTAACAGACATAGTACCACTGACTGArGAAGCAGAG

>52
CCTCAAATCACTCTTTGGCAACGACCCCTCGTCACAATAAAGATAGGGGGGCAAACAAAG
GAAGCTCTATTAGATACAGGAGCAGATGATACAGTATTAGAAGACATGAATTTGCCAGGG
AGGTGGAAACCAAAAATGATAGGGGGAATTGGAGGTTTTATCAAAGTAAGACAGTATGAT
CAGATACCyATAGAAATCTGTGGGCACAAGACTGTAGGTACAGTATTAATAGGACCTACA
CCTGTCAACATAATTGGAAGAAATCTGTTGACTCAAATTGGTTGCACTTTAAATTTTCCC
ATTAGTCCTATTGAAACTGTACCAGTAAAATTAAAGCCAGGGATGGATGGCCCAAAAGTT
AAACAATGGCCATTGACAGAAGAAAAAATAAAAGCATTAGTAGAAATTTGTACAGAAATG
GAAAAAGAAGGrAAAATTTCAAAAATyGGGCCTGAAAATCCATACAATACTCCAGTATTT
GTCATAAAGAGAAAACACGGTACTAAATGGAGAAAATTAACAGATTTCAGrGAACTTAAT
AAAAGAACTCAAGACTTTTGGGAAGTTCAATTAGGAATACCACATCCAGCAGGGTTAGAA
AAGAAAAAATCTGTAACAGTCCTGGATGTGGGTGATGCATATTTCTCGGTCCCTyTAGAT
AAAGACTTCAGGAAGTATACTGCATTTACCATACCyAGTATAAACAATGAGACACCAGGG
ATCAGATATCAGTACAATGTGCTTCCACAGGGATGGAAGGGATCACCAGCAATATTTCAG
AGTAGCATGACAAAAATCTTAGAGCCTTTTAGAAAACAAAATCCAGACATAGTTATCTGT
CAATATATGGATGATTTGTATGTAAGCTCTGACTTAGAAATAGGGCAGCATAGAGCAAAA
ATAGAGGAACTGAGAAATCATCTGTTGAAGTGGGGATTTACCACTCCAGACGAAAAATAT
CAGAAAGAACCTCCATTTCTTTGGATGGGTTATGAACTCCATCCTGATAAATGGACAGTA
CAGCCTATACAGCTGCCAGAAAAGGACAGCTGGACTGTCAATGACATACAGAArTTAGTG
GGAAAATTGAATTGGGCAAGCCAGATCTATGCAGGGATTAAGGTAAGGGAATTATGTAAA
CTCCTTAGGGGAACCAAAGCACTAACAGAAGTAGTACCrCTAACAAAGGAAGCAGAG

>53
CCTCAAATCACTCTTTGGCAACGACCCCTTGTCACAATAAArATAGGGGGGCAATTAAAG
GAAGCCCTATTAGATACAGGAGCAGATGATACAGTATTAGAAGAAATGAATTTGCCAGGA
AGATGGAAACCAAAAATGATAGGGGGAATTGGAGGTTTTGTCAAAGTAAGACAGTTTGAT
CAGGTACCCATAGAAATCTGTGGACACAAAACTGTAGGTACAGTATTAMTAGGACCTACA
CCTGCCAACATAATTGGAAGAAATTTGTTGACTCAACTTGGTTGCACTTTAAATTTTCCC
ATTAGTCCTATTGAAACTGTACCAGTAAAATTAAAGCCAGGAATGGATGGCCCAAGAGTT
AAACAATGGCCATTGACAGAAGAAAAAATAAAAGCATTAGTGGAAATTTGTACAGAAATG
GAAAAGGAAGGAAAAATTTCAAAAATAGGGCCTGAAAATCCATACAATACTCCAGTATTT
GCCATAAAGAAAAAGGACGGyACTAAATGGAGAAAATTAGTAGATTTCAGGGAACTTAAT
AAAAGAACTCAAGACTTCTGGGAAGTTCAATTAGGAATACCACATCCCGCAGGGTTAAAA
AAGAAAAAATCAATAACAGTGCTGGATGTGGGTGATGCATACTTCTCAGTCCCTTTAGAT
AAAGACTTTAGGAAGTACACTGCATTTACCATACCTAGTGTAAACAATGAGACACCAGGG
ATCAGATATCAGTACAATGTGCTTCCACAGGGATGGAAAGGATCACCAGCAATATTCCAG
TGTAGCATGACAAAAATCTTAGAGCCTTTTAGAAAACAAAATCCAGACATAGTTATCTAT
CAATATGTGGATGATTTGCTTGTAGGATCTGACTTAGAAATAGGGCAGCATAGAGCAAAA
ATAGAGGAACTGAGACAACATCTGTTGArGTGGGGAyTyACCACACCAGACAAAAArCAT

CAGAAAGAACCTCCATTCCTTTGGATGGGTCATGAACTCCATCCAGATAAATGGACAGTA
CAGCCTATCGAGCTGCCAGAAAAGGACAGCTGGACGGTCAATGACATACAGAAGTTAGTG
GGAAAGTTGAATTGGGCAAGTCAGATTTATTCAGGGATTAAGGTAAGGGAATTATGTAAA
CTTCTTAGGGGAACCAAAGCACTAACAGAAGTAGTACCACTAACAGAAGAAGCAGAG

>54
CCTCAAATCACTCTTTGGCAACGACCCATTGTCACAGTAAAAATAGGrGGACAGCTAAAA
GAAGCTCTATTAGATACAGGAGCAGATGATACAGTATTAGAAGATATAAATTTGCCAGGA
AAATGGAAACCAAAAATGATAGGAGGAATTGGAGGTTTTATCAAAGTAAGGCAATATGAT
CAGATACTTATAGAAATTTGTGGGAAAAAGGCTATAGGTACAGTGCTAGTAGGACCTACA
CCTGTCAACATAATTGGAAGAAATATGTTGACyCAAATTGGTTGyACTTTAAATTTCCCC
ATTAGTCCTATTGACACTGTACCAGTAACATTAAAGCCAGGAATGGATGGACCAAAGGTT
AAACAGTGGCCATTGACAGAAGAAAAAATTAAAAGCATTAACAGAAATTTGTAATGAGATG
GAAAAGGAAGGAAAAATTTCAAAAATTGGGCCTGAAAATCCATACAATACTCCAGTATTT
GCTATAAAGAAAAAGGACAGCACCAAATGGAGAAAATTAGTAGATTTCAGAGAGCTCAAT
AAAAGAACTCAAGACTTTTGGGAAGTTCAATTAGGAATACCGCATCCAGCAGGTTTAAGA
AAGAAAAAATCAATAACAGTACTAGATGTGGGAGATGCATATTTCTCWGTCCCTTTAGAT
GAAArCTTTAGAAAGTATACTGCATTCACCATACCTAGTATAAACAATGAGACACCAGGA
GTCAGATATCAGTACAATGTrCTGCCACAGGGATGGAAAGGrTCACCrGCAATATTCCAr
TGTAGTATGACAAAAATCCTAGAGCCCTTTAGAAGAAAAAATCCAGATATGGTTATCTGT
CAATACGTGGATGACTTGTATGTAGGATCTGATTTGGAAATAGGGCArCACAGArCAAAA
ATAGAGGAGCTrAGAGCTCATCTATTGAACTGGGGATTTACTACACCTGACAArAAGyAT
CAGAAGGAACCTCCATTyCkTTGGATGGGATATGArCTCCATCCTGACAAATGGACAGTC
CAGCCTATAGArCTGCCAGAAARAGACAGTTGGACTGTCAATGATATACARAAATTAGTG
GGAAAACTAAATTGGGCAAGTCAAATTTATGCAGGGATTAAGGTAAAGCAACTGTGTAGA
CTCCTTAGGGGGGCCAAAGCACTAACAGACATAGTACCAyTGACTGAAGAAGCAGAG

>55
CCTCAAATCACTCTTTGGCAACGACCCCTTGTCACAATAAAAATAGAAGGACAGTTrAAA
GAAGCTCTATTAGATACAGGAGCAGATGATACAGTATTAGAAGATATAAATTTGCCAGGr
AAATGGAAACCAAAAATGATAGGGGGAATTGGAGGTTTTATCAAAGTAAGACAATATGAT
CAGATACTTATAGAAATTTGTGGAAAAAAGGCTGTAGGTACAGTGTTAGTAGGACCTACA
CCTGTCAACATAATTGGACGAAATATGTTGACTCAGCTTGGTTGTACTTTAAATTTCCCA
ATTAGTCCTATTGACACTGTACCAGTGAAATTAAAGCCAGGAATGGATGGACCAAAGGTT
AAACAGTGGCCATTGACAGAGGAAAAAATAAAAGCATTAACAGAAATTTGTAAAGAGATG
GAAGAGGAAGGrAAAATCTCAAAAATTGGGCCTGAAAATCCATACAATACyCCAGTATTT
GCyATAAAGAAAAAGGACAGCwCCAAATGGAGAAAATTAGTAGAyTTCAGAGArCTCAAT

AAAAGGACTCAGGACTTTTGGGAAGTTCAATTAGGAATACCACATCCAGCAGGGCTAAAA
AAGAAAAAATCAGTAACAGTACTAGATGTGGGAGATGCATATTTyTCAGTTCCTTTAGAT
GAAAGCTTTAGAAAGTATACTGCATTCACCATACCTAGTACAAACAATGAGACACCAGGA
ATCAGATATCAGTACAATGTGCTGCCACAGGGATGGAAAGGATCACCGGCAATATTCCAr
wGTAGCATGACAArAATCTTAGAGCCCTTTAGAATAAAAAATCCAGAAATAATTATCTGT
CAATACGTGGATGACTTGTATGTAGGATCTGATTTAGAAATAGAGCAGCACAGAGCAAAG
GTAGAAGAGTTAAGAGCTCATTTATTGAGCTGGGGGTTTACGACACCAGACAAAAAGCAT
CAGAAGGAACCCCCATTCCTTTGGATGGGATATGAACTCCATCCTGACAAATGGACAGTC
CAGCCTATAGAACTGCCAGAAArAGATAGCTGGACTGTCAATGATATACAGAAATTAGTG
GGAAAACTAAATTGGGCAAGyCAAATTTATGCAGGGATTAAGATAAAGCAAyTGTGTAAA
CTCATCAGGGGAACTAAAGCACTAACAGAArTAGTACCACTAACTGArGAAGCAGAG

>56
CCTCAAATCACTCTTTGGCAACGACCCCTCGTCACAATAAAGATAGGGGGGCAATTAAAG
GAAGCTCTATTAGATACAGGAGCAGATGATACAGTATTAGAAGACATGAATTTGCCAGGA
AGATGGAAACCAAAAATGATAGGGGGAATTGGAGGTTTTATCAAAGTAAGACAGTATGAT
CAGATACCCATAGAAATCTGCGGACACAAGGCTGTAGGTACAGTATTAATAGGACCTACA
CCTGTCAACATAATTGGGAGAAATCTGTTGACTCAGCTTGGTTGCACTCTAAATTTTCCC
ATTAGTCCTATTGAAACTGTACCAGTAAAATTAAAGCCAGGAATGGATGGCCCAAAAGTT
AAACAATGGCCATTGACAGAAGAAAAAATAAAAGCATTAGTAGArATTTGTACTGAAATG
GAAAAGGAAGGGAAAATTTCAAGAATAGGGCCTGAAAATCCATACAATACTCCAGTATTT
GTCATAAAGAGAAAAGAyAGTACTAAATGGAGAAAATTAGTAGATTTCAGGGAACTTAAT
AAAAGAACTCAAGACTTTTGGGAAGTTCAATTAGGAATACCACATCCTGCAGGGTTArAA
AAGAAAAAATCCATAACAATCCTGGATGTGGGTGATGCATATTTCTCAGTCCCTTTAGAT
AAAGACTTCAGGAAGTATACTGCATTTACCATACCTAGTGTAAACAATGAGACACCAGGG
ATTAGATATCArTACAATGTGCTTCCACAGGGATGGAArGGATCACCAGCAATATTCCAG
wGTAGCATGACAAAAATCTTAGAGCCyTwTAGAAAACAAAATCCAGACATAGTTATTTGT
CAATACGTGGATGATTTGTATGTAGsATCTGATTTAGAAATAGGGCAGCATAGAGCAAAA
ATAGAGGAACTGAGACArCATCTGTTGAGGTGGGGATTTACCACACCAGACAAAAAACAT
CAGAAAGAACCyCCATTCCTkTGGATGGGTTATGAACTCCATCCkGAwAAATGGACrGTA
CAGCCTATATTrCTrCCAGAAAAGGACAGCTGGACTGTCAATGACATACAGAArTTAGTG
GGAAAATTAAATTGGGCAAGTCAGATTTATGCAGGGATTAAGGTAAGGGAATTATGTAAA
CTCCTGAGGGGAACTAAAGCGCTAACAGAAGTmATACCACTAACAGAAGAAGCAGAG

>57
CCTCAAATCACTCTTTGGCAACGACCCCTTGTCACAGTAAAAATAGGAGGACAGATAAAA

GAAGCTCTATTAGATACAGGAGCAGATGATACAGTATTAGAAGATATAAATTTGCCAGGA
AAATGGAAACCrAAAATGATAGGGGGAATTGGAGGTTTTATTAAGGTAAGGCAATATGAT
CAGATACTTATAGAAATTTGTGGAAAAAGGGCTATAGGTACAGTrTTAGTAGGACCCACG
CCTGTCAACATAATTGGACGAAATATGTTGACTCAGATTGGTTGTACTTTAAATTTCCCA
ATTAGTCCTATTGACACTGTACCAGTAACATTAAAGCCAGGAATGGATGGACCAAAAGTT
AAACAGTGGCCATTGACAGAAGAAAAAATAAAAGCATTAACAGAAATTTGTAAGGArATG
GAAGAGGAAGGAAAAATCTCAAAAATCGGGCCTGAAAATCCATACAATACTCCArTATTT
GCTATAAAGAAAAAGGACAGCACCAAATGGAGrAAATTAGTAGATTTCAGAGAGCTTAAT
AAAAGAACTCAGGATTTTTGGGAAGTTCAATTAGGAATACCrCAyCCAGCAGGyTTAAAA
AAGAAyAAATCAGTAACAGTACTAGATGTGGGAGATGCATATTTTTCAATTCCTTTAGAT
GAAAATTTTAGAAAGTATACWGCATTCACCATACCTAGTATAAATAATGAGACACCAGGA
ATCAGATATCAGTACAAyGTGCTGCCACAGGGATGGAAAGGATCACCAGCAATATTCCAG
AGTAGCATGAyAAAAATCTTAGArCCCTTTAGAAAAAGAAATCCAGAAATGGTTATTTAT
CAATACATGGATGACTTGTATGTAGGATCTGATTTAGAAATAGGGCAGCACAGAACAAAA
ATAGAGGAGyTGAGAGCTCATCTATTGAGCTGGGGACTTACTACACCAGATAArAAGCAT
CAGAAGGAACCTCCATTCCTTTGGATGGGRTATGAACTCCATCCTGACAmATGGACAGTC
CAGCCTATArAACTGCCAGAAAAAGACAGCTGGACTGTCAATGATATACAGAAATTAGTG
GGAAArCTAAATTGGGCAAGTCAAATATATGCAGGAATTAGGGTAAGrCAACTGTGTAAA
CTCCTCAGGGGAGCTAAAGCACTAACAGACATAGTACCATTGACTGAAGAAGCAGAG

>58
CCTCAAATCACTCTTTGGCAACGACCCCTTGTCACAATAAAAATAGGAGGACAGyTGAAA
GAAGCTcTATTAGATACAGGAGCAGATGATACAGTATTRGAAGATATAAATTTGCCAGGA
AAATGGAAACCAAAAATGATAGGGGGAATTGGAGGTTTTATCAAAGTAAGGCAATATGAT
CAGATAsTTATAGAAATTTGTGGAAAAAAGGCTATAGGTACAGTGTTAGTAGGACCTACA
CCTGTCAACATAATTGGACGAAATATGTTGACTCAgATTGGTTGTACTTTAAACTTTCCA
ATTAGTCCTATTGACACTGTACCAGTAACATTAAAGCCAGGAATGGATGGACCAAAGGTT
AAACAGTGGCCATTGACAGAAGAAAAAATAAAAGCATTAACAGAAATTTGTAGGGAAATG
GAAGAGGAAGGAAAAATTTCAAAAATTGGGCCTGAAAATCCATATAATACTCCAGTATTT
GCTATAAAGAAAAAGrACAGCACCAAATGGAGGAAATTAGTAGATTTCAGAGAGCTCAAT
AAAAGAACTCAGGACTTTTGGGAAGTTCAATTAGGGATACCGCATCCAGCAGGATTAAAA
AAGArAAAATCAATGACAGTACTAGATGTGGGAGATGCATwTTTCTCAGTCCCTTTAGAT
GAAAGCTTTAGAAAGTATACTGCATTCACCATACCTAGTATAAACAATGAGACACCAGGA
ATCAGATATCAGTACAATGTGCTACCACAGGGATGGAAAGGATCTCCGGCAATATyTCAG
TGTAGCATGACAAAAATCTTAGAGCCCTTTAGAAAACAAAATCCAGAGATAGATATCTAT
CAATACGTGGATGACTTGTATGTAGGATCTGATTTAGAAATAGGGCAGCACAGAGCAAAA
ATAGATGAGCTAAGAGCTCATCTATTGAGCTGGGGATTTACTACACCAGACrAAAAGCAT
CAGAAGGAACCGCCATTkCTTTGGATGGGATATGAACTCCATCCGGACAGATGGACAGTC
CAGCCTATAGAACTGCCAGAAAAAGACAGCTGGACTGTCAATGATATACAGAAATTAGTG
GGAAAACTAAATTGGGCAAGTCAAATATATGCAGGGATTAAGATAAAGCAACTGTGTAAA

CTCCTCAGGGGAGCTAAAGCATTAACAGATGTAGTACCACTGACTGAAGAAGCAGAG

>59
CCTCAAATCACTCTTTGGCAACGACCCmTTGTCACAGTAAAAATAGGAGGACArCTGAAA
GAAGCTCTTTTAGATACAGGAGCAGATGATACAGTATTAGAAGATATAAATTTGCCAGGA
AAATGGAAACCAAAAATGATAGGGGGAATTGGAGGTTTTATCAArGTAArACAATATGAT
CAGATACTTATAGAAATTTGTGGAAAAAAGGCTATAGGTACAGTATTAGTAGGACCTACA
CCTGTCAACATAATTGGACGAAATATGTTGACTCAGATTGGTTGTACTTTAAATTTCCCA
ATTAGTCCTATTGAAACTGTACCAGTAACATTAAAGCCAGGrATGGATGGrCCAAAGGTT
AArCAGTGGCCATTGACAGAAGAAAAAATAAAAGCATTAACAGAAATTTGTAAAGArATG
GAAGAGGAAGGAAArATCTCAAAAATTGGrCCTGAAAATCCATACAATACCCCAGTATTC
GCTATAAAGAAAAAAGACAGCACCAAATGGAGGAAGCTAGTAGATTTCAGAGAGCTCAAT
AAAAGAACTCAGGAyTTTTGGGAAGTTCAATTAGGAATACCACACCCAGCAGGTTTAAAA
AAGAAAAAATCArTAACAGTACTAGATGTGGGAGATGCATATTTTTCAGTTCCTTTAGAT
GAAAGCTTTAGAAAGTAyACTGCATTCACCATACCTAGTATAAACAATGAGACACCAGGA
ATCAGATATCAGTACAATGTGCTGCCACAGGGATGGAAAGGATCACCAGCAATATTCCAG
TGTAGCATGACAAAAATCTTAGAGCCCTTTAGAATAAAAAATCCAGAAATAAyTATCTAy
CAATATATGGATGATTTrTAyGTAGCATCTGATTTAGAAATAGGACAGCATAGArCAAAA
ATAGArGAGCTrAGAGCTCATCTATTGAGCTGGGGrTTTACTACACCAGACAAAAAGCAT
CAGAAGGAACCTCCATTCCTTTGGATGGGGTATGArCTCCATCCTGACAGATGGACAGTC
CAGCCTATAGAACTACCAGAAAAAGAYAGCTGGACTGTCAATGATATACAGAAATTAGTG
GGAAAACTAAATTGGGCAAGTCAAATTTATSCAGGGATTAAGrTAAAACAATTGTGTAAA
CTCCTCAGGGGAACTAAAGCACTAACAGACATAGTGCCACTGACTGAGGAAGCAGAG

>60
CCTCAAATCACTCTTTGGCAACGACCCCTCGTCACAATAAGGATAGGGGGGCAATTAAAG
GAAGCTCTATTAGATACAGGAGCAGATGATACAGTGTTAGAAGAAATGAATTTGCCAGGA
AGATGGAAACCAAAAATGATAGGGGGAATTGGAGGTTTTATCAAAGTAAGACAGTATGAT
CAGGTACCCATAGAAATTTGTGGACACAAGGCTGTAGGTACAGTATTAATAGGACCCACA
CCTGTCAACATAATTGGGAGAAATCTGTTGACTCAGCTTGGTTGCACTTTAAATTTTCCT
ATTAGTCCTATTGAAACTGTACCAGTAAAATTAAAGCCAGGAATGGATGGCCCAAGAGTT
AAACAATGGCCATTAACAGAAGAAAAAATAAAAGCATTAGTAGAAATTTGTACAGAAATG
GAAAAGGAAGGGAAAATTTCAAAAATAGGGCCTGAAAACCCATACAATACTCCAGTATTT
GCAATAAAGAAAAAAGACAGTACTAAATGGAGAAAATTAGTAGATTTCAGGGAACTTAAT
AAAAGAACTCAAGACTTCTGGGAAGTCCAATTAGGAATACCACATCCAGCAGGGCTAAAA
AAGAGCAAATCAGTAACAGTCCTGGATGTGGGTGATGCATATTTCTCAGTCCCTTTAGAT

GAAGACTTCAGGAAGTATACTGCATTTACCATACCTAGTGTAAACAATGAGACACCAGGG
ATCAGATATCAGTACAATGTGCTTCCACAGGGGTGGAAAGGATCACCAGCAATATTCCAA
TGTAGCATGACAAAAATCTTAGAGCCTTTTAGAAAACACAATCCAGACATAGTTATCTAT
CAGTACGTGGATGATTTGTATGTAAGCTCTGACTTAGAAATAGGGCAGCATAGAGCAAAA
ATAGAAGAGCTGAGACAACATCTGTTGGGGTGGGGATTTACCACACCAGACAAAAAACAT
CAGAAAGAACCTCCATTCCTTTGGATGGGGTATGAACTCCATCCTGATAAATGGACAGTC
CAGCCTATAGTGCTGCCAGAAAAGGACAGCTGGACTGTCAATGACATACAGAAGTTAGTG
GGAAAATTGAATTGGGCAAGTCAAATTTATGCAGGGATTAAGGTAAGGGAATTATGCAAA
CTCATTAGGGGAACCAAAGCACTAACAGAAGTAATACCACTCACAGAAGAAGCAGAG

>61
CCTCAAATCACTCTTTGGCAACGACCCCTCGTCACAATAAAGATAGGGGGGCAATTAAAG
GAAGCTCTATTAGATACAGGAGCAGATGATACAGTATTAGAAGACATGAATTTGCCAGGA
AGATGGAAACCAAAAATGATAGGGGGGATTGGAGGTTTTATCAAAGTAAGACAGTATGAT
CAGATACCCATAGAAATCTGCGGACACAArGCTGTAGGAACAGTATTAATAGGACCTACA
CCTATCAACATAATTGGAAGAAATCTGTTGACTCAGCTTGGGTGCACTTTAAATTTTCCC
ATTAGTCCTATTGAAACTGTACCGGTAAAATTAAGGCCAGGAATGGATGGCCCAAAAGTT
AAACAATGGCCATTGACAGAAGAAAAAATAAAAGCATTGGTAGAGATTTGTACAGAAATG
GAAAAGGAAGGGAAAATTTCAAAAATTGGACCTGAAAATCCATACAATACTCCAGTATTT
GCCATAAGGAAAAAAAACAGTACTAAGTGGAGAAAATTAGTAGATTTCAGGGAACTTAAT
AAAAGAACTCAAGACTTCTGGGAAATTCAATTAGGAATACCACATCCCGCAGGGTTAAAA
AAGAAAAAATCTGTAACAGTCCTGGATGTAGGTGATGCATATTTCTCAGTCCCTTTAGAT
AAAGACTTCAGGAAGTATACTGCATTTACCATACCTAGTGTAAACAATGAGACACCAGGG
GTCAGATATCAGTACAATGTGCTTCCACAGGGGTGGAAGGGATCACCAGCAATATTCCAA
TGTAGCATGACAAAAATCTTAGAGCCTTTTAGAAAACAAAATCCAGACATAATTATCTGT
CAATACATAGATGATTTGTATGTAGGATCTGATTTAGAAATAGGGCAGCATAGAACAAAA
ATAGAGGAACTGAGGCAACATCTGTTGAGGTGGGGATTTACCACTCCAGACAAAAAATAT
CAGGAAGAACCTCCACTCCTTTGGATGGGTTATGAACTCCATCCTGATAAATGGACAGTA
CAGCCTATAGTGCTGCCAGAAAAGGACAGCTGGACTGTCAATGACATACAGAAGTTAGTG
GGAAAGTTAAATTGGGCAAGTCAGATTTATACAGGGATTAAGGTAAGAGAATTATGTAAA
CTCCTTAGGGGAACCAAAGCACTAACAGAAGTAATACCACTAACAGAAGAAGCAGAG

>62
CCTCAAATCACTCTTTGGCAACGACCCCTTGTTGCAGTAAAAGTAGGAGGACAGATSAAA
GAAGCTCTATTAGATACAGGAGCAGATGATACAGTATTAGAAGATrTAAATTTGCCAGGA
AAATGGAAACCAAAAATGATAGGGGGAATTGGAGGTTTTATCAAGGTAAArCAATATGAT
sAGGTAmmkATAGAAATTTGTGGAAAAAAGGCTATAGGTACAGTGTTAGTAGGACCTACA

CCTATCAACATAATTGGACGAAATATGTTGACTCAGATTGGTTGTACTTTAAATTTCCCA
ATTAGTCCTATTGACmCTGTACCAGTAAmATTAAAGCCAGGAATGGATGGACCAAAAGTT
AAACArTGGCCATTAACAGAAGAAAAAATAAAAGCrTTAACAGAAATyTGTAAAGAGATG
GAAGCGGAAGGAAAAATCTCAAArATTGGGCCTGAAAATCCATACAATACTCCAGTATTT
GCTATAAAGAAAAAGGACAGCACCAAATGGAGGAAATTAGTAGATTTCAGAGAGCTCAAT
AAAAGAACTCAGGAyTTTTGGGAGGTTCAATTAGGAATACCGCATCCAGCAGGTTTAAAG
AAAAAGAAATCAGTAACAGTACTAGATGTGGGAGATGCATATTTTTCAGTTCCTTTAGAT
GAAAACTTTAGAAAGTATACTGCATTTACCATACCTAGTACAAACAATGAGACACCAGGA
ATCAGATATCAGTACAATGTGCTGCCACAGGGATGGAAAGGATCACCGGCAATATTCCAG
AGTAGCATGACAAAGATCTTAGAGCCmTTTAGAAGAAAAAATCCAGAAATAGATATCTrT
CAATACrTGGATGACTTGTATGTATGCTCTGATTTAGAAATAGGrCAGCACAGAATAAAA
GTAGAGGAGCTAAGAGCTCATCTATTGAGCTGGGGATTTACAACCCCAGACAAAAArCwT
CAGAAGGAACCTCCATTCCTTTGGATGGGATATGAACTCCATCCTGACAAATGGACAGTy
CAGCCTATAGAACTGCCAGAAAAGGACAGCTGGACTGTCAATGATATACAGAAATTAGTA
GGRAAACTyAATTGGGCAAGTCAAATTTACCCAGGAATTAArGTAAAGCAACTGTGTAGA
CTCCTyAGGGGAACTAAAGCACTAACAGACATAGTrCCACTGACTGGAGAAGCAGAG

>63
CCTCAAATCACTCTTTGGCAGCGACCCCTCGTCTCAATAAAGATAGGGGGGCAACAAAAG
GAAGCTCTATTAGATACAGGAGCAGATGATACAGTATTAGAAGAAATGCATTTACCAGGA
AAATGGAAACCAAAAATGATAGGGGGAATTGGAGGTTTTATCAAAGTAAGACAGTATGAT
CAGATACTCATAGAAATTTGTGGCyATAAAGCTATAGGTACAGTATTAATAGGACCTACA
CCTGTCAACATAATTGGAAGAAATCTGTTGACTCAGATTGGCTGCACTTTAAATTTTCCT
ATTAGTmCTGTTGAAACTGTACCAGTAAAATTAAAGCCAGGTATGGATGGCCCAAAAGTT
AAACAATGGCCAyTGACAGAAGAAAAAATAAAAGCATTAGTAGAAATTTGTACAGAAATG
GAGAAGGAAGGAAAAATTTCAAAAATAGGGCCTGAAAATCCATACAATACTCCAGTATTT
GCAATAAAGAAAAAAGACAGyACTAAATGGAGAAAATTAGTAGATTTCAGAGAACTCAAT
AAAAGAACTCAAGACTTCTGGGArGTTCAATTAGGAATACCACATCCCGGAGGGTTAAAA
AAGAAAAAATCAATAACAGTACTGGATGTGGGTGATGCATATTTTTCAGTTCCCTTATAT
GAGGACTTTAGGAAGTATACTGCATTTACCATACCYAGTACAAACAATGAGACACCAGGG
ATTAGGTATCAGTACAATGTGCTTCCACArGGATGGAArGGATCACCAGCAATATTyCAr
AGTAGCATGACAAAAATCTTAGATCCTTTTAGAAAACAAAATCCAGACATAGTGATCTGT
CAGTACATGGATGAyTTGTATGTAGGATCTGACTTAGAAATAGGGCAACATAGAACAAAA
GTAGAGGAACTGAGACAGCATCTGTTGAAGTGGGGATTAACCACACCAGACAAAAAATAT
CAGAAAGAACCTCCATTCCTTTGGATGGGTTATGAACTCCATCCTGATAAATGGACAGTA
CAGCCTATAGTGyTGCCAGAAAAGGACAGCTGGACTGTCAATGACATACAGAArTTAGTA
GGAAAACTGAATTGGGCAAGTCAAATTTATGCAGGGATTAAAGTAAAGCAATTATGTAAA
CTCCTTAGGGGAGCCAAATCACTAACAGAAGTAGTACCACTAACACATGAAGCAGAG

>64
CCTCAGATCACTCTTTGGCAGCGACCCCTTGTCTCArTaAAAGTAGGGGGCCAAATAAAA
gAGGCTCTCTTAGACACaGGAGCAGATGATACAGtAyTAGAAGAArTAAATTTGCCAGGA
AAATGGAAACCAAaAATGATAGGAGGAATTGGAGGTTTTATCAAAGTAAGACAATaTGAg
CAAATACCTATAgAAATCTGTGGAAAAAAGGCTATAGGTaCAGTATTAGTGGGACCCACa
CCTGTCAACATAaTTGGAAGAAATATGTTGACYCAGCTTGGATGCACAcTAAATTTTCCA
ATyagTCCCATTGAAACTGTACCAGTAAAATTAAAGCCAGGAATGGATGGCCCAAAGGTT
AAACAATGGCCATTrACAGAAGArAAAATAAAAGCATTAACAGCAATTTGTGATGAAATG
GArAAGGAAGGAAAAATTACAAAAATyGGGCCTGACAATCCATATAACACTCCAATATTT
GTCATAAAAAAGAAGGACAGTAATAAGTGGAGAAAATTAGTAGATTTCAGGGAACTyAAT
AAAAGAACTCAAGATTTTTGGGAAGTTCAATTAGGAATACCACACCCAGCAGGGTTAAAA
AAGAAmAAATCAGTAACAGTCCTGGATATAGGTGATGCmTATTTCTCAGTTCCTTTAGAT
rAAGACTTCAGGAAGTATACTGCATTTACCATACCTAGTrTAAACAATGAGACACCAGGr
ATTAGATATCAGTACAATGTGCTTCCACAGGGATGGAAAGGATCACCAGCAATATTyCAA
TGTACCATGACAAAAATCTTAGAGCCCTTyAGAAArCAAAATCCAGAyATAGwTATCTAT
CAATACATGGATGACTTGTATGTAGGATCTGACTTAGAAATAGGrCAGCATAGAACAAAA
ATAGAGGAACTGAGAGAACATCTGTTAAAGTGGGGATTTACCACACCAGACAAGAAACAT
CAGAAAGAACCTCCATTTCTTTGGATGGGrTATGAACTCCATCCTGATAAATGGACAGTA
CAGCCTATACAGCTGCCAGAAAAGGAyAGCTGGACTGTCAATGATATACAGAAGTTAGTG
GGAAAATTAAACTGGGCAAGTCAGATTTACCCAGGAATTAAAGTAAGGCAACTTTGTAAA
CTTmTTAGGGGGACTAAAGCACTAACAGAAATAGTACCACTAACTGAAGAAGCAGAG

>65
CCTCAAATCACTCTTTGGCAACGACCCCTCGTCACAATAAAGATAGGGGGGCArCTAAAG
GAAGCTCTATTAGACACAGGAGCAGATGATACAGTATTAGAAGAAATGArTTTGCCAGGA
AGATGGAAACCAAAAATGATAGGGGGAATTGGAGGTTTTATCAAAGTAAGACAGTATGAT
CAGrTAyCCATAGAAATTTGTGGACATAAAGCTATAGGTACAGTATTAATAGGACCyACA
CCAGTCAACATAATTGGAAGAAATCTGTTGACTCAGATTGGCTGYACTCTAAATTTTCCC
ATTAGTCCTGTTGAAACTGTACCAGTAAAATTAAAGCCAGGCATGGATGGCCCAAAAGTT
AAACAATGGCCATTAACAGAAGArAAAATAAArGCATTAGTAGAAATTTGTACAGAAATG
GAAAAGGAAGGwAAAATTTCAAAAATTGGGCCTGAAAATCCATACAATACTCCAGTATTT
GCCATAAAGAAAAAAGACAGTACTAAATGGAGAAAATTAGTAGATTTCAGAGAACTCAAT
AAGAGAACTCAAGACTTCTGGGAGGTTCAATTAGGAATACCACATCCAGCAGGGCTmAAA
AAGAAAAAATCAGTAACAGTACTGGATGTGGGTGATGCATATTTCTCAATTCCTTTAGAT
GAAGACTTCAGGAAGTATACTGCATTTACCATACCTAGCACrAACAATGAAACACCAGGG
ATTAGATATCAGTACAATGTGCTTCCACAGGGATGGAAAGGATCACCAGCAATATTCCAA
AGTAGCATGACAAGAATCTTAGAGCCTTATAGAAAACAAAATCCAGACATAGTGATCTAT

CAATACATGGATGATTTrTATGTAGGATCTGATTTAGAGATAGAGCAACATAGGACAAAA
ATAGAGGAACTrAGACAACATCTGTTGAGGTGGGGATTAACCACACCAGACAAAAAGCAT
CAGAAAGAACCTCCATTCCTTTGGATGGGTTATGAACTCCATCCTGATAAATGGACAGTA
CAGCCyATAATGCTGCCAGAAAArGACAGCTGGACTGTCAATGACATACAGAAGTTAATA
GGAAAATTGAATTGGGCAAGTCAAATCTATsCAGGGATyAAAGTAAAGCAATTATGTAAA
CTyCTTAGAGGAACCAAAGCACTAACAGAAGTAGTACCAyTAACAGAAGAAGCAGAG

>66
CCTCAAATCACTCTTTGGCAGCGACCCCTTGTCACAATAAAAATAGGAGGACAGCTGAAA
GAAGCTCTrTTAGATACAGGAGCAGATGATACAGTATTAGAAGATATAAATyTGCCAGGA
AAATGGAAACCAAAAATGATAGGGGGAATTGGAGGTTTTATCAAAGTAAGGCAATATGAT
CAGATACTTATAGAAATTTGTGGAAAAAAGGCTATAGGTACAGTGTTAGTAGGACCTACA
CCTGTCAACATAATTGGACGAAATATGTTGACTCAGrTTGGTTGTACTTTAAATTTTCCA
ATTAGTCCTATTGACACTGTACCAGTAACATTAAAGCCAGGAATGGATGGACCAAAGGTT
AAACArTGGCCATTGACAGAAGAAAAAATAAAAGCATTAACAGAAATTTGTAGGGAAATG
GAAGAGGAAGGAAAAATCTCAAAAATTGGGCCTGAAAATCCATATAATACTCCAGTATTT
GCTATAAAGAAAAAGGATAGCACCAAATGGAGAAAATTAGTAGATTTCAGAGAGCTCAAT
AAAAGAACTCAGGACTTTTGGGAAGTACAATTAGGAATACCGCATCCAGCAGGATTAAAA
AAGAAAAAATCAGTGACAGTACTAGATGTGGGAGATGCATATTTTTCAGTCCCTTTAGAT
GAAAGCTTTAGAAAGTATACTGCATTCACCATACCTAGTACAAACAATGAGACACCAGGA
ATCAGATATCAGTACAATGTGCTGCCACAGGGATGGAAAGGATCTCCGGCAATATTCCAG
TGTAGCATGACAAAAATCTTAGAGCCCTTTAGAAGAAAAAATCCAGAGATGGTTATCTAT
CAATACATGGATGACTTGTATGTAGGATCTGATTTAGAAATAGGGCAGCACAGAACAAAA
ATAGATGAGCTAAGAGCTCATCTATTGAGCTGGGGATTTACTACTCCAGACAAAAAGCAT
CAAAAAGAACCGCCATTTCTTTGGATGGGATATGAACTCCATCCGGACAGATGGACAGTC
CAGCCTATAGAACTGCCAGAAAAAGACAGCTGGACTGTCAATGATCTACAGAAATTAGTG
GGAAAACTAAATTGGGCAAGTCAAATTTATGCAGGGATTAAGGTAAAGCAACTGTGTAAA
CTCCTCAGGGGAGCTAAAGCATTAACAGAAGTCGTACCACTGACTGAAGAAGCAGAG

>67
CCTCAGATCACTCTTTGGCAACGACCCCTTGTTrCAATAAAArTAGGAGGCCAGRTAAAA
GAGGCTCTCTTAGACACAGGAGCAGATGATACAGTATTGGAAGAkATAAATTTrCCAGGA
AAATGGAAACCAAAAATGATAGGAGGAATTGGAGGTTTTATCAAAGTAAGACAATATGAG
CAAATATCTATAGAAATTTGTGGAAAAAAGGCTATAGGTACAGTATTAGTGGGACCCACA
CCTGTCAACATAATTGGAAGAAATATGTTGACCCAGCTTGGATGyACACTAAATTTTCCA
ATTAGTCCCATTGAAACTGTACCAGTAAAATTAAAGCCAGGAATGGATGGCCCAAAGGTT

AAACAATGGCCATTrACAGAAGAGAAAATAAAAGCATTAACAGCAATTTGTGATGAAATG
GAGAAGGAAGGAAAAATTACAAAAATTGGGCCTGAyAATCCATATAACACTCCArTATTT
GCCATAAAAAAGAAGGACAGTACTAArTGGAGAAAATTAGTAGAyTTTAGrGAGCTCAAT
AAAAGAACTCAAGATTTTTGGGAAATTCAATTAGGAATACCACATCCAGCAGGrTTAAAA
AAGAAyAAATCAGTAACArTCCTGGATGTGGGTGATGCATATTTCTCAGTTCCTTTAGAT
AAAGACTTCAGGAAGTATACTGCATTTACCATACCTAGTGTAAACAATGAGACACCAGGr
ATTAGATATCAGTACAATGTGCTTCCGCAGGGATGGAAAGGATCACCAGCAATATTCCAA
AGTAGCATGACAAAAATCTTAGAGCCTTTTAGAAAACAAAATCCAGACATAGTTATCTAT
CAATACGTGGATGACTTGTATGTAGGrTCTGACTTAGAAATAGGGCAGCATAGAACAAAA
ATAGAGGAACTGAGAGAACATCTGTTAAAGTGGGGATTTACCACACCAGACAAGAAACAT
CAGAAAGAACmTCCATTTCTTTGGATGGGGTATGAACTCCATCCTGACAAATGGACAGTA
CAGCCTATACAGCTACCAGAAAARGATAGCTGGACTGTCAATGATATACAGAAGTTAGTG
GGAAAATTAAACTGGGCAAGTCAGATTTACCCAGGAATyAAAGTAAGGCAACTTTGTAAA
CTCCTTAGGGGGGCCAAGGCACTAACAGACATAGTACCACTAACTGAAGAAGCAGAG

>68
CCTCAAATCACTCTTTGGCAACGACCCCTTGTCACAATAAAAATAGGGGGGCAATTAAAG
GAAGCTCTATTAGATACAGGAGCAGATGATACAGTATTAGAAGAAATGAATTTrCCAGGA
AGATGGAAACCAAAAATGATAGGGGGAATTGGAGGyTTTATCAAAGTAAGrCArTATGAT
CAGrTATCCATAGAAATCTGTGGACACAArGCTGTAGGTACAGTATTAATAGGACCTACw
CCAGTCAACATAATTGGrAGAAATCTGTTGACTCAGATTGGTTGCACTTTAAATTTTCCC
ATTAGTCCTATTGAAACTGTACCAGTAAAATTAAAGCCAGGAATGGATGGCCCAAAAGTT
AAACAATGGCCATTGACAGAAGArAAAATAAAAGCATTAGTAGAAATTTGTACAGAAATG
GAAAAGGAAGGGAAAATTTCAAAAATTGGrCCTGAAAATCCATAYAATACTCCAGTATTT
GCCATAAAGAAAAAAGACAGTACkAAATGGAGAAAATTAGTAGATTTyAGGGAACTTAAT
AAAAGAACTCAAGACTTCTGGGArGTyCAAyTAGGAATACCACATCCyGCrGGrTTAAAA
AAGAAAAAATCTGTAACAGTCCTGGATGTGGGTGATGCATATTTCTCAGTTCCTTTAGAT
rAAGAATTCAGGAAGTATACTGCATTTACCATACCTAGTGTAAACAATGAGACACCAGGG
ATyAGATAyCAGTAyAATGTGCTkCCACAGGGATGGAAAGGATCACCAGCAATATTCCAr
TGTAGCATGACAAAAATCTTAGAGCCTTTTAGAAAACAAAATCCAGACATAGTkATCTAT
CAATAyATGGATGATTTGTATGTAGGATCTGAyTTrGAAATAGGrCAGCAyAGAGmAAAA
ATAGAGGAACTGAGACAACATCTGTTGrrGTGGGGrTTTACCACACCAGAyAAAAAACAT
CAGAAAGAACCTCCATTyCTTTGGATGGGTTATGAACTCCATCCTGATAAATGGACAGTA
CAGCCTATAGTGCTGCCAGAAAAGGACAGCTGGACTGTCAATGACATACAGAAGTTrGTG
GGAAAGTTrAATTGGGCAAGTCAAATTTATGCAGGGATTAAGGTAAAGGAATTATGTAAA
CTCATTAGGGGAACCAArGCACTAACAGAAGTGGTACCACTAACAGAAGAAGCAGAG

>69
CCTCAAATCACTCTTTGGCArCGACCCyTAGTCACAATAAAAATAGGAGGACAGCTAAGA
GAAGCTCTATTAGATACAGGAGCAGATGATACAGTATTAGAAGATATAAATTTGCCAGGA
AAATGGAAGCCAAAAATGATAGGGGGAATTGGAGGTTTTATCAAAGTAAGACAATATGAT
CAGATACTTATAGAAATTTGTGGAAAAAAGGCTATAGGTACAGTGTTAGTAGGACCTACA
CCTrTCAACATAATTGGACGAAATATGTTGACTCAGCTTGGTTGTACTTTAAATTTCCCA
ATTAGTCCTATTGACACTGTACCAGTAACATTAAArCCAGGAATGGATGGACCAAAGGTT
AAACAGTGGCCTTTGACAGAAGAAAAAATAAAAGCATTAACAGAAATTTGTAAAGAGATG
GAAGAGGAAGGAAAAATCTCAAAAATTGGGCCTGAGAATCCATATAATACTCCAGTATTT
GCTATAAAGAAAAAGGACAGCACCAAATGGAGGAAATTAGTAGATTTCAGAGAGCTCAAT
AAAAGAACTCAGGATTTTTGGGAAGTTCAATTAGGGATACCGCATCCAGCAGGATTAAAA
AAGAAAAAATCAATGACAGTACTAGATGTGGGAGATGCCTATTTTTCAGTTCCTTTAGAT
GAAAGCTTTAGAAAGTATACTGCATTCACCATACCTAGTATAAACAATGAGACACCAGGA
ATTAGATATCAGTATAATGTrCTACCACAAGGATGGAAAGGATCTCCGGCAATATTCCAG
TGTAGCATGACAAAAATCTTAGAGCCCTTTAGAATAAAAAATCCAGAGATAGATATCTAT
CAATACATGGATGACTTGTATGTAGGATCTGATTTAGAAATAGGGCAGCACAGAACAAAA
ATAGAGGAGCTAAGAACTCATCTATTGAGCTGGGGATTTACTACACCAGACAAAAAACAT
CAGAAGGAACCTCCATTTCTTTGGATGGGATATGArCTCCATCCGGACAGATGGACAGTC
CAGCCTATAGAACTGCCAGAAAArGACAGCTGGACTGTCAATGATATACAGAAATTAGTG
GGAAAATTAAATTGGGCAAGTCAAATTTATGCAGGGATTAAGGTAAAGCAACTGTGTAGA
CTCCTCAGGGGAGCTAAAGCATTAACAGAAATAGTACCACTGACTGAAGAAGCAGAG

>70
CCTCAAATCACTCTTTGGCAACGACCCCTCGTCCCAATAAGGATAGGGGGGCAATTAAAr
GAAGCTCTATTAGATACAGGAGCAGATGATACAGTATTAGAAGACATGAATTTGCCAGGA
AGATGGAAACCAAAAATGATAGGGGGAATTGGAGGTTTTATCAAAGTAAGACArTATGAT
CAGATACCyATAGAAATyTGyGGACACAAGrCTGwAGGTACAGTrTTAATAGGACCTACA
CCTGTCAACATAATTGGrAGAAATCTGTTGACTCAGCTTGGTTGCACTTTAAATTTTCCC
ATTAGTCCTATTGAAACTGTACCAGTAAAATTAAAGCCAGGrATGGATGGCCCAAAAGTT
AAACArTGGCCATTGACAGAAGArAAAATAAAAGCATTAGTAGAAATyTGTACAGAAATG
GAAAAGGAAGGGAAAATTTCAAAAATTGGGCCTGAAAATCCATAyAATACTCCAGTATTT
GCCATAAAGAAAAAAGACAGTACTAAATGGAGAAAATTAGTAGAyTTyAGrGAACTTAAT
AAAAGAACTCAAGACTTCTGGGAAGTTCAATTAGGAATACCACATCChGCAGGGTTAAAA
AAGAAAAAATChGTAACAGTyyTGGATGTGGGTGATGCATAyTTCTCAGTyCCTyTAsAT
GAAGACTTCAGGAAGTATACTGCATTTACCATACCTAGTGTAAACAATGAGACACCAGGG
ATCAGATATCAGTAyAATGTGCTTCCACAGGGATGGAArGGATCACCAGCAATATTyCAA
TGTAGCATGACAAAAATyTTAGAGCCTTTTAGAAAACAAAATCCAGACATAGTkATCTAT
CAATACATGGATGATTTGTATGTAGGmTCTGACTTAGAAATAGGGCArCATAGAGCAAAA
ATAGArGAACTGAGACArCATyTGTTGAGsTGGGGATTyACmACACCAGACAAAAArCAT

CAGAAAGAACCyCCATTCCTTTGGATGGGTTATGAACTCCATCCTGATAAATGGACAGTA
CAGCCTATAGTGCTGCCAGAAAArGAmAGCTGGACTGTCAATGACATACAGAAGTTAGTG
GGAAAGTTrAATTGGGCAAGTCArATTTATGCAGGGATTAAGGTAAGGGAATTATGTAAA
CTCCTTAGGGGAACCAAAGCACTAACAGAAGTAGTACCACTAACAGAAGAAGCAGAG

>71
CCTCAGATCACTCTTTGGCAACGACCCCTTGTCACAATAAAAATAGGGGGrCAGCTrAAA
GAGGCTCTATTAGATACAGGAGCAGATGATACAGTGTTAGAAGATATAAATTTGCCAGGA
AAATGGAAACCAAAAATGATAGGGGGAATTGGAGGTTTTATyAAGGTAAGGCAATATGAT
GAGATACCTATAGAAATTTGTGGAAAAAAGGTTATAGGTACAGTATTAATAGGACCTACA
CCkGTCAACATAATTGGACGrAATATGTTGACTCAGCTTGGTTGTACTTTAAATTTCCCA
ATTAGTCCTATTGAAACTGTACCAGTAACATTAAArCCAGGAATGGATGGACCAAAGGTT
AAACAGTGGCCATTGACAGAAGArAAAATAAAAGCATTAACAGAAATTTGTAGAGAAATG
GAAGAAGAAGGAAAAATCTCAAAAATTGGGCCTGAAAATCCATACAATACTCCAATATTT
GCTATAAAGAAAAAGGACGGTACCAAATGGAGGAAATTAGTAGATTTCAGAGAGCTTAAT
AAAAGAACTCAGGATTTTTGGGAAGTYCAATTrGGAATACCrCATCCAGCAGGTTTAAAA
AAGAAAAAATCAGTAACAGTACTGGATGTGGGAGATGCATATTTTTCAGTTCCTTTAGAT
AAAAACTTCAGAAAGTATACTGCATTCACCATACCTAGTACAAACAAyGAGACACCAGGA
ATCAGATATCAGTACAATGTGCTGCCACAGGGATGGAAAGGATCACCAGCAATATTCCAG
AGTAGCATGACAAAAATCTTAGAGCCCTTTAGAACAAAGAATCCAGArATAGTTATCTAT
CAATACATGGATGACTTGTATGTAGGATCTGATTTAGAAATAGGGCAGCACAGAGCAAAA
ATAGAAGAGCTGAGAGCTCATCTATTGAGCTGGGGATTAACTACACCAGACAAAAAGCAT
CAAAAGGAACCTCCATTCCTTTGGATGGGATATGAACTCCATCCTGATAAATGGACAGTC
CAGCCTATAGAACTGCCrGAAAAAGACAGCTGGACTGTCAATGATATACAGAAATTAGTG
GGAAAATTAAATTGGGCAAGTCAAATTTATGCAGGyATTAAGGTAAArCAACTGTGTARA
CTCCTCAGGGGArCTAAAGCACTAACAGACATAGTACCACTGACTGAAGAAGCAGAG

>72
CCTCAAATCACTCTTTGGCAACGACCCCTCGTCCCAATAAGGATAGrGGGGCAATTAAAG
GAAGCTCTATTAGATACAGGAGCAGATGATACAGTATTAGAAGACATGAATTTGCCAGGr
AAATGGAAACCAAAAATGATAGGGGGAATTGGAGGTTTTATCAAAGTAAGrCAGTATGAy
CAGRTAdCCATAGAAATCTGTGGACACAAGGCTGTAGGTACAGTwTTAATAGGACCYACA
CCTrTCAACATAATTGGrAGAAATCTGTTGACTCAGCTTGGTTGyACTTTAAATTTTCCy
ATTAGTCCTATTGAAACTGTACCAGTAAAATTAAAGCCAGGAATGGATGGCCCAAAAGTT
AAACAATGGCCATTGACAGAAGAAAAAATAAAAGCATTAGTAGAAATTTGTACAGAAATG
GAAAAGGAAGGrAAAATTTCAAAAATTGGGCCTGAAAATCCATACAATACTCCAGTATTT
GCCATAAAGAAAAAAGACAGTACTAAATGGAGAAAATTAGTAGATTTCAGGGAACTTAAT

AAAAGAACTCAAGACTTCTGGGAAGTTCAATTAGGAATACCACATCCCGCAGGGTTAAAA
AAGAAAAAAATCAGTAACAGTCCTGGATGTGGGTGATGCATATTTTTCAGTCCCTTTAGAT
AAAGACTTCAGGAAGTATACTGCATTTACCATACCTAGTGTAAACAATGAGACACCAGGG
ATCAGATATCAGTACAATGTGCTTCCACAGGGATGGAAAGGATCACCAGCAATATTCCAG
TGTAGCATGACAAAAATCTTAGAGCCTTTTAGrAAACAAAATCCAGACATAGTTATCTAT
CAATACATGGATGATTTGTATGTAGGATCTGAyTTAGAAATAGGGCAGCATAGAGmAAAA
ATAGAGGAACTGAGACAACATCTGTTGAGGTGGGGATTTACCACACCAGAyAAAAAACAT
CAGAAAGAACCyCCATTCCTTTGGATGGGTTATGAACTCCATCCTGATAAATGGACAGTA
CAGCCTATAGTGCTGCCAGAAAAGGACAGCTGGACTGTCAATGACATACAGAAGTTAGTG
GGAAAGTTrAATTGGGCAAGTCAGATTTATGCAGGrATTAAGGTAArGGArTTATGTAAA
CTCCTTAGGGGAACCAAAGCACTAACAGAAGTArTACCACTAACAGAAGAAGCAGAG

>73
CCTCAAATCACTCTTTGGCAACGACCCCTTGTTACCATAAAAGTAGGAGGACAGCTGAGA
GAAGCTCTATTAGATACAGGAGCAGATGATACAGTATTAGAAGACATAAATTTGCCAGGA
AAATGGAAACCAAAAATGATAGGGGGAATTGGAGGTTTTATCAGGGTAAGGCAATATGAT
CAGATACyTATAGAAATTTGTGGAAAAAAGGCTATAGGTACAGTGTTAGTAGGACCTACA
CCTGTCAACATAATTGGACGAAATATGTTGACTCAGCTTGGTTGTACTTTAAATTTTCCA
ATCAGTCCTATTGACACTGTACCAGTAAAATTAAAACCAGGAATGGATGGACCAAAGGTT
AAACAGTGGCCATTGACAGAAGAAAAAATAAAAGCATTAACAGAAATTTGTAAAGAGATG
GAAGAGGAAGGAAAAATTTCAAAAATTGGGCCTGAAAACCCATACAATACTCCAGTATTT
GCTATAAAGAGAAAGGATGGCACCACATGGAGGAAATTAGTAGATTTCAGAGAGCTTAAT
AAAAGGACACAGGATTTTTGGGAAATTCAATTAGGAATACCGCATCCAGCAGGCTTACAA
AAGAAAAAATCAGTAACAGTACTAGATGTGGGAGATGCATATTyTTCAGTyCCATTAGAT
AAAGACTTTAGAAAGTATACTGCATTCACCATACCTAGTATAAACAATGAGACACCAGGA
ATCAGATATCAGTACAATGTGCTACCACAGGGrTGGAAAGGATCACCAGCAATATTCCAG
AGTAGCATGACAArAATCTTAGAGCCCTTTAGAATAAAAAATCCAGAATTAACTATCTGT
CAATACATGGATGACTTGTATGTATCATCyGATTTAGAAATAAAACAGCATAGwGCAAAA
ATAGAGGAGCTAAGAGCTCATCTATTGAACTGGGGATTTACTACACCAGACAAAAAGTAT
CAGAAGGAACCGCCTTTCCTTTGGATGGGATATGAACTCCATCCTGACAAATGGACAGTC
CAGCCTATACAACTGCCAGAAAAAGACAGCTGGACTGTCAATGATATACAGAAATTAGTG
GGAAAACTAAATTGGGCAAGTCAGATTTATGCAGGGATTAAGATAAAACAACTGTGTAAA
CTTATCAGGGGAACTAAAACACTAACAGAAATAGTACCACTGACTGCAGAAGCAGAG

>74
CCTCAGATCACTCTTTGGCAACGACCCCTCGTCGCAATAAAGATAGGGGGGCAATTAAAG

GAAGCTCTATTAGATACAGGAGCAGATGATACAGTATTAGAAGACATGAATTTGCCAGGA
AAATGGAAACCAAAAATGATAGGGGGAATTGGAGGTTTTATTAAAGTAAGACAGTATGAT
CAGGTACCCATAGAAATTTGCGGACACAAGGCTGTAGGTACAGTATTAATAGGACCTACA
CCTGTCAACATAATTGGAAGAAATCTGTTGACTCAGCTGGGTTGCACTTTAAATTTTCCT
ATTAGTCCTATTGAAACTGTACCAGTAAAGTTAAAGCCAGGAATGGATGGCCCAAAAGTT
AAACAATGGCCATTGACAGAAGAAAAAATAAAAGCATTAGTAGAAATTTGTACAGAAATG
GAAAAGGAAGGGAAAATTTCAAAAATCGGGCCTGAAAATCCATACAATACTCCAGTATTT
GCCATAAAGAAAAAAGACAGTACTAAATGGAGAAAATTAGTAGATTTCAGGGAACTTAAT
AAAAGAACACAAGACTTCTGGGAAGTCCAATTAGGAATACCACATCCTGCGGGGTTAAAA
AAGAACAAATCTGTAACAGTCCTGGATGTGGGTGATGCATATTTCTCCGTCCCTTTAGAT
AAAGACTTCAGGAAGTATACTGCATTTACCATACCTAGTGTAAACAATGAGACACCAGGG
ATCAGATATCAGTATAATGTGCTTCCACAGGGATGGAAAGGATCACCAGCAATATTCCAA
TGTAGCATGACAAAAATCTTAGAGCCTTTTAGAAAACAAAATCCAGACATAGTTATCTAT
CAATACATGGATGATTTGTATGTAGGATCTGACTTAGAAATAGGGCAGCATAGAGCAAAA
ATAGAGGAACTGAGGCAGCATTTGTTGAAGTGGGGATTTACCACACCAGACAAAAAACAT
CAGAAAGAACCTCCATTCCTTTGGATGGGGTATGAACTCCATCCTGATAAATGGACAGTA
CAGCCTATAGTGCTGCCAGAAAAGGACAACTGGACTGTCAATGACATACAGAAGTTAGTG
GGAAAGTTGAATTGGGCAAGTCAGATTTATGCAGGGATTAAGGTAAGGGAATTATGTAAA
CTCCTTAGGGGAACCAAAGCACTGACAGAAGTAATACCGCTAACAGAAGAAGCAGAG

>76
CCTCAAATCACTCTTTGGCAACGACCCTTAGTCACAATAAAAATAGGAGGACAGCTrARA
GAAGCTCTATTAGATACAGGAGCAGATGATACAGTATTAGAAGATATAAATTTGCCAGGA
AAATGGAAACCAAAAATGATAGGGGGAATTGGAGGTTTTATCAAAGTAAGGCAATATGAT
CAGATACyTATAGAAATTTGTGGAAAAAAGGCTATAGGTACAGTGTTAGTAGGACCTACA
CCTGTCAACATAATTGGAmGAAATATGTTGACTCAGCTTGGTTGTACTTTAAATTTTCCA
ATTAGTCCTATTGACACTGTACCAGTAACATTAAAACCAGGAATGGATGGACCAAAGGTT
AAACAGTGGCCATTGACAGAAGAAAAAATAAAAGCATTAACAGAAATTTGTAGGGArATG
GAArAGGAAGGAAAAATCTCAAAAATTGGGCCTGAAAATCCATATAATACTCCAGTATTT
GCTATAAAGAAAAAGGACAGCACCAAATGGAGGAAATTAGTAGATTTCAGAGAGCTCAAT
AAAAGAACTCAGGAyTTTTGGGAAGTTCAATTAGGAATACCGCATCCAGCAGGATTAAAA
AAGAAAAAATCAGTGACAGTACTrGATGTGGGAGATGCATATTTTTCAGTCCCyTTAGAT
GAAAGCTTTAGAAArTATACTGCATTCACCATACCTAGTATAAACAATGAAACACCAGGA
ATCAGATATCAGTACAATGTGCTACCACAGGGATGGAAAGGATCTCCGGCAATATTCCAG
TGTAGCATGACAAAAATCTTGGAGCCCTTTAGAAGCAAAAATCCAGAGATrGTGATCTAT
CAATACATGGATGAyTTGTATGTAGGATCTGATCTAGAAATAGGGCAGCACAGrAwAAAA
GTAGATGAGCTrAGAGCTCATCTATTAAGCTGGGGATTTACTACmCCAGACAAAAAGCAT
CAGAAAGAACCGCCATTTCTTTGGATGGGATATGAACTCCATCCGGACAGATGGACAGTC
CAGCCTATAGAACTGCCAGAAAARGACAGCTGGACTGTCAATGATATACAGAAATTAGTG
GGrAAACTAAATTGGGCAAGTCAAATTTATCCAGGGATTAAGGTAAAGCAACTGTGTAAA

CTCCTCAGGGGAGCTAAAGCATTAACAGACGTAGTACCTCTGACTGAAGAAGCAGAG

>77
CCTCAAATCACTCTTTGGCAACGACCCCTTGTTACAGTAAAAATAGGAGGACAGATGAAA
GAAGCTCTATTAGATACAGGGGCAGATGATACAGTATTAGAAGATATAAATTTGCCAGGA
AAGTGGArACCAAAAATGATAGGGGGAATTGGAGGTTTTATCAAGGTAAAGCAATATGAT
CAGATACTTATAGAAATTTGTGGAAAAAAGGCTATAGGTACAGTGTTAGTAGGACCTACA
CCTrTyAACATAATTGGACGAAATATGTTGACTCAGATTGGTTGTACTTTAAATTTCCCA
ATTAGTCCTATTGACACTGTACCAGTAACATTAAAGCCAGGAATGGATGGACCAAAAGTT
AAACAGTGGCCATTAACAGAAGAAAAAATAAAAGCATTAACAGAAATTTGTAAAGArATG
GAAGAGGAAGGAAAAATyTCAAAAATTGGGCCTGAAAATCCATACAATACTCCAGTATTT
GCTATAAAGAAAAAGGACAGCACCAAATGGAGrAAATTAGTAGATTTCAGAGAGCTCAAy
AAAAGAACTCAGGACTTTTGGGAAGTTCAATTAGGAATACCGCATCCAGCAGGTTTrAAG
AAAAAGAAATCAGTAACAGTACTAGATGTGGGAGATGCATATTTTTCAGTTCCTTTAGAT
GAAAGCTTTAGAAAGTATACTGCATTTACCATACCTAGTACAAAyAATGAGACACCAGGA
ATCAGATATCAGTACAATGTGCTGCCACAGGGATGGAAAGGATCACCrGCAATATTCCAG
AGTAGCATGACAAAGATCTTAGAGCCCTTTAGAATAAAAAATCCAGAArTAGTTATCTAT
CAATACATGGATGACTTGTATGTAGGCTCTGATTTAGAAATAGGGCAGCACAGAATAAAA
ATArAGGAGCTrAGAGCTCATCTATTGAGCTGGGGACTTACTACCCCAGACAAAAAGCAT
CAGAAGGAACCTCCATTCCTTTGGATGGGATATGAACTCCATCCTGACAAATGGACAGTC
CAGCCTATAGAACTGCCAGAAAAAGACAGCTGGACTGTCAATGATATACAGAAATTAGTG
GGGAAACTCAATTGGGCAAGTCAAATTTATGCAGGAATTAAGGTAAAACAACTGTGTAAA
CTCCTCAGGGGAACTAAAGCACTAACAGACATAGTGCCATTGACTGAGGAAGCAGAG

>78
CCTCAAATCACTCTTTGGCAACGACCCCTTGTCACAGTAaAAGTAGGAGGACAGCTGAAA
GAGGCTCTATTAGATACAGGAGCAGATGATACAGTATTAGAAGAGATAAATTTGCCAGGG
AAATGGAAACCAAAAATGATAGGGGGAATTGGAGGTTTTATTAArGTAAGGCAATATGAT
CAGATACyyMTAgAAATTTGTGGAAAAAAGGCTATAGGTACAGTGYTAGTAGGACCTACA
CCTGTCAACATAATTGGACGAAATATGTTGACTCAGCTTGGTTGTACTTTAAATTTCCCA
ATTAGTCCTATTGACACTGTACCAGTAAmATTAAAGCCAGGAATGGATGGACCAAAGGTT
AAACAGTGGCCATTGACAGAAGAAAArATAAAAGCATTAACAGAAATTTGTAmrGArATG
GAArAGGAAGGAAAAATyTCAAAAATTGGGCCTGAAAATCCATACAATACTCCAATATTT
GCTATAAAGAAAAAGGACAGCACyAAATGGAGrAAATTAGTAGAyTTCAGAGAGCTyAAy
AAAAGAACTCArGACTTTTGGGAAGTTCAATTAGGAATACCGCATCCAGCAGGGTTAAAA
AAGAAGAAATCAGTAACAGTACTAGATGTGGGAGATGCATATTTTTCAGTTCCkTTrGAT

GAAAGCTTCAGrAAGTATACTGCATTCACCATACCTAGTATAAACAATGAGACACCAGGA
ATCAGATATCARTATAATGTGCTGCCACAGGGATGGAAAGGrTCACCAGCAATATTCCAG
AGTAGCATGACAAAAATCTTAGAGCCCTTTAGrmTAAAAAATCCAGAAGTAGTTATCTAy
CAATACATGGATGACTTGTATGTAGGATCTGATTTAGAAATAGGGCAGCACAGAACAAAA
GTAGAGGrrCTrAGAGCTCATyTAyTGAGCTGGGGGCTTACTACACCAGACAAAAAGCAT
CAGAAGGAACCTCCATTyCTTTGGATGGGATATGAACTCCATCCTGACAAATGGACAGTC
CAGCCTATAGAACTGCCAGAAAAAGACAGCTGGACTGTCAATGATATACAGAAATTAGTG
GGAAAACTAAAyTGGGCAAGTCAAATCTATCCAGGGATCAAGGTAAAGCAACTrTGTAAA
CTCCTCAGrGGrGCTAAAGCACTAACAGAGGTAGTACCACTGACTGAGGAAGCAGAG

>79
CCTCAAATCACTCTTTGGCAACGACCCGTTGTCACAGTAAAAATAGGAGGGCAGCTGAAA
GAAGCCCTATTAGATACAGGAGCAGATGATACAGTATTAGAAGATATmAATCTGCCAGGA
AAATGGAAACCAAAAATGATAGGGGGAATTGGAGGTTTTATCAArGTAAAGCAATATGAT
CAGATACTTATAGAAATTTGTGGAAAAAGGGCTATAGGTACAGTrTTAGTAGGACCTACA
CCTGTCAACATAATTGGACGAAATATGTTGACTCAGATTGGTTGTACTTTAAATTTCCCA
ATTAGTCCTATTGACACTGTACCAGTAAAATTAAAGCCAGGAATGGATGGACCAAArGTT
AAACAGTGGCCATTGACAGAAGAAAAAATAAAAGCATTAACAGATATTTGTAAAGAAATG
GAACAGGAAGGAAAAATCTCAAAAATTGGGCCTGAAAATCCATACAATACTCCAGTATTT
GCTATAAAGAAAAAGGACAGCACCAAATGGAGAAAATTGGTAGATTTCAGAGArCTTAAT
AAAAGAACTCAGGACTTTTGGGAAGTTCAATTAGGAATACCGCATCCAGCAGGTTTAAAA
AAGAGAAAATCCATAACAGTACTAGATGTGGGAGATGCATATTTTTCAGTTCCACTAGAT
AAAGACTTTAGAAAGTATACTGCATTCACCATACCTAGTACAAACAATGAGACACCAGGA
ATCAGATATCAGTACAATGTGCTTCCACAGGGATGGAAGGGATCACCAGCAATATTCCAA
AGTAGCATGACAAAAATCTTAGAGCCTTTTAGAAAACAAAATCCAGAAATAGATATCTAT
CAATACGTGGATGATTTGTATGTAGCATCTGACTTAGAAATAGGGCAGCATAGAGCAAAA
ATAGAGGAACTGAGGCAACATCTGTTAAGGTGGGGACTTACCACACCAGACAAAAAACAT
CAGAAGGAACCCCCATTCCTTTGGATGGGATATGAGCTCCATCCTGACAAATGGACAGTC
CAGCCTATAGAACTGCCAGAAAAGGACAGTTGGACTGTCAATGATATACAGAAATTAGTA
GrAAAACTAAATTGGGCAAGCCAGATTTATGCAGGGATTAAGGTAAAACAACTGTGTAAr
CTCCTCAGGGGAGCwAAAGCACTAACAGACATAGTACCACTGACTGCAGAAGCAGAG

>80
CCTCAAATCACTCTTTGGCAACGACCmATGGTCACAGTAAAAATAGGAGGGCAGCTrAwA
GAAGCCCTATTAGATACAGGAGCAGATGATACAGTATTAGAAGAAATAAATCTGCCAGGA
AAATGGAAACCAAAAATGATAGGGGGAATTGGAGGTTTTATCAArGTAAAGCAATATGAT

CAGATACTTATAGAAATTTATGGAAAAAGGGCTATAGGTACAGTATTAGTAGGACCTACA
CCTGTCAACATAATTGGACGAAAyATGTTGACTCAGATTGGTTGTACTTTAAATTTCCCA
ATTAGTCCTATTGAyACTGTACCAGTAAAATTAAAGCCAGGAATGGATGGACCAArGGTT
AAACAGTGGCCATTGACAGAAGAAAAAATAAAAGCATTAACAGATATTTGTAAAGAAATG
GAACAGGAAGGAAAAATCTCAAAAATWGGGCCTGAAAATCCATACAAYACTCCAGTATTT
GCTATAAAGAAAAAGGACAGCACCAAATGGAGAAAATTGGTAGATTTCAGAGAGCTTAAT
AAAAGAACTCAGGACTTTTGGGAAGTTCAATTAGGAATACCkCATCCAGCAGGTTTAAAA
AAGAGAAAATCCATGACAGTACTAGATGTGGGAGATGCATATTTTTCAGTTCCwCTAGAT
AAArACTTTAGAAAGTATACTGCATTCACCATACCTAGTAyAAACAATGAGACACCAGGA
ATCAGATATCAGTACAATGTGCTTCCACAGGGATGGAAGGGATCACCAGCAATATTCCAA
AGTAGCATGryAAAAATCTTAGArCCTTTTAGAAAACAAAATCCAGAAATAGACATCTAT
CAATACGTGGATGATTTGTATGTAGsATCTGACTTAGAAATAGGGCAGCATAGAGCAAAA
GTAGAGGAACTGAGGCAACATCTGTTAvGGTGGGGACTTACCACACCAGACAAAAAACAT
CAGAAGGAACCTCCATTCCTTTGGATGGGATATGAACTCCATCCTGACAAATGGACAGTC
CArCCTATAGAACTGCCAGAAAAGGACAGTTGGACTGTCAATGATATACAGAAATTAGTA
GGAAAACTAAATTGGGCAAGCCAGATTTATGCAGGrATTAAGGTAAAACAACTGTGTAGA
CTCCTCAGGGGAGCTAAAGCACTAACAGACATAGTACCACTGACTAGAGAAGCAGAG

>81
CCTCAAATCACTCTTTGGCAACGACCCCTTGTTACCATAAAGATAGGGGGGCAATTAAAG
GAAGCTCTACTAGATACAGGAGCAGATGATACAGTATTAGAAGACATGGATTTGCCAGGG
AGATGGAAACCAAAAATGATAGGGGGAATTGGAGGTTTTATCAAAGTAAGACAGTATGAA
CAGATACCCATAGAAATCTGTGGACATAAAGCTATAGGTACAGTATTAGTAGGRCCTACA
CCTGTCAACATAATTGGAAGAAATCTGTTGACTCAGCTTGGTTGTACTTTAAATTTTCCA
ATCAGTCCTATTGAAACTGTACCAGTAAAACTAAAGCCAGGAATGGATGGCCCAAAGGTT
AAACAATGGCCATTGACAAAAGAGAAAATAGAAGCATTAACAGCAATTTGTGATGAAATG
GAAAAGGAAGGAAAAATTACAArAATTGGGCCTGAAAATCCATACAACACTCCAATATTT
GCCATAAAAAAGAAAGACAGTACTAAGTGGAGAAAATTAGTAGATTTCAGGGAACTCAAT
AAAAGAACTCAAGATTTTTGGGAAGTTCAATTAGGAATACCACACCCAGCAGGATTAAAA
AAGAAAAAATCAGTGACAGTGCTGGATGTGGGAGATGCATATTTTTCAGTTCCTTTATAT
GAAGACTTCAGGAAATATACTGCATTCACCATACCTAGTrTAAACAATGAAACACCAGGG
ATTAGGTATCAGTACAATGTACTYCCACAGGGATGGAAAGGATCACCAGCAATATTTCAA
TGTAGCATGACAAAAATCTTAGAGCCTTTTAGAAAACAAAATCCAAACATAGTCATCTAT
CAATACATGGATGATTTGTATGTAGGATCTGACTTAGAGATAGGACAGCAyAGAACAAAA
ATAGAGGAACTAAGACAACATTTGTTGArGTGGGGATTTACCACACCAGACAAGAAACAT
CAGAAAGAACCTCCATTTCTTTGGATGGGGTATGAACTCCATCCTGACAAATGGACAGTA
CAGCCTATACAGCTGCCAGTACAAGATAGCTGGACTGTCAATGATATACAAAAGTTAGTG
GGAAAATTAAACTGGGCAAGTCAGATTTATCCTGGAATTAAAGTAAGGCAACTTTGTAAA
CTCCTYAGGGGGGCCAAAGCACTAACAGACATAGTACCACTAACTGAAGAAGCAGAG

>82
CCTCAAATCACTCTTTGGCAACGACCCCTTGTCACAATAAAAATAGCAGGACAGCTGAGA
GAAGCTCTATTAGATACAGGAGCAGATGATACAGTATTAGAAGATATAAATTTGCCAGGA
AAATGGAAGCCAAAAATGATAGGGGGAATTGGAGGTTTTATCAAGGTCAGGCAATATGAT
CAGATACTTATAGAAATTTGTGGAAAAAAGGCTATAGGTACAGTGTTAGTAGGACCTACA
CCTGTCAACATAATTGGACGAAATATGTTGACTCArCTTGGTTGTACTTTAAATTTCCCA
ATTAGTCCTATTGACACTGTACCAGTAAAATTAAAGCCAGGAATGGATGGGCCAAAGGTT
AAACAATGGCCATTGACAGAAGAAAAAATAAAAGCATTAACAGAAATTTGTAAAGAAATG
GAAGAGGAAGGAAAAATCTCAAAAATTGGGCCTGAAAATCCATACAATACTCCAGTATTT
GCTATAAAGAAAAAAGACAGCACCAAATGGAGGAAATTGGTAGACTTCAGAGAGCTCAAT
AAAAGAACTCAGGACTTTTGGGAAGTTCAGTTAGGAATACCGCATCCAGCAGGTTTAAAA
AAGAAAAAAATCAGTAACAGTACTAGATGTAGGAGATGCmTATTTTTCAGTTCCTTTAGAT
GAAAGCTTTAGAAAGTATACTGCATTCACCATACCTAGTAGAAACAATGAGACACCAGGA
ATTAGATATCArTACAATGTGCTTCCACAGGGATGGAAAGGATCACCAGCAATATTCCAA
AGTAGCATGACAAAAATCTTAGAGCCTTTTAGAAAACAAAATCCAGACATAGArATCTAT
CAATACATGGATGATTTGTATGTAGGATCTGACTTAGAAATAGGGCAGCATAGAACAAAA
ATAGAGGAGCTGAGACAACATTTGTTGAAGTGGGGACTTACCACACCAGACAAGAAACAT
CAGAArGAACCCCCGTTCCTTTGGATGGGTTATGAACTCCATCCTGATAAATGGACAGTA
CAGCCTATAGCGCTGCCAGATAAGGATAGCTGGACTGTCAATGACATACAAAAGTTAGTG
GGAAAATTAAATTGGGCrAGTCAAATTTATGCAGGGATCAArGTGAAGCAACTGTGCAAG
CTCCTCAGGGGAGCTAAGGCGCTAACAGACATAGTACCACTAACTGAAGAAGCAGAG

>83
CCTCAAATCACTCTTTGGCAACGACCCATTGTCACAGTAAAAATAGGAGGACAGCTAAAA
GAAGCTCTATTAGATACAGGAGCAGATGATACAGTATTAGAAGATATAAATTTGCCAGGA
AAATGGAArCCAAAAATGATAGGGGGAATTGGAGGTTTTATCAAGGTAAGACAATATGAT
CAGATAhCTATAGAAATTTGTGGACAAAAGGCTATAGGTACAGTGTTRGTAGGACCTACA
CCTGTCAACATAATTGGGCGAAATATGTTGACTCAGATTGGCTGTACTTTAAATTTCCCA
ATTAGTCCTATTrACACTGTrCCAGTAAmATTAAAGCCAGGAATGGATGGACCAAAAGTT
AAACAATGGCCATTAACAGAAGAAAAAATAAAAGCATTAACAGAAATTTGTAAAGAGATG
GAGGCAGAAGGAAAAATCTCAAAAATTGGGCCTGAAAATCCATACAATACTCCAATATTT
GCTATAAAGAAAAAGGATGGCACCAAATGGAGAAAATTAGTAGACTTyAGAGArCTCAAT
AAAAGAACTCAGGACTTTTGGGArGTTCAATTAGGAATACCrCATCCAGCAGGwTTAAAA
AAGAAAAAAATCAGTAACAGTACTAGATGTGGGAGATGCATATTTTTCAGTTCCTTTAGAT
GAAAGCTTTAGAAAGTATACTGCATTCACCATACCTAGTATAAACAATGAGACACCAGGA
ATCAGATATCAGTACAATGTGCTGCCACAAGGATGGAAAGGATCACCdGCAATATTyCAA

AGyAGCATGACAAAAATCTTAGAGCCCTTTAGAATrAAAAATCCAGACATAGTGATyTAT
CAATACATGGATGACTTGTATGTAGGATCTGATTTrGAAATAGAGCAGCACAGArCAAAr
ATAGAGGAGCTrAGAGCTCATCTATTrAGCTGGGGrTTTACTACACCAGACAAAAArCAT
CAGAArGAACCTCCATTyCTTTGGATGGGrTATGAACTCCATCCTGACARATGGACAGTC
CAGCCTATAGAACTGCCAgAAAAAGACAGCTGGACTGTTAATGATATACAGAAATTAGTr
GGAAAGCTAAATTGGGCAAGTCAgATTTATGCAGGGATTAAgATAAAGCAAmTGTGTAAA
CTCMTC

>84
CCTCAGATCACTCTTTGGCAACGACCCCTTGTCTCAATAAAAGTAGGGGGTCAGATAAAA
GAGGCTCTCTTAGACACAGGAGCAGATGATACAGTATTAGAAGAAGTAAATTTGCCAGGA
AAATGGAAACCAAGAATGATAGGAGGAATTGGGGGTTTTATCAAAGTAAGrCAATATGAG
CAAATACCTATAGAAATTTGTGGAAAAAAGGCTATAGGTACAGTATTAGTGGGACCCACA
CCTGTCAACATAATTGGAAGAAATATGTTGACCCArCTTGGATGyACACTAAATTTTCCA
ATCAGTCCCATTGAAACTGTACCAGTAAAATTAAAGCCAGGAATGGATGGCCCAAAGGTT
AAACAATGGCCATTGACAGAAGAGAAAATAAAAGCATTAACAGCAATTTGTGATGAAATG
GArAAAGAAGGAAAAATyACAAAAATTGGGCCTGACAATCCATATAATACTCCAATATTT
GCTATAAAAAAGAAGGAyAGyACTAAGTGGAGAAAATTAGTAGAYTTCAGGGAACTCAAT
AAAAGRACTCAAGATTTTTGGGAAATTCArTTAGGAATACCACACCCAGCAGGGTTAAAA
AAGAARAAATCAGTAACAGTyCTGGATGTGGGTGATGCATATTTCTCAGTTCCTTTAGAT
AAAGACTTCAGGAAGTATACTGCATTTACCATACCTAGTATAAACAATGAGACWCCAGGG
ATTAGATATCAGTAyAATGTGCTTCCACAGGGATGGAAAGGATCACCrGCAATATTCCAA
AGTAGCATGACAAAAATCTTAGAGCCTTTTAGAAAACAAAATCCAGACATAGTTATCTAy
CAATACATGGATGACTTGTATGTAGGATCTGACTTAGAAATAGGGCAGCATAGArCAAAA
ATAGAGGAACTGAGAGAACATCTGTTAAAGTGGGGGTTTACTACACCAGACAAGAAACAT
CAGAAAGAACCTCCATTTCTTTGGATGGGGTATGAACTCCaTCCTGACAAATGGACAGTA
CAGCCTATACAGyTGCCAGAAAAGGATAGCTGGACTGTCAATGATATACAGAAGTTAGTG
GGAAAATTAAACTGGGCAAGTCAGATTTACCCAGGAATTAAAGTAAAGCAACTTTGTAAA
CTCCTTAGGGGGACCAAAGCACTAACAGACATAGTACCACTAACTGAAGAAGCAGAG

>85
CCTCAAATCACTCTTTGGCAACGACCCCTCGTCACAATAAAGATAGGGGGGCAATTAAAG
GAAGCTCTATTAGATACAGGAGCAGATGATACAGTATTAGAAGAAATGAATTTGCCAGGG
AAATGGAAACCAAAAATGATAGGGGGAATTGGAGGTTTTATCAAAGTAAAACAGTATGAA
GArATACCCATAGAGATCAGTGGGCACAAGGCTATAGGTACAGTATTAGTGGGACCYACA
CCTGTCAACATAATTGGAAGAAATyTGTTGACTCAGATTGGTTGCACTTTAAATTTTCCr
ATCAGTCCCATTGAAACTGTACCAGTAAAATTAAAGCCAGGAATGGATGGCCCAAArGTT

AAACAATGGCCATTGACAGAAGAGAAAATAAAAGCATTAACAGAAATTTGTrATGAAATG
GAAAAGGAAGGAAAAATTTCAAAAATTGGGCCTGAAAATCCATATAACACTCCAATATTT
GCCATAAAAAAGAAGGACAGTACTAAGTGGAGGAAATTAGTAGATTTCAGGGAACTCAAT
AAAAGAACTCAAGATTTTTGGGAAGTTCAATTAGGAATACCACACCCAGCAGGGTTAAAA
AAGArAAAATCAGTGACAGTACTGGATGTGGGGGATGCATATTTTTCAGTTCCTTTATAT
GAAGACTTCAGGAAGTATACTGCATTCACCATACCTAGTACAAACAATGAAACACCAGGr
ATTAGGTATCAGTACAATGTACTTCCACAGGGATGGAAAGGGTCACCAGCAATATTCCAA
AGTAGCATGACAAAAATCTTAGAGCCrTTTAGAAAGCAAAATCCAGACATAGATATCTGT
CAATACGTGGATGATTTGTATGTAGGATCTGACTTAGAGATAGGGCAACATAGAACAAAA
ATAGAGGAACTrAGACAACATTTGTTGAGGTGGGGATTTACCACACCAGACAAGAAACAT
CAGAAAGAACCTCCATTTCTTTGGATGGGGTATGAACTCCAyCCTGACAAATGGACAGTA
CAGCCTATACAGCTGCCAGAAAAAGATAGCTGGACTGTCAATGATATACAAAAGTTAGTG
GGAAAATTAAAyTGGGCAAGTCAGATTTATCCTGGAATTAAAGTAAGGCAACTTTGTAAA
CTCCTTAGGGGGrCCAAAGCACTAACAGACATAGTACCACTAACTGAAGAAGCAGAG

>86
CCTCAAATCACTCTTTGGCAACGACCCCTCGTCyCAATAAAGATAGGGGGGCAAGTAAAG
GAAGCTCTATTAGATACAGGAGCrGATGATACAGTATTAGAAGAsATGAATTTrCCAGGA
AGATGGAAACCAAAAATGATAGGGGGAATTGGAGGTTTTATCAAAGTAAGACAGTATGAT
CAGATAbCCATAGAAATYTGyGGACACAAGACAGTAGGTACAGTATTAATAGGACCTACA
CCTGTTAAyATAATTGGRAGAAATCTGTTrACTCAGCTTGGTTGCACTTTAAAyTTTCCC
ATTAGTCCTATTGAAACTGTACCAGTAAAATTAAAGCCAGGAwTGGATGGCCCAAAAGTT
AAACAATGGCCATTAACAGAAGAAAAAATAAAAGCATTAGTAGAAATTTGyACAGAAATG
GAAAAGGAAGGAAAAATTTCAAAAATCGGGCCTGAAAATCCATACAATACTCCAGTATTT
GCCATAAAGAAAAAAGACAGTACTAAATGGAGAAAATTAGTAGAyTTCAGAGAACTCAAT
AAAAGrACTCAAGACTTCTGGGAAGTTCAATTAGGAATACCACATCCyGCAGGGTTAAAA
AAGAGAAAATCTGTAACAGTmCTGGATGTGGGkGATGCATACTTCTCAGTCCCTTTAGAT
rAAGAATTTAGrAArTATACTGCATTTACCATACCTAGTTTAAACAATGAGACACCAGGG
ATCAGATATCAGTACAATGTGCTTCCACAGGGATGGAArGGATCACCAGCAATATTCCAA
TGTAGCATGACAAAAATTTTGGAGCCTTTTAGAAArCAAAATCCAGACATAGwTATCTAT
CAATACATGGATGAyTTGTATGTAGGATCTGACTTAGAAATAGGGCAACATAGAGCAAAA
ATAGArGAACTGAGACAACATCTGTTrAGwTGGGGrTTTACCACACCAGACAAAAAACAT
CAGAAAGAACCTCCATTCCTTTGGATGGGkTATGAACTCCATCCTGATAAATGGACrGTA
CAGCCTATAGTGyTGCCAGAAAAGGACAGCTGGACTGTCAATGACATACAGAAGTTAGTG
GGAAArTTGAATTGGGCAAGTCAGATTTATGCAGGGATTAAGGTAArGGAATTATGTAAA
CTCCTTAGrGGAACCAAArCACTAACAGAAGTAATACCACTAACAGAAGAAGCAGAG

>87
CCTCAAATCACTCTTTGGCArCGACCCCTCGTCACAATAAAGATAGGGGGrCAATTAAAG
GAAGCTCTRTTAGATACAGGAGCAGATGATACAGTATTAGAAGAmATGAATTTGCCAGGA
AGATGGAAACCAAAAATGATAGGGGGAATTGGAGGTTTTATCAAAGTAAGACAGTATGAT
CAGATACCCATAGAAATyTGyGGACACAAGGCTGwAGGTACAGTATTAATAGGACCTACA
CCTGTyAACATAATTGGrAGAAATTTGTTGACTCAGCTTGGTTGCACTTTAAATTTTCCC
ATTAGTCCTATTGAAACTGTACCAGTAAAGTTAAAGCCAGGrATGGATGGCCCAAAAGTT
AAACAATGGCCmTTGACrGAAGAAAAAATAAAAGCATTAGTAGAAATTTGTACAGAAATG
GArAAGGAAGGrAAAATTTCAAAAATyGGGCCTGAAAATCCATACAATACTCCAGTrTTT
GCyATAAAGAAAAAAGACAGTACTAAATGGAGAAAATTAGTAGATTTCAGrGAACTTAAT
AAAAGAACACAAGACTTCTGGGAAGTTCAATTAGGAATACCACATCCTGCAGGGyTAAAA
AAGAAAAAATCyrTAACAGTyCTGGATGTGGGTGATGCATATTTyTCAGTCCCTTTAGAT
AAAGACTTCAGGAAGTATACTGCATTYACCATACCTAGTGTAAACAATGAGACACCAGGG
ATCAGrTATCAGTACAATGTrCTTCCACAGGGATGGAAAGGATCACCAGCwATATTCCAA
TGTAGCATGACAAAAATCTTAGAGCCTTTTAGAAArCAAAATCCAGAyATAGTTATCTAT
CArTACATGGATGAyTTGTATGTAGGATCTGACTTAGAAATAGGGCAGCATAGArCAAAA
ATAGAGGAACTGAGACAACATTTGTTGAGGTGGGGATTTACCACACCAGACAAAAAACAy
CAGAArGAACCyCCATTCCTTTGGATGGGTTATGAACTCCATCCTGATAArTGGACAGTT
CAGCCTATAGTGCTGCCAGAAAAGGACAACTGGACTGTCAATGACATACAGAAGTTAGTG
GGAAAGTTGAATTGGGCAAGTCAGATTTATGCAGGGATTAAGGTrAGGGAATTATGTAAA
CTCCTTAGGGGAACCAArGCACTAACAGArGTrATACCACTAACAGAAGAAGCAGAG

>88
CCTCAAATCACTCTTTGGCAACGACCCCTTGTCACAATAAAAGTAGGAGGACAGCTGAAA
GAAGCTCTATTAGATACAGGAGCAGATGATACAGTATTAGAAGATATAAATTTGCCAGGA
AAATGGAAACCAAAAATGATAGGGGGAATTGGAGGTTTTATCAAAGTAAGGCAATATGAT
CAGATACTTATAGAAATTTGTGGAAAAAAGGCTATAGGTACAGTGTTAGTAGGACCTACA
CCTGTCAACATAATTGGACGAAATATGTTGACTCAGCTTGGTTGTACTTTAAATTTTCCr
ATTAGTCCTATTGACACTGTACCAGTAACATTAAAGCCAGGAATGGATGGACCAAAGGTT
AAACAGTGGCCATTGACAGAAGAAAAAATAAAAGCATTAACAGAAATTTGTAGGGAAATG
GAAGAGGAAGGAAAAATCTCAAGAATTGGGCCTGAAAATCCATATAATACTCCAGTATTT
GCTATAAAGAAAAAGGACrGCACCAAATGGAGGAAATTAGTAGATTTCAGAGAGCTCAAT
AAAAGAACTCAGGACTTTTGGGAAGTACAATTAGGAATACCGCATCCAGCAGGATTAAAA
AAGAAAAAATCAGTGACAGTACTAGATGTGGGAGATGCATATTTTTCAGTCCCTTTAGAT
GAArrCTTTAGAAAGTATACTGCATTCACCATACCTAGTAGAAACAATGAGACACCAGGA
ATCAGATATCAGTACAATGTGCTACCACAGGGATGGAAAGGATCTCCGGCAATATTCCAG
TGTAGCATGACAAAAATCTTAGAGCCCTTTAGAAAACAAAATCCAGAAATGGTTATCTAT
CAATACATGGATGACTTGTATGTAGGATCTGATTTAGAAATAGGGCAGCACAGAACAAAA
ATAGATGAGCTAAGAGCTCATCTATTrAGCTGGGGATTTACTACACCAGACAAAAAGCAT

CAGAAGGAACCGCCATTTCTkTGGATGGGATATGAACTCCATCCGGACAGATGGACAGTC
CAGCCTATAGAACTGCCAGAAAAAGACAGCTGGACTGTCAATGATATACAGAAATTAGTG
GGAAAACTAAATTGGGCAAGTCAAATTTATGCAGGGATTAAGGTAAGGCAACTGTGTAAR
CTCCTCAGGGGAGCTAAAGCATTAACAGACGTAGTACCACTGACTGAAGAAGCAGAG

>90
CCTCAAATCACTCTTTGGCAACGACCCCTTGTTACAGTAAAAATAGGAGGACAGATGAAA
GAAGCTCTATTAGATACAGGGGCAGATGATACAGTATTAGAAGAyATAAATTTGCCAGGA
AAGTGGAAACCAAAAATGATAGGGGGAATTGGAGGTTTTATCAAGGTAAAGCAATATGAT
CAGATACTTATAGAAATTTGTGGAAAAAGGGCTATAGGTACAGTGTTAGTAGGACCTACA
CCTrTCAACATAATTGGACGAAATATGTTGACTCAGATTGGTTGTACTTTAAATTTCCCA
ATTAGTCCTATTGACACTGTACCAGTAACATTAAAGCCAGGAATGGATGGACCAAAAGTT
AAACAGTGGCCATTAACAGAAGAAAAAATAAAAGCATTAACAGAAATTTGTAAAGAGATG
GAAGAGGAAGGAAAAATCTCAAAAATTGGGCCTGAAAATCCATACAATACTCCAATATTT
GCTATAAAGAAAAAGGACAGCACCAAATGGAGGAAATTAGTAGATTTCAGAGAGCTCAAT
AAAAGAACTCAGGACTTTTGGGAAGTTCAATTAGGAATACCGCATCCAGCAGGTTTrArG
AAAAAGAAATCAGTAACAGTACTrGATGTGGGAGATGCATATTTTTCAGTTCCTTTAGAT
GAAAGCTTTAGAAAGTATACTGCATTTACCATACCTAGTACAAACAATGAGACACCAGGA
ATCAGATATCAGTACAATGTGCTGCCACAGGGATGGAAAGGATCACCGGCAATATTCCAG
AGTAGCATGACAAAGATCTTGGAGCCCTTTAGAACAAAAAATCCAGAAATAGTTATCTAT
CAATACATGGATGACTTGTATGTAGGCTCTGATTTAGAAATAGGGCAGCACAGAATAAAA
ATAGAGGAGCTGAGAGCTCATCTATTGAGCTGGGGATTTACTACCCCAGACAAAAAGCAT
CAGAAGGAACCTCCATTCCTTTGGATGGGATATGAACTCCATCCTGACAAATGGACAGTC
CAGCCTATAGAACTGCCAGAAAArGACAGCTGGACTGTCAATGATATACAGAAATTAGTG
GGGAAACTCAATTGGGCAAGTCAAATTTATGCAGGAATTAAGATAAAACAACTGTGTAAA
CTCCTCAGGGGAACTAAAGCACTAACAGACATAGTGCCATTGACTGAAGAAGCAGAG

>92
CCTCAAATCACTCTTTGGCAACGACCCCTCGTCACAATAAAGATAGGGGGGCAACTAAAG
GAAGCTCTATTAGACACAGGAGCAGATGATACAGTGTTAGAAGAAATGAACTTACCAGGA
AGATGGAAACCAAAAATGATAGGGGGAATTGGAGGTTTTATCAAAGTAAGACAGTATGAT
CAGATAGCCATAGAAATCTGTGGACATAAAGCTATAGGTACAGTATTAATAGGACCTACA
CCTGTCAACATAATTGGAAGAAATCTGTTGACTCAGATTGGCTGCACTTTAAATTTTCCT
ATTAGTCCTATTGAAACTGTACCAGTAAAATTAAAGCCAGGAATGGATGGCCCAAAAGTT
AAACAATGGCCATTGACAGAAGAAAAAATAAAAGCATTAGTAGAAATTTGTACAGAAATG
GAAAAGGAAGGGAAAATTTCAAAAATTGGGCCTGAAAATCCATACAACACTCCAGTATTT

GCTATAAAGAAAAAAGACAGTACTAAATGGAGAAAATTAGTAGATTTCAGAGAACTTAAT
AAGAGAACTCAAGACTTCTGGGAGGTCCAATTAGGAATACCACACCCTGCAGGGTTAAGA
AAGAGAAAATCAGTAACAGTrCTGGATGTGGGTGATGCATATTTTTCAGTTCCCTTAGAT
GAAGACTTCAGGAAGTATACTGCCTTTACCATACCTAGTACAAACAATGAGACACCAGGG
ATTAGATATCAGTACAATGTGCTTCCACAGGGATGGAAAGGATCACCAACAATATTCCAA
AGTAGCATGACAAAAATCTTAGAGCCTTTTAGAAAACAAAATCCAGCAATAGAGATCTGT
CAATACGTGGATGACTTGTATGTAGGATCTGACTTAGAAATAGGGCAACATAGAGCAAAG
ATAGAGGAACTGAGAGAACATCTGTTAAGGTGGGGATTAACCACACCAGATAAAAAACAT
CAGAAAGAACCTCCATTCCTTTGGATGGGGTATGAACTCCATCCTGACAAATGGACAGTA
CAGCCCATAATGCTGCCAGAAAAGGACAGCTGGACTGTCAATGACATACAGAAGTTAATA
GGAAAATTAAATTGGGCAAGTCAAATTTATGCAGGGATTAAAGTAAAACAACTATGTAAA
CTCCTTAGGGGAACCAAAGCACTAACAGAAGTAGTACCATTAACCGAAGAAGCAGAG

>93
CCTCAAATCACTCTTTGGCAACGACCCCTTGTCACAATAAAGATAGGAGGACAGCTAAAA
GAAGCTCTATTAGATACAGGAGCAGATGATACAGTATTAGAAGATATAAATTTGCCAGGr
AAATGGAAACCAAAAATGATAGGGGGAATTGGAGGTTTTATCAAGGTAAGGCAATATGAT
CAGATACTTATAGAAATTTGTGGrAAAAAGGCTATAGGTACAGTATTAGTAGGACCTACA
CCTGTCAACATAATTGGACGAAATATGTTGACTCAGATTGGTTGTACTTTAAATTTCCCA
ATTAGTCCTATTrACACTGTACCAGTAACATTAAAGCCAGGAATGGATGGACCAAAGGTT
AAACAGTGGCCATTGACAGAAGAAAAAATAAAAGCATTAACAGAAATTTGTAAAGAGATG
GAAGAGGAAGGAAAAATCTCAAAAATTGGGCCTGAAAATCCATATAATACTCCAGTATTT
GCTATAAAGAAAAAGGATAGCACCAAATGGAGGAAATTAGTAGATTTCAGAGAACTTAAT
AAAAGAACTCAGGACTTTTGGGAAGTTCAATTAGGAATACCACATCCAGCAGGATTAAAA
AAGAAAAAATCAGTGACAGTACTAGATGTGGGAGATGCATATTTTTCAGTTCCTTTAGAT
GAAAGCTTTAGAAAGTACACTGCATTCACCATACCTAGTATAAACAATGAAACACCAGGA
ATCAGATATCAGTACAATGTGCTACCACAGGGATGGAAAGGATCTCCGGCAATATTCCAr
TGTAGCATGACAAAAATCTTAGAGCCCTTCAGAAGAAAAAATCCAGAGATGATTATCTAT
CAATACATGGATGACTTGTATGTAGCATCTGATTTAGAAATAGGGCAGCACAGAACAAAA
ATAGAGGAGCTAAGAGCCCATCTATTGAGCTGGGGATTTACTACACCAGACAAAAAGCAT
CAGAAGGAACCTCCATTTCTTTGGATGGGATATGAACTCCATCCGGAyAGATGGACAGTC
CAGCCTATAGAACTGCCGGAAAAAGACAGCTGGACTGTCAATGATATACAGAAACTAGTG
GGAAAACTAAATTGGGCAAGTCAAATTTATGCAGGGATTAAGGTAAAGCAACTGTGTAGA
CTCCTCAGGGGAGCTAAAGCACTAACAGACATAGTACCACTGACTGAAGAAGCAGAG

>94

CCTCAAATCACTCTTTGGCAGCGACCCCTTGTCTCAATAAAAGTAGGGGGACArATAAAG
GAGGCCCTCTTAGACACAGGAGCAGATGATACAGTATTAGAAGAAATmAGTTTGCCAGGA
AAATGGAAACCAAAAATGATAGGAGGAATTGGAGGTTTTATCAAAGTAAGACAGTATGAT
CAAATATCTATAGAAATATGTGGAAAAAAGGCTATAGGTACAGTATTAGTGGGACCTACA
CCTGTCAACATAATTGGAAGAAATTTGTTGACTCAGCTTGGATGCACACTAAATTTTCCA
ATTAGTCCTATTGAAACTGTACCAGTAAAATTAAAGCCAGGAATGGATGGCCCAAAGGTT
AGACAATGGCCATTGACAGAAGAAAAAATAAAAGCATTAACAGAAATTTGTTTAGAAATG
GAAAAAGAAGGAAAAATTTCAAAAATTGGGCCTGAAAATCCATATAACACTCCAGTATTT
GCCATAAAAAAGAAGGACAGTACTAAGTGGAGAAAATTAGTAGATTTCAGGGAACTCAAT
AAAAGAACTCAAGACTTTTGGGAAGTTCAATTAGGAATACCACACCCAGCAGGGTTAAAA
AAGAAAAAATCAGTGACAGTACTGGATGTGGGAGATGCATATTTTTCAGTACCTTTAGAT
GAAAATTTCAGrAAATATACTGCATTCACCATACCTAGTATAAACAATGAAACACCAGGA
ATTAGATATCAATATAATGTGCTTCCACAGGGATGGAAAGGATCACCAkCTATTTTCCAG
AGTAGCATGACAAAAATTTTAGAGCCCTTTAGGACACAAAATCCAGAAATGGTCATCTAT
CAATATATGGATGACTTGTATGTGGGATCTGACTTAGAAATAGGGCAACATAGAGCAAAA
ATAGAGGAGTTAAGAGAACATTTATTAAAGTGGGGATTTACCACACCAGACAAGAAACAT
CAGAAAGAACCCCCATTTCTTTGGATGGGGTATGAACTCCATCCTGACAAATGGACAGTA
CAGCCTATACAGCTGCCAGTCAAGGATAGCTGGACGGTCAATGATATACAGAAGTTAGTG
GGAAAATTAAACTGGGCAAGTCAGATTTACCCAGGGATTAGGGTGArGAATCTTTGTAGA
CTACTTAGGGGAGCCAAAGCACTAACAGACATAGTACCACTAACTGAAGAAGCAGAG

>75
CCTCARATCACTCTTTGGCAGCGACCCCTCGTCwCAATAAAGATAGGGGGrCAACAAAAG
GAAGCTTTATTAGATACAGGAGCAGATGATACAGTATTAGAAGAAATGCATTTACCAGGA
ArATGGAAACCAAAAATGATAGGGGGAATTGGAGGTTTTATCAAAGTAAGRCAGTATGAT
CAGATACTCATAGAAATTTGTGGACATAArGCTATAGGTACAGTATTAATAGGACCTACA
CCTGTCAACATAATTGGAAGAAATCTGTTGACTCAGATTGGCTGCACTTTAAATTTTCCC
ATTAGTCCTATTGAAACTGTACCAGTrAAATTAAAGCCAGGTATGGATGGCCCAAAAGTT
AAACAATGGCCATTGACAGAAGAAAAAATAAAAGCATTAGTAGAAATTTGTACAGAAATG
GAAAAGGAAGGAAAAATTTCAAAAATAGGGCCTGAAAATCCATACAATACTCCAGTATTT
GCAATAAAGAAAAAAGACAGTACTAAATGGAGAAAATTAGTAGATTTCAGAGAACTCAAT
AAAAGAACTCAAGACTTCTGGGAGGTTCAATTAGGAATACCACATCCCGCAGGGTTAGAA
AAGAAAAAATCAATAACAGTACTGGATGTGGGTGATGCATATTTTTCAATTCCCTTrkAT
GAGGACTTTAGGAAGTATACTGCATTTACCATACCTAGTACAAACAATAAAACACCAGGG
ATTAGGTATCAGTACAATGTGCTTCCACAGGGATGGAAAGGATCACCAGCAATATTCCAA
GGTAGCATGACAAAAATCCTAGATCCTTTTAGAAAACAAAATCCAGACATAGTGATCTAT
CAGTACATGGATGATTTGTATGTAGGATCTGACTTAGAAATAGGGCAACATAGAACAAAA
GTAGAGGAACTGAGACAACATCTGTTrAGGTGGGGATTAACCACACCAGACAAAAAACAT
CAGAAAGAACCTCCATTCCTTTGGATGGGTTATGAACTCCATCCTGATAAATGGACAGTA

CAACCTATAATACTGCCAGAAAAGGACAGCTGGACTGTCAATGACATACAGAAGTTrATA
GGAAAACTGAATTGGGCAAGTCAAATTTATGCAGGGATTAAAGTAAAGCAATTATGTAAA
CTCC
