# Supplementary material for: HIV drug resistance in HIV positive individuals under antiretroviral treatment in Shandong Province, China
Source: PLoS One. 2017 Jul 27;12(7):e0181997. doi: 10.1371/journal.pone.0181997 (PMC5531464; doi:10.1371/journal.pone.0181997)
Supplement: S3 File — (PDF) [file pone.0181997.s003.pdf]

>1
---------------------CCCACCAGCAGAAGACTGGGGAATGGGAGGAGAGAAAAC
AGC---------CYTACCGAAGCAGG---------------AGCAGAAAGACCAGGACCA
------TCCCTCAATTTCCCTCAAATCACTCTTTGGCAACGACCCCTTGTCACAATAAAA
ATAGGAGGACAGCTAAAAGAAGCTCTGTTAGATACAGGAGCAGATGATACAGTATTAGAA
GATATAAATTTGCCAGGAAAATGGAAACCAAAAATGATAGGGGGAATTGGAGGTTTTATC
AAGGTAAGGCAATATGATCAGATACTTATAGAAATTTGTGGAAAAAAGGCTATAGGTACA
GTATTAGTAGGACCTACACCTGTCAACATAATTGGACGRAATATGTTGACTCAGATTGGT
TGTACTTTAAATTTCCCAATTAGTCCTATTGACACTGTACCAGTAACATTAAAGCCAGGA
ATGGATGGACCAAAGGTTAAACAGTGGCCATTGACAGAAGAAAAAATAAAAGCATTAAYA
GAAATTTGYARAGARATGGAAGAGGAAGGAAAAATCTCAAAAATTGGGCCTGAAAATCCA
TATAATACCCCAGTATTTGCTATAARGAAAAAGGACAGCAMCAAATGGAGGAAATTAGTA
GATTTCAGAGAGCTCAATAAAAGAACTCAGGACTTTTGGGAAGTTCAATTAGGAATACCR
CATCCAGCAGGATTAAAAAAGAAAAAATCAGTGACAGTATTAGATGTGGGAGATGCATAT
TTTTCAGTTCCTYTAGATGAAAGCTTTAGAAAGTATACWGCATTCACCATACCTAGTATA
AACAATGAGACACCAGGAATCAGATAYCARTACAATGTGCTACCACAGGGATGGAAAGGA
TCTCCRGCAATATTCCAGWGTAGCATGACAAAAATCTTAGAGCCCTTTAGARCAAAAAAT
CCAGAGATKGTTATCTATCAATACATGGATGACTTGTATGTAGGATCTGATTTAGAAATA
GGGCAGCACAGAATAAAAATAGAGGARCTRAGAGCTCATCTATTGAGCTGGGGATTTACT
ACACCAGACAAAAARCATCAGAAGGAACCTCCATTTCTTTGGATGGGATATGAACTCCAT
CCGGACARATGGACAGTCCAGCCTATACAACTGCCAGAAAAAGACAGCTGGACTGTCAAT
GATATACAGAARTTAGTGGGAAAAYTAAATTGGGCAAGTCAAATTTATGCAGGGATYMRG
GTAAAGCAACTGTGTAAACTCCTCAGGGGAGCTAAAGCACTAACGGACATAGTACCACTG
ACTGAAGAAGCAGAG---

>2
------------------------ACCAGCAGAGGACKGGGGGATGGAAGGAGAGATAAC
CTC---------CTTACCRAAGCAGG---------------AGCAGAAAGACAAGGAACA
------TCCTTCAGTYTCCCTCAAATCACTCTTTGGCAACGACCCCTTGTCACAATAARR
RTAGAAGGACAGCTAAGAGAAGCTCTATTAGATACAGGAGCAGATGATACAGTATTAGAA
GRTATAAATTTGCCAGGAAAATGGAAACCAAAAATGATAGGGGGAATTGGAGGTTTTATC
AAGGTAAGGCAATATGATCARATACTTATAGAAATTTGTGGAAAAAGGGCTATAGGTACA
GTGTTAGTAGGACCTACACCTGTCAACATAATTGGACGAAATATGTTGACTCAGCTTGGT
TGTACTTTAAATTTCCCAATTAGTCCTATTGACACTGTTCCAGTAACATTAAAGCCAGGA
ATGGATGGACCAAAGGTCAAACARTGGCCATTGACAGAAGAAAAAATAAAAGCATTAACA
GAAATTTGTAARGAGATGGAAGAGGAAGGAAAAATCTCAAAAATTGGGCCTGAAAATCCA
TATAATACTCCAGTATTTGCTATAAAGAAAAAGGACAGTACCAAATGGAGAAAATTAGTA
GATTTYAGAGARCTCAATAAAAGAACTCAGGACTTTTGGGAAGTTCAATTAGGAATACCG
CATCCAGCAGGATTGAAAAAGAAAAAAGCAGTGACAGTACTAGATGTAGGAGATGCATAT
TTTTCAGTTCCTTTAGATGAAAACTTTAGAAAGTATACTGCATTYACCATACCTAGTACA
AACAATGAGACACCAGGAATCAGATATCAGTACAATGTGCTACCACAGGGATGGAAAGGA
TCTCCKGCAATATTTCAGTGTAGCATGACAAAAATCTTAGAGCCCTTTAGAAKWMAAAAT
CCAGARATAGTTATCTATCAATACATGGATGACTTRTATGTAGGATCTGATTTAGAAATA
GGGCAGCACAGAGCAAAAATAGARGAGCTAAGAGCTCAYCTATTGAGCTGGGGATTTACY

ACACCAGACAAAAAGCATCAGAAGGARCCTCCATTTCTTTGGATGGGATATGAACTCCAT
CCGGACAGATGGACAGTCCAGCCTATAGAAYTGCCAGAAAAGGATAGCTGGACTGTCAAT
GATATACAGAAATTAGTGGGAAAAYTAAATTGGGCAAGTCAAATTTATGSAGGGATTAGG
RTAAAGCAACTGTGTAAACTCCTCAGGGGAGCTAAAGCACTAACAGACATAGTACCACTG
ACAGAAGAAGCAGAGC--

>4

---------------TCAGAAGCAGG---------------AGCCGATAGACAAGGAACT
ATA---TCCTTTAGCCTCCCTCAAATCACTCTTTGGCAACGACCCCTYGTCMCARTAAGR
RTAGGGGGGCAATTAAAGGAAGCTYTATTAGATACAGGAGCAGATGATACAGTATTAGAA
GACATGAATTTGCCAGGAAGATGGAAACCAAAAATGATAGGGGGAATTGGAGGTTTTATC
AAAGTAARACAGTATGATCARRTACCYATAGAAATCTGTGGACACAAGGCTGTAGGTACA
GTRTTAATAGGACCTACACCTGTCAACATAATTGGRAGAAATYTGTTGACTCAGCTTGGT
TGCACTTTAAATTTTCCCATTAGTCCTATTGAAACTGTACCAGTAAAATTAAAGCCAGGA
ATGGATGGCCCAAAAGTTAAACAATGGCCATTGACAGAAGAAAARATAAAAGCWTTAGTA
GAAATYTGTACAGAAATGGARAAGGAAGGRAAAATTTCAAAAATYGGGCCTGAAAATCCA
TACAATACTCCAGTATTTGCYATAAAGAAAAAAGACAGTACTAAATGGAGAAAATTRGTA
GATTTCAGGGAACTTAATAAAAGAACTCAAGACTTCTGGGAAGTTCAATTAGGAATACCA
CATCCTGCAGGGTTAAAAAAGAAAAAATCCGTAACAGTYYTGGATGTGGGTGATGCATAT
TTCTCAGTYCCTTTAGATAAAGAYTTYAGGAAGTATACTGCMTTTACCATACCTAGTRYA
AACAATGAGACACCAGGGATCMGATATCAGTACAATGTGCTTCCACARGGATGGAAAGGA
TCACCAGCAATATTCCAGTRTAGCATGACAAAAATCTTAGASYCTTTTAGRAAACAAAAT
CCAGAYATAGTDATCTATCARTAYATGGATGATTTGTATGTAGGRTCTGACTTAGAAATA
GGRCAGCATAGAGCAAAAATAGAGGAAYTGAGACAACATCTGTTRAGGTGGGGATTTACC
ACACCAGAYAAAAAACATCAGAAAGAACCTCCATTYCTKTGGATGGGTTATGAACTCCAT
CCTGATAAATGGACAGTACARCCTATAGTRCTGCCAGAAAAGGACAGCTGGACTGTCAAT
GACATACAGAAGTTAGTGGGAAAGTTGAATTGGGCAAGTCAGATTTATSCAGGGATTAAR
GTAAAGGARTTATGTAAACTYCTTAGGGGAACCAAAGCRCTAACAGAAGTAATACCACTA
ACAGAAGAAGCAGAGCT---

>5

---------------TCAGAAGCAGG---------------AGCCGATAGACAAGGAACT
ATA---TCCTTTAGCCTCCCTCAAATCACTCTTTGGCAACGACCCCTYGTCMCARTAAGR
RTAGGGGGGCAATTAAAGGAAGCTCTATTAGATACAGGAGCAGATGATACAGTATTAGAA
GACATGAATTTGCCAGGAAGATGGAAACCAAAAATGATAGGGGGAATTGGAGGTTTTATC
AAAGTAARACAGTATGATCARRTACCYATAGAAATCTGTGGACACAARGCTGTAGGTACA
GTRTTAATAGGACCTACACCTGTCAACATAATTGGRAGAAATCTGTTGACTCAGCTTGGT
TGCACTTTAAATTTTCCCATTAGTCCTATTGAAACTGTACCAGTAAAATTAAAGCCAGGA
ATGGATGGCCCAAAAGTTAAACAATGGCCATTGACAGAAGAAAARATAAAAGCWTTAGTA
GAAATYTGTACAGAAATGGARAAGGAAGGRAAAATTTCAAAAATYGGGCCTGAAAATCCA
TACAATACTCCAGTATTTGCYATAAAGAAAAAAGACAGTACTAAATGGAGAAAATTRGTA
GATTTCAGGGAACTTAATAAAAGAACTCAAGACTTCTGGGAAGTTCAATTAGGAATACCA

CATCCYGCAGGGTTAAAAAAGAAAAAATCCGTAACAGTYYTGGATGTGGGTGATGCATAT
TTCTCAGTYCCTTTAGATAAAGAYTTYAGRAAGTATACTGCMTTTACCATACCTAGTRYA
AACAATGAGACACCAGGGATCMGATATCAGTACAATGTGCTTCCACAGGGATGGAAAGGA
TCACCAGCAATATTCCAGTRTAGCATGACAAAAATCTTAGASYCTTTTAGRAAACAAAAT
CCAGAYATAGTDATCTATCARTAYATGGATGATTTGTATGTAGGRTCTGACTTAGAAATA
GGRCAGCATAGAGCAAAAATAGAGGAAYTGAGACAACATCTGTTGAGGTGGGGATTTACC
ACACCAGAYAAAAAACATCAGAAAGAACCTCCATTYCTKTGGATGGGTTATGAACTCCAT
CCTGATAAATGGACAGTACAGCCTATAGTGCTGCCAGAAAAGGACAGCTGGACTGTCAAT
GACATACAGAAGTTAGTGGGRAAGTTGAATTGGGCAAGTCAGATTTATSCAGGGATTAAR
GTAAAGGARTTATGTAAACTCCTTAGGGGAACCAAAGCACTAACAGAAGTAATACCACTA
ACAGAAGAAGCAGAG--

>6
--------------------CCCACCAGCAGAAGACTGGGGGATGGGAGGAGAGACAAC
CCT---------CTTACCGAAGCAAG--------------AGCAGAAAGACAAGGAACA
------TCCCTCAGTTTCCCTCAAATCACTCTTTGGCAACGACCCCTTGTCACAGTAAAA
ATAGGAGGACAGCTAAAAGARGCTCTATTAGAYACAGGAGCMGATGATACAGTATTAGAA
GATATAAATTTGCCAGGAAAATGGAAACCAAAAATGATAGGGGGAATTGGAGGTTTTATC
AAGGTAAGGCAATATGATCAGATACTTATAGAAATTTGTGGAAAAAAGGCTATAGGTACA
GTATTAGTAGGACCTACACCTGTCAACATAATTGGACGAAAYATGTTGACTCAGATTGGT
TGTACTTTAAATTTCCCCATTAGTCCYATTGACACTGTACCAGTAAAATTAAAGCCAGGA
ATGGATGGACCAAAGGTTAAACAGTGGCCATTGACAGAAGAAAAAATAAAAGCATTAGTA
GAAATTTGTACAGAAATGGAAAAGGAAGGAAAAATTTCAAAAATTGGGCCTGAAAATCCA
TACAATACTCCAGTATTTGCTATAAAGAAAAAGGACAGCACCAAATGGAGGAAATTAGTA
GATTTYAGAGAGCTCAATAAAAGAACTCAAGACTTTTGGGAAGTYCAATTAGGAATACCR
CATCCAGCAGGATTAAAAAAGAAAAAATCAGTGACAGTACTAGATGTGGGGGATGCATAT
TTCTCAGTTCCTTTAGATGAAAGCTTTAGAARGTATACTGCATTTACCATACCTAGTATA
AACAATGAGACACCAGGAATCAGATATCAGYATAATGTRCTTCCACAGGGATGGAAAGGA
TCACCAGCAATATTCCAAAGTAGCATGACAAGAATCTTAGAGCCTTTTAGAAAACAAAAT
CCAGACATAGTGATCTATCAATAYATGGATGATTTGTATRTAGGRTCTGACTTAGAAATA
GGGCAACATAGGAYAAAARTAGAGGAACTGAGGCAACATCTGTTGAAGTGGGGATTAACC
ACACCAGACAAAAAACATCAGAAGGAACCTCCATTTCTTTGGATGGGATATGAACTCCAT
CCAGACAGATGGACAGTCCAGCCTATMGAGCTGCCAGAAAAAGACAGCTGGACTGTCAAT
GATATACAGAAACTAGTGGGAAAACTAAATTGGGCAAGTCAAATTTATCCAGGRATTAAA
GTAAAGCAATTRTGTAAACTCCTTAGGGGAACCAAAGCACTAACAGAAGTAGTACCACTA
ACAG---

>7
--------------------CCCACCAGCAGAGAACTGGGGGATGGGGGAAGA------
------------CTTACTGAAGCAGG--------------AGCAGAAAGGCAAGGAACA
TCCCCCTCCTGCAATTTCCCTCAAATCACTCTTTGGCAGCGACCCCTTGTTACAATAAAA
ATAGGAGGACAGCYAAAAGAAGCTCTATTAGATACAGGAGCAGATGATACAGTATTAGAA
GATATAAATTTGCCAGGAAAATGGAAACCAAAAATGATAGGGGGAATTGGAGGTTTTATT
AAGGTAAGACAATATGATCAGGTACCTATAGAAATTTGTGGAAAAAAGGCTGTAGGTACA

GTGTTAGTGGGACCCACGCCTGTCAACATAATTGGACGAAATATGCTGACTCAGATTGGT
TGTACTTTAAATTTTCCAATAAGTCCTATTGACACTGTACCAGTAACATTAAAGCCAGGA
ATGGATGGACCAAAGGTTAAACAGTGGCCATTGACAGAAGAAAAAATAAAAGCATTAACA
GAAATTTGTAAGGAGATGGAAGAGGAGGGAAAAATCTCAAAAATTGGGCCTGAAAATCCA
TACAATACTCCAGTATTTGCTATAAAGAAAAAAGACAGCACCAAGTGGAGGAAATTAGTA
GATTTCAGAGAGCTTAATAAAAGAACTCAGGATTTTTGGGAAGTTCAATTAGGAATACCG
CATCCAGCAGGTTTAGAAAAGAAAAAATCAGTAACAGTACTAGATGTGGGAGATGCATAT
TTTTCAGTTCCTTTAGATGAAAGTTTTAGAAAGTATACTGCATTCACCATACCTAGTATA
AATAATGAGACACCAGGAATCAGATATCAGTACAATGTGCTGCCACAGGGATGGAAAGGA
TCACCAGCAATATTCCAGAGTAGCATGACAAAAATCTTAGAGCCCTTTAGAATAAAAAAT
CCAGAAATGATTATTTATCAATACGTGGATGACTTGTATGTAGCATCTGATCTAGAAATA
GGGCAGCACAGAACAAAAATAGAGGAGCTGAGAGCTCATCTATTGAACTGGGGGTTTACT
ACACCAGATAAAAAGCATCAGAAGGAACCTCCATTCCTTTGGATGGGATATGAACTCCAT
CCTGATAAATGGACAGTCCAGCCTATAGAACTGCCAGAAAAAGATAGCTGGACTGTCAAT
GATATACAGAAATTAGTAGGAAAACTAAATTGGGCAAGTCAAATTTATGCAGGGATTAAG
GTAAAGCAACTGTGTAAGCTCCTCAGAGGAGCTAAAGCACTAACTGACATAATACCATTG
ACTGAAGAAGCAGAGCT--

>8
---------------------CCCACCAATGGAGAGTTTCAGGTTCGAGGAGACAACCCC
AGC------------TCCAAAGCAGG---------------AACCGAAGGACAGGGAA--
-------CCTTTAACTTCCCTCARRTCACTCTTTGGCAACGACCCCTTGTCTCAATAAAA
GTAGGGGGYCAAATAARAGARGCTCTCTTAGACACAGGAGCAGATGATACAGTAYTAGAA
GAAATAAATTTGCCAGGAAAATGGAGACCAAAAATGATAGGGGGAATTGGAGGTTTTATC
AAAGTAAGACAATATGATCAAATACCTATAGAAATTTGTGGAAAAAAGGCTATAGGTRCA
GTATTAGTGGGACCCACACCTGTCAATATAATTGGAAGRAATATGTTGACTCAGCTTGGR
TGCACACTAAATTTTCCAATCAGTCCCATTGAAACTGTACCWGTRAAATTAAAGCCAGGA
ATGGATGGCCCAAARGTKAAACAATGGCCATTGACAGAAGASAAAATAAAAGCATTAACA
GAAATTTGTGAGGACATGGARAARGAAGGAAAAATTACAAAAATTGGGCCTGAAAATCCA
TATAACACTCCAATATTTGCCATAAAAAAGAAGGACAGTACTAAGTGGAGAAAATTAGTA
GATTTCAGGGAACTCAATAAAAGAACTCAAGATTTTTGGGAAGTTCAATTAGGAATACCA
CACCCAGCAGGGTTAAAAAAGAAAAAATCAGTGACAGTACTAGATGTGGGGGATGCATAT
TTTTCAGTTCCTTTACATGAAGACTTCAGGAAGTATACTGCATTCACCATACCTAGTATA
AATAATGAAACACCAGGGATTAGGTATCAATATAATGTGCTTCCACAGGGATGGAAAGGA
TCACCAGCAATATTYCAGAGYAGCATGACAAGAATCTTAGAGCCCTTTAGAGCAMAAAAY
CCAGAAATGGTCATCTATCAATATATGGATGACCTGTATGTAGGATCTGATTTAGAAATA
GGGCAACATAGAACAAAAATAGAGGAGTTAAGAGAACATCTGTTAAAGTGGGGGTTCACC
ACACCAGACAAGAAACATCARAAAGAACCYCCATTTCTTTGGATGGGGTATGAACTCCAT
CCTGACAAATGGACAGTACAGCCTATARAGCTGCCAGAAAAGGATAGCTGGACTGTCAAT
GATATACAGAAGTTAGTGGGAAAAYTAAACTGGGCAAGTCARATTTACCCAGGAATTAAA
GTAAGRCAACTTTGTAAACTCCTTAGGGGRACCAAAGCACTAACAGACATAGTACCACTA
ACTGAAGAAGCAGAGCTAGAACTGGCAGA------------------------------

>9

--ACAAGGARYT
ATA---TCCYTTGACTTCCCTCAAATCACTCTTTGGCAACGACCCCTCGTCCCAATAAGG
ATAGAGGGGCAATTAAAGGAAGCTCTATTAGATACAGGAGCAGATGATACAGTATTAGAA
GACATGAATTTGCCAGGRAAATGGAAACCAAAAATGATAGGGGGAATTGGAGGTTTTATC
AAAGTAAGACAGTATGATCAGGTAWCCATAGAAATCTGTGGACACAAGGCTGTAGGTACA
GTWTTAATAGGACCYACACCTRTCAACATAATTGGRAGAAATYTGTTGACTCAGCTTGGT
TGCACTYTAAATTTTCCYATTAGTCCTATTGAAACTGTACCAGTAAAATTAAAGCCAGGA
ATGGATGGCCCAAAAGTTAAACAATGGCCATTGACAGAAGAAAAAATAAAAGCATTAGTA
GAAATTTGTACAGAAATGGAAAAGGAAGGRAAAATTTCAAAAATTGGGCCTGAAAATCCA
TACAATACTCCAGTATTTGCCATAAAGAAAAAAGACAGTACTAAATGGAGAAAATTAGTA
GATTTCAGGGAACTTAATAARAGAACTCAAGAYTTCTGGGAAGTTCAATTAGGAATACCA
CATCCYGCAGGGYTAAAAAAGAAAAAATCAGTAACAGTYCTRGATGTGGGTGATGCATAY
TTCTCAGTYCCTTTAGATRAAGAHTTCAGRAAGTAYACTGCATTTACCATACCYAGTGTA
AACAATGAGACACCAGGRATCAGRTATCAGTACAATGTGCTTCCACAGGGATGGAAAGGA
TCACCAGCAATATTCCAGTGYAGYATGACAAAAATCTTAGARCCTTTTAGAAAACAAAAT
CCAGACATAGTBATYTATCAATAYATGGATGAYTTGTATGTAGGATCTGAYTTAGAAATA
GGGCAGCATAGAGMAAAAATAGAGGAACTGAGACARCATYTGTTGAGGTGGGGATTTACC
ACACCAGAYAAAAAACATCAGAAAGAACCYCCATTYCTTTGGATGGGTTATGAACTCCAT
CCTGATAAATGGACAGTACAGCCTATAGTGCTGCCAGAAAAGGACAGCTGGACTGTCAAT
GACATACAGAAGTTAGTGGGAAAGTTRAATTGGGCAAGYCARATTTATGCAGGRATTAAG
GTAARGGAATTATGTAAACTCCTTAGGGGAACCAAAGCWCTAACAGAAGTAGTACCACTA
ACAGAAGAAGCARAGCTAGAACTGGCAGA-------------------------------

>10
---------------------CCCACCAGCAGAGAGCTTCAGGTTTGGGGAGGAGACAAC
AAC------TCCATCTCAGAAGCAGG---------------AAYCGAGRGACCAGGAGCT
ATA---TCCTTTAGCCTCCCTCAAATCACTCTTTGGCAACGACCCCTCGTCACAATAAAG
ATAGGRGGGCAAKTAARGGARGCTCTATTAGATACAGGAGCAGATGATACAGTATTAGAA
GAMATGAATTTRCCAGGAAGATGGAGACCAAAAATGATAGGGGGAATTGGAGGTTTTATC
AAAGTAAAACAGTATGATCAGATACCCATAGAAATYTGTGGACACAAGGCTGTAGGTACA
GTATTAATAGGACCYACACCTRTCAACATAATTGGGAGAAAYYTGTTGACTCAACTTGGT
TGYACTTTAAATTTTCCTATTAGTCCTATTGAAACTGTACCAGTAAAATTAAAGCCAGGA
ATGGATGGCCCAAAAGTTAARCAATGGCCATTRACAGAAGAAAAAATAAAAGCMTTAGTA
GAAATTTGTACAGAAATGGAAAAGGAAGGRAAAATTTCAAAAATTGGGCCTGAAAATCCA
TACAATACTCCAGTATTTGCCATAAAGAAAAAAGACAGTACTAAATGGAGAAAATTAGTA
GATTTCAGGGAACTTAATAARAGAACTCAAGACTTCTGGGAAGTYCAATTAGGRATACCA
CATCCYGCAGGGTTAAAAAAGAAAAAATCTGTAACAGTCCTGGATGTGGGTGATGCATAC
TTCTCAGTYCCTTTAGATAAAGAMTTCAGGAAGTAYACTGCATTTACCATACCTAGTGTA
AACAATGAGACACCRGGGATCAGRTATCAGTACAATGTRCTTCCACAGGGATGGAAAGGA
TCACCAGCRATATTCCAATGTAGTATGACAAAAATCTTAGRGCCTTTTAGARWAMAAAAT
CCAGACATAGTCATCTATCAATACATGGATGATTTGTATGTAGGATCTGACTTAGAAATA
GGGCAGCATAGAGCAAAAATAGARGAACTGAGAGAACATCTGTTGAGGTGGGGATTTACC
ACACCAGACAAAAARCATCAGAAAGAACCTCCATTCCTYTGGATGGGTTATGAACTCCAT

CCTGATAAATGGACAGTACAGCCTATAGTGCTGCCAGAAAAGGACAGCTGGACTGTCAAT
GACATACAGAAGTTAGTGGGAAAGTTRAATTGGGCAAGTCAAATTTATCCAGGRATTAAA
RTAARGGAATTATGTAAACTCATTAGGGGAACCAAAGCACTAACAGAAGTAATACCACTA
ACAGAAGAAGCAGAGC--

>11
---------GAGCCAAACAGCCCCACCAGAGGAGAGCTTCAGGTTTGGGGAGGAARCAAC
AAC------TCCATCTCAGAAGCAGG---------------AGCCGATAGACAAGGAACT
ATA---TCCTTTAGCCTCCCTCAAATCACTCTTTGGCAACGACCCCTCGTCACAATAAAG
ATAGGGGGGCAATTAAAGGARGCTCTATTAGATACAGGAGCAGATGATACAGTATTAGAA
GACATGAAYTTGCCAGGAAGATGGAAACCAAAAATGATAGGGGGAATTGGAGGTTTYATC
AAAGTAAAACAGTATGATCAAATACCCATAGAAATCTGCGGACACAAGGTTGAAGGTACA
GTGTTAATAGGACCTACRCCTGTCAACATAATTGGGA-----------------------
----CTTTAAATTTTCCTATTAGTCCTATTGAAACTGTACCAGTAAAATTAAAGCCAGGA
ATGGATGGCCCAAAAGTTAAACAATGGCCATTGACAGAAGAAAAAATAAAAGCATTAGTA
GAAATTTGTACAGAAATGGAAAAGGAAGGGAAAATTTCAAAAATTGGACCTGAAAATCCA
TACAATACTCCAGTATTTGCCATAAAGAAAAAGGACAGTACTAGGTGGAGAAAATTAGTA
GATTTCAGGGAACTTAATAAACGAACTCAAGACTTCTGGGAAGTTCAATTAGGAATACCA
CATCCTGCAGGGTTAAAAAAGAACAAATCCGTAACAATCCTGGATGTGGGTGATGCATAT
TTCTCAGTCCCTTTAGATAAAGACTTCAGGAAGTATACTGCATTTACCATACCTAGCATA
AAYAATGAGACACCAGGSATCAGATATCAGTACAATGTGCTTCCACAGGGATGGAAAGGC
TCACCAGCAATATTCCAAAGTAGCATGACAAAAATCTTAGAGCCTTTTAGAAAACAAAAT
CCAGATATAGTGATCTGTCAATACGTGGATGATTTGTAYGTAGGATCTGACTTAGAAATA
GGACAGCATAGAGCAAAAATAAAGGAACTGAGARATCATCTGTTAAGGTGGGGATTTAYC
ACACCAGACGAAAAACATCAGAAAGAACCTCCATTCCGTTGGATGGGTTATGAACTCCAT
CCTGATAAATGGACAGTACAGCCTATAGTGCTGCCAGAAAAGGACAGCTGGACTGTCAAT
GATATACAGAAGCTAGTGGGAAAGTTAAATTGGGCAAGTCARATTTATGCAGGGATTAAA
GTAAGGGAATTATGTAAACTCCTTAGGGGAACCAAAGCACTAACAGAAGTAATACCACTA
ACAAAAGAAGCA--

>13
---------------------CCCACCAGAGGAGAGCTTCAGGTTTGGGGAAGAGACAAC
AMC------YCCATCTCAGAAGCAGG---------------AGCCGATAGACAAGGAACT
ATA---TCCTTTAGTYTCCCTCAAATCACTCTTTGGCAACGACCCCTCGTCACAATAAGG
ATAGGGGGRCAAYTAAAGGAAGCTCTATTAGATACAGGAGCAGATGATACAGTATTAGAA
GACATGAATTTGCCAGGAAAATGGAAACCRAAAATGATAGGGGGAATTGGAGGTTTTATC
AAAGTAAGACAGTATGAGGAGGTGCCCATAGAAATCTGCGGACACAAGGCTGTAGGTACA
GTATTAATAGGACCTACACCTGTCAACATAATTGGGAGAAATCTGTTGACTCAGCTTGGG
TGYACTTTAAATTTTCCCATTAGTCCTATTGAAACTGTRCCAGTAAAATTAAAGCCAGGA
ATGGATGGCCCAAAAGTTAAACAATGGCCATTGACAGAAGAAAAAATAAAAGCATTAGTA
GAAATTTGTRCAGAAATGGARAAGGAAGGGAAAATTTCAAAAATTGGRCCTGAAAATCCA
TACAATACTCCAGTATTTGCCATAAAGAAAAAAGACAGTACTAAATGGAGAAAATTAGTA
GATTTCAGRGAACTTAATAARAGAACTCAAGACTTYTGGGAAGTTCAATTAGGAATACCA
CATCCCGCAGGGTTAAAAAAGAAAAAATCCGTAACAATCCTGGATGTGGGTGATGCATAY

TTCTCAGTCCCTYTAGATAAAAAYTTCAGGAAGTATACTGCATTTACCATACCTAGYGTA
AACAATGARACMCCAGGGATCAGRTAYCAGTACAATGTGCTTCCACAGGGATGGAAAGGA
TCACCAGCAATATTCCAATGTAGTATGACAAAAATCTTAGAGCCTTTTAGGAAACAAAAT
CCAGAAATAGTTATYTATCAAYACATGGATGATTTGTATGTAGGATCTGACTTAGAAATA
GGGCAGCATAGAGCRAAAATAGAGGAACTGAGACAWCATTTRTTGAGCTGGGGATTCACC
ACACCAGACAAAAAACATCAGAAAGAACCTCCATTYCTTTGGATGGGTTATGAACTCCAT
CCTGATAAATGGACAGTACAGCCTATAGTGYTGCCAGAAAARGACAGCTGGACTGYCAAT
GACATACAGAAGTTAGTGGGAAAGYTGAATTGGGCAAGTCARATTTATGCAGGRATTAAR
GTAAGRGAATTATGTAAACTCCTYAGGGGAACCAAAGCACTAACAGAAGTAGTMCCACTA
ACAGARGAAGCAGA--

>14
------------------------------GGAGAGCTTCAGRTTTGGGGARGAGACAAC
ARC------TCCATCTCAGAAGCAGG---------------AGCCGATAGACAAGGAACT
ATA---TCCCTTGACTTCCCTCARATCACTCTTTGGCAACGACCCMTYGTCACARTAAAR
ATAGGGGGGCAATTAAAGGAAGCTCTATTAGATACAGGAGCAGATGATACAGTATTAGAA
GAMATGAATTTGCCAGGAARATGGAAACCAAAAATGATAGGGGGAATTGGAGGTTTTATC
AAAGTAAGACAGTATGATCAGRTATCCATGGAAATCTGTGGACAYAARRYTGTAGGTACA
GTATTARTAGGACCTACACCTGTCAACATAATTGGAAGRAATCTGTTGACTCAGATTGGT
TGCACTTTAAATTTTCCCATTAGTCCTATTGAAACTGTACCAGTAAAATTAAAGCCAGGA
ATGGATGGCCCAAAAGTTAAACAATGGCCATTRACAGAAGAAAAAATAAAAGCATTAGTA
GAAATTTGTACAGAAATGGAAAAGGAAGGRAAAATTTCAAAAATTGGGCCTGAAAATCCA
TACAATACTCCAGTRTTTGCCATAAAGAAAAAAGACAGTACAAAATGGAGAAAATTAGTA
GATTTCAGGGAACTTAATAAAAGAACTCAAGACTTCTGGGAAGTTCAATTAGGAATACCA
CATCCCGCAGGGTTAAAAAAGAAAAAATCAGTAACAGTMCTGGATGTGGGTGATGCATAT
TTCTCAGTTCCTTTAGATGAAGATTTCAGGAAATATACTGCATTTACCATACCTAGTGTA
AACAATGAGACWCCAGGGATCAGRTATCARTACAATGTGCTTCCACAGGGATGGAARGGA
TCACCAGCAATATTCCAATGTAGCATGACAAAAATYTTAGAGCCTTTTAGAAAACAAAAT
CCAGAYATAGTTATYTATCAATACATGGATGATTTGTATGTAGGATCTGAYYTAGAAATA
GRACAGCATAGAGCAAAAATAGAGGAACTGAGACAACATTTGTTRGGGTGGGGVTTTACC
ACACCAGACAAAAAACATCAGAAAGAACCYCCATTYCTYTGGATGGGTTATGAACTCCAT
CCTGATAAATGGACAGTACAGCCTATAGTGCTRCCAGAAAAGGACAGCTGGACTGTCAAT
GAYATACAGAAGTTAGTGGGAAARTTRAATTGGGCAAGTCARATTTATCCAGGGATTARA
GTAARRGAATTATGTAAACTCATTAGGGGAACCAAAGCAYTAACAGAAGTAATACCACTA
ACAGAAGAAGCAGAGCTAGAACTGGCAG--------------------------------

>15
---------------------CCCACCAGAGGAGAGCTTCAGGTTTGGGGAAGAGRCAAC
AAC------TCCATCTCAGAAGCAGG---------------AGCCGATAGACAAGGARCT
ATA---TCCYTTAGCTTCCCTCARATCACTCTTTGGCAACGACCCCTCGTCACAATAAAG
ATAGGGGGGCAATTAAAGGAAGCTYTATTAGATACAGGAGCAGATGATACAGTATTAGAA
GAMATGAATTTRCCAGGRAGATGGAAACCAAAAATGATAGGGGGAATTGGAGGTTTTATC
AAAGTAAGACAGTATGATCAAGTAGCTATAGAAATCTGTGGCCACAAGGCTGTWGGTACA
GTATTAATAGGACCTACACCTGTCAACATAATTGGRAGRAATYTGTTGACTCAGATTGGK

TGCACTTTAAATTTTCCCATTAGTCCTATTGAAACTGTACCWGTAAAGTTAAAGCCAGGA
ATGGATGGCCCAAAAGTTAAACAATGGCCACTGACAGAAGAAAARATAAAAGCATTAGTA
GAAATTTGTACAGAAATGGAAAAAGAGGGGAAAATTTCAAAAATYGGGCCTGAAAATCCA
TACAATACTCCAGTATTTGCCATAAAGAAAAAAGACAGTACTAAATGGAGAAAATTAGTA
GATTTCAGGGAACTTAATAAAAGAACACAGGACTTCTGGGAAGTTCAATTAGGAATACCA
CATCCCGCAGGGTTAAAACAGAAAAAATCAGTAACAGTCCTGGATGTGGGTGATGCATAT
TTYTCAGTCCCTYTAGATAAGGACTTCAGGAAGTATACTGCATTTACCATACCTAGTGTA
AACAATGAGACACCAGGGATCAGATATCAGTACAATGTGCTTCCACAGGGATGGAAAGGA
TCACCAGCAATATTCCAATGTAGCATGACAAAAATCTTAGAGTCTTTTAGAAAACAAAAT
CCAGACATAGTTATCTATCAATACGTGGATGATTTGTATGTAGGATCTGACTTAGAAATA
GGGCAACATAGAGMAAAAATAGAGGAACTGAGACAACATTTGTTGAGGTGGGGATTYACC
ACACCAGACAAAAAACATCAGAAAGAACCTCCATTCCTTTGGATGGGTTATGAACTCCAT
CCTGATAAATGGACAGTACAGCCTATAGTGYTGCCAGAAAAGGACMRCTGGACTGTCAAT
GACATACARAAGTTAGTGGGAAAGTTRAATTGGGCAAGTCAGATTTATGCAGGAATTAAG
RYAARGGAATTATGTAAACTCATTAGGGGAACCAAAGCACTAACAGAAGTARTACCACTA
ACAGAAGAAGCAGAGC--

>16

---TCCTCCTTCAGTTTCCCTCAAATCACTCTTTGGCARCGACCCCTTGTTACAGTAAAA
ATAGGAGGACAGATRAAAGAAGCTYTATTAGATACAGGGGCAGATGATACAGTATTAGAA
GAYATAAATTTGCCAGGAAAGTGGAAACCAAAAATGATAGGGGGAATTGGAGGTTTTATC
AAGGTAAAGCAATATGATCAGRTACTTATAGAAATTTGTGGAAAAAGGGCTATAGGTACA
GTGTTAGTAGGACCTACACCTRTCAACATAATTGGACGAAATATGTTGACTCAGATTGGT
TGTACTTTAAATTTCCCAATTAGTCCTATTGACACTGTACCAGTAACATTAAAGCCAGGA
ATGGATGGACCAAAAGTTAAACAGTGGCCATTAACAGAAGAAAAAATAAAAGCATTAACA
GAAATTTGTAAAGAGATGGAAGAGGAAGGAAAAATCTCAAAAATTGGGCCTGAAAATCCA
TACAATACTCCARTATTTGCTATAAAGAAAAAGGACAGCACCAAATGGAGGAAATTAGTA
GATTTCAGAGARCTCAATAAAAGAACTCAGGACTTTTGGGAAGTTCAATTAGGAATACCG
CATCCAKCAGGTTTRARGAAAAAGAAATCAGTAACAGTRCTRGATGTGGGAGATGCATAT
TTTTCAGTTCCTTTAGATGAAAGCTTTAGAAAGTATACTGCATTTACCATACCTAGTACA
AACAATGAGACACCAGGAATCAGATATCAGTACAATGTGCTGCCACAGGGATGGAAAGGA
TCACCGGCAATATTCCAGAGTAGCATGACAAAGATCTTGGAGCCCTTTAGAACAAAAAAT
CCAGAAATAGTTATCTATCAATACATGGATGACTTGTATGTAGGCTCTGATTTAGAAATA
GGGCAGCACAGAATAAAAATAGAGGAGCTGAGAGCTCATCTATTGAGCTGGGGAYTTACT
ACCCCAGACAAAAAGCATCAGAAGGAACCTCCATTCCTTTGGATGGGATATGAACTCCAT
CCTGACAAATGGACAGTCCAGCCTATAGAACTGCCAGAAAARGACAGCTGGACTGTCAAT
GATATACAGAAATTAGTGGGGAAACTCAATTGGGCAAGTCAAATTTATGCAGGAATTAAG
RTAAAACAACTGTGTAAACTCCTCAGGGGAACTAAAGCACTAACAGACATAGTGCCATTG
ACTGAAGAAGCAGAGCTAGAACTGGCAG--------------------------------

>17
------------------------ACCAGCAGARGACTGGGGGATGGGAGAAGAGRTAAC

CTC---------CCTACCGAAGCAGG---------------AGCAGAAAGACAAGGTCCA
TCGCCCTCCTTCAGTCTCCCTCAAATCACTCTTTGGCAACGACCCCTTGTYACAATAAAA
GTAGGAGGACAGCTGAAAGAAGCTCTATTAGATACAGGAGCAGATGATACAGTATTAGAA
GATATAAATTTGCCAGGAAAATGGAAACCAAAAATGATAGGGGGAATTGGAGGTTTTATC
AAAGTAAGGCAATATGATCAGATACTTATAGAAATTTGTGGAAAAAAGGCTATAGGTACA
GTGTTAGTAGGACCTACACCTGTCAACATAATTGGACGAAATATGTTGACTCAGCTTGGT
TGTACTTTAAATTTTCCAATTAGTCCTATTGACACTGTACCAGTAACATTAAAGCCAGGA
ATGGATGGACCAAAGGTTAAACAGTGGCCATTGACAGAAGAAAAAATAAAAGCATTAACA
GAAATTTGTAGGGAAATGGAAGAGGAAGGAAAAATCTCAAGAATTGGGCCTGAAAATCCA
TATAATACTCCAGTATTTGCTATAAAGAAAAAGGACRGCACCAAATGGAGGAAATTAGTA
GATTTCAGAGAGCTCAATAAAAGAACTCAGGAYTTTTGGGAAGTACAATTAGGAATACCG
CATCCAGCAGGATTAAARAAGAAAAAATCAGTRACAGTACTAGATGTGGGAGATGCATAT
TTTTCAGTCCCTTTAGATGAAARCTTTAGAAAGTATACTGCATTYACCATACCTAGTAKA
AACAATGAGACACCAGGAATCAGATATCAGTACAATGTGCTACCACAGGGATGGAAAGGA
TCTCCGGCAATATTCCAGTGTAGCATGACAAAAATCTTAGAGCCCTTTAGAAAACAAAAT
CCAGAAATGGTTATCTATCAATACATGGATGACTTGTATGTAGGATCTGATTTAGAAATA
GGGCAGCACAGAACAAAAATAGAKGAGCTAAGAGCKCATCTATTRAGCTGGGGATTTACT
ACACCAGACAAAAAGCATCAGAAGGAACCGCCATTTCTKTGGATGGGATATGAACTCCAT
CCGGACAGATGGACAGTCCAGCCTATAGAACTGCCAGAAAAAGACAGCTGGACTGTCAAT
GATATACAGAAATTAGTGGGAAAACTAAATTGGGCAAGTCAAATTTATGCAGGSATTAAG
GTAAGGCAACTGTGTAAACTCCTCAGGGGAGCTAAAGCATTAACAGACGTAGTACCACTG
ACTGAAGAAGCAGAGCT---

>18

---------------------CCCACCAGARGAGAGCTTCAGGTTTGGGGAAGAGACAAC
AAC------TCCATCTCAGAAGCAGG---------------AGMCGATAGACARGGATCT
ATA---TCCTTTARCCTCCCTCAAATCACTCTTTGGCAACGACCCCTAGTCACAATAAAA
ATAGGGGGGCAATTAAAGGAAGCTCTATTAGATACAGGAGCAGATGATACAGTATTAGAA
GACATGAATTTGCCAGGAAGATGGAAACCAAAAATGATAGGGGGAATTGGAGGTTTTATC
AAAGTAARACAGTATGATCAGATACCCATAGAAATCTGCGGACACAAGGTTGTAGGTACA
GTATTAATAGGACCTACACCTGTCAACATAATTGGGAGAAATCTGTTGACTCAGCTTGGK
TGCACMTTAAATTTTCCCATTAGTCCTATTGAAACTGTACCAGTAAAATTAAAGCCAGGA
ATGGATGGCCCAAAAGTTAAACAATGGCCATTGACAGAAGAAAAAATAAAAGCATTAGTA
GAAATTTGTACAGAAATGGAAAAGGAAGGRAAAATYTCAAAAATAGGRCCTGAAAATCCA
TACAATACTCCAGTATTTGCCATAAAGAAAAAAGACAGTACTAARTGGAGAAAATTAGTA
GATTTCAGRGAACTTAATAAAAGAACTCAAGACTTCTGGGAAGTTCAATTAGGAATACCA
CATCCCGCAGGATTAAAAAAGAAAAAATCYGTAACAGTCCTGGATGTTGGYGATGCATAT
TTCTCAGTCCCTTTAGATAAAGAYTTCAGGAAGTATACTGCATTTACCATACCTAGTGTA
AACAATGAGACACCAGGRATTAGATATCAGTACAATGTGCTTCCACAGGGATGGAAAGGA
TCACCMGCAATATTCCAATGTAGCATGACAAAAATCTTAGAGCCTTTTAGAAAACAAAAT
CCAGACATAGTTATCTATCAATACATGGATGATTTGTATGTAGGATCTGACTTAGAAATA
GGGCAGCATAGAGCAAAGATAGARGAACTGAGACAWCATCTGTTGAGSTGGGGATTKACT
ACACCAGACAAAAAACATCARAAAGAACCTCCATTCCTTTGGATGGGTTATGAACTCCAT
CCTGATAAATGGACAGTACAGCCTATAGTRCTGCCAGAAAARGACAGCTGGACTGTCAAT

GACATACAGAAGTTAGTGGGAAASTTGAATTGGGCAAGTCAGATTTATGCAGGGATTAAG
GTAAGGGAATTATGTAAACTCCTYAGGGGAACCAAAGCACTAACAGAAGTAATACCAYTA
ACAGAAGAAGCAGAG--

>19
----------------------CCACCAGAGGAGAGCTTCAGGTTTGGGGAGGAGACAAC
AAC------CCCAGCTCAGAAGCAGG---------------AGCCGATAGACAAAGAGAA
GTA---TCCTTTAGCCTCCCTCAAATCACTCTTTGGCAACGACCCCTCGTCACAATAAAG
ATAGGGGGGCAAYTAAAGGAAGCTCTATTAGATACAGGAGCAGATGAYACAGTATTAGAA
GACATGAATTTGCCAGGAAAATGGAAACCAAAAATGATAGGGGGAATTGGAGGTTTTATY
AAAGTAAGACAATATGAGAATATAACCATWGAAATCTGTGGACACAAGGCTGTAGGTACA
GTATTAATAGGACCTACACCTGTCAACATAATTGGGAGGAACTTGTTGACTCAAATTGGT
TGCACTYTAAATTTTCCCATTAGTCCTATTGAAACTGTMCCAGTAAAATTAAAGCCAGGA
ATGGATGGCCCAAAAGTAAAACAATGGCCATTGACAGAAGAAAAAATAAAAGCATTAGTA
GAAATTTGTACAGAACTGGAAAARGAAGGGAAAATTTCAAAGATAGGGCCTGAAAATCCA
TACAATACTCCAGTATTTGCCATAAAGAAAAAAGACAGTACTAAATGGAGAAAATTAGTA
GATTTCAGGGAACTTAATAAAAGAACTCAAGACTTCTGGGAGGTTCAATTRGGAATACCA
CAYCCCGCAGGRCTACAAAARAAAAAATCTGTAACAGTCCKGGABGTGGGTGATGCATAT
TTCTCAGTCCCTTTAGATAAARACTTCAGGAAGTATACTGCATTTACCATACCTAGTATA
AACAATGAGACACCAGGGATCARATATCAGTACAATGTGCTTCCACAAGGATGGAAAGGA
TCACCAGCAATATTYCAAAGTAGCATGACAAAAATCTTAGAGCCTTTYAGAAARCAAAAT
CCRGAYATGATTATCTATCAATACGTGGATGATTTGTATGTAGCATCTGACTTAGAAATA
GGGCAGCATAGAGAAAAAATAAAGGARYTGAGAGAACATCTGTGGAAGTGGGGATTTTTC
ACACCAGACAAAAAACATCAGAAAGAACCCCCATTCCTTTGGATGGGTTATGAACTCCAT
CCTGATAAATGGACAGTACAGCCTATAGTGCTGCCAGAAAAGGACAGCTGGACTGTCAAT
GAYATACAAAAGTTAGTGGGAAAGTTRAATTGGGCAAGCCAGATTTATGCAGGAATTAAG
GTAAGGGAATTATGTAAACTCATTAGGGGAACC---------------------------

>21
------------------------ACCAGAGGAGAGCTTCAGGTTCGAGGAGACAACCCC
AGC------------TCCGAAGCAGG---------------AACYGAAAGACAGGGAG--
-------CCCTTAACTTCCCTCAGATCACTCTTTGGCAGCGACCCCTTGTCTCAATAARA
GTAGGGGGCCAGATAAAAGAGGCTCTCTTAGACACAGGAGCAGATGATACAGTATTAGAA
GAAATAAATTTGCCAGGAAAATGGAARCCAAAAATGATAGGAGGAATTGGAGGTTTTATC
AARGTRAGACARTATGATCAAATACARATAGAAATTTGTGGRAAAAAGGCTATAGGTACA
GTATTAGTAGGACCCACACCTGTCAACATAATTGGAAGAAATCTGTTGACTCAGCTTGGT
TGYACTTTAAAYTTYCCCATTAGTCCTATTGAAACTGTACCAGTAAAATTAAAGCCAGGA
ATGGATGGCCCAAGGGTTAAACAATGGCCMTTGACAGAAGARAAAATAAAAGCATTAACA
GAAATYTGTRATGAAATGGAGAAGGAAGGAAAAATTACAAAAATTGGGCCTGAAAATCCA
TATAAYACTCCAATATTTGCCATAAAAAAGAAGGACAGTACTAAGTGGAGAAAATTAGTA
GATTTCAGRGAACTCAATAAAAGAACTCAAGATTTYTGGGAAGTTCAATTAGGAATACCA
CACCCAGCAGGGTTARAAAAGAAMAAATCAGTGACAGTACTRGATGTGGGGGATGCATAT
TTTTCAGTTCCTTTATATGAAGACTTCAGGAAGTATACTGCATTYACCATACCTAGTATA

AACAATTCGACACCAGGAATYAGATATCAGTATAATGTGCTTCCACAAGGATGGAAAGGA
TCACCAGCAATATTCCAATGTAGCATGACAAAAATCTTAGAGCCYTTTAGAAAACAMAAT
CCAGACMTAGTTATTTATCAATACRTRGATGATTTGTATGTAGSRTCTGACTTAGAAATA
GGGCAACATAGAGCAAAAATAGAGGAGTTAAGAGAACAYCTGTTAAAGTGGGGATTTACC
ACACCAGAYAAGAAACATCAGAAAGAACCTCCATTTCTTTGGATGGGGTATGAGCTCCAT
CCTGACAAATGGACAGTACAGCCTATACAGCTACCAGAAAAGGATAGCTGGACTGTCAAT
GATATACAGAAGTTAGTGGGAAAATTAAACTGGGCAAGTCAGATTTACCCAGGAATTAAA
GTAAGGCAACTTTGTAAACTCCTTAGGGGGGCCARGGCACTAACAGAAATAGTACCACTA
ACTGAAGAAGCAGAGCT--

>22
---------------------CCCACCAGAAGAGAGCTTCAGGTTTGGGGAAGAGACAGC
AAC------TCCACCTCAGAAGCAGG---------------AGCCGATAGACAAGGATCT
ATA---TCCTTTAGCCTCCCTCAAATCACTCTTTGGCAACGACCCCTCGTCACAATAAAG
ATAGGGGGGCAATTAAAGGAAGCTCTATTAGATACAGGAGCAGATGATACAGTATTAGAA
GAAATGAATTTGCCAGGAAGATGGAAACCAAAAATGATAGGGGGAATTGGAGGTTTTATC
AAAGTAAGACAGTATGATCAGATACCAATAGAAATTTGTGGACACAAAACTGAAGGTACA
GTATTAATAGGACCTACACCTGTCAACATAATTGGAAGAAATCTGTTGACTCAGCTTGGT
TGTACTTTAAATTTTCCCATTAGTCCTATTAAAACTGTACCAGTAAAATTAAAGCCAGGA
ATGGATGGCCCAAAAGTTAAACAATGGCCATTGACAGAAGAAAAAATAAAAGCATTAGTA
GAAATTTGTACAGAAATGGAAAAGGAAGGAAAAATTTCAAAAATTGGGCCTGAAAATCCA
TACAATACGCCAGTATTTGCCATAAAGAAAAAAGACAGTACTAAATGGAGAAAATTAGTA
GATTTCAGGGAACTTAATAAAAGAACTCAAGACTTCTGGGAAGTTCAATTAGGAATACCA
CATCCCGCAGGGTTAAAAAAGAAAAAATCTGTAACAGTCCTGGATGTGGGTGATGCATAT
TACTCAGTTCCTTTAGATGAAGACTTCAGGAAGTATACTGCATTTACCATACCTAGTGTA
AACAATGAGACACCAGGGATCAGATATCAGTACAATGTGCTGCCGATGGGATGGAAGGGA
TCACCAGCAATATTCCAATGTAGCATGACAAAAATCTTAGAGCCTTTTAGAAAACAAAAT
CCAGACATAGTTATCTATCAATACATAGATGATTTGCTTGYAGGATCTGACTTAGAAATA
GGGCAGCATAGAGCAAAAATAGAGGAGCTGAGAGAACATTTGTTTAGGTGGGGACTTACC
ACACCAGACAAAAAACATCAGAAAGAACCTCCATTCCTTTGGATGGGTTATGAGCTCCAT
CCTGATAAATGGACAGTACAGCCTATAGTGCTGCCAGAAAAGGACAGCTGRACTGTCAAT
GACCTACAGAAGTTAGTGGGAAAACTGAATTGGGCAAGCCAGATTTATGCAGGAATTAAG
GTAAGGGAATTATGTAAACTCATTAGGGGAACCAAAGCACTAACAGAAGTAATACCACTA
ACAGAGGAAGCAGAGC---

>23
---------------------CCCACCAGAGGAGAGCTTCAGGTTTGGGGARGAGACAAC
AAC------TCCATCTCAGAAACAGG---------------AGCCGATAGACAAGGAAAC
ATA---TCCTTTAGCCTCCCTCAAATCACTCTTTGGCAACGACCCCTTGTCACAATAAAG
ATAGGGGGGCAAYTAAAGGAAGCTCTRTTAGATACAGGAGCAGATGATACAGTATTAGAA
GACATGAATTTGCCAGGAAGATGGAAACCAAAAATGATAGGGGGAATTGGAGGTTTTATC
AAAGTAAGACAGTATGATCAGATACCCATAGAAATCTGTGGACACAARGCTGTAGGTACA
GTATTAATAGGACCTACACCTGTCAACATAATTGGRAGAAATCTGTTGACTCAAMTTGGG
TGYACTTTAAATTTTCCCATTAGTCCTATTGAAACTGTACCAGTAAAATTAAAGCCAGGA

ATGGATGGCCCAAAAGTYAAACAATGGCCATTGACAGAAGARAAAATAAAAGCATTAGTA
GAAATTTGTACAGAAATGGAAAAGGAAGGRAAAATTTCAAAAATCGGGSCTGARAATCCA
TACAATACTCCAGTATTTGCCATAAAGAAAAAAGACAGTACTAARTGGAGAAAGTTAGTM
GATTTCAGRGAACTTAATAAAAGAACTCAAGACTTCTGGGAAGTTCAATTAGGAATACCA
CATCCCGCAGGGTTAAAAAAGAAAAAATCHGTAACAGTYCTGGATGTGGGTGATGCATAY
TTCTCAGTCCCTTTAGATAAAGACTTCAGGAAGTATACTGCATTTACCATACCTAGTGTA
AACAATGAGACACCAGGGAYCAGATAYCAGTACAATGTGCTTCCACAGGGATGGAAAGGA
TCACCAGCAATATTCCAATGTAGCATGACAAAAATYTTAGAGCCTTTYAGAAAACAAAAT
CCAGACATAGTTATCTATCAATACATGGATGATTTGTATGTAGGATCTGACTTAGAAATA
GGGCARCATAGAGCAAAAATAGAGGAACTSAGACAGCATCTGTTRARGTGGGGATTTACC
ACACCAGATAAAAAACATCAGAAAGAACCTCCATTCCTTTGGATGGGGTATGAACTCCAT
CCTGAYAAATGGACAGTRCAGCCTATAGTGCTGCCAGAAAAGGACAGCTGGACTGTCAAT
GACATACAGAAGTTAGTGGGAAAGTTRAATTGGGCAAGYCAGATTTATGCAGGGATYAGG
GTAAAGGAATTATGTAAACTYCTTAGGGGAACCAAAGCACTAACAGAAGTAGTACCACTA
ACAGAAGAAGCAGAGC--

>24
------------------------ACCAGCAGAAGACTGGGGGATGGGGGAGGAGATGAC
CTC---------CTCACTGAAGCAGG---------------AGCAGAAAGAACATCCTCC
------TCCTTCAGTTTCCCTCAAATCACTCTTTGGCAACGACCCCTTGTCACAGTAAAA
ATAGAAGGACAATTRAAAGAAGCTCTATTAGATACAGGAGCAGATGATACAGTATTAGAA
GATATAAATTTGCCAGGAAAATGGAAACCAAAAATGATAGGGGGAATTGGAGGTTTTATC
AAGGTAAGGCAATATGATCAGATACATATAGAAATTTGTGGAAAAAGGGCTATAGGTACA
GTGTTAGTAGGACCTACACCTGTCAACATAATTGGACGAAATATGTTGACTCAGATTGGT
TGTACTTTAAATTTCCCAATAAGTCCTATTGACACTGTACCAGTAAAATTAAAGCCAGGA
ATGGATGGACCAAAGGTTAAACAGTGGCCATTGACAGAAGAAAAAATAAAAGCATTAACA
GAAATTTGTAAAGAGYTGGAAGAGGAAGGAAAAATCTCAAAAATTGGGCCTGAGAATCCA
TACAACACTCCAATATTTGCTATAAAGAAAAAGGACGGTACCAAATGGAGAAAATTAGTA
GATTTCAGAGAGCTCAATAAAAGAACTCAGGACTTTTGGGAAGTTCAATTAGGAATACCA
CATCCAGCAGGYTTAGAAARGAAAAAATCAGTAACCGTAYTAGATGTGGGAGATGCATAT
TTTTCAGTTCCTCTAGATGAAAGCTTTAGAAAGTATACTGCATTCACCATACCTAGTACA
AACAATGAGACACCAGGAATCAGATATCAGTACAATGTGCTACCACAGGGATGGAAAGGA
TCACCAGCAATATTCCAAAGTAGCATGACAAAAATCTTAGAGCCCTTTAGAGCAAAAAAC
CCAGAATTAACTATCTATCAATACGTGGATGACTTATATGTAGCATCTGATTTAGAAATA
GGACAACAYAGAACAAAAATAGAAGAGTTAAGAAAACATCTGTTACAATGGGGATTTTWC
ACACCAGACAAGAAACATCAGAAAGAACCTCCATTTCTTTGGATGGGGTATGAACTCCAT
CCTGACAAATGGACAGTACAGCCTATACAACTGCCAGAAAAAGATAGCTGGACTGTCAAT
GATATACAGAAGTTAGTRGGTAAATTAAACTGGGCAAGTCAGATTTACCCAGGAATTAGA
ATAAAGCAACTTTGTAAACTCATTAGGGGGGCCAAGGCACTAACAGACATAGTACCACTG
ACTGAAGAAGCAGAG--

>25

------------------GAAGCAGG---------------AGACGATAGACAAGGAACT

ATA---TCCTTTAGCCTCCCTCAAATCACTCTTTGGCAACGACCCCTCGTCACAATAAAG
ATAGGGGGGCAATTAAAGGAAGCTCTATTAGATACAGGAGCAGATGATACAGTATTAGAA
GACATAAATTTGCCAGGAAGATGGAAACCAAAAATGATAGGGGGAATTGGAGGTTTTATC
AAAGTAAGACAGTATGAACAGGTACCCCTAGAAATCTGCGGACACAAGGTTGAAGGTACA
GTATTAATAGGACCTACACCTGTCAACATAATTGGAAGAAATCTGTTGACTCAGCTTGGT
TGCACTTTAAATTTTCCCATTAGTCCTATTGAAACTGTACCAGTAAAATTAAAGCCAGGA
ATGGATGGCCCAAAAGTTAAACAATGGCCATTGACAGAGGAAAAATTAAAAGCATTAGTG
GAAATTTGTACAGAAATGGAAAAGGAAGGGAAAATTTCAAAAATCGGGCCTGAAAATCCA
TACAATACTCCAGTATTTGCCATAAAGAGAAAAGACAGTACTAAATGGAGAAAATTAGTA
GATTTCAGGGAACTTAATAAAAGAACTCAAGATTTCTGGGAAGTTCAATTAGGGATACCA
CATCCTGCAGGATTAAAAAAGAGAAAATCTGTAACAGTCCTGGATGTGGGTGATGCATAT
TTCTCAGTCCCTTTAGATGAAGACTTCAGGAAGTATACTGCATTTACCATACCTAGTGTA
AACAATGAGACACCAGGGATCAGATATCAGTACAATGTGCTTCCACAGGGATGGAAAGGA
TCACCAGCAATATTCCAATGTAGCATGACAAAAATCTTAGAACCTTTTAGAAAACAAAAT
CCAGACATAGATATCTGTCAATACATAGATGATTTGTATGTAGGATCTGATTTAGAAATA
GGGCAGCATAGAGCAAAAATAGAGGAACTGAGACAACATTTGTTGAGGTGGGGATTTACC
ACACCAGACAAAAAACATCAGAAAGAACCTCCATTCCTTTGGATGGGTTATGAACTCCAT
CCTGATAAATGGACAGTACAGCCTATAGAGCTGCCAGAAAAGGACAGCTGGACTGTCAAT
GACATACAGAAGTTAGTGGGAAAGTTGAATTGGGCAAGTCAAATTTATCCAGGGATTAGG
GTAAGGGAGTTATGTAAACTCCTTAGGGRAACCAAAGCACTAACAGAAGTAATACCACTA
ACAGAAGAAGCAGAGCT---

>26
------------------AGCCCCACCAGCAGAAGACTGGGGGATGGGAGGAGAGATAAC
CTC---------CTTACCGAAGCAAG---------------AGCAGAGAGACAAGGAACA
------TCCCTTAGTTTCCCTCAAATCACTCTTTGGCAACGACCCCTTGTCACAGTAAAA
RTAGGAGGACAGCTAAAAGAAGCTCTATTAGATACAGGAGCAGATGATACAGTATTAGAA
GATATAAATTTGCCAGGAAAATGGAAACCAAAAATGATAGGGGGAATTGGAGGTTTTATC
AAGGTAAGGCAATATGATCAGATACTTATAGAAATTTGTGGAAAAAAGGCTATAGGTACA
GTATTAGTAGGACCTACACCTGTCAACATAATTGGACGAAATATGTTGACTCAGATTGGT
TGTACTTTAAATTTTCCCATTAGTCCTATTGACACTGTACCAGTAAAATTAAAGCCAGGA
ATGGATGGACCAAAGGTTAAACAGTGGCCATTGACAGAAGAAAAAATAAAAGCATTAACA
GAAATTTGTAMAGAGATGGAAAAGGAAGGAAAAATCTCAAARATTGGGCCTGAAAATCCA
TATAATACTCCAGTATTTGCTATAAAGAAAAAGGACAGCACAAAATGGAGGAAATTAGTA
GATTTCAGAGAACTCAATAAGAGAACTCAAGACTTCTGGGAGGTGCAATTAGGAATACCA
CATCCCGCAGGGTTAAAAAAGAAGARATCAGTAACAGTATTAGATGTGGGTGATGCATAT
TTCTCAATTCCTTTAGATGCAAACTTCAGAAAGTATACTGCATTTACCATACCTAGTATA
AACAATGAAACGCCAGGGATTAGATATCAGTATAATGTGCTTCCACAGGGATGGAAAGGA
TCACCAGCAATATTCCAAAGTAGCATGACAAAAATYYTAGAGCCTTTTAGAAAACAAAAT
CCAGACATAGTGATCTATCAATATATGGATGATTTGTATGTAGGGTCTGATTTAGAAATA
GGACARCATAGGATAAAAATAGAGGAACTGAGACAACATCTGTTGAAGTGGGGATTAACC
ACACCAGACAAAAAACATCAGAAGGAACCTCCATTTCTTTGGATGGGATATGAACTCCAT
CCAGACAGATGGACAGTCCAGCCTATAGAGCTGCCAGAWARAGACAGCTGGACTGTCAAT
GATATACAGAAACTAGTGGGAAAACTAAATTGGGCAAGTCAAATTTATCCAGGGATTAAA

ATAAAGCAATTATGTAAACTCCTTAGGGGAACCAAAGCACTAACAGAAGTAGTACCACTA
ACAGAAGAAGCAGAGCTAGAACTGGCAGA-------------------------------

>27
------------------------ACCAGCAGAGAGCTTCAGGTTCGAGGAGACAACCCC
GGG------------YCCGAAGCAGG---------------AACCGAAAGACAGGGAA--
-------CCCTTAATTTCCCTCAAATCACTCTTTGGCARCGACCCCTTGTCTCAATAAAA
GTAGGGGGTCAAATAAAAGAGGCTCTTTTAGACACAGGAGCAGATGATACAGTATTAGAA
GAAKTAAATTTGCCAGGAAAATGGAAACCAAAAATGATAGGAGGAATTGGAGGTTTTATC
AAAGTAAGACAATATGAGCAAATAACTATAGAAATTTGTGGAAAAAAGGCTATAGGTACA
GTATTAGTGGGACCCACACCTGTTAACATAATTGGAAGGAAYATGTTAACCCAGCTTGGA
TGCACACTAAATTTTCCAATCAGTCCCATTGAAACTGTACCARTAAAATTAAAGCCAGGA
ATGGATGGRCCAAAGGTTAAACAATGGCCATTGACAGAAGAGAAAATAAAAGCATTAACA
GCAATTTGTGATGAAATGGAAAAGGAAGGAAAAATTTCAAGAATTGGGCCTGACAATCCA
TATAACACTCCAATATTTGCCATAAAAAAGAARGACAGTACTAAGTGGAGAAAATTAGTG
GATYTCAGGGAACTCAATAAAAGAACTCAAGATTTTTGGGAAGTTCAATTAGGAATACCA
CACCCAGCAGGGTTARAAAAGAAAAAATCAGTAACAGTTCTAGATGTGGGTGATGCATAT
TTCTCAGTTCCTTTAGATAAAGACTTCAGGAAGTATACTGCATTTACCATACCTAGTGTA
AACAATGAGACACCAGGGATCAGATATCAGTACAATGTGCTCCCACRGGRATGGAAAGGA
TCACCAGCAATATTCCAAWGTAGCATGRCAAAAATCTTGGAGCCTTTTAGAAAACAAAAT
CCAGACATAGTTATTTGTCAATACATGGATGACTTATATGTGGGATCTGACTTAGAAATA
GGGCAGCATAGAACAAAAATAGAGGARCTGAGAGAACATCTGTTAAAGTGGGGATTTACC
ACACCAGACAAGAAACATCAGAAAGAACCTCCATTTCTYTGGATGGGATATGAACTCCAT
CCTGACAAATGGACAGTACAGCCTATACAGCTGCCAGAAAAGGATAGCTGGACTGTCAAT
GAYATACAGAAGTTAGTGGGAAAATTAAACTGGGCRAGTCAGATTTACCCAGGAATTAAA
GTAAGGCAACTGTGTAAACTCCTTAGGGGGGCCAAAGCACTAACAGAAATAGTACCACTA
ACTGAAGAAGCAGAG--

>28
----------------------CCACCAGCAGAGAGTTTCAGGTTCGAGGAGACAACCCC
AGC------------TCCGAAACAGG---------------AACCAAAGGACAGGGAA--
-------CCCTTAACTTCCCTCAAATCACTCTTTGGCAACGACCCGTTGTCGCAATAAGA
GTAGGAGGCCAGATAAAAGAGGCTCTATTAGACACAGGAGCAGATGATACAGTATTAGAA
GAAATAAATTTGCCAGGAAAATGGAAACCAAAAATGATAGGGGGAATTGGAGGATTTATC
AAAGTAAGACAATATGATCAAATACCTATAGAAATTTGTGGAAAAAAGGCTATAGGTACA
GTATTGGTAGGACCTACACCTGTCAACATAATTGGAAGAAATCTGTTGACTCAGCTTGGT
TGCACTTTAAATTTTCCCATTAGTCCTATTGAAACTGTACCAGTAAAGTTAAAGCCAGGA
ATGGATGGCCCAAAAGTTAAACAATGGCCATTGACAGAAGAAAAAATAAAAGCATTAACA
GAAATTTGTATGGAAATGGAAAAGGAGGGAAAAATTTCAAAAATTGGACCTGAAAATCCA
TACAATACTCCAGTATTTGCCATAAAAAAGAAAGACAGTACTAAGTGGAGAAAATTAGTA
GATTTTAGGGAACTCAATAAAAGAACTCAAGATTTTTGGGAAGTCCAATTAGGAATACCA
CACCCGGCAGGGTTAAAAAAGAAAAAATCAGTGACAGTACTGGATGTGGGGGATGCATAC
TTTTCAGTTCCTTTATATGAAGACTTCAGGAAATATACTGCATTCACCATACCTAGTAGA
AACAATGAAACACCAGGGATTAGGTATCAGTACAATGTGCTTCCACAAGGATGGAAAGGA

TCACCAGCAATATTCCAGTGTAGCATGACAAAGATTTTAGAACCTTTTAGAAAACAAAAT
CCAGACATAGTTATCTATCAATACATGGATGATTTGTATGTAGGATCTGACTTAGAAATA
GGGCAACATAGAGCAAAAATAGAAGAGTTAAGAGAACACCTGTTGAAGTGGGGGTTTACC
ACACCAGACAAGAAACATCAGAAAGAACCTCCATTTCTTTGGATGGGGTATGAACTCCAT
CCTGACAAATGGACAGTACAGCCTATACAGCTGCCAGAACGGGATAGCTGGACTGTCAAT
GATATACAGAAGTTAGTGGGAAAATTAAACTGGGCAAGTCAGATATATCCTGGAATTAAA
ATAAGGCAACTTTGTAAACTCCTTAGGGGGGCCAAAGCACTAACAGACATAGTACCACTA
ACTGAAGAAGCAGAGCTAGAACTGGCAGA------------------------------

>32
--------------------CCCACCAGCAGAGAGCTTCAGGTT---CGAGGAGACAAC
CCC---------AACTCTGAAGCAGG---------------AATCGAGAAACAGGGAA--
-------CCCTTAACTTCCCTCAAATCACTCTTTGGCAACGACCCCTTGTCACAGTAAAA
ATAGAAGGACAGCTGAAAGAAGCYCTATTAGATACAGGAGCAGATGATACAGTATTAGAA
GATATAAATTTGCCAGGAAAATGGAAACCAAAAATGATAGGGGGAATTGGAGGTTTTATC
AAGGTAAGGCAATATGATCAGATAACTATGGAAATTTGTGGAAAAAAGGCTATAGGTACA
GTATTAGTAGGACCTACACCTGTCAACATAATTGGACGAAATMTGTTGACTCAGATTGGT
TGTACTYTAAATTTCCCAATTAGTCCTATTGACACTGTACCAGTAAAATTAAAGCCAGGA
ATGGATGGACCAAAGGTTAAACAGTGGCCATTGACAGAAGAAAAAATAARAGCATTAACA
GAAATTTGTAAAGAAATGGAAGAGGAAGGAAAAATCTCAAAAATTGGGCCTGAAAATCCA
TACAATACTCCAGTATTTGCTATAAAGAAAAAGGACAGCACTAAATGGAGAAAATTAGTA
GATTTTAGAGAGCTCAATAAAAGAACTCAGGACTTTTGGGAAGTTCAATTAGGAATACCG
CATCCAGCAGGTTTAAAAAAGAACAAATCAGTGACAGTACTAGATGTGGGAGATGCATAT
TTTTCAGTTCCTTTGGATGAAAGCTTTAGAAAGTATACTGCATTCACCATACCTAGTATA
AACAATGAGACACCAGGAATCAGATATCAGTACAATGTGCTGCCACAGGGATGGAAAGGA
TCACCAGCAATATTCCAGTGTAGCATGACAAAAATCTTACAGCCCTTTAGAGCAAAAAAT
CCAGAAATAGTTATCTATCAATACATGGATGATTTATATGTAGGATCTGATTTAGAAATA
GAGCAGCACAGAATAAAAATAGAGGAGCTAAGAGCTCATCTATTGAGCTGGGGATTTACT
ACACCAGACAAAAAGCATCAGAAGGAACCTCCATTCCTTTGGATGGGATATGAACTCCAT
CCTGACAGATGGACAGTCCAACCTATAGAACTGCCAGAAAARGACAGTTGGACTGTCAAT
GATATACAGAARTTAGTGGGYAAAYTAAACTGGGCAAGTCAGATTTACCCAGGAATTATG
ATAAAGCAACTTTGTAAACTCCTTAGGGGGGCCAAGGCACTAACAGACATAGTACCACTG
ACTCCAGAAGCAGAG--

>33
-----------------------ACCAGCAGAGAGCTTCAGGTTCGAGGAGACAACCCC
AGC------------TCCGAAGCAGG---------------AACCGAAAGACAGGGAA--
-------GCCTTAACCTCCCTCAGATCACTCTTTGGCAGCGACCCCTTGTCACAATAAAA
GTAGGGGGCCAGATAAAAGAGGCTCTATTAGACACYGGAGCAGATGATACAGTATTAGAA
GAAGTAGCTTTGCCAGGAAAATGGAAACCAAAAATGATAGGRGGAATTGGAGGTTTTATC
AAAGTAAGACAATATGATCAAATACCTATAGAAATTTGTGGAAAAAGGGCTATAGGTACA
GTRTTAGTGGGACCCACACCTGTCAACATAATTGGAAGGAATCTGTTGACTCAGCTTGGA
TGCACGCTAAATTTTCCAATCAGTCCCATTGAAACTGTACCAGTAAAATTAAAGCCAGGA
ATGGATGGCCCAAAGGTTAAACAATGGCCATTGACAGAAGAGAAAATAAAAGCATTAACA

GMAATTTGTRATGAAATGGAGAAGGAGGGAAAAATTWCAAARATTGGGCCTGAAAATCCA
TATAACACTCCAATATTTGCCATAAAAAAGAAGGACAGTACTAAGTGGAGAAAGTTAGTA
GATTTCAGGGAACTCAATAAAAGAACTCAAGATTTTTGGGAAGTTCAATTAGGAATACCA
CATCCAGCAGGGTTAAAAAAGAAAAAATCAGTGACAGTACTGGACGTGGGGGATGCATAC
TTTTCAGTCCCTTTATATGAAGACTTCAGGAAATATACTGCATTCACCATACCTAGTATA
AACAATGAAACACCAGGGATTAGGTATCAATATAATGTACTTCCACAGGGATGGAAAGGA
TCACCAGCAATATTCCAGAGTAGCATGACAAAAATCTTRGAACCYTTTAGGGCAAAAAAT
CCAGAAATAGACATCTATCAATATATGGATGAYTTGTATGTAGGATCTGACTTAGAAATA
GGGAAACATAGAGMAAAARTAGAAGAGTTAAGAGAACATCTGTTAAGGTGGGGATTTACC
ACACCAGAYAAGAAACATCAGAAAGAACCRCCATTTCTTTGGATGGGGTATGAACTTCAT
CCTGACAAATGGACAGTACAGCCTATAAAGCTGCCAGAAAAGGATAGCTGGACTGTCAAT
GATATACAGAAGTTAGTGGGAAAATTAAACTGGGCAAGTCAGATTTACCCAGGAATTAAA
GTAAGGCAACTCTGTAAACTCCTTAGGGGGGCCAAAGCACTAACAGACATAGTACCACTA
ACTGAARAAGCARAG--

>34
-----------------------ACCAGCAGAAAATTGGGGGACGGGGGAGGAGACAGC
CTC---------CCTACCGAGGCAGG---------------AGCAGAAAAACAAGGAATA
TCCTTCTCCTGCAATTTCCCTCAAATCACTCTTTGGCAACGACCCCTTGTCACAATAAAA
ATAGGAGGACAGCTGAAAGAAGCTCTACTAGAYACAGGAGCAGATGATACAGTAYTAGAA
GATATAAATTTGCCAGGRAAATGGAAACCAAAAATGATAGGGGGAATTGGAGGTTTTATC
AAAGTAAGACAATATGAGCAAATACCTATAGAAATTTGTGGAAAAAAGGCTATAGGTACA
GTATTAGTGGGACCCACACCTGTCAACATAATTGGAAGAAATATGTTGACCCAGCTTGGA
TGCACACTAAATTTTCCAATCAGTCCCATTGAAACTGTACCAGTAAAATTAAAGCCAGGA
ATGGATGGCCCAAAGGTTAAACAATGGCCATTGACAGAAGAGAAAATAAAAGCATTAACA
GAAATTTGTGAGGAAATGGAGAAGGAAGGAAAAATTACAAARATTGGACCTGACAATCCA
TATAACACTCCAATATTTGCCATAAAAAAGAAGGACGGTACTAAGTGGAGAAAATTAGTA
GATTTCAGGGARCTMAATAAAAGAACTCAAGATTTTTGGGAAATTCAATTAGGAATACCA
CACCCAGCAGGGTTAAAAAAGAAAAAATCAGTAACAGTCCTGGATGTGGGTGATGCATAT
TTCTCAGTTCCTTTAGATAAAGACTTCAGGAAGTATACTGCCTTTACCATACCTAGTGTR
AACAATGAGACACCAGGGATTAGATATCAGTACAATGTGCTTCCACAGGGATGGAAAGGA
TCACCAGCAATATTCCAAAGTAGCATGACAARAATCTTAGAGCCTTTTAGAAAACAAAAT
CCAGACATAGTTATCTATCAATACATGGATGACTTGTATGTAGGATCTGACTTRGAAATA
GGGCAGCACAGAACAAAAATAGAGGAACTGAGAGCTCATCTATTGAGCTGGGGGTTAACT
ACACCAGACAAGAAGCATCAGAAAGARCCTCCATTTCTTTGGATGGGGTATGAACTCCAT
CCTGACAAATGGACAGTACAGCCTATACAGCTGCCAGAAAAGGATAGCTGGACTGTCAAT
GATATACAGAAGTTAGTGGGRAAATTAAACTGGGCAAGTCAAATTTATCCAGGGATTAAG
GTAAAGCAACTGTGTAAACTCCTCAGGGGAACTAAAGCATTAACAGACGTAGTGCCAYTG
ACTGAAGAAGCAGAGCT---

>35
-----------------------------------CTGGGGAATGGGGGAAGARATAGC
CTC---------CTCMCTGAARCAGG---------------AGCAGAAAGACAAGGAACA
YCCTCCTCCTTCAGTTTGCCTCAAATCACTCTTTGGCAACGACCCATTGTCCCAGTAAGA

ATAGGAGGGCAGCTAAAAGAAGCTCTATTAGATACAGGAGCAGATGATACAGTATTAGAA
GACATAGATTTACCAGGAAAATGGAAACCAAAAATGATAGGGGGAATTGGAGGTTTTATC
AAAGTAAAGCAATATGATCAGATACCTATAGAAATTKGTGGAAAAARGGCTATAGGAACA
GTGTTAGTAGGACCTACACCTGTCAACATAATTGGACGAAATATGTTGACTCAGATTGGT
TGTACTTTAAATTTYCCAATTAGTCCTATTGACACTGTACCAGTAAAATTAAAACCAGGA
ATGGATGGACCAAARGTTAAGCARTGGCCATTGACAGAAGAAAAAATAAAAGCATTAAYA
GAAATTTGTAAAGARATGGAAGAGGAAGGAAARATCTCAAAAATTGGRCCTGARAATCCA
TATAATACCCCAGTGTTTGCTATAAAGAAAAAAGACAGCACCAAATGGAGGAAATTAGTA
GATTTCAGAGAGCTCAATAAAAGAACTCAAGACTTTTGGGAAGTTCAATTAGGAATACCR
CATCCAGCAGGTTTAAGAAAGAAAAAATCAGTAACAGTACTAGATGTGGGRGATGCATAC
TTTTCAGTWCCATTAGATAAAGAATTTAGAAAGTATACTGCATTCACCATACCTAGTATA
AACAATGAGACACCAGGAATCAGATATCAATAYAATGTGCTGCCACAGGGATGGAAAGGA
TCACCAGCAATATTCCAGAGTAGCATGACAARAATCTTAGAGCCCTTTAGAATAAAGAAT
CCAGAAATAACTATYTATCAATACATGGATGACTTGTATGTAGGGTCTGATTTAGAAATA
GGACAACATAGAACAAAARTAGAGGAGCTAAGAGCTCATCTATTGAGCTGGGGRTTTACT
ACACCAGACAAAAARCATCAGAAAGAACCTCCATTCCTTTGGATGGGATATGAACTCCAT
CCTGACAAATGGACAGTCCAGCCTATAGAACTGCCAGAAAAAGACAGCTGGACTGTCAAT
GATATACAGAAATTAGTGGGAAAATTAAAYTGGGCAAGTCAAATTTATGCAGGGATTAAR
ATAAAGCAACTGTGTAAACTCATTAGGGGAACTAAAGCACTAACAGACATAGTACCACTA
ACTGAAGAAGCAGAGCTAGAA--------------------------------------

>36
---AGC
ATC---------CTCACTGAAGCAGG---------------AGCAGAAAGACAGGGAACA
CCCTCCTCCTTTAGTTTCCCTCAAATCACTCTTTGGCAACGACCCATTATCCCAGTAAGA
ATAGGAGGRCAGCTAAGAGAAGCTCTATTAGATACAGGAGCAGATGATACAGTATTAGAA
GACATAGATTTACCAGGAAAATGGAAACCAAAAATGATAGGGGGAATTGGAGGTTTTATC
AAAGTAARGCAATATGATCAGATACCTATAGAAATYKGTGGWAAAAAGGCTATAGGAACA
GTGTTAGTAGGACCTACACCTGTCAACATAATTGGACGAAATATGTTGACTCAGATTGGT
TGTACTTTAAATTTCCCAATTAGTCCTATTGACACTGTACCAGTAAAATTAAAACCAGGA
ATGGATGGRCCAAAGGTTAARCAGTGGCCATTGACAGAAGARAAAATAAAAGCATTAACA
GAAATTTGTAAAGAGATGGAAGARGAAGGAAAAATYTCAAAAATTGGGCCTGAAAATCCA
TACAATACTCCAGTGTTTGCTATAAAGAAAAAAGACAGCAACAAATGGAGGAAATTAGTA
GATTTTAGAGAGCTCAATAAGAGAACTCAAGACTTTTGGGAAGTTCAATTAGGAATACCG
CATCCAGCAGGTTTAAGAAAGAAAAAATCAGTAACAGTACTAGATGTGGGAGATGCATAT
TTTTCARTYCCATTAGATRAAGARTTTAGAAAGTATACTGCATTCACCATACCTAGTATA
AACAATGAGACACCAGGAATCAGATATCAATACAATGTGCTACCACAGGGATGGAAAGGA
TCACCAGCAATATTCCAGAGTAGCATGACAAAAATCTTAGAGCCCTTTAGAATAAAAAAT
CCAGAAATAACTATCTGTCAATACATGGATGACTTGTATGTAGGGTCTGATTTRGAAATA
GGRCAACATAGAACAAAARTRGAGGAGCTRAGAGCTCATCTATTGAGCTGGGGGTTTACT
ACACCAGACAAAAAGCATCARAAGGAACCTCCATTTCTTTGGATGGGATATGAACTCCAT
CCTGACAAATGGACAGTCCAGCCTATAGAACTGCCAGAAAAAGACAGCTGGACTGTCAAT
GATATACAGAAGTTAGTGGGAAAATTAAATTGGGCAAGTCARATTTATGCAGGRATTAAG
GTAAGRCAACTGTGTAAACTCCTCAG---------------------------------

>37
------------------------ACCAGCAGAGAGCTTCAGGTTCGAGGAGACAACCCC
AGC------------TCCGAAGCAGG---------------AGCTGAAAGACAGGGAA--
-------GCCTTAACTTCCCTCAAATCACTCTTTGGCAACGACCCCTTGTCTCAATAAGA
GTAGGGGGCCAGACAAAAGAGGCTCTCTTAGATACAGGAGCAGATGATACAGTRTTAGAA
GACATAGATTTGCCAGGAAGATGGAAACCAAAAATGATAGGGGGAATTGGAGGTTTTATC
AAAGTAAGACAGTATGATCAGATAACTATAGAAATTTGTGGACACAAGGTTACAGGTACA
GTATTAGTAGGACCTACACCTGTCAACATAATTGGAAGAAATTTATTGACTCAGCTTGGT
TGCACTTTAAATTTTCCCATTAGTCCTATTGAAACTGTACCAGTAAAATTAAAGCCAGGA
ATGGATGGCCCAAGGGTTAAACAATGGCCATTGACAGAAGAAAAAATAAAAGCATTAACA
GCAATTTGTGATGAAYTGGAGAAGGAAGGAAAAATTTCAAAAATTGGGCCTGAAAATCCA
TATAACACTCCAGTATTTGCCATAAAAAAGAAGGACAGTACTAAGTGGAGAAAATTAGTA
GATTTCAGGGAACTCAATAAAAGAACTCAAGATTTCTGGGAAGTTCAACTAGGAATACCA
CACCCAGSAGGGTTAAAAAAGAACAAATCAGTGACAGTACTAGATGTGGGGGATGCATAT
TTTTCAGTTCCTTTATATGAAGACTTCAGGAAGTATACTGCATTCACCATACCTAGTATA
AACAATCAAACCCCAGGGATTAGGTATCAATATAATGTGCTCCCACAAGGTTGGAAAGGA
TCACCAGCAATATTTCAGTGTAGCATGACAAAGATCTTAGAGCCCTTTAGGGCACAAAAT
CCAGAACTGGTCATCTATCAATATGTGGATGACTTGTATGTAGGATCTGACTTAGAAATA
GGGCAACATAGAGCAAAAATAGAGRAGTTAAGAGCACATCTGTYAAAATGGGGATTCTAC
ACACCAGACAAGAAACATCAGAAAGAACCCCCATTTCTTTGGATGGGGTATGAACTCCAT
CCTGACAAATGGACAGTACAGCCTATACAGCTGCCAGAAAAGGATAGCTGGACTGTCAAT
GATATACAAAAGTTAGTGGGAAAATTAAACTGGGCAAGTCAGATTTACCCAGGAATTAAA
GTAAGACAACTTTGTAAACTTCTTAGGGGGACCAAGGCACTAACAGACATAGTACCGCTA
ACTGAGGAAGCAGAGCT---

>39
-----------------------CACCAGCAGA-AGCTTCAGGTTTGGGGAGGAGACAAC
AAC------TCCCCCTCAGAAGCAGG---------------AGCCGACAGACAAGGAWCT
GTA---TCCTTTRGCTTCCCTCAAATCACTCTTTGGCAACGACCCATCGTCACAATAAGG
ATAGGGGGGCAACTAAAGGAAGCTCTATTAGACACAGGAGCAGATGATACAGTGTTAGAA
GAAATGAACTTGCCAGGAAGATGGAAACCAAAAATGATAGGGGGAATTGGAGGTTTTATC
AAAGTAAGACAGTATGATCAGGTATCCATAGAAATCTGTGGACATAAAGCTATAGGTACA
GTATTAATAGGACCTACACCTGTCAACATAATTGGAAGAAATCTGYTGACTCAGCTTGGC
TGCACTCTAAATTTYCCCATWAGTCCTATTGAAACTGTACCAGTAAAATTAARGCCAGGA
ATGGATGGCCCAAAAGTTAAACAATGGCCATTGACAGAAGAGAAAATAAAAGCATTAGTA
GAAATTTGTACAGAAATGGAAAAGGAAGGAAAAATTTCAAAAATTGGGCCTGAAAATCCW
TACAATACYCCAGTATTTGCCATAAAGAAAAAGGACAGTACTAAATGGAGAAAATTAGTA
GATTTCAGAGAGCTTAATAAGAGAACTCAAGATTTCTGGGAGGTCCAATTAGGAATACCA
CACCCTGCAGGGTTAAAAAAGAAAAAATCAGTAACAGTACTGGATGTGGGTGATGCATAT
TTTTCAGTCCCCTTATATGAAGACTTCAGGAAGTATACTGCCTTTACCATACCTAGTACA
AACAATGAGACACCAGGRATCAGATATCAGTACAATGTGCTTCCACAGGGATGGAAAGGA
TCACCAGCAATATTCCAAAGTAGCATGACAAAAATCTTAGAGCCTTTTAGAAAACAAAAT

CCAGCACTAGAGATCTGTCAATACGTAGATGACTTGTATGTAGGATCTGACTTAGAAATA
GGGCAACACAGAGCAAAAATAGAGGAACTGAGAGAACATCTGTTAAGATGGGGATTAACC
ACACCAGATAAAAAACATCAGAAAGAACCTCCATTCCTTTGGATGGGGTATGAACTCCAT
CCTGATAAATGGACAGTGCAGCCTATAATGCTGCCAGAAAAGGACAGCTGGACTGTCAAT
GACATACAGAAGTTAATAGGAAAATTAAATTGGGCAAGYCAAATTTATGCAGGGATTAAA
GTAAAACAACTATGTAAACTCCTT------------------------------------

>41

---GAAAGACAAGGACCA
TCACYCTCCTTCAGTTTCCCTCAAATCACTCTTTGGCAGCGACCCCTTGTCACAATAAGA
ATAGGAGGACAGCTGAAAGAAGCTCTATTAGATACAGGAGCAGATGATACAGTATTAGAA
GATATAAATTTGCCAGGAAAATGGAAACCAAAAATGATAGGGGGAATTGGAGGTTTTATC
AAAGTAAGGCAATATGATCAGATACTTATAGAAATTTGYGGGAAAAAGGCTATAGGTACA
GTGTTAGTAGGACCTACACCTGTCAACATAATTGGACGAAATATGTTGACTCAGATTGGT
TGTACTTTAAATTTTCCAATTAGTCCTATTGACACTGTACCAGTAACATTAAAGCCAGGA
ATGGATGGACCAAAGGTTAAACARTGGCCATTGACAGAAGAAAARATAAAAGCATTAACA
GAAATTTGTAGGGAAATGGAAGAGGAAGGAAAAATCTCAAAAATTGGGCCTGAAAATCCA
TATAATACTCCAGTATTTGCTATAAAGAAAAAGGATAGCACCAAATGGAGAAAATTAGTA
GATTTCAGAGAGCTCAATAAAAGAACTCAGGACTTTTGGGAAGTACAATTAGGAATACCG
CATCCAGCGGGATTAAAAAAGAAMAAATCAGTGACAGTACTAGATGTGGGAGATGCATAT
TTTTCAGTCCCTTTAGATGAAAGCTTTAGAAAGTATACTGCATTCRCCATACCTAGTACA
AACAATGAGACACCAGGAATYMGATATCAGTACAATGTGCTGCCACAGGGATGGAAAGGA
TCTCCGGCAATATTCCAGTGTAGCATGACAAAAATCTTAGAGCCCTTTAGAAGRAAAAAT
CCAGAGATGGTTATCTATCAATACATGGATGACTTGTATGTAGGATCTGATTTAGAAATA
GGRCAGCACAGAACAAAAATAGATGAGCTAAGAGCTCATCTATTGAGCTGGGGATTTACT
ACTCCAGACAARAAGCATCAAAARGAACCRCCATWTCTTTGGATGGGATATGAACTCCAT
CCGGACAGATGGACAGTCCAGCCTATAGAACTGCCAGAAAAAGACAGCTGGACTGTCAAT
GATATACAGAAATTAGTGGGAAAACTAAATTGGGCAAGTCAAATTTATGCAGGGATTAAG
GTAAAGCAAYTGTGTAAACTCCTCAGGGGAGCTAAAGCATTAACAGAAGTAGTACCACTG
ACTGAAGAAGCAGAG---

>42
------------GCCAACAGCCCCACCAGAGGAGAGCTTCAGGTTTGGGGAGGAGACAAC
MAC------TCCATCTCAGAAGCAGG---------------AGCCGATAGACARGGAGCT
ATA---TCCTTTAGCCTCCCTCAAATCACTCTTTGGCAACGACCCCTYGTCACAATAAAG
ATAGGGGGGCAATTAAAGGAAGCTYTATTAGATACAGGAGCAGATGATACAGTATTAGAA
GACATAAATTTRCCAGGAAGATGGAAACCAAAAATGATAGGGGGAATTGGAGGTTTTATT
AAAGTAARACAGTATGATCAGATACCCATAGAAATYTGYGGACATAAGGCTGTAGGTACA
GTRTTAATAGGACCTACTCCTGTCAACATAATTGGRAGAAATCTGTTGACTCAGMTTGGT
TGCACTTTAAATTTTCCTATTAGTCCTATTGAAACTGTACCAGTAAAATTGAAGCCAGGA
ATGGATGGCCCAAAAGTTAAACAATGGCCATTGACAGAAGAAAAAYTAAAAGCATTAKTA
GAAATTTGTACAGAAATGGAGAAGGAAGGAAAAATTTCAAAAATTGGGCCTGAAAATCCA

TATAATACTCCAGTATTTGCCATAAAGAAAAAAGACAGTACTAAATGGAGAAAATTAGTA
GATTTCAGGGAACTTAATAAAAGRACTCAAGAYTTCTGGGAAGTTCAATTAGGAATACCA
CATCCCGCAGGGTTGAAAAAGAAAAAATCTGTAACAGTYYTGGATGTGGGTGATGCATAT
TTCTCAGTCCCTCTAGATAAAGAYTTCAGGAAGTAYACTGCATTTACCATACCTAGTRTA
AACAATGAGACACCAGGGATYAGATATCAGTACAAYGTGCTTCCACAGGGATGGAAAGGA
TCACCAGCAATATTCCAATGTAGTATGACAAAAATCTTAGAKYCTTTTAGAAAACAAAAT
CCAGACATAGTTATCTATCAATACATGGATGACTTGTATGTAGGATCTGACTTAGAAATA
GGGCARCATAGAACAAAAATAGAGGAACTGAGACAACATCTGTTGAGATGGGGGTTWACC
ACACCAGACAAAAAACATCAGAAAGAACCTCCATTCCTTTGGATGGGGTATGAACTCCAT
CCTGATAAATGGACAGTACAGCCTATAGAGCTGCCAGAAAAGGACAGCTGGACTGTCAAT
GACATACAAAAGTTAGTGGGAAAGTTGAATTGGGCAAGYCAGATTTATGCAGGAATTAAG
GTAAGRGAATTATGCAAACTCCTTAGGGGAACCAAAGCACTAACAGAWGTARTACCACTA
ACARAWGAAGCAGAGC---

>44
-----------------------CACCAGCAGAAGACTGGGGGATGGGAGAACAGATAAC
CTC---------CTTACCGGGGCAGG---------------AGCAGAAAGACAAGGACCA
TCACCCTCCTTCAGTTTCCCTCAAATCACTCTTTGGCAGCGACCCCTTGTCACAATAAAA
ATAGGGGGACAGTTGAAAGAAGCTCTATTAGATACAGGAGCAGATGATACAGTATTAGAA
GATATAAATTTGCCAGGAAAATGGAAACCAAAAATGATAGGGGGAATTGGAGGTTTTATC
AARGTAAGGCAATATGATCAGATACTTATAGAAATTTGTGGGAAAAAGGCTATAGGTACA
GTGTTAGTAGGACCTACACCTGTCAACATAATTGGACGAAATATGTTGACTCAGATTGGT
TGTACTTTAAATTTTCCAATTAGTCCTATTGACACTGTACCAGTAACATTAAAGCCAGGA
ATGGATGGACCAAAGGTTAAACAGTGGCCATTGACAGAAGAAAAAATAAAAGCATTAACA
GAAATTTGTAGGGAAATGGAAAAGGAAGGGAAAATCTCAAAAATTGGGCCTGAAAATCCA
TATAATACTCCAGTATTTGCTATAAAGAAAAAGRATAGCACCAAATGGAGGAAATTAGTA
GATTTCAGAGAGCTCAATAAAAGAACTCAGGACTTTTGGGAAGTACAATTAGGAATACCG
CATCCAGCAGGATTAAAAAAGAAAAAATCAATGACAGTACTAGATGTGGGAGATGCGTAT
TTTTCAGTCCCTTTACATGAAAGCTTTAGAAAGTATACTGCATTCACCMTACCTAGTACA
AACAATGAGACACCAGGAATCAGATATCAGTACAATGTGCTACCACAGGGATGGAAAGGA
TCTCCGGCAATATTCCAGTGTAGCATGACAAAAATCTTAGAGCCCTTTAGAAGAAATAAT
CCAGAGATGGATATCTATCAATACGTGGATGACTTGTATGTAGGATCTGATTTAGAAATA
GGGCAGCACAGAACAAAAATAGATGAGCTRAGAGCTCATCTATTGAGCTGGGGATTTACT
ACTCCAGACAAAAAGCATCAAAAAGAACCGCCATTTCTTTGGATGGGATATGAACTCCAT
CCGGACAGATGGACAGTCCAGCCTATAGAACTGCCAGAAAAAGACAGYTGGACTGTCAAT
GATATACAGAAATTGGTGGGAAAACTAAATTGGGCAAGTCAAATTTATCCAGGGATTAAG
GTAAAGCAACTGTGTAAACTCCTCAGGGGAGCTAAAGCATTAACAGAAGTAGTACCACTG
ACTGAAGAAGCAGA---

>45
--CCCACCAGAGCCAA-CAGCCCCACCAGCGGAGAGCTTCAGGTTTGGGGAAGAGACAAT
AGC------TCCATCTCAGAAGCAGG---------------AGCCGATGGACAAGGAACT
ATA---TCCTTTAGCCTCCCTCAAATCACTCTTTGGCAACGACCCCTCGTCACAATAAGG
ATAGGGGGGCAATTAAAGGAAGCTCTATTAGATACAGGAGCAGATGATACAGTGTTAGAA

GAAATGAATTTGCCAGGAAGATGGAAACCAAAAATGATAGGGGGAATTGGAGGTTTTATC
AAAGTAAGACAGTATGATCAGGTACCCATAGAAATTTGTGGACACAAGGCTGTAGGTACA
GTATTAATAGGACCCACACCTGTCAACATAATTGGGAGAAATCTGTTGACTCAGCTTGGT
TGCACTTTAAATTTTCCTATTAGTCCTATTGRRACTGTACCAGTAAAATTAAAGCCAGGA
ATGGATGGCCCAAGAGTTAAACAATGGCCATTAACAGAAGAAAAAATAAAAGCATTAGTA
GAAATTTGTACAGAAATGGAAAAGGAAGGGAAAATTTCAAAAATAGGGCCTGAAAACCCA
TACAATACTCCAGTATTTGCAATAAAGAAAAAAGACAGTACTAAATGGAGAAAATTAGTA
GATTTCAGGGAACTTAATAAAAGAACTCAAGACTTCTGGGAAGTCCAATTAGGAATACCA
CATCCAGCAGGGYTAAAAAAGAGCAAATCAGTAACAGTCCTGGATGTGGGTGATGCATAT
TTCTCAGTCCCTTTAGATGAAGACTTCAGGAAGTATACTGCATTTACCATACCTAGTGTA
AACAATGAGACACCAGGGATCAGATATCAGTACAATGTGCTTCCACAGGGGTGGAAAGGA
TCACCAGCAATATTCCAAAGTAGYATGACAAAAATCYTAGAGCCTTTTAGAAAACACAAT
CCAGACATAGTTATCTATCAGTACGTGGATGATTTGTATGTAAGCTCTGACTTAGAAATA
GGGCAGCATAGAGCAAAAATAGAAGAGCTGAGACAACATCTGTTGRGGTGGGGATTWWMC
ACACCAGACAAAAAACATCAGAAAGAACCTCCATTCCTTTGGATGGGGTATGAACTCCAT
CCTGATAAATGGACAGTCCAGCCTATAGTGCTGCCAGAAAAGGACAGCTGGACTGTCAAT
GACATACAGAAGTTAGTGGGAAAATTGAATTGGGCAAGTCAAATTTATGCAGGGATTAAG
GTAARGGAATTATGCAAACTCATTAGGGGAACCAAAGCACTAACAGAAGTAATACCACTC
ACAAAAGAAGCAGAG---

>46
-----------------------CACCAGCAGAAAACTGGGGGATGGGGGAAGAGACAAC
CTC---------CYCACTGAAGCAGG---------------AGCAGAAAGACAAGGACCA
TCCTCCTCCTTCAGTTTCCCTCAAATCACTCTTTGGCAACGACCCCTTGTTACAGTAAAA
ATAGGAGGACAGATRAAAGAAGCTCTATTAGATACAGGGGCAGATGATACAGTATTAGAA
GATATAAATTTGCCAGGAAAGTGGARACCAAAAATGATAGGGGGAATTGGAGGTTTTATC
AAGGTAAARCAATATGATCAGATACTTATAGAAATTTGTGGAAAAAAGGCTATAGGTACA
GTGTTAGTAGGACCYACACCTRTCAACATAATTGGACGAAATATGTTGACTCAGATTGGT
TGTACTTTAAATTTCCCAATTAGTYMTATTGACACTGTACCAGTAAMATTAAAGCCAGGA
ATGGATGGACCAAAAGTTAAACARTGGCCATTRACAGAAGAAAAAATAAAAGCATTAACA
GAAATTTGTAAAGAGATGGAAGAGGAAGGAAAAATYTCAAAAATTGGGCCTGAAAATCCA
TACAATACTCCAGTATTTGCTATAAAGAAAAAGGACAGCACCAAATGGAGRAAATTAGTA
GATTTCAGAGAGCTCAATAAAAGAACTCAGGACTTTTGGGAAGTTCAATTAGGAATACCG
CATCCAGCAGGTTTGAAGAAAAAGAAATCAGTAACAGTACTAGATGTGGGAGATGCATAT
TTTTCAGTTCCTTTAGATGAAAGCTTTAGAAAGTATACTGCATTTACCATACCTAGTACA
AAYAATGAGACACCAGGAATCAGATATCAGTACAATGTGCTGCCACAGGGATGGAAAGGA
TCACCRGCAATATTCCAGAGTAGCATGACAAAGATCTTAGAGCCCTTTAGAATAAAAAAT
CCAGAAATAGTTATCTATCAATACATGGATGACTTGTATGTAGGCTCTGATTTAGAAATA
GGGCAGCACAGAATAAAAATAGAGGAGCTGAGAGCTCAYCTATTGAGCTGGGGACTYACT
ACCCCAGACAAAAAGCATCAGAAGGAACCTCCATTCCTTTGGATGGGATATGAACTCCAT
CCTGACAAATGGACAGTCCAGCCTATAGAACTGCCAGAAAAAGACAGCTGGACTGTCAAT
GATATACAGAAATTAGTGGGGAAACTCAATTGGGCAAGTCAAATTTATGCAGGAATTAAG
GTAAAACAACTGTGTAAACTCCTCAGGGGAACTAAAGCACTAACAGACATAGTRCCATTR
ACTGA--

>47

-------------------AAGCAGG---------------AGCAGAAAGACAAGGAACA
CCCTSCTCCCTTAGTTTCCCTCAAATCACTCTTTGGCAACGACCCCTTGTCACAGTAAAA
GTAGGAGGACAGCTGAAAGAGGCTCTATTAGATACAGGAGCAGATGATACAGTATTAGAA
GAGATAAATTTGCCAGGGAAATGGAAACCAAAAATGATAGGGGGAATTGGAGGTTTTATT
AARGTAAGGCAATATGATCAGATAYYYMTAGAAATTTGTGGAAAAAAGGCTATAGGTACA
GTGTTAGTAGGACCTACACCTGTCAACATAATTGGACGAAATATGTTGACTCAGCTTGGT
TGTACTTTAAATTTCCCAATTAGTCCTATTGAMACTGTACCAGTAAMATTAAARCCAGGA
ATGGATGGACCAARRGTTAAACAGTGGCCRTTRACAGAAGAAAARATAAARGCATTAACA
GAAATTTGTAARGAGATGGAARAGGAAGGAAARATYTCMAAAATTGGGCCTGAAAATCCA
TACAATACTCCARTATTTGCTATAAAGAAAAAGGACAGCAMYAAATGGAGRAAATTAGTA
GATTTCAGAGAGCTCAATAAAAGAACTCARGACTTTTGGGAAGTTCAATTAGGAATACCG
CATCCAGCAGGGTTAAAAAAGAASAAATCAGTAACAGTACTAGATGTGGGAGATGCATAY
TTYTCAGTTCCTTTRGATGAAAGCTTCAGRAAGTATACTGCATTYACCATACCTAGTATA
AACAATGAGACACCAGGAATCAGATATCARTATAATGTGCTGCCACAGGGATGGAAAGGR
TCACCAGCAATATTCCAGAGTAGCATGACAAAAATCTTAGAGCCCTTTAGRMTAAAAAAY
CCAGAARTAGTTATCTRYCAATACRTGGATGACTTGTATGTAGGATCTGATTTAGAAATA
GGGCAGCACAGAACAAAAGTAGAGGARCTRAGAGCTCATYTAYTGAGCTGGGGGCTTACT
ACACCAGACAAAAAGYATCAGAAGGAACCTCCATTYCKTTGGATGGGATATGAACTCCAT
CCTGACAAATGGACAGTCCAGCCTATAGAACTGCCAGAAAARGACAGCTGGACTGTCAAT
GATATACAGAAATTAGTGGGAAAACTAAATTGGGCAAGTCAAATCTATCCAGGGATCAAG
GTAAAGCAACTRTGTAAACTCCTCAGRGGRRCTAAAGCACTAACAGAGGTAGTACCACTG
ACTGARGAAGCAGAG---

>48
----------------------CCACCAGCAGAGGACTGGGGGATGGGAGAAGAGATAAC
CTC---------CTTACCGAAGCAGG---------------AGCAAAAAGACAAGGACCA
TCACCCTCCTTCAGTTTCCCTCAAATCACTCTTTGGCAACGACCCCTCGTCACAATAAAA
ATAGGAGGACAGCTGAARGAAGCTCTATTAGATACAGGAGCAGATGATACAGTGTTAGAA
GATATAAATTTACCAGGAAAATGGAAACCAAAAATGATAGGGGGAATTGGRGGTTTTATC
AAAGTAAGGCAATATGATCAGATACTTATAGAAATTTGTGGAAAAAGGGCTATAGGTACA
GTGTTAGTAGGACCTACACCTGTCAACATAATTGGACGAAATATGTTGACTCAGCTTGGT
TGTACTTTAAATTTTCCAATTAGTCCTATTGACACTGTACCAGTAACATTAAAGCCAGGA
ATGGATGGGCCAAAGGTTAAACAGTGGCCACTGACTGAAGAAAAAATAAAAGCATTAACA
GAAATTTGCAGAGAAATGGAAAAGGAAGGAAAAATCTCAAAAATTGGGCCTGAAAACCCA
TATAATACTCCAGTATTTGCTATAAAGAAAAAGGACAGCACCAAATGGAGGAAATTAGTA
GATTTCAGAGAGCTCAATAAAAGAACTCAGGACTTTTGGGAAGTTCAATTAGGAATACCA
CATCCAGCAGGATTAAAAAAGAAAAAATCAGTGACAGTACTAGATGTGGGRGATGCATAT
TTTTCAGTCCCTTTAGATGAAAGCTTTAGAAAGTATACTGCATTCACCATACCTAGTATA
AACAATGAGACACCAGGAATCAGATATCAGTACAATGTGCTACCACAGGGATGGAAAGGA
TCTCCAGCAATATTCCAGTGTAGCATGACAAAAATCTTAGAGCCCTTTAGAAAACAAAAT
CCAGAGATGGTTATCTATCAATACATGGATGACCTGTATGTAGGATCTGATTTAGAAATA

GGGCAGCACAGAACAAAAATAGATRAGCTAAGAGCTCATCTATTRAGCTGGGGATTTACT
ACACCAGACAAAAAGCATCAGAAGGRACCACCATTTCTTTGGATGGGATATGAACTCCAT
CCGGACARATGGACAGTTCAGCCTATAGAACTGCCAGAAAAAGACAGCTGGACTGTCAAT
GATATACAGAAATTAGTGGGAAAACTAAATTGGGCAAGCCAAATTTATSCAGGGATTARG
GTAAGGCAACTGTGTAAACTCCTCAAGGGAGCTAAAGCATTAACAGACGTAGTACCACTG
ACTGAAGAAGCAGAGCT--

>49
--------------------CCCACCAGCAGAGGATTGGGGGATGGGGGAAGAGATAAC
CTC---------CTCACCAAAGCAGG---------------AGCAGAAAGACAAGGGACA
GCAYCCTCCYGTAGTTTCCCTCAAATCACTCTTTGGCAACGACCCCTTGTCACAATAAAA
ATAGCAGGACAGCTGAGAGAAGCTCTATTAGATACAGGAGCAGATGATACAGTATTAGAA
GATATAAATTTGCCAGGAAAATGGAAGCCAAAAATGATAGGGGGAATTGGAGGTTTTATC
AAGGTCAGGCAATATGATCAGATACTTATAGAAATTTGTGGAAAAAAGGCTATAGGTACA
GTGTTAGTAGGACCTACACCTGTCAACATAATTGGACGAAATATGTTGACTCAGCTTGGT
TGTACTCTAAATTTCCCAATTAGTCCTATTRACACTGTACCAGTAAAATTAAAGCCAGGA
ATGGATGGGCCAARGGTTAAACAATGGCCATTGACAGAAGAAAAAATAAWAGCATTAACA
GAAATTTGTAAAGAAATGGAAGAGGAAGGAAAAATYTCAAGAATTGGGCCTGAAAATCCA
TACAATACTCCAGTATTTGTTATAAAGAAAAAAGACAGCACCAAATGGAGGAAATTAGTA
GACTTCAGAGAGCTCAATAAAAGAACTCAGGACTTTTGGGAAGTTCAGTTAGGAATACCG
CATCCAGCAGGTTTAAAAAAGAAAAAATCAGCAACAGTACTAGATGTAGGAGATGCATAT
TTTTCAGTTCCTTTAGATGAAAGCTTTAGAAAGTATACTGCATTCACCATACCTAGTAGA
AACAATGAGACACCAGGAATTAGATATCAGTACAATGTGCTTCCACAGGGATGGAAAGGA
TCACCAGCAATATTCCAAARTAGCATGACAAAAATCTTAGAGCCTTTTAGAAAACAAAAT
CCAGACATAGAGATCTATCAATACGTGGATGATTTGTATGTAGGATCTGACTTAGAAATA
GGGCAGCATAGAACAAAAATAGAGGAGCTGAGACAACATTTATTGAAGTGGGGACTTACC
ACACCAGACAAGAAACATCAGAAAGAACCCCCGCTCCGTTGGATGGGTTATGAACTCCAT
CCTGATAAATGGACAGTACAGCCTATAGTGCTGCCAGATAAGGATAGCTGGACTGTCAAT
GACATACAAAAGTTAGTGGGAAAATTAAATTGGGCAAGTCAAATTTATGCAGGGATCARG
GTGAAGCAACTGTGCAAGCTCCTCAGGGGAGCTAAGGCGCTAACAGACATAGTGCCACTA
ACTGAAGAAGCAGAGCTAGAACTGGCAGA------------------------------

>50
---------------------CCACCAGCAGAAGACTGGGGGATGGGAGAAGAGATAAC
CCC---------CTTACCGAAGCAGG---------------AGCAAARRGACAAGGACCA
TCCCCCTCCTTCARTTTCCCTCAAATCACTCTTTGGCAACGACCCATTGTCACAGTAAAA
ATAGGAGRACAGCTGAAAGAAGCTCTATTAGATACAGGAGCAGATGATACAGTATYAGAA
GATATAAATTTGCCAGGAAAATGGAAACCAAAAATGATAGGGGGAATTGGAGGTTTTATC
AAAGTAAGGCAATATGATCAGATACTTATAGAAATTTGTGGAAAAAGGGCTATAGGTACA
GTGTTARTAGGACCTACACCTGTCAACATAATTGGACGAAATATGTTGACTCAGATTGGT
TGTACTTTAAAYCTTCCAATTAGTCCTATTGACACTGTACCAGTAACATTAAAGCCAGGA
ATGGATGGACCAAGRGTTAAACAGTGGCCATTGACAGAAGAAAAAATAAAAGCATTAACA
GAAATTTGTRAGGAACTGGAAGAGGAAGGAAAAATCTCAAAAATAGGGCCTGAAAATCCA
TATAATACTCCAGTATTTGCTATAAAGAAAAAGRACRGCACCARATGGAGGAAATTAGTA

GATTTCAGAGAGCTCAATAAAAGAACTCAGGAYTTTTGGGAAGTTCAATTAGGAATACCG
CATCCAGCAGGATTAAAAARGAGAAAATCAATGACAGTACTAGATGTGGGAGATGCATAT
TTTTCAGTTCCTTTAGATGAAAGCTTTAGAAARTAYACTGCATTCACCATACCTAGTATA
AACAATGAGACACCRGGAATCAGATATCAGTACAATGTGCTACCACAGGGATGGAAAGGA
TCTCCGGCAATATTCCAGAGTAGCATGACAAAAATCTTAGAGCCCTTTAGAAAACAAAAT
CCAGAGATAGATATCTATCAATACGTGGATGACTTGTATGTAGGATCTGATTTAGAAATA
GGGCAGCAYAGAACAAAAATAGMTGAGCTRAGAGCTCATCTATTGAGCTGGGGAYTWACT
ACACCAGAYMAAAAGCATCAGAAGGAACCGCCATTTCTTTGGATGGGATATGAACTCCAT
CCGGACAGATGGACAGTCCAGCCTATAGAACTGCCAGAAAARGACAGCTGGACTGTCAAT
GATATACAGAAATTAGTGGGAAAACTAAATTGGGCAAGTCAAATATATGCAGGGATTAAG
GTAAAGCAACTGTGTAAACTCCTCAGGGGAGCTAAAGCATTAACAGATGTAGTACCAYTA
ACTRAAGAAGCAGAGCTAGAACTGGCAGA-----------------------------

>51
--------------------CCCACCAGCAGARAACTGGGGGATGGGGGAAGAGATAAC
TTC---------CCCACTGAAGCAGG---------------AGCAGAARGACAAGGAACA
GCCTCCTCCTTCAATTTCCCTCAAATCACTCTTTGGCAACGACCCSTTGTYRCAGTAAAA
ATAGGRGGACAGCTGAGAGARGCTCTATTAGATACAGGAGCAGATGATACAGTATTAGAA
GAAATAGATTTRCCAGGRAAATGGAAACCAAAAATGATAGGGGGAATTGGAGGTTTTATC
AARGTAAGGCAATATGATCAGRTACTTATAGAAATTTGTGGAAARARRGCTATAGGTACA
GTGTTAGTWGGACCTACACCTGTCAAYATAATTGGRCGAAAYATGTTGACTCAGATTGGT
TGTACTTTAAATTTCCCAATTAGTCCTATTGACACTGTACCAGTAAAATTAAARCCAGGA
ATGGATGGGCCAAAGGTTAAACAGTGGCCMTTRACAGAAGAAAAAATAAAAGCATTAACA
GAAATTTGTAARGAAATGGAAGAGGAAGGAAARATCTCAAAAATTGGGCCTGAAAATCCA
TATAATACTCCARTATTTGCTATAAAGAAAAAGGACAGTACCAAATGGAGGAAATTAGTA
GATTTCAGAGARCTCAATAAAAGRACTCAGGAYTTTTGGGAAGTWCAATTAGGAATACCG
CATCCAGCAGGKYTAAAAAAGAAAAAATCAGTAACAGTACTAGATGTGGGAGATGCATAT
TTTTCAGTKCCATTAGATAAAGACTTTAGAAAGTATACTGCATTCACCATACCTAGTATA
AACAATGAGACACCAGGAATYAGATATCAGTACAATGTGYTGCCACAGGGATGGAAAGGA
TCACCAGCAATATTCCAGWGTAGCATGACAAAAATCTTAGARCCCTTTAGARKAAAAAAT
CCAGAAATAATTATCTATCAATACATGGATGACTTGTATGTAGGATCTGATTTAGAAATA
GGGCAGCACAGAGMAAAAATAGAGGAGCTAAGAGCYCATCTATTGAGCTGGGGATTTACT
ACACCAGACAAAAAGCATCAGAAGGAACCTCCATTCCTTTGGATGGGATATGARCTCCAT
CCYGACAGRTGGACAGTCCAGCCTATARAACTGCCAGAAAARGAYAGCTGGACTGTCAAT
GATATACARAAATTAGTGGGAAAACTAAATTGGGCAAGCCAGATCTATCCAGGRATTAGA
RTAAAGCAACTGTGTAAACTCCTCAGGGGAGCTAAAGCACTAACAGACATAGTACCACT-

>52
--------------------CCCACCAGCAGAGAGCTTCAGGTTCGAGGAGACARACCC
AGC------------TCCGAAGCAGG---------------AMTCGAARGACARGGAA--
-------CCCTTAACTTCCCTCAAATCACTCTTTGGCAGCGACCCCTTGTCTCAATAAAA
GTAGGGGGMCAAATAAAAGAGGCTCTTTTAGACACAGGAGCAGATGATACAGTATTAGAA
GAARTAAATTTGCCAGGAAAATGGAAACCAAAAATGATAGGAGGAATTGGAGGTTTTATT

AAAGTAAGACAATATGATCAAATATCTATAGAAATTTGTGGRAAAAAGGCTATAGGTACA
GTATTAGTGGGACCCACACCTRTCAACATAATTGGAAGGAATATGTTGACTCAGCTYGGA
TGTACACTAAATTTTCCAATTAGTCCCATTGAAACTGTACCAGTAAAATTAAAGCCAGGA
ATGGATGGCCCAAAGGTTAAACAATGGCCATTGACAGAAGAGAAAATAAAAGCATTAACA
GCAATTTGTGATGAAATGGAGAAGGAAGGAAAAATTACAAAAATTGGRCCTGAAAAYCCA
TATAATACTCCAATATTTGCCATAAAAAAGAAGGACAGYACWAAGTGGAGAAAATTAGTR
GATTTCAGGGAACTCAATAAAAGAACYCAAGATTTTTGGGAAGTTCAATTAGGAATACCA
CACCCAGCAGGGTTAAAAAAGAAAAAATCAGTAACAGTAYTGGACGTGGGGGATGCATAT
TTTTCAGTTCCTTTAYATGARGACTTCAGGAAATATACTGCATTCACCATACCTAGTATA
AACAATGAAACACCAGGGATTAGGTATCAATATAATGTGCTTCCACAGGGATGGAAAGGA
TCACCAGCAATATTCCAGYATAGCATGACAAAAATCTTAGAGCCCTTTAGAGCAAAAAAT
CCAGAAWTARTCATCTATCAATATATGGATGACTTGTATGTAGGATCTGATTTAGAAATA
GGGCAACATAGAGTAAAAATAGARGARTTAAGAGRMCATCTRTTAARGTGGGGATTTACC
ACACCAGACAAAAAACATCAGAAAGAACCTCCMTTTCTTTGGATGGGGTATGAACTCCAT
CCTGATAARTGGACAGTACAGCCTATACAGCTGCCAGAAAAGGATAGCTGGACTGTCAAT
GATATACAGAAGTTAGTGGGAAAATTAAACTGGGCAAGTCAGATTTACCCAGGWATTAAR
ATAAGGCAACTTTGTAARCTCCTTAGGGGGGCCAAGGCACTAACAGACATAGT-------

>53
--------------------------------AAACTGGGGGATGGGGGAAGAGATAAC
CTC---------CTTACAGAAGCAGG---------------AGCAGAAAGACARGGAACA
TCCYMCTCCCTTAGTTTCCCTCAAATCACTCTTTGGCAACGACCCMTTGTCACAGTAAAA
ATAGGAGGACAACTGAAAGAAGCTCTTTTAGATACAGGAGCAGATGATACAGTATTAGAA
GATATAAATTTGCCAGGAAAATGGAAACCAAAAATGATAGGGGGAATTGGAGGTTTTATC
AARGTAARACAATATGATCAGATACTTATAGAAATTTGTGGAAAAAAGGCTATAGGTACA
GTATTAGTAGGACCYACACCTGTCAACATAATTGGACGAAATATGTTGACTCAGATTGGT
TGTACTTTAAATTTCCCAATTAGTCCTATTGAAACTGTACCAGTAACATTAAAGCCAGGR
ATGGATGGRCCAAAGGTTAARCAGTGGCCATTGACAGAAGAAAAAATAAAAGCATTAACA
GAAATTTGTAAAGARATGGAAGAGGAAGGAAARATCTCAAAAATTGGGCCTGAAAATCCA
TACAATACCCCAGTATTCGCTATAAAGAAAAAAGACAGCACCAAATGGAGRAAGCTAGTA
GATTTCAGAGAGCTCAATAAAAGAACTCARGAYTTTTGGGAAGTTCAATTAGGAATACCA
CACCCAGCAGGKTTAAAAAAGAAAAAATCARTAACAGTACTAGATGTGGGAGATGCATAT
TTTTCAGTTCCTTTAGATGAAARCTTTAGAAAGTATACTGCATTCACCATACCTAGTATA
AACAATGAGACACCAGGAATCAGATATCAGTACAATGTGCTGCCRCAGGGATGGAAAGGA
TCACCAGCAATATTCCAGTGTAGCATGACAAAAATCTTRGAGCCCTTTAGAATAAARAAT
CCAGAAATAAYTATCTAYCAATATATGGATGATTTRTAYGTAGCATCTGATTTAGAAATA
GGRCAGCATAGARCAAAAATAGARGAGCTRAGAGCTCATCTRTTGAGCTGGGGRTTTACT
ACACCAGACAAAAAGCATCAGAAGGAACCTCCATTCCTTTGGATGGGGTATGARCTCCAT
CCTGACARATGGACAGTCCAGCCTATAGAACTACCAGAAAAAGAYAGYTGGACTGTCAAT
GATATACAGAAATTAGTGGGAAAACTAAATTGGGCAAGTCAAATTTATSCAGGGATTARR
RTAAAACAATTGTGTAAACTCCTCAGGGGAACYAAAGCACTAACAGACATAGTGCCACTG
ACTGAGGAAGCAGAGCT---

>54
----------------------CCACCAGCAGAAGATTGGGGGATGGGGGAGGAGATGAC
CTT---------CTCACCGAAGCAGG---------------AGAAGAAGGACAAGGA---
TCCTCCTCCTTCAGTTTCCCTCAAATCACTCTTTGGCAACGACCCCTTGTCACAGTAAAA
ATAGAAGGACAATTGATAGAAGCTCTATTAGATACAGGAGCAGATGATACAGTATTAGAA
GATATAAATTTGCCAGGGAAATGGAAACCAAGAATGATAGGGGGAATTGGAGGTTTTATC
AAGGTAAAGCAATATGATCAGATACCTATAGAGATTTGTGGAAAAAAGGCTATAGGTACA
GTGTTAGTAGGACCTACACCTGTCAACATAATTGGACGAAATATGTTGACTCAGGTTGGT
TGTACTTTAAATTTCCCAATCAGTCCTATTGACACTGTACCAGTAAAATTAAAGCCAGGA
ATGGATGGACCAAAGGTTAAACAGTGGCCATTGACAGAAGAAAAAATAAAAGCATTAACA
GAAATTTGTAAAGAAATGGAAAAGGAAGGAAAAATVTCAAAAATTGGGCCTGAAAATCCA
TACAATACTCCAGTATTTGCTATAAAGARAAAGGRCAGCACCARATGGAGGAAATTATTA
GATTTCAGAGAGCTTAATAAAAGAACTCAGGACTTCTGGGAAGTTCAATTAGGAATACCG
CATCCAGCAGGGTTAAAAMAGAAAAAATCAGTAACAGTACTAGATGTGGGAGATGCATWT
TTCTCAGTTCCATTGGATAAAGACTTCAGAAAGTATACTGCATTCACCATACCTAGTATA
AACAATGAGACACCAGGAATCAGATATCAGTATAATGTGCTGCCACAGGGATGGAAAGGA
TCACCAGCAATATTCCAGAGTAGCATGACAAAAATCTTAGAGCCCTTTAGAATAAAAAAT
CCAGAAGTAATTATCTATCAATACGTGGATGACCTGTATGTACAATCAGATTTAGAAATA
GGACAGCATAGAACAAAAATAGAGGAGCTAAGAGCTCATCTATTAAGCTGGGGATTCACT
ACACCAGACRAAAAGCATCAGAAGGAACCTCCATTCCTTTGGATGGGATATGAACTCCAT
CCTGACAAATGGACAGTCCAGCCTATAGAACTGCCAGAAAAAGACAGCTGGACTGTCAAT
GATATACAAAAATTAGTGGGAAAACTAAATTGGGCAAGCCAGATTTATGCAGGGATTAAG
GTAAAGCAACTGTGTAAACTCCTCAGGGGAGCTAAAGCATTAACAGACATAGTACCACTG
ACTACAGAAGCAGAG--

>55
-----------------------CACCAGCAGAAAACTGGGGGATGGGGGARGA------
------------------GAAGCAGG---------------AGCAGAAA---------CA
GCCTCCTCCYTCAATTTCCCTCAAATCACTCTTTGGCAACGACCCCTTGTTACAGTAAAA
ATAGGAGGACAGCTAAAAGAAGCTCTATTAGATACAGGAGCAGATGATACAGTGTTAGAG
GRGATAAATTTGCCAGGAAAATGGAAACCAAAAATGATAGGGGGAATTGGAGGTTTTATC
AARGTAAGRCAATATGATCAGATACTTATAGAAATTTGTRGAAAAAAGGCTATAGGTACA
GTATTAGTAGGACCCACACCTGTCAACATAATTGGACGAAATATGTTGACTCAGATTGGT
TGTACTCTAAATTTCCCAATTAGTCCTATTGACACTGTACCAGTAACATTAAAGCCAGGA
ATGGATGGACCAAAGGTTAAGCAATGGCCATTAACYGAAGAAAAAATAAAAGCCTTAACA
GAAATTTGTAATGAGATGGAGAAGGAAGGAAAAATCTCAAAAATTGGGCCTGAAAAYCCA
TACAATACTCCRGTATTTGCTATAAAGAAAAAGGACAGCACTAAATGGAGRAAATTAGTR
GACTTCAGAGAGCTYAATAAAAGAACTCAGGATTTTTGGGAAGTTCAATTAGGAATACCG
CATCCAGCAGGTTTAAAAAAGAAAAAATCAGTAACAGTACTAGATGTGGGAGATGCATAC
TTTTCAGTTCCTTTAGATGAAAGCTTTAGRAAATATACTGCATTTACCATACCTAGTACA
AACAATGAGACACCGGGAATCAGATATCAGTACAATGTGCTGCCACAGGGATGGAARGGA
TCACCAGCAATATTCCAGTGTAGCATGACAAAAATCTTAGAGCCCTTTAGAATAAAAAAT
CCAGAAATAGTTATCTATCAATACATGGATGACTTGTATGTAGGATCTGATTTAGAAATA
GGGCAGCACAGAATAAAAATAGAAGAGCTAAGAAGTCATCTATTGAGCTGGGGATTTACT

ACACCAGATAAAAARCATCAGAARGAACCTCCMTTCCTWTGGATGGGATATGAACTCCAT
CCTGACAGATGGACAGTCCAGCCTGTAGAAYTGCCAGAAAAAGRYAGCTGGACTGTCAAT
GATATACAGAAATTAGTGGGAAAACTAAATTGGGCAAGTCARATTTATGCAGGRATYAAA
GTGAAGCAACTGTGTAAACTCCTCAGGGGAGCTAAAGCATTAACAGACATAGTACCACTG
ACTGAAGAAGCAGAGC--

>57
----------------------CCACCAGCAGAAGACTGGGGGATGGGAGAAGAGATARC
CTC---------CTTACCGAGACAGG---------------AGCAGAAAGACAAGGACCA
YYRCCCTCCCTCAGTCTCCCTCAAATCACTCTTTGGCAACGACCCCTTGTYACAGTAAAR
GTAGGAGGACAGTTGAAAGAAGCTCTRTTAGAYACAGGAGCAGATGATACAGTATTAGAA
GATATARATTTGCCAGGRAARTGGAAACCAAAAATGATAGGGGGAATTGGAGGTTTTATC
AAAGTAAGACAATATGATCAGATACTTATAGAAATTTGTGGAAAAARGGCTATAGGTACA
GTATTAGTAGGACCTACACCTGTCAACATAATTGGACGRAAYATGTTGACTCAAATTGGT
TGTACTTTAAATTTTCCAATWAGTCCTATTGACACTGTACCAGTAAAATTAAAGCCAGGA
ATGGATGGACCAAAGGTTAAACAGTGGCCATTGACAGAAGAAAAAATAAAAGCATTAACA
GAAATTTGTAAGGAAATGGARGAGGAAGGRAAAATTTCAAAAATTGGGCCTGAAAATCCA
TATAATACTCCAGTATTTGCTATAAAGAAAAAGAACAGCACMGAATGGAGGAAATTAGTA
GATTTCAGAGAGCTCAATAAAAGAACTCAGGACTTTTGGGAAGTACAATTAGGAATACCA
CAYCCAGCAGGATTAAAAAAGAGAAAATCAATGACAGTACTAGATGTGGGAGATGCATAT
TTTTCAGTCCCTTTAGATGAAARCTTTAGAAAGTATACTGCATTYACCATACCTAGTATA
AACAATGAGACACCAGGAATCAGGTATCAGTATAATGTGCTACCACAGGGATGGAAAGGA
TCTCCGGCAATATTCCAGTGTAGCATGACAAAAATCTTAGAGCCCTTTAGAAAACAAAAT
CCAGACATGGATATCTATCAATACGTGGATGACTTGTATGTAGGATCTGATTTAGAAATA
GGGCAGCACAGARCAAAAATAGATGAGCTRAGAGCTCATCTATTRAGCTGGGGATTTACT
ACACCAGACAAAAAGCATCAGAAGGARCCRCCATTTCTTTGGATGGGATATGAACTCCAT
CCGGACAGGTGGACAGTCCAGCCTATAGAACTGCCAGAAAAAGACAGCTGGACTGTCAAT
GATATACAGAAATTAGTGGGAAAACTAAATTGGGCAAGTCAAATTTATGCAGGGATTAAA
GTAAAGCAATTGTGTAAACTCCTCAGGGGAGCTAAAGCATTAACAGACGTAGTACCACTG
ACTGAAGAAGCAGAG---

>58
----------------------CCACCAGCAGAGAGCTTCAGGTTTGGGGAGGAGACAGC
AAC------TTCCCCTCAGAAGCAAG---------------AGCAGACAGACAAAGAGCT
GTA---TCCTTTAACTTCCCTCAAATCACTCTTTGGCAACGACCCCTCGTCACAGTAAAG
ATAGGGGTGCAACTAAAGGAAGCTCTATTAGACACAGGAGCAGATGATACAGTGTTAGAA
GAAATGAATTTACCAGGAAGATGGAAACCAAAAATGATAGGGGGAATTGGAGGTTTCATC
AAAGTAAGACAGTATGATCAGATAGCCATAGAAATCTGTGGACATAAAGCTATAGGTACA
GTGTTAATAGGACCTACACCTGTCAACATAATTGGAAGAAATCTATTGACTCAGATTGGC
TGCACTTTAAATTTTCCTATTAGTCCTATTGAAACTGTACCAGTAAAATTAAAGCCAGGA
ATGGATGGCCCAAAAGTTAAACAATGGCCATTGACAGAAGAGAAAATAAAAGCATTAGTA
GAAATTTGTACAGAAATGGAAAAGGAAGGAAAAATTTCAAAAATTGGGCCTGAAAATCCA
TACAATACTCCAGTATTTGCTATAAAGAAGAAAGACAGTACTGAATGGAGAAAATTAGTA
GATTTCAGAGAACTTAATAAGAGAACTCAAGACTTCTGGGAGGTCCAATTAGGAATACCA

CACCCTGCAGGGTTAAAAAAGAGAAAATCAGTAACAATACTGGATGTGGGTGATGCATAT
TTTTCAGTTCCCTTAGATGAAGACTTCAGGAAGTATACTGCCTTTACCATACCTAGTACA
AACAATGAGACACCAGGGATTAGATATCAGTACAATGTGCTTCCACAGGGATGGAAAGGA
TCACCAGCAATATTCCAAAGTAGCATGACAAAAATCTTAGAGCCTTTTAGAAAACAAAAT
CCAGCAATAGAGATCTGTCAATACGTGGATGACTTGTATGTAGGATCTGACTTAGAAATA
GGGCAGCATAGAGCAAAGATACAGGAACTGAGAGAACATCTGTTAAGGTGGGGATTAACC
ACACCAGATAAAAAACATCAGAAAGAACCTCCATTCCTTTGGATGGGGTATGAACTCCAT
CCTGACAATTGGACAGTACAGCCCATAATGCTGCCAGAAAAGGACAGCTGGACTGTCAAT
GACATACAGAAGTTAATAGGAAAGTTAAATTGGGCAAGTCAAATTTATGCAGGGATTAAA
GTAAAACAACTATGTAAACTCCTTAGGGGAACCAAAGCACTAACAGAAGTAGTACCATTA
ACCAAAGAAGCAGAGC--

>59
------------------AGCCCCACCAGCAGAAGACTGGGGGATGGGAGGAGAGATARC
CTC---------CTTACCGAGGCAGG---------------AGAAGGAGGACRAGGAACA
------TCCTTCAGTCTCCCTCAAATCACTCTTTGGCAACGACCCCTTGTCACAATAARG
ATAGGAGGACAGCTAAAAGAAGCTCTATTAGAYACAGGAGCAGATGATACAGTATTAGAA
GATATAAATTTGCCAGGAAAATGGAAACCAAAAATGATAGGGGGAATTGGAGGYTTTATC
AARGTWAGGCAATATGATCAGATACTTATAGAAATTTGTGGAAAAAAGGCTATAGGTACA
GTRTTAGTAGGACCTACACCTGTCAACATAATTGGRCGAAATATGTTGACTCARATTGGT
TGTACTTTAAATTTCCCAATTAGTCCTATTGACACTGTACCAGTAACATTAAAGCCAGGA
ATGGATGGACCAAAGGTTAAACAGTGGCCATTGACAGAAGAAAAAATAAAAGCATTAACA
GAAATTTGTAAAGARATGGAAGAGGAAGGAAAAATCTCAAAAATTGGGCCTGARAATCCA
TATAATACTCCAGTATTTGCTATAAAGAAAAAGGATAGCACCAAATGGAGGAAATTAGTA
GATTTYAGAGAACTTAATAAAAGAACTCAGGACTTTTGGGAAGTTCAATTAGGAATACCA
CATCCAGCAGGATTAAAAAAGAAAAAATCAGTGACAGTACTRGATGTGGGAGATGCATAT
TTTTCAGTTCCTTTAGATGAAARCTTTAGAAAGTACACTGCATTCACCATACCTAGTATA
AACAATGAAACACCAGGAATCAGATATCAGTACAATGTGCTACCACAGGGATGGAAAGGA
TCTCCGGCAATATTCCAGWGTAGCATGACAAAAATMTTAGAGCCCTTYAGAAGAAAAAAT
CCAGAGATRATTATCTATCAATAYATGGATGACTTGTATGTAGGATCWGATTTAGAAATA
GGGCAGCACAGAACAAAAATAGAGGAGCTAAGAGCCCATCTATTGAGCTGGGGATTTACT
ACACCAGACAAAAAGCATCAGAAGGAACCTCCATTTCTTTGGATGGGATATGAACTCCAT
CCGGACAGATGGACAGTCCAGCCTATAGAACTGCCRGAAAAAGACAGCTGGACTGTCAAT
GATATACAGAAATTAGTGGGAAAACTAAATTGGGCAAGTCAAATTTATKCAGGGATTAAG
GTAAAGCAACTGTGTARACTCCTCAGGGGAGCTAAAGCACTAACAGACATAGTACCACTG
ACTGAAGAAGCAGAGCT---

>60
-----------------------CACCAGARGAGAGCTTCAGGTTTGGGGAGGAGACARC
AVC------TCCATCTCAGAAGCAGG---------------AGCCGATAGACAAGGAHCT
ATA---TCCTTTAGCCTCCCTCAAATCACTCTTTGGCAACGACCCCTCGTCACAATAAAG
ATAGGGGGGCAATTAAAGGAAGCTCTATTAGATACAGGAGCAGATGATACAGTWTTAGAA
GAYATGAATTTGCCAGGAAGATGGAAACCAAAAATGATAGGGGGAATTGGRGGTTTTATC
AAAGTAAGACAGTATGATCAGATACCCATAGAAATCTGCGGAYACAARRCTGTAGGAACA

GTATTAATAGGACCTACACCTGTCAACATAATTGGGAGAAATCTGTTGACTCAGCTTGGT
TGCACTTTAAATTTTCCCATWAGTCCTATTGAAACTGTACCAGTAAAATTAAAACCAGGR
ATGGATGGCCCAAAAGTTAAGCAATGGCCATTGACAGAAGAAAAAATAAAAGCCTTAGTA
GAAATTTGTACAGAAATGGAAAAGGAAGGRAAAATTTCAAARATYGGGCCWGAAAATCCA
TACAATACTCCAGTATTTGCCATAAAGAAAAAAGACAGTACTAAATGGAGAAAATTAGTA
GATTTCAGGGAACTTAATAAAAGAACTCAAGACTTCTGGGAAGTTCAATTAGGAATACCA
CATCCTGCAGGGTTAAAAAAGAAMAAATCARTAACAGTCCTGGATGTGGGTGATGCATAT
TTCTCAGTYCCTTTAGATAAAGAATTCAGGAAGTATACTGCATTTACCATACCTAGTGTA
AACAATGAGACACCAGGGATCAGATATCAGTACAATGTRCTTCCACAAGGATGGAAAGGA
TCACCAGCAATATTCCAATGTAGCATGACAAAAATCTTAGAGCCTTTTAGAAAACAAAAT
YCAGAMATRGTTATCTATCAATACATGGATGATTTGTATGTAGGATCTGACTTAGAAATA
ARGCAGCATAGAGCAAAGRTAGAGGAAYTRAGACAACATCTGTTGAGGTGGGGRTTTACC
ACMCCAGACAAAAAACATCAGAAAGAACCTCCATTCCTTTGGATGGGTTATGAACTCCAT
CCRGATAAATGGACAGTACAGCCTATAGTGCTGCCAGAAAARGACAYCTGGACTGTCAAT
GACATACAGAAGTTAGTGGGAAAGYTRAATTGGGCRAGTCAGATTTATGCAGGRATTAAG
GTAAGGGAAYTATGTAAACTCMTTAGGGGAACTAARGCACTAACAGAAGTAATACCACTA
ACAGAAGAAGCAGAGCT--

>61
--------------------CCCACCAGAGGAGAGCTTCAGGTTTGGGGAAGRGACAAC
AAC------TCCATCYCAGAAGCAGG--------------AGCCGAMAGACAAGGAACT
ATA---TCCTTTAGCCTCCCTCAGATCACTCTTTGGCAACGACCCCTCGTCGCAATAAAG
ATAGGGGGGCAATTAAAGGAAGCTCTATTAGATACAGGAGCAGATGATACAGTATTAGAA
GACATGAATTTGCCAGGAAAATGGAAACCAAAAATGATAGGGGGAATTGGAGGKTTTATC
AAAGTAAGACARTATGATCAGATACCYATAGAAATTTGCGGACACAAGGCTGTRGGTACA
GTATTARTAGGACCTACACCTGTCAACATAATTGGRAGAAATCTGTTGACTCAGCTGGGT
TGCACTYTAAATTTTCCTATTAGTCCTATTGAAACTGTACCAGTAAAGTTRAAGCCAGGA
ATGGATGGCCCAAAAGTTAAACAATGGCCATTGACAGAAGAAAAAATAAAAGCATTAGTA
GAAATTTGTACAGAAATGGAAAAGGAAGGGAAAATTTCAAAAATCGGGCCTGAAAAYCCA
TACAATACTCCAGTRTTTGCCATAAAGAAAAAAGACAGTACTAAATGGAGAAAAYTAGTA
GATTTCAGGGAACTTAATAAAMGAACACAAGACTTCTGGGAAGTCCAATTAGGAATACCA
CATCCTGCAGGGTTAAAAAAGAAMAAATCTGTAACAGTYCTGGATGTRGGTGATGCATAT
TTCTCAGTCCCTTTAGATAAAGACTTCAGGAAGTAYACTGCATTTACCATACCTAGTGTA
AACAATGARACACCAGGGATCAGATATCAGTATAATGTGCTTCCACAGGGATGGAAAGGA
TCACCAGCAATATTCCAATGTAGCATGACAAAAATCTTAGAGCCTTTTAGAAAACAAAAT
CCAGACATAGTTATYTATCAATACATGGATGATTTRTATGTAGGATCTGACTTAGAAATA
GGGCAGCATAGAGCAAAAATAGAGGAACTGAGRCAGCATYTGTTGAAGTGGGGATTTACC
ACACCAGAYAAAAARCATCAGAAAGAACCTCCATTCCTTTGGATGGGTTATGAACTCCAT
CCTGATAAATGGACAGTACAGCCTATAGTGCTGCCAGAAAARGACAAYTGGRCTGTCAAT
GACATACAGAAGTTAGTRGGRAARTTGAATTGGGCAAGTCAGATTTATGCAGGGATTAAG
GTAAGRGAATTATGTAAACTMCTTAGGGGRACCAAAGCACTAACAGAAGTAATACCACTA
ACAGAAGAAGCAGAGCT--

>62

---------------------CCCACCAGCAGARAATTGGGGGATGGGGGAGGAGATGAC
CTT---------CTTACCGAAGCAGG---------------AGCAGAAGGACAAGGARCA
TCCTCCTCCTTCAGTTTCCCTCAAATCACTCTTTGGCAACGACCCCTTGTCACAGTAARA
ATAGGAGGACAATTGARGGAAGCTCTATTAGATACAGGAGCAGATGATACAGTATTAGAA
GAYATARAKTTGCCAGGRAAATGGAAACCAAGAATGATAGGGGGAATTGGAGGTTTTATC
AAGGTAAARCAATATGAACAGATACCTATAGAAATTTGTGGAAAAAAGGCTATAGGTACA
GTRTTAGTAGGACCTACACCTGTCAACATAATTGGACGAAATATGTTGACTCAGGTTGGT
TGTACTTTAAATTTCCCAATTAGTCCTATTGACACTGTACCAGTAAAATTAAAGCCAGGA
ATGGATGGACCAAAGGTTAAACAGTGGCCATTGACAGAAGAAAAAATAAAAGCATTAACR
GAAATTTGTAAAGAAATGGAAGAGGAAGGAAAAATCTCAAAAATTGGGCCTGAAAATCCA
TACAATACTCCAGTATTTGCTATAAAGAAAAAGGACAGTAMCAAATGGAGGAAATTAGTA
GATTTCAGAGAGCTTAATAAAAGAACTCAGGACTTCTGGGAAGTTCAATTAGGAATACCG
CATCCAGCAGGTTTAAAARAGAAAAAATCAGTAACAGTACTAGATGTGGGAGATGCATAT
TTCTCAGTTCCATTGGATAAAGAYTTYAGAAARTATACTGCATTCACCATACCTAGTACA
AACAATGAGACACCAGGAATYAGATATCAGTATAATGTGCTGCCRCAGGGATGGAAAGGA
TCACCAGCAATATTCCAGAGTAGCATGACAAAAATCTTAGAGCCCTTTAGAATAAAAAAT
CCAGAATTATTYATCTRTCAATACATGGATGACTTGTATGTAGSATCAGATTTAGAAATA
GGRCAGCATAGAACAAAAATAGAGGAGCTAAGAGCTCATCTATTGAGCTGGGGATTMACT
ACACCAGACAAAAAGCATCAGAAGGAACCYCCATTCCTKTGGATGGGATATGAACTCCAT
CCTGACARATGGACAGTCCAGCCTATAGAACTGCCAGAAAAAGACAGCTGGACTGTCAAT
GATATACAAAAATTAGTGGGAAAACTAAATTGGGCAAGCCAGATTTATGCAGGRATTAAR
ATAAAGCAACTGTGTAAACTCCTCAGGGGAGCTAAAGCATTAACAGAYATAGTRCCAYTG
ACTACAGAAGCAGAGCTAGAA--------------------------------------

>63
----------------------CCACCAGCAGAGAACTGGGGGATGGGGGAAGAGACAAC
CTC---------CTCACTGAAGCAGG---------------AGCAGAAAGACAGGGACCA
TCCTCCTCCTTCAGTTTCCCTCAAATCACTCTTTGGCAACGACCCCTTGTCACAGTAARA
ATAGGAGGACAGGTGAAAGAAGCTCTATTAGATACAGGAGCAGATGATACAGTATTAGAA
GATATAAATTTGCCAGGAAAATGGAAGCCAAAAATGATAGGGGGAATTGGAGGTTTTATC
AAGGTAAAGCAATATGATCAGATACTTATAGAAATTTGTGGAAAAAAGGCTATAGGTACT
GTGTTAGTAGGACCTACACCTGTCAACATAATTGGACGAAATATGTTGACTCAGATTGGT
TGYACTTTAAATTTCCCAATTAGTCCTATTGACACTGTACCAGTAACATTAAAGCCAGGA
ATGGATGGACCAAAAGTTAAACAGTGGCCATTAACAGAAGAAAAAATAAAAGCATTAACA
GAAATTTGTAAAGAGATGGAAGAGGAAGGAAAAATCTCAARAATTGGGCCTGAAAATCCA
TACAATACTCCAGTATTTGYTATAAAGAAAAAGGACAGCACCAAATGGAGGAAATTAGTA
GATTTCAGAGAGCTCAATAAAAGAACTCAGGACTTTTGGGAAGTTCAAYTAGGAATACCG
CATCCAGCAGGTTTAAAGAAAAAGAAATCAGTAACAGTACTAGATGTGGGAGATGCATAT
TTTTCAGTTCCTTTAGATGAAAGCTTTAGAAAGTACACTGCATTTACCATACCTAGTACA
AACAATGAGACACCAGGAATCAGATATCAGTACAATGTGCTGCCACAGGGATGGAAAGGA
TCACCGGCAATATTCCAGAGTAGCATGACAAAGATCTTAGAGCCCTTTAGAATRAAAAAT
CCAGAAATARTTATCTATCAATACGTGGATGACTTGTATGTAAGCTCTGATTTAGAAATA
GGACAACACAGAACAAAAATAGAGGAGCTGAGAGCTCATCTATTGAGCTGGGGACTTACT
ACCCCAGACAAAAAGCATCAGAAGGAACCTCCATTCCTTTGGATGGGATATGAACTCCAT

CCTGACAAATGGACAGTCCAGCCTATAGAACTGCCAGAAAAAGACAGCTGGACTGTCAAT
GATATACAGAAATTAGTGGGGAAACTCAATTGGGCAAGTCAAATTTATGCAGGTATTAAG
ATAAAACAACTGTGTAAACTCCTCAGGGGAACTAAAGCACTAACAGATRTAGTACCACTG
ACTGAAGAAGCAGAGCT---

>64
---------------------CCCACCAGCAGAAGACTGGGGGATGGGAGAAGAGATAGC
TTG---------CKTRCCGAAGCAGG---------------CRCAGAARGACAAGGAATG
------TCCCTCAGTTTCCCTCAAATCACTCTTTGGCAGCGACCCCTTGTCACAGTAAAA
ATAGGGGGACWGCTAAAAGAAGCTCTATTAGATACAGGAGCAGATGATACAGTATTAGAA
GATATAAATTTRCCAGGAAAATGGAAACCAAAAATGATAGGGGGAATTGGAGGTTTTATC
AAGGTAAGGCAATATGATCAGATATCTATAGAAATTTGTGGAAAAAGGGCYRTAGGTACA
GTGTTGGTAGGACCTACACCTGTCAACATAATTGGACGAAATATGTTGACTCAGMTTGGT
TGTACTTTAAATTTCCCAATTAGTCCTATTGACACTGTACCAGTAACATTAAAGCCAGGA
ATGGATGGACCAAAGGTTAAACAGTGGCCATTGACAGAAGAAAAAATAAAAGCATTAACA
GAAATTTGTAAAGARATGGAAGAGGAAGGAAAAATCTCAAAAATWGGGCCTGAAAATCCA
TATAACACTCCAGTATTYGCTATAAAGAAAAAGGACAGCACCAAATGGAGGAAATTAGTA
GATTTCAGAGAGCTCAATAAAAGAACTCAGGACTTTTGGGAAGTTCAATTAGGAATACCG
CATCCAGCAGGATTAAGGAAGAAAAAATCAGTGACAGTACTAGATGTGGGAGATGCATAT
TTTTCAGTTCCTCTAGATGAAAGCTTTAGAAAGTATACTGCATTYACCATACCTAGTACA
AACAATGAGACACCAGGAATCAGGTATCAGTACAATGTGCTACCACAGGGATGGAAAGGA
TCTCCGGCAATATTCCAGTGTAGCATGACAAAAATCTTAGAACCYTTTAGAAGAAAAAAT
CCAGARATAGTTATCTATCAATACATGGATGAYTTGTATGTAGGATCTGATTTAGAAATA
GAGCAGCACAGAACAAAAATAGAGGAGCTAAGAGCTCATCTATTGAGCTGGGGATTTACT
ACACCAGACAAAAAGCATCARAAGGAACCTCCATTTCTTTGGATGGGATATGAACTCCAT
CCGGACAGATGGACAGTCCAGCCTATAGAACTGCCAGAAAAAGACAGCTGGACTGTCAAT
GATATACAGAAATTAGTGGGAAAGCTAAATTGGGCAAGTCAAATTTATGCAGGGATTAAG
GTAAAGCAACTGTGTAAACTCCTCAGRGGAGCTAAAGCACTAACAGACATAGTACCACTG
ACT---

>65
CCCACCAGTAGAACCAACAGCCCCGCCAGCAGAAAACTGGGGAATGGGGGAAGAGACAAC
CTC---------CMCACTGAGACAGG---------------AGCAGAAAGAACAGGGCCA
TCCTCCTCCTTTAGTTTCCCTCAAATCACTCTTTGGCAACGACCCGTTGTCACAGTAAAA
ATAGAAGGACAGATGAGAGAAGCTCTAYTAGATACAGGAGCAGATGATACAGTATTAGAA
GATATAAATTTGCCAGGAAAATGGAAACCAAAAATGATAGGGGGAATTGGAGGTTTTATC
AAGGTAAAGCAATATGATCAGATACTTATAGAAATTTGTGGGAAAAAGGCTATAGGTACA
GTGTTAGTAGGACCTACACCTGTCAACATAATTGGACGAAATATGTTGACTCAGCTTGGT
TGTACTTTAAATTTCCCAATTAGCCAAATTGACACTGTACCAGTAAAATTAAAGCCAGGA
ATGGATGGACCAAAGGTTAAACAGTGGCCATTAACAGAAGAAAAAATAAAAGCATTAACA
GAMATTTGTGCAGAKATGGAAAAGGAAGGAAAAATCTCAAAAATTGGGCCTGAAAATCCA
TACAAYACTCCAGTATTTGCTATAAAGAAAAAGGACAGCACCAAATGGAGAAAATTAGTA
GATTTCAGAGAGCTTAATAAAAGAACTCAGGACTTTTGGGAAGTTCAATTAGGAATACCG
CATCCAGCAGGTTTAAAGAAAAAGAAATCAGTAACAGTACTAGATGTAGGAGATGCATAT

TTTTCAGTTCCTTTAGATGAAAGCTTTAGAAAGTATACTGCATTTACCATACCTAGTACA
AATAATGAGACACCAGGAATCAGATATCAGTACAATGTGCTGCCACAGGGGTGGAAAGGA
TCACCAGCAATATTCCAAAGTAGTATGACAAAGATCTTAGAGCCCTTTAGAACAAAAAAT
CCAGAAATAGTTATCTATCAATACATGGATGACTTGTATGTAGGCTCTGATTTAGAAATA
GGGCAGCACAGAATAAAAATAGAGGAGCTAAGAGCTCATCTATTGAGCTGGGGACTTACT
ACCCCAGACAAAAAGCATCAGAAGGAACCTCCATTCCTTTGGATGGGATATGAACTCCAT
CCTGATAAATGGACAGTMCAGCCTATAGAACTGCCAGAAAGAGACAGCTGGACTGTCAAT
GATATACAGAAATTAGTAGGAAAACTAAATTGGGCAAGTCAAATTTATGCAGGAATTMAG
GTAAAGCAACTGTGTAAACTCCTCAGGGGAACTAAAGCACTAACAGAYATAGTACCACTG
ACTGAAGAAGCAGAGCT--

>67
----------------------------GCCGAGASCTTCGGGTTTGGGGAGGAGACAAC
AAC------TCCCCMTCAGAAGCAGG---------------AGAAGACAGACAAGGAACT
GYA---TCCTTTAGCTTCCCTCARATCACTCTTTGGCAGCGACCCCTCGTCTCAATAAAG
ATAGGGGGGCAACAMAAGGAAGCTTTATTAGATACAGGAGCAGATGATACAGTATTAGAA
GAAATGYATTTACCAGGAAGATGGAAACCAAAAATGATAGGGGGAATTGGAGGTTTTATC
AAAGTAAGACAGTATGATCAGAYACTCRTAGAAATTTGTGGACATAAAGCTATAGGTACA
GTATTAATAGGACCTACACCTGTCAACATAATTGGAAGAAATCTGTTGACTCAGATTGGC
TGCACTTTAAATTTTCCCATTAGTCCTATTGAAACTGTACCAGTGAAATTAAARCCAGGT
ATGGATGGCCCAAAAGTTAAACAGTGGCCATTGACAGAAGAAAAAWTAAAAGCATTAGTA
GAAATTTGTACAGAAATGGAAAAGGAAGGAAAAATTTCAAAAATAGGGCCTGAAAATCCA
TACAATACTCCAGTATTTGCAATAAAGAAAAAAGACAGTACTAAATGGAGAAAATTAGTA
GATTTCAGAGAACTCAATAAAAGAACTCAAGACTTCTGGGAGGTTCAATTAGGAATACCA
CATCCCGCAGGRTTAAAAAAGAAAAAATCAATAACAGTACTGGATGTGGGTGATGCATAT
TTTTCAATTCCCTTAGATGAGGACTTTAGGAAGTATACTGCATTTACCATACCTAGTACA
AACAATGAAACACCAGGGATTAGGTATCAGTACAATGTRCTTCCACAGGGATGGAAAGGA
TCACCARCAATATTCCAAAGTAGCATGACAAAAATCCTAGATCCTTTTAGAAAACAAAAT
CCAGACATAGTGATCTGTCAGTACGTGGATGATTTGTATGTAGSATCTGACTTAGAAATA
GGGCAACATAGAACAAAAGTAGAGGAACTGAGACAACATCTGTTGARGTGGGGATTATTC
ACACCAGACAAAAAAYATCAAAAAGAACCTCCATTCCTTTGGATGGGTTATGAACTCCAT
CCTGATAAATGGACAGTACAACCTATAAYACTGCCAGAAAAGGACAGCTGGACTGTCAAT
GACATACAGAAGTTAATAGGAAAACTGAATTGGGCAAGTCAAATTTATGCAGGGATTAAA
GTAAAGCAATTATGTAAACTCCTTAGGGGARCCAAATCACTAACAGAAGTAGTACCACTA
ACACATGAAGCAGAGCT--

>68
------------------------ACCMGCCGAGAGCTTCGGGTTTGGGGAGGAGACAAC
AAC------TCCCCMTCAGAAGCAGG---------------AGAAGACAGACAAGGAACT
GTA---TCCTTTAGCTTCCCTCARATCACTCTTTGGCAGCGACCCCTCGTCTCAATAAAG
ATAGGGGGGCAACAAAAGGAAGCTTTATTAGATACAGGAGCAGATGATACAGTATTAGAA
GAAATGCATTTACCAGGAAGATGGAAACCAAAAATGATAGGGGGAATTGGAGGTTTTATC
AAAGTAAGACAGTATGATCAGATACTCRTAGAAATTTGTGGACATAAAGCTATAGGTACA
GTATTAATAGGACCTACACCTGTCAACATAATTGGAAGAAATCTGTTGACTCAGATTGGC

TGCACTTTAAATTTTCCCATTAGTCCTATTGAAACTGTACCAGTGAAATTAAAGCCAGGT
ATGGATGGCCCAAAAGTTAAACAGTGGCCATTGACAGAAGAAAAAWTAAAAGCATTAGTA
GAAATTTGTACAGAAATGGAAAAGGAAGGAAAAATTTCAAAAATAGGGCCTGAAAATCCA
TACAATACTCCAGTATTTGCAATAAAGAAAAAAGACAGTACTAAATGGAGAAAATTAGTA
GATTTCAGAGAACTCAATAAAAGRACTCAAGACTTCTGGGAGGTTCAATTAGGAATACCA
CATCCCGCAGGRTTAAAAAAGAAAAAATCAATAACAGTACTGGATGTGGGTGATGCATAT
TTTTCAATTCCCTTAGATGAGGACTTTAGGAAGTATACYGCATTTACCATACCTAGTACA
AACAATGAAACACCAGGGATTAGGTATCAGTACAATGTRCTTCCACAGGGATGGAAAGGA
TCACCARCAATATTCCAAAGTAGCATGACAAAAATCCTAGATCCTTTTAGAAAACAAAAT
CCAGACATAGTGATCTGTCAGTACGTGGATGATTTGTATGTAGCATCTGACTTAGAAATA
GGGCAACATAGAACAAAAGTAGAGGAACTGAGACAACATCTGTTGAAGTGGGGATTATTC
ACACCAGACAAAAAAYATCAAAAAGAACCTCCATTCCTTTGGATGGGTTATGAACTCCAT
CCTGATAAATGGACAGTACAACCTATAAYACTGCCAGAAAAGGACAGCTGGACTGTCAAT
GACATACAGAAGTTRATAGGAAAACTGAATTGGGCAAGTCAAATTTATGCAGGGATTAAA
GTAAAGCAATTATGTAAACTCCTTAGGGGAGCCAAATCACTAACAGAAGTAGTACCACTA
ACACATGAAGCAGAG--

>69
------------CCCACCAGCCCCACCAGAGGAGTGCTTCAGGTTTGGGGAAGAGACAGC
AAC------TCCAKCTCAGAAGCAGGGGCCGATAGACAAGGAGCCGAGAGACAAGGARYT
ATA---TCCCTTAACCTCCCTCAAATCACTCTTTGGCAACGACCCYTTGTCACAATAAAR
ATAGGGGGGCAATTAARGGAAGCCCTATTAGATACAGGAGCAGATGATACAGTATTAGAA
GAAATGAATTTGCCAGGAAGATGGAAACCAAAAWTGATAGGGGGAATTGGAGGTTTTGTC
AAAGTAAGACAGTTTGATCAGGTACCCATAGAAATCTGTGGACACAAAACTGTAGGTACA
GTATTAMTAGGACCTACACCTGYCAACATAATTGGAAGAAATTTGTTRACTCAACTTGGT
TGCACTTTAAATTTTCCCATTAGTCCTATTGAAACTGTACCAGTRAAATTAAAGCCAGGA
ATGGATGGCCCAAAAGTYAAACAATGGCCAYTGACAGAAGAAAAAATAAAAGCATTAGTR
GAAATTTGTACAGAAATGGAAAARGAAGGAAAAATTTCAAAAATAGGGCCTGAAAATCCA
TACAATACTCCAGTATTTGCCATAAAGAAAAARGACRGTACTAAATGGAGAAAATTAGTA
GATTTYAGGGAACTTAAYAAAAGAACTCAAGACTTCTGGGAAGTTCAATTAGGAATACCA
CATCCYGCAGGGTTAAAAAAGAAAAAATCAATAACAGTGCTGGATGTGGGTGATGCATAC
TTCTCAGTCCCTTTAGATAAAGACTTYAGGAAGTACACTGCATTTACCATACCTAGTGTA
AACAATGAGACACCAGGGRTCAGATATCARTACAATGTGCTTCCACAGGGATGGAAAGGA
TCACCAGCAATATTCCAGTGTAGCATGACAAAAATCTTAGAGCCTTTTAGAAAACAAAAT
CCAGACATAGTTATCTATCAATACGTGGATGATTTGCTTGTAGGATCTGACTTAGAAATA
GGGCAGCATAGAGCAAAAATAGAGGAACTGAGACAACATCTGTTGARGTGGGGATTCACC
ACACCAGACAAAAAACATCAGAAAGAACCTCCATTCCTTTGGATGGGTCATGAACTCCAT
CCAGATAAATGGACAGTACAGCCTATMGAGCTGCCAGAAAAGGACAGCTGGACGGTCAAY
GACATACAGAAGTTAGTGGGAAAGTTGAATTGGGCMAGTCAGATTTAYTCAGGGATTAAR
GTAAGGGAATTATGTAAACTTCTTAGGGGAACCAAAGCACTAACAGAAGTAGTACCACTA
ACAGAAGAAGCAGAGCTAGAACTGGCAGA------------------------------

>70
--------------------CCCACCAGCAGAGGACTGGGGGATGGGGGAAGAGAGAAC

CTC---------CTTACCGAAGCAGG---------------AGCAGAAAGACAAGGACCA
TCACCCTCCTTTAGTTTCCCTCAAATCACTCTTTGGCAACGACCCCTTGTCACAATAAAA
ATAGGAGGACAGCTGAAAGAAGCTCTATTAGATACAGGAGCAGATGATACAGTATTAGAA
GATATAAATTTGCCAGGAAAATGGAAACCAAAAATGATAGGGGGAATTGGAGGTTTTATC
AAAGTAAGGCAATATGATCAGATACTTATAGAAATTTGTGGAAAAAAGGCTATAGGTACA
GTATTAGTAGGACCTACACCTGTCAACATAATTGGACGAAATATGTTRACTCAGATTGGT
TGTACTTTAAATTTCCCAATTAGTCCTATTGACACTGTACCAGTAACATTAAAGCCAGGA
ATGGATGGGCCAAAGGTTAAACAGTGGCCATTAACAGAAGAAAAAATAAAAGCATTAACA
GAAATTTGTAGGGAGATGGAAGAGGAAGGAAAAATCTCAAAAATTGGGCCTGAAAATCCA
TATAATACTCCAGTATTTGCTATAAAGAAAAAGGACAGCACCAAATGGAGGAAATTAGTA
GATTTCAGAGAGCTCAATAAAAGAACTCAGGACTTTTGGGAAGTTCAATTAGGAATACCG
CATCCAGCAGGATTAAAAAAGAAAAAATCAGTGACAGTACTAGATGTGGGAGATGCATAT
TTTTCAGTTCCTTTAGATGAAAGCTTTAGAAAATATACTGCATTCACCATACCTAGTAGA
AAYAATGAGACACCAGGAATCAGATATCAGTACAATGTGCTACCACAGGGATGGAAAGGA
TCTCCAGCAATATTCCAGTGTAGCATGACAAAAATCTTAGAGCCCTTTAGAAGCAAAAAT
CCAGAGATAGTTATCTATCAATACATGGATGATTTATATGTAGGATCTGATTTAGAAATA
GGGCAGCACAGAAYAAAARTAGAGGAGCTAAGAGCTCATCTATTGAGCTGGGGATTTACT
ACACCAGACAAAAAGCATCAGAAGGAACCTCCATTTCTTTGGATGGGATATGAACTCCAT
CCGGACAGATGGACAGTCCAGCCTATAGAACTGCCAGAAAAAGACAGCTGGACTGTCAAT
GATATACAGAAATTAGTGGGAAAACTAAATTGGGCAAGTCAAATTTATGCAGGGATTAAA
GTAAAGCAACTGTGTAAACTCCTCAGGGGAACCAAAGCATTAACAGACGTAGTACCACTG
ACTGAAGAAGCAGAGCT--

>72
---------------------CCACCAGCAGAAGACTGGGGGATGGAGGGAGAGATAAC
CTC---------CTTACCGAAGCAGG---------------AGCAGAAAGACAGGGAACA
------TCCCTCAGTCTCCCTCAAATCACTCTTTGGCAACGACCCCTTGTCACAATAAAA
ATAGGGGGACAACTAARAGAAGCTCTATTAGATACAGGAGCAGATGATACAGTATTAGAA
GATATAAATTTGCCAGGAAAATGGAAACCAAAAATGATAGGGGGAATTGGAGGTTTTATC
AARGTAAGGCAATATGATCAGATACTTMTAGAAATTTGTGGAAAAAAGGCAATAGGTACA
GTATTAGTAGGACCTACACCTGTCAACATAATTGGACGAAATATGTTGACTCAAATTGGT
TGTACTTTAAATTTCCCCATTAGTCCTATTGACACTGTACCAGTAAAATTAAAGCCAGGA
ATGGATGGACCAAAGGTTAAACAGTGGCCATTGACAGAAGAAAAAATAAAAGCATTAACA
GAAATTTGTAAAGAGATGGAAGAGGAAGGAAAAATCTCAAAAATTGGGCCTGAAAATCCA
TATAATACTCCAGTATTTGCTATAAAGAAAAAGGACAGCACTAAATGGAGGAAATTAGTA
GATTTCAGAGAGCTCAATAAAAGAACTCAGGACTTTTGGGAAGTTCAATTAGGAATACCG
CATCCAGCAGGATTAAAAAAGAACAAATCAGTGACARTACTAGATGTGGGAGATGCATAT
TTTTCAGTTCCTTTAGATGAGAGCTTTAGAAAGTATACTGCATTCACCATACCTAGTATA
AACAATGAGACACCAGGGATCAGATATCAGTACAATGTGCTACCACAGGGATGGAAAGGA
TCTCCGGCAATATTCCAGTGTAGCATGACAAAAATCTTGGAGCCCTTTAGGAGAAAAAAT
CCAGAGATGGTTATCTATCAATACGTGGATGACTTGTATRTAGSATCTGATTTAGAATTA
GAGCAGCACAGAACAAAAATAGAGGAGCTAAGAGCTCATCTRTTGMGCTGGGGATTTACT
ACACCAGACAAAAAGCATCAGAAGGAACMTCCATTTCKTTGGATGGGATATGAACTCCAT
CCGGACAGATGGACAGTCCAGCCTATAGAACTACCAGAMAAAGACAGCTGGACTGTCAAT

GATATACAGAAATTAGTGGGAAAGCTAAATTGGGCAAGTCAAATTTATGCAGGGATTAAG
RTAAAGCATCTGTGTAAACTMCTCAGGGGAGCTAAAGCACTAACAGAAGTAGTACCACTG
ACTGCAGAAGCAGAGCTAGAACTGGCAGA------------------------------

>74
----------------ACAGCCCCACCAGCAGAAGACTGGGGGATGGGAGAGGAGATAAC
CTC---------CTTGCCGAAGCAGG---------------AGCAGAAAGACAAGGAACC
TCACCCTCCCTCAGTTTCCCTCAAATCACTCTTTGGCAACGACCCCTTGTCACAATAAAA
ATAGGAGGACAGCTRAAAGAAGCTCTATTAGATACAGGAGCAGATGATACAGTATTAGAA
GATATAAATTTGCCAGGAAAGTGGAAACCAAAAATGATAGGGGGAATTGGGGGTTTTATC
AAAGTAAGGCAATACGATCAGATACTTATAGAAATTTGTGGAAAACAGGCTATAGGTACA
GTGTTAGTAGGACCTACACCTGTCAACATAATTGGACGAAATATGTTGACTCAGCTTGGT
TGTACTTTAAATTTTCCAATTAGTCCTATTGAAACTGTACCAGTAACATTAAAGCCAGGA
ATGGATGGACCAAGGGTTAAACAGTGGCCATTGACAGAAGAAAAAATAAAAGCATTAACA
GAAATTTGTAAGGAGATGGAAGCGGAAGGAAAAATCTCAAAAATTGGRCCTGAAAATCCA
TATAATACTCCAGTATTTGCTATAAAGAAAAAGGACAGCACCAAATGGAGGAAATTAGTA
GATTTCAGAGAGCTCAATAAAAGAACTCAGGACTTTTGGGAAGTTCAATTAGGAATACCA
CATCCAGCGGGATTAAAAAAGAAAAAATCAGTGACAGTACTAGATGTGGGAGATGCATAT
TTTTCAGTCCCTTTAGATAAAAACTTTAGAAAGTATACTGCATTCACCATACCTAGTACA
AACAATGAGACACCAGGAATCAGATATCAGTACAATGTGCTACCACAGGGATGGAAAGGA
TCTCCGGCAATATTCCAGTGTAGCATGACAAAAATATTAGAGCCCTTTAGAAGAAAAAAT
CCAGAGATGATTATCTATCAATATGTGGATGACTTGTATGTAGCATCTGATTTAGAAATA
GGGCAGCACAGAACAAAAATAGATGAGCTGAGAGCTCATCTATTGAGCTGGGGATTTACT
ACACCAGACAAAAAGCATCAGAAAGAACCGCCATTTCTTTGGATGGGATATGAACTCCAT
CCGGACAGATGGACAGTCCAGCCTATAGAACTGCCAGAAAAAGACAGCTGGACTGTCAAT
GATATACAGAAATTAGTGGGAAAACTAAATTGGGCAAGTCAAATTTATGCAGGGATTAAG
GTAAAGCAACTGTGTAGACTCCTCAGGGGAGCTAAAGCATTAACAGAAGTAGTACCACTG
ACTAAAGAAGCAGAGCTAGAACTGGCAG-------------------------------

>75
---------------------CCCACCAGCAGAAGACTGGGGGATGGAAGGAGAGATAAC
CTC---------CTYACCGAAGCAGG---------------AGCAGAAAGACAGGGAACA
------TCCCTCAGTCTCCCTCARATCACTCTTTGGCAACGACCCCTTGTCACAATAAAA
ATAGGAGGACAGCTAAGAGAAGCTCTATTAGATACAGGAGCAGATGATACAGTATTAGAA
GATATAAATTTACCAGGAAAATGGAAACCAAAAATGATAGGGGGAATTGGAGGTTTTATC
AAAGTAAGGCAATATGATSAGRTAMCTATAGAMATTTGTGGAAAAARGGCTATAGGTACA
GTGTTAGTAGGACCTACACCTGTCAACATAATTGGACGAAACATGTTGACTCAGCTTGGY
TGYACYTTAAATTTCCCAATTAGTCCTATTGACCCTGTACCAGTAACATTAAAGCCAGGA
ATGGATGGACCAAAGGTTAAACAGTGGCCATTGACAGAAGAAAAAATAAAAGCATTAACA
GAAATTTGTAAAGAGATGGAAGAGGAAGGAAAAATCTCAAAAATTGGGCCTGAAAATCCA
TATAACACTCCAGTATTTGCTATAAAGAAAAAGGATAGCACCAAATGGAGAAAATTAGTA
GATTTCAGAGAGCTCAATAAAAGAACTCAGGACTTTTGGGAAGTTCAATTAGGAATACCR
CATCCAGCAGGATTAAAAAAGAAAAAATCAGTGACAGTAYTAGATGTAGGAGATGCATAY
TTTTCAGTTCCTTTAGATGAAAGCTTTAGRAAGTACACTGCATTCACCATACCTAGTATA

AACAATGAGACMCCAGGAGTCAGATATCAGTACAATGTGCTACCACAAGGATGGAAAGGA
TCTCCGGCAATATTCCAGTRTAGCATGACAAAAATCTTAGAGCCCTTTAGAAGAAAAAAT
CCAGAGATAATTATCTATCAATACATGGATGACTTGTATGTAGGATCTGATTTAGAAATA
GGACAGCAYAGAACAAAAATAGAGGAGCTRAGAGCTCATCTATTGAGCTGGGGATTTACT
ACACCAGACAAAAAGCATCAGAAGGAACCTCCATTTCTTTGGATGGGATATGAACTCCAT
CCGGACAAATGGACAGTCCAGCCTATAGAACTGCCAGAAAAAGATAGCTGGACTGTCAAT
GATATACAGAAATTAGTGGGAAAATTAAATTGGGCAAGTCAAATTTATCCAGGGATTAGG
GTAAAGCAACTGTGTAAACTCCTCAGGGGAGCCAAAGCACTAACAGACATAGTACCACTG
ACTGAAGAAGCAGAGCT--

>76

-----CTCCTTCAGTTTCCCTCAAATCACTCTTTGGCAACGACCYCTYGTCACAATAAAA
ATAGGAGGACAGCTGAAAGAAGCTCTATTAGATACAGGAGCAGATGATACAGTATTAGAA
GATATRAATTTGCCAGGAAAATGGAAACCAAAAAYGATAGGGGGAATTGGAGGTTTTATC
AAAGTAARRCARTATGATCAGATATGYATAGAAATTTGTGGAMAWARRGCTATAGGTACA
GTRTTAGTAGGACCTACACCTGTCAACATAATTGGAMGAAATMTGTTGACTCAGATTGGT
TGYACTTTAAAYTTTCCMATTAGTCCTATTGACACTGTACCAGTRACATTAAARCCAGGA
ATGGATGGACCAARRGTTAAACAGTGGCCATTGACAGAAGAAAAAATAAAAGCATTARYA
GAAATTTGTAMRGAAATGGAARAGGAAGGAAAAATYTCAAAAATTGGGCCTGAAAATCCA
TAYAATACTCCAGTATTTGCTATAAAGAAAAARGACAGYACYAAATGGAGRAAATTAGTA
GATTTCAGAGAGCTCAATAAAAGAACTCAGGACTTTTGGGAAGTTCAATTAGGAATACCA
CATCCAGCAGGATTAAAAAAGAAAAAATCARTRACAGTACTRGATGTGGGWGATGCATAT
TTTTCARTYCCTTTAGATGARRRCTTTAGRAAGTATACTGCATTYACCATACCTAGTAYA
AACAATGAGACACCAGGRATYAGRTATCAGTACAATGTRCTWCCACAGGGATGGAAAGGA
TCTCCDGCRATATTCCARWGTAGCATGACAAAAATCTTAGAKCCYTTTAGAAAACAAAAT
CCAGASATRGTKATCTRTCARTACRTGGATGAYTTGTATGTAGSATCTGAYTTAGAAATA
GGGCAACAYAGAACAAAARTAGAKGARCTRARASMWCATCTRTTGARSTGGGGATTWWYY
ACACCAGACAAAAARCATCARAARGAACCKCCATTYCTTTGGATGGGWTATGAACTCCAT
CCWGAYARATGGACAGTMCARCCTATAGAACTGCCAGAAAARGACAGCTGGACTGTCAAT
GATATACAGAARTTAGTGGGAAAACTRAATTGGGCAAGTCAAATWTATGCAGGRATTAAG
GTAAAGCAACTGTGTAAACTCCTCAGGGGAGCTAAAGCATTAACAGAAGTAGTACCACTG
ACTGAAGAAGCAGAGCTAGAACTGGCAGA------------------------------

>79
--------------------CCCACCAGAAGAGAGCTTCAGGTTTGGGGAGGAGACAGC
AAC------TCCCCCTCAGAAGAAGG---------------AGCCGACAGACAGGGAACT
GTA---TCCTTTAGCTTCCCTCAAATCACTCTTTGGCAACGACCCCTCGTCACAGTAAAA
GTGGGAGGGCAACTAAWGGAAGCTYTATTAGATACAGGAGCAGATGATACAGTATTAGAA
GAAATRAGTTTRCCAGGGAGATGGAAACCAAAAATGATAGGGGGAATTGGAGGTTTTATC
AAAGTAAGACAGTATGATCAGATACTCATAGAAATTTGTGGACATAAGGCCATAGGTACA
GTRTTAATAGGACCTACACCTGTCAACATAATTGGAAGAAATCTGTTGACTCAGATTGGT
TGTACCTTAAATTTTCCCATTAGTCCTATTGAAACTGTACCAGTAAAATTAAAGCCAGGA

ATGGATGGGCCAAAARTTAARCAATGGCCATTAACAGAAGAAAAAATAAAAGCATTAGTA
GAAATTTGTACAGAAATGGAAAAGGAAGGGAAAATTTCAAAAATTGGGCCTGAAAATCCA
TACAATACTCCAGTATTTGCCATAAAGAAAAAGGACAGTACTAAATGGAGAAAATTAGTA
GATTTCAGAGAGCTTAACAAGAGAACACAAGACTTCTGGGAAGTTCAATTAGGAATACCA
CACCCTGCAGGGTTAAAAAAGAAAAAATCAGTGACAGTACTAGATGTGGGTGATGCATAT
TTTTCAGTWCCCTTAGATARAGACTTCAGGAAGTATACTGCATTTACCATACCTAGTACA
AACAATGAGACACCAGGGATTAGATATCAGTACAATGTACTTCCACAGGGATGGAAGGGA
TCACCAGCAATATTCCAAAGTAGCATGACAAAAATCTTAGAGCCTTTTAGAAAACAAAAT
CCAGACATAGTCATTTATCAATACATGGATGATTTGTATGTAGGATCTGACTTAGAAATA
GAAAAGCATAGAACAAAAGTAGAGGAACTGAGACAACACCTGTTAARATGGGGATTTACY
ACACCAGATAAAAARCATCAGAAAGAACCTCCATTCCTCTGGATGGGTTATGAACTCCAT
CCTGATAAATGGACAGTACAGCCTATAAWGCTGCCAGAMAAAGACAGCTGGACTGTTAAT
GATATACAGAAGTTAGTGGGAAAATTAAATTGGGCAAGTCAGATTTATGCAGGAATTAAA
GTAAAGCAATTATGTAAACTTCTTAGGGGAACCAAAGCACTAACAGAAATAATACCACTA
ACAGAAGAAGCAGAGCT--

>80
---------GAGCCAAACAGCCCCACCAGCAGAAAACTGGGGGATGGGGGAAGAGACAAC
CTC---------CTCACTGAAGCAGG---------------AGCAGAAAGACAAGGACCA
CCCTCCTCCCTTAGTTTCCCTCAAATCACTCTTTGGCAACGACCCCTTGTCACAGTAAAA
ATAGGAGGACAGATGAAAGAAGCTCTACTAGATACAGGAGCAGATGATACAGTATTAGAA
GATATAAATTTGCCAGGAAAATGGAAACCAAAAATGATAGGGGGAATTGGAGGTTTTATC
AAAGTAAGGCAATATGATCAGATACTTATAGAAATTTGTGGAAAAARGGCTATAGGTACA
GTGTTAGTAGGACCTACACCTGTCAACATAATTGGACGAAATATGTTGACTCAGATTGGT
TGTACTTTAAATTTCCCAATTAGCCCTATTGACMCTGTACCAGTAMAATTAAAGCCAGGA
ATGGATGGGCCAAAGGTTAAACAATGRCCATTAACAGAAGAAAAAATAAAAGCATTAACA
GAAATTTGTAAAGAAATGGAAGAGGAAGGAAAAATCTCAAAAATTGGGCCTGAAAATCCA
TACAATACTCCAGTATTTGCTATAAAGAAAAAGGACAGCACCAAATGGAGAAAATTAGTA
GATTTCAGAGAGCTCAATAAAAGAACTCAGGACTTTTGGGAGGTTCAATTAGGAATACCG
CATCCAGCAGGCTTAAAGAAAAAGAAATCAGTAACAGTACTGGATGTGGGAGATGCATAT
TTTTCAGTTCCTTTAGATGAAAGCTTTAGAAAGTATACTGCATTTACCATACCTAGCATA
AACAATGAGACACCAGGAATCAGATATCAGTACAATGTGCTGCCACAGGGATGGAAAGGA
TCACCGGCAATATTCCAGAGTAGCATGACAAAAATCTTARAGCCCTTTAGRATAAAAAAT
CCAGAACTAGTTATCTACCAATACATGGATGACTTGTATGTAGGCTCTGATTTAGAAATA
GGGCAGCACAGAATAAAGATAGAGGAGCTAAGAGCTCATCTATTGAGCTGGGGACTTACT
ACCCCAGACAAAAAGCATCAGAAGGAACCTCCATTCCTTTGGATGGGATATGAACTCCAT
CCTGACAGATGGACAGTCCAGCCTATAGAACTGCCAGAAAAAGACAGCTGGACTGTCAAT
GATATACAGAAATTAGTGGGRAAACTAAATTGGGCAAGTCAAATTTATCCAGGAATTCAG
ATAAAGCAACTGTGTAAACTCCTCAGGGGAGCTAARGCACTAACAGACATAGTACCACTG
ACTGAGGAAGCARAGCTAGAACTGGCAGA-------------------------------

>81
---------------------CCCACCAGCAGAGGACTGGGGGATGGGAGAAGAGATAAC
CTC---------CTTACCGAGGCAGG---------------AGCAGAAAGACAAGGACCA

TCACCCTCCTTCAGTTTCCCTCAAATCACTCTTTGGCAACGACCCCTTGTCACAGTAAAA
ATAGGAGGGCAGCTGAAAGAAGCTCTATTAGATACAGGAGCAGATGATACAGTATTAGAA
GATATAAATTTGCCAGGAAAATGGAAACCAAAAATGATAGGGGGAATTGGAGGTTTTATC
AAAGTAAARCAATATGATCAGATACTTATAGAAATTTGTGGAAAAAAGGCTATAGGTACA
GTRTTAGTAGGACCTACACCTGTCAACATAATTGGACGAAATATGTTGACTCAGATTGGT
TGTACTTTAAATTTTCCAATTAGTCCTATTGACACTGTACCAGTAACATTAAAGCCAGGA
ATGGATGGACCAAAGGTTAAACAGTGGCCATTGACAGAAGAAAAAATAAAAGCATTAACA
GAAATTTGTAAGGAAATGGAAGAGGAAGGAAAAATCTCAAAAATTGGGCCTGAAAATCCA
TATAATACYCCAGTGTTTGCTATAAAGAAAAAGGATGGCACCAAATGGAGGAAATTAGTA
GATTTCAGAGAGCTCAATAAAAGAACTCAGGACTTTTGGGAAGTTCAATTAGGAATACCG
CATCCAGCAGGATTAAAAAAGAAAAAATCAGTGACAGTACTAGATGTGGGAGATGCATAT
TTTTCAGTCCCKTTAGATGAAAACTTTAGAAAGTATACTGCATTCACCATACCTAGTGTA
AACAATCAGACACCAGGAATTAGATATCAGTACAATGTGCTACCACAGGGATGGAAAGGA
TCTCCGGCAATATTCCAGTGTAGCATGACAAAAATCTTAGAGCCCTTTAGAAGGCAAAAT
CCAGAAATGRTTATCTATCAATAYGTGGATGACTTGTATGTAGCGTCTGATTTAGAAATA
GGGCAGCACAGAACAAAAATAGATGAGCTGAGAGCTCATCTATTGAGCTGGGGATTTACT
ACACCAGACAAAAAGCATCAGAAGGAACCACCATTTCTTTGGATGGGATATGAACTCCAT
CCGGACAGATGGACAGTCCAGCCTATAGAACTGCCAGAAAAAGACAGCTGGACTGTCAAT
GATATACAGAAATTAGTGGGAAAACTAAATTGGGCAAGTCAAATTTATGCAGGAATTAAG
ATAAAGCAACTGTGTAAACTCC-------------------------------------

>82
-----------------AGCCCCACCAGCAGAATACTGGGGGATGGGGGAGGAGACAAC
CTC---------CTCACTGAAGCAGG---------------AGCAGAAAGACAAGGACCA
TCCTCCTCCTTTAACTTCCCTCAAATCACTCTTTGGCAACGACCCCTTGTCACAGTAAAA
ATAGGAGGACAARTGAAAGAAGCTCTATTAGATACAGGAGCAGATGATACAGTATTAGAA
GATATAAATTTGCCAGGAAAATGGAAACCAAAAATGATAGGGGGAATTGGAGGTTTTATC
AAGGTAAAGCAATATGATCAAATACTTATAGAAATTTGTGGAAAAAGGGCTATAGGTACA
GTGTTAGTAGGACCTACACCTGTCAACATAATTGGACGAAATATGTTGACTCAGATTGGT
TGTACTCTAAATTTCCCAATTAGTCCTATTGACACGGTACCAGTAACATTAAAGCCAGGA
ATGGATGGACCAAAGGTTAAACAGTGGCCATTAACAGAAGAAAAAATAAAAGCATTAACA
GAAATTTGTAAAGAGATGGAAGAGGAAGGAAAAATCTCAAAAATTGGGCCTGAAAATCCA
TACAATACTCCAGTATTTGCTATAAAGAGAAAGGACAGTACCAAATGGAGGAAATTAGTA
GATTTCAGAGAGCTCAATAAAAGAACTCAGGACTTTTGGGAAGTTCAATTAGGAATACCT
CATCCAGCAGGTTTASAGCAAAAGAAATCAGTAACAGTACTAGATGTGGGAGATGCATAT
TTTTCAGTTCCCTTAGATGAAAGCTTTAGAAAGTATACTGCATTTACCATACCTAGTACA
AACAATGAGACACCAGGAATCAGATATCAGTACAATGTGCTGCCACAGGGATGGAAAGGA
TCACCAGCAATATTCCAGAGTAGCATGACAAAGATCTTAGAGCCCTTTAGAATAAAAAAT
CCAGAAATAGTTATCTRTCAATACATGGATGACTTGTATGTAAGCTCTGATTTAGAAATA
GGGCAGCACAGAATAAAAATAGAGGAGCTGAGAGCTCATCTATTGAGCTGGGGATTTACT
ACCCCAGACAAAAAGCATCAGAAGGAACCTCCATTCCTTTGGATGGGATATGAACTCCAT
CCTGACAAATGGACAGTCCAGCCTATAGAACTGCCAGAAAAAGACAGCTGGACTGTCAAT
GATATACAGAAATTAGTGGGGAAACTAAATTGGGCAAGTCAAATTTATGCAGGAATTAAG

GTAAAGCAACTGTGTAAACTCCTCAGGGGAACTAAAGCACTAACAGACGTAGTACCACTG
ACTGAAGAAGCAGAGC--

>83
-----------GCCAAACAGCCCCACCAGAGGAGAGCTTCAGGTTTGGGGAAGAGACAGC
AAC------CCCACCTCAGAAGCAGG---------------AGCCGATAGACAAGGAACT
ATA---TCCTTTAGCCTCCCTCAAATCACTCTTTGGCAACGACCCCTCGTCTCAATAARG
ATAGGGGGGCAAGTAAAGGAAGCTCTATTAGATACAGGAGCAGATGATACAGTMTTAGAA
GACATGAATTTGCCAGGAAGATGGAAACCAAAAATGATAGGGGGAATTGGAGGTTTTATC
AAAGTAAGACAGTATGATCAGATACCCATAGAAATCTGYGGACACAAGACAGTAGGTACA
GTATTAATAGGACCTACACCTGTTAACATAATTGGGAGAAATCTGTTGACTCAGCTTGGT
TGCACTTTAAATTTTCCCATTAGTCCTATTGAAACTGTACCAGTAAAATTAARGCCAGGA
ATGGATGGCCCAAAAGTTAAACAATGGCCATTAACAGAAGARAAAATAAAAGCATTAGTA
GAAATTTGTACAGAAATGGAAAAGGAAGRAAAAATTTCAAAAATCGGGCCTGAAAATCCA
TACAATACTCCAGTATTTGTCATAAAGAAAAAAGATAGTACTAAATGGAGAAAATTAGTA
GATTTCAGAGAACTCAATAAAAGGACTCAAGACTTCTGGGAAATTCAATTAGGAATACCA
CATCCCGCAGGGTTAAAAAAGAGRAAATCTGCAACAGTACTGGATGTGGGGGATGCATAC
TTCTCAGTCCCTTTAGATAAAGAATTTAGAAAGTATACTGCATTTACCATACCTAGTTTA
AACAATGAGACACCAGGGATCAGATATCAGTACAATGTGCTTCCACAGGGATGGAAGGGA
TCACCAGCAATATTCCAAWGTAGCATGACAAAAATTTTGGARCCTTTTAGRAAACAGAAT
CCAGACATAGATATCTATCAATACGTGGATGATTTGTATGTAGGATCTGACTTAGAAATA
GGGCAACATAGAGCAAAAATAGAGGAACTRAGACAACATCTGTTAAGTTGGGGATTYACC
ACACCAGACAAAAAACATCAGAAAGAACCTCCATTCCTYTGGATGGGRTATGAACTCCAT
CCTGATAAATGGACAGTACAGCCTATAGTGCTGCCAGAAAAGGACAGCTGGACTGTCAAT
GACATACAGAAGTTAGTGGGAAAGTTGAATTGGGCAAGYCAGATTTATGCAGGGATTAAG
GTAARGGAATTATGTAAACTCCTTAGRGGAACCAAAGCACTAACAGAAGTARTACCACTA
ACARAAGAAGCAGAGCTAGAAMTGGCAG--------------------------------

>84

----CATCCCTCAAYTTCCCTCAAATCACTCTTTGGCAACGACCCCTAGTCACAATAAAA
ATAGGAGGACAGYTGAGAGAAGCYCTATTAGATACAGGAGCAGATGATACAGTATTAGAA
GATATAAATTTGCCAGGAAAATGGAAACCAARAATGATAGGGGGAATTGGAGGTTTTATC
AAAGTAAGGCAATATGATCAGATACYTATAGAAATTTGTGGAAAACAGGCTATAGGTACA
GTGTTAATAGGACCTACACCTGTCAAYATAATTGGACGAAATATGTTGACTCAGATTGGT
TGTACTTTAAATTTTCCAATTAGTCCTATTGAKACTGTACCAGTAACATTAAAACCAGGA
ATGGATGGACCAAAGGTTAAACARTGGCCATTGACAGAAGAAAAAATAAAAGCATTAACA
GAAATTTGTARGRARATGGAARAGGAAGGAAAAATCTCAAAGATTGGGCCTGAAAATCCA
TATAATACTCCAGTATTTGCTATAAAGAAAAAGAACAGCACCGAATGGAGGAAATTAGTA
GATTTCAGAGAGCTCAATAAAAGAACTCAGGATTTTTGGGAAGTTCAATTAGGAATACCG
CATCCAGCAGGATTAAAAAAGAGRAAATCAATGACAGTACTAGATGTGGGAGATGCATTT
TTTTCAGTCCCTTTAGATGAGAACTTTAGAAAGTATACTGCATTCACCATACCTAGTATA
AATAATGAGACACCAGGAATCAGATATCAGTACAATGTGCTACCACAGGGATGGAAAGGA

TCTCCAGCAATATTCCAGTGTAGCATGACAAAAATCTTAGAGCCCTTTAGAAGCAAAAAT
CCAGAGATAGATATCTATCAATATGTGGATGACTTGTATGTAGGATCTGATTTAGAAATA
GGGCARCACAGARCAAAAATAGAGGAGCTAAGAGCYCATCTATTAAGCTGGGGATTTACT
ACACCAGAYAAAAAGCATCAGAAGGAACCGCCATTTCTTTGGATGGGATATGAACTCCAT
CCGGACARATGGACAGTCCAGCCTATASAACTGCCAGAAAARGACAGCTGGACTGTCAAT
GATATACAGAAATTAGTGGGAAAACTAAATTGGGCAAGYCAAATTTATCCAGGGATTAGG
ATAAAGCAAYTGTGTAAACTCCTCAGGGGAACWAAAGCATTAACAGACGTAGTACCATTG
ACTGAAGAAGCAGAGCT--

>85
-----------------CAGCCCCACCAGAGGAGAGCTTCAGGTTTGGGGAAGAGACAGC
AAC------TCCATCTCAGAAGCAGG---------------AGCCGAGGGACAAGGAACT
ATA---TCCTTTGACCTCCCTCAGATCACTCTTTGGCAACGACCCCTCGTTACAATAAAG
ATAGGGGGGCAATTAAAGGAAGCTCTATTAGATACAGGAGCAGATGATACAGTATTAGAA
GAAATGAATTTGCCAGGAAGATGGAAACCAAAAATGATAGGAGGAATTGGAGGTTTTATC
AAAGTAAGACAGTATGATCAGGTATCCATAGAAATYTGTGGACACAARGYTRTGGGTACA
GTATTAATAGGACCTACACCYGTCAACATAATTGGGAGAAATCTGTTGACTCAGCTTGGT
TGYACTTTAAATTTTCCTATTWGTCCTATTGAAACTGTACCAGTAAAATTAAAGCCAGGA
ATGGATGGGCCAAAAGTTAAACAATGGCCATTAACAGAAGAAAAAATAAAAGCATTAGTA
GAAATTTGTACAGAACTGGAAAAGGAAGGGAAAATTTCAAAAATTGGGCCTGAAAATCCA
TACAATACTCCAGTATTTGCCATAAAGAAAAAAGACAGTACTAAATGGAGAAAATTAGTA
GATTTCAGGGAACTTAATAAAAGAACTCAAGACTTCTGGGAAGTGCAATTAGGAWTACCA
CATCCCGCAGGGTTAAAAAAGAAAAAATGTGCAACAGTCCTGGATGTGGGTGATGCATAC
TTCTCAGTCCCTTTAGATAAAGACTTCAGGAAGTATACTGCATTTACCATACCTAGTGTA
AACAATGAGACACCAGGGATCAGATATCAGTACAATGTGCTTCCACAGGGATGGAAAGGA
TCACCAGCAATWTTCCAATGTAGCATGACAAAAATCTTAGAKCCTTTTAGAAAACAAAAT
CCAGAYATARTTATCTATCAATACGTRGATGATTTGTATGTAGSATCTGACTTAGAAATA
GGGCAGCATAGAGCAAAAGTAGAGGAACTAAGACAACATTTGTGGGGGTGGGGATTTTAC
ACACCAGACAAAAAACACCAGAAAGAGCCTCCACTCCGTTGGATGGGTTATGAACTCCAT
CCTGATAAATGGACAGTACAGCCTATAGTGCTGCCAGAAAAGGACAGCTGGACTGTCAAT
GACATACAGAAGTTAGTGGGAAAGTTAAATTGGGCAAGTCAGATTTATGCAGGAATTAAR
GTAAGGGAATTATGTAAACTCCTTAGGGGAGCCAAAGCACTAACAGAAGTAATACCACTA
ACAGAAGAAGCAGAGCTAGAACTGGC---------------------------------

>86

----------------------AGG---------------AACAGAAAGACAGGGAA--
-------CCTTTAACTTCCCTCAGATCACTCTTTGGCAGCGACCCCTTGTCTCAATAAGA
GTAGGGGGCCAGATAAAAGAGGCTCTCTTAGATACAGGAGCAGATGATACAGTATTAGAA
GAAATAGACTTGCCAGGAAGATGGAAACCAAAAATGATAGGAGGAATTGGAGGTTTTATC
AAAGTAAGACAATATGATCAAATACCCATAGAAATTTGTGGRAAAAAGGCTATAGGTACA
GTATTAGTAGGACCMACACCTRTCAACATAATTGGAAGRAATYTGTTGACTCAGCTTGGG
TGYACACTAAATTTTCCTATCAGTCCTATTGAAACTGTACCAGTAAAATTAAAGCCAGGA
ATGGATGGCCCRAARGTTAAACAATGGCCATTGACAGAAGAAAAAATAAAAGCATTAACA

GAAATTTGTAATGAAATGGAGAAGGARGGAAAAATTTCAAAAATTGGRCCTGAAAATCCA
TATAATACTCCAATATTTGCYATAAAAAAGAARRACAGTACTAAGTGGAGAAARTTAGTA
GATTTCAGGGAACTCAATAAAAGAACTCAAGATTTCTGGGAAGTGCAATTAGGAATACCA
CAYCCAGCAGGATTRAAACAGAAMAAATCAGTRACAGTACTRGATGTGGGGGATGCATAT
TTTTCAGTTCCTTTATATGAAGAHTTCAGRAAGTATACTGCATTCACCATACCTAGTGTR
AACAATGAGACACCAGGGATTAGRTATCAGTATAATGTGCTTCCACAGGGATGGAAAGGA
TCACCAGCAATATTYCAATGTAGCATGACAAAAATCTTAGAGCCTTTTAGAAARMRAAAT
CCAGACATAGTTATMTATCAATACATGGATGATTTGTATGTWGSATCAGACTTAGAAATA
GGGCARCATAGAGCAAAAATAGAGGAACTGAGAGAACATCTGTTAARGTGGGGATTYACC
ACACCAGACAAGAAACATCAGAAAGAACCYCCATTTCTKTGGATGGGRTATGAACTCCAT
CCKGACAAATGGACAGTACAGCCTATMCAGCTGCCAGAACAGGATAGCTGGACTGTCAAT
GATATACAGAAGTTAGTGGGAAAATTAAACTGGGCAAGTCAGATTTAYCCAGGAATYAAR
GTAAGGCARCTTTGTAAACTCCTTAGGGGGGTCAAAGCRTTAACAGAAATAGTACCACTA
ACTGARGAAGCAGAGC--

>87
----------------ACAGCCCCACCAGCGGAGAGTTTCGGGTTTGGGGAAGAGACAAC
AAC------TCCATCCCAGAAGCAGG---------------AGCCGGTAGAGAAGGAGCT
ATA---TCCTTTAGCCTCCCTCAAATCACTCTTTGGCAACGACCCCTCGTCACAATAAAG
ATAGGGGGGCAATTAAARGAAGCTCTATTAGATACAGGAGCAGATGATACAGTATTAGAA
GACATGAATTTGCCAGGAAGATGGAAACCAAAAATGATAGGGGGAATTGGAGGTTTTATC
AAAGTAAGACAGTATGATCAGATACCCATAGAAATCTGYGGACACAARGCTGTAGGTACA
GTATTAATAGGACCTACACCTGTCAACATAATTGGGAGAAATCTGTTGACTCAGCTTGGT
TGCACTCTAAATTTTCCCATTAGTCCTATTGAAACTGTACCAGTAAAATTAAAGCCAGGA
ATGGATGGCCCAAAAGTTAAACAATGGCCATTGACAGAAGAAAAAATAAAAGCATTAGTA
GARATTTGTACTGAAATGGAAAAGGAAGGRAAAATTTCAAGAATAGGGCCTGAAAATCCA
TACAATACTCCAGTATTTGYCATAAAGAGAAAAGAYARTACTAAATGGAGAAAATTAGTA
GATTTCAGGGAACTTAATAAAAGAACTCAAGACTTTTGGGAAGTTCAATTAGGAATACCA
CATCCTGCAGGGTTAGAAAAGAAAAAATCCATRACARTCCTGGATGTGGGTGATGCATWT
TTCTCAGTCCCTTTAGATAAAGACTTCAGGAAGTATACTGCATTTACCATACCTAGTGTA
AACAATGAGACACCAGGGATTAGATATCARTACAATGTGCTTCCACAGGGATGGAAAGGA
TCACCAGCAATATTCCAGAGTAGCATGACAAAAATCTTAGAGCCTTTTAGAAAACAAAAT
CCAGACATAGTTATTTGTCAATACGTGGATGATTTGTATRTAKCATCTGATTTAGAAATA
GGGCAGCATAGAGCAAAAATAGAGGAACTGAGACARCATCTGTTGAGGTGGGGATTTACC
ACACCAGACAAAAARCATCAGAAAGAACCYCCATTCCTKTGGATGGGTTATGAACTCCAT
CCKGAWAAATGGACRGTACAGCCTATATTRCTRCCAGAAAAGGACAGCTGGACTGTCAAT
GACATACAGAARTTAGTGGGAAAATTAAATTGGGCAAGTCAGATTTATGCAGGGATTAAG
GTAAGGGAATTATGTAAACTCCTRAGGGGAACTAAAGCGCTAACAGAAGT----------

>88
--------------------CCCACCAGCGGAAGACTGGGGGATGGGGGAAGAGATAAC
CTC---------CTCGCAGAAACAGG---------------AGCAGAAAGACAGGGAAC-
-----CTCCTCCAACTTCCCTCAAATCACTCTTTGGCAACGACCCATGGTCACAGTAAAA

ATAGGAGGGCAGCTGATAGAAGCCCTATTAGATACAGGAGCAGATGATACAGTATTAGAA
GATATAAATCTGCCAGGAAAATGGAAACCAAAAATGATAGGGGGAATTGGAGGTTTTATC
AAAGTGAAGCAATATGATCAGATACTTATAGRAATTTATGGAAAAAGGGCTATAGGTACA
GTATTAGTAGGACCTACACCTGTCAACATAATTGGACGAAACATGTTGACTCAGATTGGT
TGTACTCTAAATTTCCCAATTAGTCCTATTGATACTGTACCAGTAAAATTAAAGCCAGGA
ATGGATGGACCAAAGGTTAAACAGTGGCCATTGACAGAAGAAAAAATAAAAGCATTAACA
GAAATTTGTAAAGAAATGGAACAGGAAGGAAAAATCTCAAAAATTGGGCCTGAAAATCCA
TACAATACTCCAGTATTTGCTATAAAGAAAAAGGACAGCACCAAATGGAGAAAATTGGTA
GATTTCAGAGAGCTTAATAAAAGAACTCAGGACTYTTGGGAAGTTCAATTAGGAATACCG
CATCCAGCAGGTTTAAAAAAGAGAAAATCCATGACAGTACTAGATGTGGGAGATGCATAT
TTTTCAGTTCCTCTAGATAAAGACTTTAGAAAGTATACTGCATTCACCATACCTAGTACA
AACAATGAGACACCAGGAATYAGATATCAGTACAATGTGCTTCCACAGGGATGGAAGGGA
TCACCAGCAATATTCCAAAGTAGCATGATAAAAATCTTAGAGCCTTTTAGAAAACAAAAT
CCAGAAATAGACATCTATCAATACGTGGATGATTTGTATGTAGGATCTGACTTAGAAATA
GGGCAGCATAGAGCAAAAGTAGAGGAACTGAGGCAACATCTGTTACGGTGGGGACTTACC
ACACCAGACAAAAAACATCAGAAGGAACCTCCATTCCTTTGGATGGGATATGAACTCCAT
CCTGACAAATGGACAGTCCAGCCTATAGAACTGCCAGAAAAGGACAGTTGRACTGTCAAT
GATATACAGAAATTAGTAGGAAAACTAAATTGGGCAAGCCAGATTTATGCAGGAATTAAG
GTAAAACAACTRTGTAGACTCCTCAGGGGAGCTAAAGCACTAACAGAAATAGTACCACTG
ACTAGAGAAGCAGAGC---

>90
----------------------------GARGAGAGCTTCAGGTTTGGGGAAGAGACARC
AAC------TCCATCTCAGAAGCAGG---------------AGCCGATAGACAAGGAMCT
RTA---TCCTYTARCYTCCCTCAAATCACTCTTTGGCAACGACCCCTMGTCACAATAAAG
ATAGGGGGGCAATTAAAGGAAGCTCTATTAGATACAGGAGCAGATGATACAGTATTAGAA
GACATGAATTTGCCAGGAARATGGAAACCAAAAATGATAGGGGGAATTGGAGGTTTTATC
AAAGTAAGACAGTATGATAAGATACCCATAGAAATCTGTGGACACAAGGCTATAGGTACA
GTATTAATAGGACCTACACCTRTCAACATAATTGGAAGAAATCTGTTGACTCAGCTTGGT
TGCACTTTAAATTTYCCCATTAGTCCTATTGAAACTGTACCAGTAAAATTAAAGCCAGGA
ATGGATGGCCCAARAGTTAAACAATGGCCATTGACAGAAGARAAAATAAAAGCMTTAGTA
GAAATTTGTACAGARATGGAAAAGGAAGGRAAAATTTCAAAAATYGGGCCTGAAAATCCA
TAYAATACTCCAGTATTTGCCATAAAGAAAAAAGAYAGTACTAAATGGAGAAARTTAGTA
GATTTCAGRGAACTTAATAAAAGAACTCAGGACTTCTGGGAAGTTCAATTAGGAATACCA
CATCCMGCAGGGTTRAARAAGAAAAAATCTGTAACAGTYCTGGATGTGGGTGATGCATAT
TTCTCAGTYCCTTTAGATAAAGACTTCAGGAAGTATACTGCATTTACCATACCTAGTGTA
AACAATGAGACACCAGGGATYAGATATCAGTACAATGTGCTTCCACAGGGATGGAAAGGR
TCACCAGCAATATTYCAATGTAGCATGACAAAAATYTTAGAGCCTTTTAGAAAACAGAAT
CCAGACATAGTTATCTATCAATACATGGATGATTTGTATGTAGGATCWGAYTTAGAAATA
GGRCAGCATAGAGCAAAAATAGAGGAACTGAGACAACATTTGTTGAGGTGGGGATTTACC
ACACCAGACAAAAARCATCAGAAAGAACCYCCATTCCTTTGGATGGGTTATGAACTCCAT
CCTGATAAATGGACAGTACAGCCTATAGAGCTGCCAGAAAAGGACAKCTGGACTGTCAAT
GACATACAGAAGTTAGTRGGAAAGTTRAATTGGGCAAGYCARATTTATGCAGGGATYARG
GTAAAGGAATTATGTAAACTCCTTAGGGGARCCAAAGCACTAACAGAGGTAATACCACTA

ACAGAAGAAGCAGAGC--

>91

--------------------CCCACCAGAAGAGAGCTTCAGRTT---CGAGGAGACAAC

CCC---------AGCTCCGAAGCAGG---------------GAACGAAAGACAGGGAA--

-------GCCTTAACTTCCCTCAGATCACTCTTTGGCAACGACCCCTTGTCWCAATAAGA

GTAGGGGGCCAGACAAAAGAGGCTCTCCTAGATACAGGAGCAGATGATACAGTATTAGAA

GAAGTAAATTTGCCAGGCAAATGGAAACCAAAAATGATAGGGGGAATTGGAGGTTTTATC

AAAGTAAGACAGTATGATCAGRTACCCATAGAAATTTGCGGRCACAAAGCTATAGGTACA

GTATTAGTAGGACCTACACCTGTCAACATAATTGGAAGAAATCTGTTGACTCAGCTTGGT

TGCACTTTAAATTTTCCCATTAGTCCTATTGAAACRGTACCAGTAAAATTAAAGCCAGGA

ATGGATGGCCCAAAGGTTAAACAGTGGCCATTGACAGAAGAAAAAATAAAAGCATTAACA

GAAATTTGCAAAGAAATGGAAGAGGAAGGGAAAATCTCAAAAATTGGGCCTGAAAATCCA

TACAATACTCCAGTATTTGCTATAAAGAAAAAGGACAGCACCAAATGGAGGAAACTAGTA

GATTTCAGAGAGCTCAATAAAAGAACTCAGGATTTTTGGGAAGTTCAATTAGGAATACCA

CATCCAGCAGGATTAAAAAAGAAAAAATCAGTAACAGTACTAGATGTGGGAGATGCATAT

TTTTCAGTTCCKTTAGATGAAARCTTTAGAAAGTATACTGCATTCACCATACCTAGTATA

AACAATGAGACACCAGGAATCAGATATCAGTACAATGTGCTGCCACAGGGATGGAAAGGA

TCACCAGCAATATTCCAGAGTAGCATGACAAAAATCTTAGAGCCCTTTAGAGTAAAAAAT

CCAGAAATAATTATCTATCAATACATGGATGACTTGTATGTAGGATCTGATTTAGAAATA

GGGCAGCATAGAACAAAAATAGAGGAGCTRAGAGCTCATCTATTGAGCTGGGGGTTTACT

ACACCAGACAAAAAGCATCAGAAGGAACCCCCATTCCTTTGGATGGGATATGAACTCCAT

CCTGATAGATGGACAGTYCAGCCTATAAAACTGCCAGAAAAAGAYAGCTGGACTGTCAAT

GATATACAGAAATTAGTRGGAAAACTAAATTGGGCAAGTCARATTTATGCAGGGATTAAG

GTAAAGCAACTGTGTAAACTCCTTAGG---------------------------------

>92

--------------------CCCACCAGAAGAGAGCTTCAGGTT---CGAGGAGACAAC

CCC---------AGCTCAGAAGCAGG---------------GAACGAAAGACAGGGAA--

-------GCCTTAACTTCCCTCAGATCACTCTTTGGCAACGACCCCTTGTCTCAATAAGA

GTAGGGGGCCAGACAARAGAGGCTCTCCTAGATACAGGAGCAGATGATACAGTATTAGAA

GAAGTAAATTTGCCAGGAAAATGGAAACCAAAAATGATAGGGGGAATTGGAGGTTTTATC

AAAGTAAGACAGTATGATCAGGTACCCATAGAAATYTGCGGACACAAAGCTATAGGTACA

GTATTAGTAGGACCYACACCTGTCAACATAATTGGAAGAAATCTGTTGACTCAGCTTGGT

TGCACTTTAAATTTTCCCATTAGTCCTATTGAAACGGTACCAGTAAAATTAAAGCCAGGA

ATGGATGGCCCAAAGGTTAAACAGTGGCCATTGACAGAAGAAAAAATAAAAGCATTAACA

GAAATTTGCAAAGAAATGGAAGARGAAGGRAAAATCTCAARAATTGGGCCTGAAAATCCA

TACAATACTCCAGTATTTGCTATAAAGAAAAAGGACAGCACCAAATGGAGRAAACTAGTA

GATTTCAGAGAGCTCAATAAAAGAACTCAGGATTTTTGGGAAGTTCAATTAGGAATACCA

CATCCAGSAGGATTAAAAAAGAAAAAATCAGTAACAGTRYTAGATGTGGGAGATGCATAT

TTTTCAGTTCCTTTAGATGAAAGCTTTAGAAAGTATACTGCATTCACCATACCTAGTATA

AACAATGAGACACCAGGAATCAGATATCAGTACAATGTGCTGCCACAGGGATGGAAAGGA

TCACCAKCAATATTCCAGAGTAGCATGACAAAAATCTTAGAGCCCTTTAGAGYAAAAAAT

CCAGAAATAATTATCTGTCAATACGTGGATGACTTGTATGTAGGATCTGATTTAGAAATA
GGACAGCATAGAACAAAAATAGAGGAGCTAAGAGCTCATCTATTGAGCTGGGGGTTTACT
ACACCAGACAAAAARYATCAGAAGGAACCCCCATTCCKTTGGATGGGATATGAACTCCAT
CCTGATAGATGGACAGTCCAGCCTATAGAACTGCCAGAAAAAGACAGCTGGACTGTCAAT
GATATACAGAAATTRGTGGGAAAACTAAATTGGGCAAGTCAAATTTATGCAGGGATTAAG
GTAAAGCAACTGTGTAAACTCCTTAGGGGGACTAAAGCACTAACAGACATAGTACCACTG
ACTGAAGAAGCARAG--

>93
---------------------CCACCAAWGGAGAGCTTCAGGTTTGGGGAAGAGRCAGY
AAC------TCMRTCTCAGAAGCAGG---------------ARCCGATAGACAAGGAATT
ATA---TCCTTTGACCTCCCTCAAATCACTCTTTGGCAACGACCCMTCGTCCCAATAAGG
GTAGGGGGGSARTTAAAGGAAGCTCTATTAGATRCAGGAGCAGATGATACAGTATTAGAA
GACATGAATTTGCCAGGRAAATGGAGACCAAAAATGATAGGGGGAATTGGAGGTTTTATC
AAAGTAAGACAGTAYGATCAGATACCCATAGAAATYTGCGGRCAYAAAGCTATAGGTACA
RTATTAGTAGGACCTACACCTGTCAACATAATTGGAAGAAATYTGTTGACTCAGCTTGGT
TGYACYTTAAATTTTCCCATTAGTCCTATTGAHACTRTACCAGTAAAAYTAAARCCAGGA
ATGGATGGCCCKAAAGTYAAACAATGGCCATTGACAGAAGARAAAATAARAGCATTARYA
GAAATTTGTACAGAAATGGAAAAGGAAGGRAAAATTTCAAAAATYGGGCCTGAAAATCCA
TACAATACTCCAGTATTTGCCATAAAGAAAAAAGACAGTACCAAATGGAGAAAATTAGTA
GATTTCAGGGAACTTAATAAAAGAACTCAAGACTTCTGGGAAGTTCAATTAGGAATACCA
CAYCCYGCAGGRYTAAAAAAGAAVAAATCYRTAACAGTCCTAGATGTGGGTGATGCATAT
TTCTCMGTYCCTTTAGATGAAGACTTCAGGAAGTATACTGCATTTACCATACCTAGTKTA
AACAATGAGACACCAGGGGTTAGATATCAGTACAATGTGCTTCCACAGGGATGGAAAGGR
TCACCAGCAATATTCCAATSTAGCATGACAAAAATCTTAGAGCCTTTTAGARAACRAAAT
CCAGATMTARTTATCTATCAATACATGGATGATTTGTATGTAGSATCTGACTTRGAAATA
GGRCAGCATAGAACAAAAATAGAGGAACTGAGAMAACATCTGWTGSMGTGGGGATTTACC
ACACCAGACAAAAAACATCAGAAAGAACCTCCRTTCCTTTGGATGGGTTATGAACTYCAT
CCWGATAAATGGACAGTACAGCCTATARTRYTGCCAGAAAAAGACAGCTGGACTGTCAAT
GACATACAGAAGTTAGTGGGRAARTTRAATTGGGCAAGTCAGATTTATGCAGGMATTAAG
GTAAARGAATTATGTAAACTCCTTAGGGGAACCAAAGCAYTAACAGAAGTAGTACCACTR
ACAGAAGAAGCAGAGCTAGAACTGGCAGA-------------------------------

>94

CCC---------CTCACCGAAGCAGG---------------AGCAGAAAGACCAGGAACA
TCCTCCTCCCTCAGTTTCCCTCAAATCACTCTTTGGCAACGACCYATTGTCACAGTAAAA
ATAGCAGGACAGCTGAARGAAGCTCTATTAGATACAGGAGCAGATGATACAGTATTAGAA
GATATAAATTTGCCAGGAAAATGGAAACCAAAAATGATAGGAGGAATTGGAGGYTTTATC
AARGTAAAGCAATATGATCAGATACTTATAGAAATTTGTGGACACARGGCTRTAGGTACA
GTGTTAGTAGGACCCACACCTGTCAACATAATTGGGCGAAATATGTTGACTCAASTTGGT
TGTACTTTAAATTTCCCAATTAGTCCTATTGACACTGTACCAGTAACATTAAAGCCAGGA
ATGGATGGACCAAARGTTAAACARTGGCCMTTGACAGAAGAAAAAATAAWAGCRTTAAYR
GAAATTTGTAAAGAGATGGAAAAGGAAGGAAAAATTTCAAAAATWGGGCCTGAAAATCCA

TACAATACTCCAGTATTTGCTATAAAGAAAAAGGACAGCACCAAATGGAGRAAATTAGTA
GATTTTAGAGAACTTAATAAAAGAACTCAGGACTTTTGGGAAGTTCAATTAGGAATACCG
CATCCAGCGGGGTTAGAAAGRAAAAAATCAGTAACAGTACTAGATGTGGGAGATGCATAT
TTTTCAGTTCCTTTAGATGAAAGCTTTAGAAAGTATACTGCATTCACCATACCTAGTATA
AACAATGAGACACCAGGAATTAGATATCAATACAATGTGCTGCCACAGGGATGGAAGGGG
TCACCAGCAATATTCCAGAGTAGCATGACAAAAATCTTAGAGCCCTTTAGAAGAAAAAAT
CCAGAAATAATTATCTATCAATACGTGGATGACTTATATGTAGCATCTGATTTAGAAATA
GGGCAGCACAGARCAAAAATAGAGGAGCTAAGAGCTCATCTATTRAGCTGGGGATTTACT
ACCCCAGACAAAAAGCATCAGAAGGAACCTCCGTTCCTTTGGATGGGATATGAACTCCAT
CCTGACAGATGGACAGTCCAGCCTATAGAACTGCCAGAAAAAGACAGCTGGACTGTCAAT
GATATACARAAATTAGTGGGAAAACTAAATTGGGCAAGTCAAATTTATGCAGGGATTAAG
GTAAAGCAACTGTGTAGACTCATCAGGGGAACTAAAGCACTAACAGACATAGTACCACTG
ACTGCAGAAGCAGAGCTAG--

>95
---------------------CCCACCAGCAGAAGACTGGGGGATGGGAGGGGAGATAAC
CTC---------CTTACCGAAGCAGG---------------AGCAGAAAGACAAGGAACC
------TCCYTCAGTCTCCCTCAAATCACTCTTTGGCAACGACCCCTTGTCACAATAAAA
ATAGAAGGACAGCTAAGAGAAGCTCTATTAGATACAGGAGCAGATGATACAGTATTAGAA
GATATAAATTTGCCAGGAAAATGGAAACCAAAAATGATAGGGGGAATTGGAGGTTTTATC
ARGGTAAGGCAATATGATCAGATACYTATAGAAATTTGTGGAAAAAGGGCTATAGGTACA
GTGTTAGTAGGACCTACACCTGTCAACATAATTGGACGAAATATGTTGACTCAGCTTGGT
TGTACTTTAAATTTYCCAATTAGTCCTATTGACACTGTACCAGTAACATTAAAGCCAGGA
ATGGATGGACCAAGGGTTAAACAGTGGCCATTGACAGAAGAAAAAATAAAAGCATTAACA
GAAATTTGTAAAGAGATGGAARAGGAAGGAAAAATTTCAAAAATTGGGCCTGAAAATCCA
TATAATACTCCAGTATTTGCYATAAAGAAAAAAGACAGTAACAAATGGAGAAAATTAGTA
GAYTTCAGAGAGCTCAATAAAAGRACTCAGGACTTTTGGGAAGTTCAATTAGGAATACCG
CATCCAGCAGGATTRAAAWGGAAYAAAGCAGTAACARTACTAGATGTAGGAGATGCATAT
TTTTCAGTTCCTTTAGATRAAAACTTTAGAAAGTATACTGCATTCACCATACCTAGTATA
AACAATGAGACACCAGGAATCAGATATCAGTACAATGTRCTACCACAGGGATGGAAAGGA
TCTCCGGCAATATTTCAGTGTAGCATGACAAAAATCTTAGAGCCCTTTAGAAGAAGAAAT
CCAGAGCTAGTTATCTATCAATACATGGATGACTTGTATGTAGGATCTGATTTAGAAATA
GGGCAGCACAGAGCAAAAATAGAGGAGCTAAGAGCTCATCTATTGAGCTGGGGATTTACT
ACACCAGACAAAAAGCATCAGAAGGAACMTCCATTTCTTTGGATGGGATATGAACTCCAT
CCGGACAGATGGACAGTCCAGCCTATAGAACTGCCAGAAAAAGATAGCTGGACTGTCAAT
GATATACAGAAATTAGTGGGAAARTTAAATTGGGCAAGTCAAATTTATCCAGGGATTAGG
GTAAAGCAACTRTGTAAACTCCTCAGGGGAGCTAAAGCTCTAACAGACATAGTACCACTG
ACAGAAGAAGCAGAGCT---

>96
----------------------GACTGGGGGATGGGAGGAGAGATAACCCCCTTCTTACC
GAA---------GCAGGGGCAGAAGG---------------AGCAGAAAGACGAGGAACA
------TCCTTCARTCTCCCTCAAATCACTCTTTGGCAACGACCCCTTGTCACAATAARA
ATAGGAGGACAACTAAARGAAGCTCTATTAGATACAGGAGCWGATGATACAGTATTAGAR

GATATAAATTTGCCAGGAAGATGGAAACCAAAAATGATAGGGGGAATTGGAGGTTTTATC
AARGTAARGCAATATGATCAGATACCTATAGAAATTTGTGGAAAAAAGGCTATAGGTACA
GTATTAGTAGGACCTACWCCTGTCAACATAATTGGACGAAATATGTTGACTCAGCTTGGT
TGTACTTTAAATTTCCCAATTAGTCCTATTGACACTGTACCAGTAAMATTAAAGCCAGGR
ATGGATGGRCCAAAGGTTAAACAGTGGCCATTGACAGAAGAAAAAATAAAAGCATTAACA
GAAATTTGTAAAGAGATGGAAGAGGAAGGRAAAATCTCAAAAATTGGGCCTGAAAATCCA
TATAATACTCCTGTATTTGCTATAAAGAAAAAGGACAGCACCAAATGGAGGAAATTRGTA
GATTTCAGAGAGCTCAATAAAAGAACTCAGGACTTTTGGGAAGTTCAATTAGGAATACCG
CATCCAGCAGGATTAAAAAAGAAAAAATCAGTGACAGTACTRGATGTGGGAGATGCATAT
TTTTCAGTYCCTTTAGATGAAAGCTTTAGAAAGTATACTGCATTCACCATACCTAGTATA
AACAATGAGACACCAGGAATCAGATATCAGTACAATGTAYTACCACAGGGATGGAAAGGA
TCTCCAGCAATATTCCAGTGTAGCATGACAAAAATYTTAGAGCCCTTTAGAAAAARMAAT
CCAGACATGRTTATCTATCAATACATGGATGACTTGTATGTAGGATCTGATTTAGAAATA
GGGCAGCACAGAACAAAAATAGAGGAGCTAAGAGMTCAYCTATTRAGCTGGGGATTTACT
ACACCAGACAAAAAGCATCAAAAGGAGCCTCCATTTCTTTGGATGGGATATGAACTCCAT
CCGGACAGATGGACAGTCCAGCCTATAGAACTGCCAGAAAAAGACAGCTGGACTGTCAAT
GATATACAGAAATTAGTGGGAAAACTAAATTGGGCAAGTCAAATTTATGCAGGGATTAAG
GTAAGGCAACTGTGTAAACTCMTCAGGGGAACTAAAGCAYTAACAGAAATAGTACCACTA
ACTGAAGAAGCAGA--

>97
----------------------CCACCAGCAGAGGACTGGGGGATGGGAGAAGAGATAAC
CTC---------ATTACCGAGGCAGG---------------AGCAGAAAGAGAAGGACCA
GCGCCCTCCTTCAGTTTCCCTCAAATCACTCTTTGGCAACGACCCCTYGTCACAGTAAAA
ATAGAAGGGCAGCTGAGAGAAGCTCTATTAGATACAGGAGCAGATGATACAGTATTAGAA
GATATAAATTTGYCAGGAAAATGGAAACCAAAAATGATAGGGGGAATTGGAGGTTTTATC
AAAGTAAGGCAATATGATCAGATACTTATAGAAATTTGTGGAAAAAAGGCTATAGGTACA
GTGCTAGTAGGACCTACACCTGTCAACATAATTGGACGAAATATGTTGACTCAGATTGGT
TGTACTTTAAATTTTCCAATTAGTCCTATTGACACTGTACCAGTAAMATTAAAACCAGGA
ATGGATGGACCAAAGGTTAAACAGTGGCCATTGACAGAAGAAAAAATAAAAGCATTAACA
GAAATTTGTAAGGAAATGGAARAGGAAGGAAAAATCTCAARAATTGGGCCTGAAAATCCA
TACAATACTCCAGTATTTGCTATAAAGAGAAAGGACAGCACCAAATGGAGGAAATTAGTR
GATTTCAGAGAGCTCAATAAAAGAACTCAGGATTTTTGGGAAGTTCAATTAGGAATACCA
CATCCAGCAGGATTAGAAAAGAAAAAATCAGTGACAGTACTAGATGTGGGAGATGCATAT
TTTTCAGTCCCTTTAGATGAAAATTTTAGAAAGTATACTGCATTCACCATACCTAGTACA
AACAATGAGACACCAGGAATCAGATATCAGTACAATGTGCTACCACAGGGATGGAAAGGA
TCTCCAGCAATATTCCAGTGTAGCATGACAAAAATCTTAGACCCCTTTAGAAAAAGAAAT
CCAGAGATGGTTATCTGTCAATACATGGATGACTTGTATGTAAGTTCTGATTTAGAAATA
GGGCAGCACAGARCAAAAATAGATGAACTGAGAGCTCATCTATTGAGCTGGGGATTTACT
ACACCAGACAAAAAGTATCAGAAGGAACCACCATTTCTTTGGATGGGATATGAACTCCAT
CCGGACAGATGGACAGTCCAGCCCATAGAACTGCCAGAAAAAGACAGCTGGACTGTCAAT
GATATACAGAAATTAGTGGGAAAACTAAATTGGGCAAGTCAAATTTATGCAGGAATTAAG
GTAAAGCAACTGTGTAAACTCCTAAGGGGAGCTAAAGCATTAACAGAAGTAGTACCACTG
ACTGAAGAAGCA--

>98

------------------GCAGAAGG---------------AGCAGAARGACGAGGRACA
------TCCTTCARTCTCCCTCAAATCACTCTTTGGCAACGACCCCTTGTYACAATAARA
ATAGGAGGRCAACTAAAAGARGCTCTATTAGATACAGGAGCAGATGATACAGTATTAGAA
GATATAAATTTGCCAGGRAGATGGAAACCAAAAATGATAGGGGGAATTGGAGGTTTTATC
AAGGTAAGRCAATATGATCARATACCTATAGAAATTTGTGGRAAAAAGGCTATAGGYACA
GTRTTAGTAGGACCTACACCTGTCAACATAATTGGACGAAATATGTTGACTCAGCTTGGT
TGTACTTTAAATTTCCCAATTAGTCCTATTGACACTGTACCAGTAACATTAAAGCCAGGA
ATGGATGGACCAAAGGTTAAACAGTGGCCATTGACAGAAGAAAAAATAAAAGCATTAACA
GAAATTTGTAAAGAGATGGAAGAGGARGGAAAAATYTCAAAAATTGGGCCTGAAAATCCA
TATAATACTCCWGTATTTGCTATAAAGAAAAAGGACAGCACCAAATGGAGRAAATTAGTA
GATTTCAGAGAGCTCAATAAAAGAACTCARGACTTTTGGGAAGTTCAATTAGGAATACCR
CATCCAGCAGGATTAAAAAAGAAMAAATCARTGACAGTACTAGATGTGGGAGATGCATAT
TTTTCAGTWCCTTTAGATGAAAGCTTTAGAAAGTATACTGCATTCACCATACCTAGTATA
AACAATGAGACACCAGGAATCAGATATCAGTACAATGTACTACCACAGGGATGGAAAGGA
TCTCCAGCAATATTCCAGTGTAGYATGACAAAAATCTTAGAGCCCTTTAGAAAMRMAAAT
CCAGACATGATTATCTATCAATACATGGATGACTTGTATGTAGGATCTGATTTAGAAATA
GGGCAGCACAGAACAAAAGTAGAGGAGCTAAGAAAGCATCTATTGAGCTGGGGATTTACT
ACACCAGACAAAAAGCATCAAAAGGAGCCTCCATTTCTTTGGATGGGATATGAACTCCAT
CCGGACAGATGGACAGTCCAGCCTATAGAACTGCCAGAAAAAGACAGCTGGACTGTCAAT
GATATACAGAAAYTWGTGGGAAAACTAAATTGGGCAAGTCAAATTTATSCAGGGATTAAG
GTAAAGCAACTGTGTAAACTCCTCAGGGGAACTAAAGCACTAACAGAAATAGTACCACTA
ACTGAAGAAGCAGAGCT---

>99
------------------------ACCAGCAGAAGACTGGGGGATGGGAGAAGAGATAAC
CTC---------CTTACCGAAACAGG---------------AGCAGAAAGACAAGGACCA
TCRCCCTCCCTCAGTCTCCCTCAAATCACTCTTTGGCAACGACCCCTTGTTACAGTAARA
GTAGGAGGACAGTTGATAGAAGCTCTATTAGATACAGGAGCAGATGATACAGTATTAGAA
GATATAAATTTGCCAGGAAAATGGAAACCAAAAATGATAGGGGGAATTGGAGGTTTTATC
AAAGTAAGACAATATGATCAGATACTTATAGAAATTTGTGGAAAAAAGGCTATAGGTACR
GTATTAGTAGGACCTACACCTGTCAACATAATTGGACGAAATATGTTGACTCAAATTGGT
TGTACTTTAAATTTTCCAATTAGTCCTATTGACACTGTACCAGTAACATTAAAGCCAGGA
ATGGATGGACCAAAGGTTAAACAGTGGCCATTGACAGAAGAAAAAATAAAAGCATTAACA
GAAATTTGTAAGGAGATGGAAGAGGAAGGAAAAATTTCAAGAATTGGGCCTGAAAATCCA
TATAATACTCCAGTATTTGCTATAAAGAAAAAGGACAGCACCAAATGGAGGAAATTAGTA
GATTTCAGAGAGCTCAATAAAAGAACTCAAGACTTTTGGGAAGTACAATTAGGAATACCA
CATCCAGCAGGATTAGAAAAGAAAAAATCAGTAACAGTACTAGATGTAGGAGATGCATAT
TTTTCAGTCCCTTTAGATGAAAGCTTTAGAAAGTATACTGCATTCACCATACCTAGTACA
AACAATGAGACACCAGGAATCAGGTATCAGTATAATGTGCTACCACAGGGATGGAAAGGA
TCCCCGGCAATATTCCAGTGTAGCATGACAAAAATCTTAGAGCCCTTTAGAAAACAAAAT
CCAGAAATGATTATCTATCAATACGTGGATGACTTGTATGTAGCATCTGATTTAGAAATA

GGGCAGCACAGAACAAAAATAGATGAGCTAAGAGCTCATCTATTGAGCTGGGGATTTACT
ACACCAGACAAAAAGCATCAGAAGGAGCCGCCATTTCTTTGGATGGGATATGAACTCCAT
CCGGACAGGTGGACAGTCCAGCCTATAGAACTGCCAGAAAAAGACAGCTGGACTGTCAAT
GATATACAGAAATTAGTGGGAAAACTAAATTGGGCAAGTCAAATTTATGCAGGGATTAAA
ATAAAGCAATTGTGTAAACTCCTCAGGGGAGCTAAAGCATTAACAGACGTAGTACCACTG
ACTGAAGAAGCAGAG--

>100
----------------------CCACCAGCAGAAAACTGGGGAATGGGGGAGGAGATAAC
CTC---------CTTACCGAAGCAGG---------------AGCAGAAAGACAAGGAACA
TCTCCCTCCCTCAGTTTCCCTCAAATCACTCTTTGGCAACGACCCCTTGTCACAATAAAA
ATAGAAGGACAGCTGAAAGAAGCTCTATTAGATACAGGAGCAGATGATACAGTATTAGAA
GATATCAATTTGCCAGGAAAGTGGAAACCAAAAATGATAGGGGGAATTGGAGGTTTTATC
AAGGTAAGGCAATATGATCAGATACTTATAGAAATTTGTGGAAAAAGAGCTATAGGTACA
GTATTAGTAGGACCTACACCTGTCAACATAATTGGGCGAAATATGTTGACTCAGATTGGT
TGTACTTTAAATTTCCCAATTAGTCCTATTGACACTGTACCAGTAAAATTAAAGCCAGGA
ATGGATGGACCAAAGGTTAAACAGTGGCCATTAACAGAAGAAAAAATAAAGGCATTAACA
GAAATTTGTAAAGAGATGGAAGAGGAAGGAAAAATTTCAAAAATTGGGCCTGAAAATCCA
TATAATACTCCAGTATTTGCTATAAAGAAAAAGGACAGCACCAAATGGAGAAAATTGGTA
GATTTCAGAGAGCTCAATAAAAGAACTCAGGACTTTTGGGAAGTTCAATTAGGAATACCA
CATCCAGCAGGATTAAAAAGGAACAAATCAGTGACAGTACTAGATGTGGGAGATGCATAT
TTTTCAGTTCCTTTAGATGAAAGCTTTAGAAAATATACTGCATTCACCATACCTAGTAGA
AACAACGAGACACCAGGAATCAGATATCAGTACAATGTGCTACCACAGGGATGGAAAGGA
TCTCCGGCAATATTCCAGTGTAGCATGACAAAAATCTTAGAGCCCTTTAGAAAGAAAAAT
CCAGAAATGGTTATCTATCAATACATGGATGACTTATATGTAGGATCTGATTTAGAAATA
GGGCAACACAGAACAAAAATAGACGAGCTAAGAGCTCATCTGTTGAGCTGGGGATTTACT
ACACCAGACAAAAAGCATCAGAAGGAACCTCCATTTCTTTGGATGGGGTACGAACTCCAT
CCGGACAGATGGACAGTCCAGCCTATAGAACTGCCAGAAAAAGACAGCTGGACTGTCAAT
GATATACAGAAATTAGTGGGAAAACTAAATTGGGCAAGTCAAATTTATGCAGGGATTAAG
GTAAAGCAACTGTGTAAACTCCTTAGGGGAGCCAAAGCACTAACAGACATAGTACCACTG
ACTGAAGAAGCAGAGCTAGAACTGGCA--------------------------------

>101
-------------------GCCCCACCAGCAGAAGACTGGGGGATGGGAGGAGAGATAAC
CCC---------CKTACCAAAGAAGG---------------GGCAGAAAGACGAGGAAMM
------TCCTTCAATCTCCCTCARATCACTCTTTGGCAACGACCCCTTGTCACAATAAAA
ATAGGRGGACAGCTAAAAGAAGCTCTATTAGATACAGGAGCAGATGATACAGTATTAGAA
GATATAAATTTGCCAGGAAAATGGAAACCAAAAATGATAGGGGGAATTGGAGGTTTTATC
AAGGTAAGGCAATATGATCAGATACCTATAGAAATTTGTGGAAAAAAGGCTATAGGTACA
GTGTTAGTAGGACCTACACCTGTCAACATAATTGGACGAAATATGTTGACTCAGATTGGT
TGTACTYTAAATTTCCCAATTAGTCCTATTGACACTGTACCAGTAAAATTAAAGCCAGGA
ATGGATGGACCAAAGGTTAAACAGTGGCCATTGACAGAAGAAAAAATAAAAGCATTAACA
GAAATTTGTAAAGAGATGGAAGAGGAAGGAAAAATCTCAAAAATTGGGCCTGAAAATCCA
TATAATACTCCAGTATTTGCTATAAAGAAAAAGGAYAGCACCAAATGGAGGAAATTAGTA

GATTTCAGAGAGCTCAATAAAAGAACTCAGGACTTTTGGGAAGTTCAATTAGGAATACCG
CATCCAGCAGGATTAAAAAAGAAAAAATCAGTGACAGTACTGGATGTGGGAGATGCATAT
TTTTCAGTTCCTTTRGATGAAAGCTTTAGAAAGTATACTGCATTCACCATACCTAGTATA
AACAATGAGACACCAGGAATCAGATATCAGTACAATGTGCTACCACAGGGATGGAAAGGA
TCTCCGGCAATATTCCAGTGTAGTATGACAAAAATCYTAGAGCCCTTTAGAAGAAAAAAT
CCAGARATGGTTATCTATCAATACATGGATGACTTGTATGTAGGATCTGATTTAGAAATA
GGGCAGCACAGAATAAAAATAGAGGAGCTAAGAGCTCATCTATTGAGCTGGGGATTTACT
ACACCAGACAAAAAGCATCAGAAGGAACCTCCATTTCTTTGGATGGGATATGAACTCCAT
CCGGACAGATGGACAGTCCAGCCTATAGAACTGCCAGCAAAAGACAGCTGGACTGTCAAT
GATATACAGAAATTAGTAGGAAAACTAAATTGGGCAAGTCAAATTTATGCAGGGATCAAG
GTAAAGCAACTGTGTAGACTCCTCAGGGGAGCTAAAGCACTAACAGACATAGTACCACTG
ACTGAAGAAGCAGAG--

>102

--CAGGGAG--
-------GCCTTAACTTCCCTCARATCACTCTTTGGCAGCGACCCCTTGTCTCAATAAAA
GTAGGGGGACAGATAAAAGAGGCTCTYTTAGACACAGGAGCAGATGATACAGTATTAGAA
GAATTAAGTTTGCCAGGAAAATGGAAACCAAAAATGATAGGAGGAATTGGAGGTTTTATC
AAAGTAAGACAATATGAGCAAATACCTATAGAAATTTGTGGAAAAAAGGCTATAGGTACA
GTATTAGTGGGACCCACACCTGTCAACATAATTGGAAGAAAYCTGTTGACTCAGCTTGGA
TGTACATTAAATTTTCCAATCAGTCCCATTGAAACTGTACCAGTAAAATTAAAGCCAGGA
ATGGATGGCCCAAAGGTTAAACAATGGCCATTGTCAGAAGARAAAATAAAAGCATTAACA
GAAATTTGTAAYGAAATGGAGAAGGAAGGAAAAATTACAARAATTGGGCCTGAAAATCCA
TATAACACTCCAATATTTGCCATAAAAAAGAARGACAGTACTAAGTGGAGAAAATTAGTR
GATTTCAGGGAACTCAATAAAAGGACTCAAGATTTTTGGGAAGTTCAATTAGGAATACCA
CACCCAGCAGGGTTAAAAAAGAARAAATCAGTGACAGTACTGGACGTGGGGGATGCATAT
TTTTCAGTTCCYTTASATGAAGACTTCAGGAAATATACTGCATTCACCATACCTAGTATA
AACAATGAGACACCAGGGATTAGGTATCAATATAATGTGCTTCCACAGGGATGGAAAGGA
TCACCCGCAATATTCCAGCATAGCATGACAAAAATTCTAGARCCCTTTAGGGCACAAAAT
CCAGAAATAATCATCTATCAATATGTGGATGATTTGTTGGTAGGGTCTGATTTAGAAATA
GGGCAACATAGAGCAAAAATAGAGGAGTTAAGAGCACATCTGTTAAAGTGGGGATTTACC
ACACCAGACAAGAAACATCAGAAAGAACCTCCATTTCTTTGGATGGGGTATGAACTCCAT
CCTGACAARTGGACAGTACAGCCTATAAAGCTGCCAGAAAAGGATAGCTGGACTGTCAAT
GATATACAGAAGTTAGTGGGAAAATTAAACTGGGCAAGTCAGATTTACCCAGGAATTAAA
GTRARGCAACTTTGYAAACTCCTTAGGGGGACYAAAGCACTAACAGAYATAGTACCACTA
ACTGAAGAAGCAG--

>103

--GACAGGGAG--
-------GCCTTAACTTCCCTCAGATCACTCTTTGGCAACGACCCCTTGTCTCAATAAAA
GTAGGGGGACAGATAAAAGAGGCTCTYTTAGACACAGGAGCAGATGATACAGTATTAGAA
GAACTAAGTTTGCCAGGGAGATGGAAACCAAAAATGATAGGAGGAATTGGAGGTTTTATC

AAAGTAAGACAATATGAGCAAATACCTATAGAAATTTGTGGRAAAAAGGCTATAGGTACA
GTATTAGTGGGACCCACWCCTGTCAACATAATTGGAAGRAAYATGTTGACTCAGCTTGGA
TGTACATTAAATTTTCCMATYAGTCCCATTGAAACTGTACCAGTAAAATTAAAGCCAGGA
ATGGATGGCCCAARRGTTAAACAATGGCCATTRTCAGAAGAGAAAATAAAAGCATTAACA
GAAATTTGTAATGAAATGGAGAAGGAAGGAAAAATTACAAAAATTGGGCCTGAAAATCCA
TATAACACTCCAATATTTGCYATAAAAAAGAAGGACAGTACTAAGTGGAGAAAATTAGTA
GATTTYAGGGARCTCAATAAAAGGACTCAAGATTTTTGGGAARTTCAATTAGGAATACCA
CAYCCAGCAGGGTTAAAAAAGAAGAAATCAGTGACAGTACTGGAYGTGGGGGATGCATAT
TTTTCAGTTCCTTTATATGAAGACTTCAGGAAATATACTGCATTCACCATACCTAGTATA
AACAATRAGACACCAGGGATTAGGTATCAATATAATGTGCTTCCACAGGGATGGAAAGGA
TCACCCGCAATATTCCAGCATAGCATGACAARAATTTTAGAGCCCTTTAGRRCRCAAAAT
CCAGAAATAATCATTTATCAATATGTGGATGATTTGYATGTAGGRTCTGAYTTAGAAATR
GGGCAACATAGAGCAAAAATAGARGAGTTAAGAGCACATCTGTTAAAGTGGGGATTTACC
ACACCAGAYAAGAAACATCAGAAAGAACCYCCATTTCTTTGGATGGGGTATGAACTCCAT
CCTGACAAATGGACAGTACAGCCTATAMARCTGCCAGAAAARGATAGCTGGACTGTCAAT
GATATACAGAAGTTAGTGGGAAARTTAAACTGGGCAAGTCARATTTACCCAGGAATTAAR
GTAAGGCAACTTTGTAAACTCCTTAGGGGGACCAAAGCACTAACAGACATAGTACCACTA
ACTGAAGAAGCAGAGCT--

>104
---------------------CCACCAGCAGAGAGCTTCAGGTT---CGARGAGACAAC
YCC---------AGCTCCGAAGCAGG---------------GAYCGAAAGACAGGGAA--
-------GCTTTAACTTCCCTCAGATCACTCTTTGGCAACGACCCCTTGTCTCAATAAGA
GTAGGGGGCGAGACAAAAGAAGCTCTCTTAGACACAGGAGCAGATGATACAGTATTAGAA
GAAWTAAATTTACCAGGRAAATGGAARCCAAAAATGATAGGGGGAATTGGAGGTTTTATC
AARGTAAGACAATATGATCAAATACCTATAGAAATTTGTGGAAAAAGGGCAATAGGTACA
GTGTTAGTGGGACCCACACCTGTCAACATAATTGGAAGAAATATGTTGACTCAGCTTGGA
TGCACGCTAAATTTTCCAATCAGTCCCATTGAAACTGTACCAGTAAAATTAAAGCCAGGA
ATGGATGGCCCAAARGTTAAACAATGGCCATTRACAGAAGAGAAAATAAAAGCATTATTA
GAAATTTGTACAGARATGGAAAAGGAAGGAAAAATTTCAAAAATTGGGCCTGAAAATCCA
TAYAACACTCCAATATTTGCCATAAAGAAAAAAGATGGTACTAAATGGAGRAAATTAGTA
GATTTCAGRGAACTTAATAAAAGAACTCAAGACTTYTGGGAAGTTCAATTAGGAATACCA
CACCCAGSAGGATTAAAAAAGAACAAATCAGTAACAGTCCTGGATGTGGGTGATGCATAT
TTCTCAGTTCCTTTAGATAAAGAATTTAGGAAGTATACTGCATTTACCATACCTAGTATA
AACAATGAGACACCAGGGATTAGATATCAGTACAATGTGCTGCCACAGGGATGGAAAGGA
TCACCGGCAATATTYCAGAGTTGCATGACAAAAATCTTAGAGCCCTTTAGAATAAAAAAT
CCAGAATTAGTTATCTATCAATACATGGATGACCTGTATGTAGGATCTGATTTAGAAATA
GGGCAGCACAGAATAAAAATAGARGAGCTAAGAGCTCATCTATTGGACTGGGGATTTACT
ACACCAGATAAAAAGCATCAGAAGGAACCCCCATTCCTTTGGATGGGATATGAACTCCAT
CCTGACAAATGGACAGTCCAGCCTATAGAACTGCCAGAAAARGACAGCTGGACTGTCAAT
GATATACAGAAATTAGTGGGAAAACTGAATTGGGCAAGTCAAATTTATGCAGGRATTAAG
GTAAAGCAACTGTGTAAACTCCTCAGGGGAGCTAAAGCACTAACAGARATAGTACCACTG
ACTGAAGAAGCAGAG--

>107
----------------------CCACCAGCAGAAGACTGGGGGATGGAAGGAGAGATAAC
CTC---------CTTACCGAAGCAGG---------------AGCAGAAAGACAGGGAACA
ACA---TCCTTCAGTYTCCCTCAAATCACTCTTTGGCAACGACCCCTYGTCACAATAAAA
ATAGGAGGACAGCTAAGAGAAGCTCTATTAGATACAGGAGCAGATGATACAGTATTAGAA
GATATAAATTTACCAGGAAAATGGAAACCAAAAATGATAGGGGGAATTGGAGGTTTTATC
AAAGTMAGGCAATATGATCARATACCTATAGAAATTTGTGGRAAAAGRGCTATAGGTACA
GTGTTAGTAGGACCTACACCTGTCAACATAATTGGACGAAATATGTTGACTCAGCTTGGT
TGCACYTTAAATTTCCCAATTAGTCCTATTGACACTGTACCAGTGACATTAAAGCCAGGA
ATGGATGGACCAAAGGTTAAACAGTGGCCATTGACAGAAGAAAAAATAAAAGCATTAACA
GAAATTTGTAARGAGATGGAAGAGGAAGGAAAAATCTCAAAAATTGGGCCTGAAAATCCR
TATAATACTCCAGTATTTGCTATAAAGAAAAARGACAGCACCAAATGGAGAAAATTAGTA
GATTTCAGAGAGCTCAATAAAAGAACTCAGGAYTTTTGGGAAGTTCAATTAGGAATACCG
CATCCAGCAGGATTAAARAAGAAMAAATCAGTGACAGTACTAGATGTAGGRGATGCATAT
TTTTCAGTTCCTTTAGATGAAAGCTTTAGAAAGTACACTGCATTCACCATACCTAGTATA
AACAATGAGACACCAGGAGTCAGATATCAGTACAATGTGCTACCACAAGGATGGAAAGGA
TCTCCAGCAATATTCCAGTGTAGCATGACAAAAATCTTAGAGCCCTTTAGAAGAAAAAAT
CCAGAGATAATTATCTATCAATACATGGATGACTTGTATGTAGGATCTGATTTAGAAATA
GGCCAGCACAGAACAAAAATAGAGGAGCTAAGAGCTCATCTATTGAGCTGGGGATTTACT
ACACCAGACAAAAAGCATCAGAAGGAACCTCCATTTCTTTGGATGGGATATGAACTCCAT
CCGGACAAATGGACAGTCCAGCCTATAGAACTGCCAGAAARAGATAGCTGGACTGTCAAT
GATATACAAAAATTAGTGGGAAAATTAAATTGGGCAAGTCAAATTTATCCAGGGATTAGG
GTAAAGCAACTGTGYAAACTCCTCAGGGGARCCAAAGCACTAACAGATATAGTACCACT-

>109
---------------------CCCACCAGCAGAGAGCTTCAGGTTCGAGGAGACAACCCC
AGC------------TCCGAAGCAGG---------------AACCGAAGGACAGGGAA--
-------CCYTTAACTTCCCTCAGATCACTCTTTGGCAGCGACCCCTTGTCTCAATAAAA
GTAGGGGGYCAGATAAAAGARGCTCTCTTAGAYACAGGAGCAGATGATACAGTATTAGAA
GAARTAAMTTTGCCAGGAAAATGGAARCCAAAAATGATAGGAGGAATTGGAGGTTTTATC
AAAGTAAGACAATATGAGCAAATACYTATAGAAATTTGTGGAAAAAAGGCTATAGGTACA
GTATTAGTGGGACCCACACCTGTCAACATAATTGGAAGAAATATGTTGACCCAGCTTGGA
TGYACACTAAATTTTCCAATYAGTCCCATTGAAACTGTACCAGTAAAATTAAAGCCAGGA
ATGGATGGCCCAAAGGTTAAACAATGGCCATTGACAGAAGAGAAAATAAAAGCATTAACA
GCAATTTGTGAGGARATGGAGAAGGAAGGAAAAATTACAAAAATTGGGCCTGACAATCCA
TATAACACTCCAATATTTGCCATAAAAAAGAARGACAGTACTAAGTGGAGAAAATTAGTA
GATTTCAGGGAACTCAACAAAAGRACYCAAGATTTTTGGGAAGTTCAATTRGGAATACCA
CACCCAGCAGGGTTAAARAAGAAAAAATCAGTAACAGTCYTGGATGTGGGTGATGCATAT
TTCTCAGTTCCTTTAGATRAAGAYTTCAGGAAGTAYACTGCATTTACCATACCTAGTGTA
AACAATGAGACACCAGGRATTAGATATCAGTACAATGTGCTTCCACAGGGATGGAAAGGA
TCMCCAGCAATATTCCAATGTAGCATGACAAAAATCTTAGAGCCTTTTAGAAAACAYAAT
CCAGACATAGTTATCTATCAATACATGGATGACTTGTATGTRGGATCTGACTTAGAAATA
GGRCAGCATAGAACMAAAATAGAGGAACTGAGAGAACAYCTGTTAAAGTGGGGGTTTACC

ACACCAGACAAGAAACATCAGAAAGAACCYCCATTTCTTTGGATGGGGTATGAACTYCAT
CCTGACAAATGGACAGTACAGCCTATACARTTGCCAGAAAARGATAGCTGGACTGTCAAT
GATATACAGAARTTAGTGGGAAAATTAAACTGGGCAAGTCAGATTTACCCAGGAATTMAA
GTAARGCAACTTTGTAAACTCCTTAGGGGGRCCAAAGCACTAACAGACATARTACCACTR
ACTGAAGAAGCAGAG--

>110

CACCAGAGGAGAGCYTCAGATTTGGGGAA-----GAGATRACAACTCCATCTCAAAARCA
GGA---GCCAATAACTTCCCTCAAATCACTCTTTGGCAACGACCCCTTGTTACAATAAAG
ATAGGGGGGCAATTAAAGGAAGCTCTATTAGATACAGGAGCAGATGATACAGTATTAGAA
GACATGAATTTGCCAGGGAAATGGAAACCAAAAATGATAGGGGGAATTGGAGGTTTTATC
AAAGTAAGACAGTATGAACAGATACCCATAGAAATCTGTGGAMAKAAAGCTATAGGTACA
GTATTAGTAGGACCTACACCTGTCAACATAATTGGAAGAAATCTGTTAACTCAGCTTGGK
TGTACTTTAAACTTTCCAATCAGYCCTATTGAAACTGTACCAGTAAAACTAAAGCCAGGA
ATGGATGGCCCAAAGGTTAAACAATGGCCATTGACAAAAGARAAAATAGAAGCATTAACA
GCAATTTGTGATGAAATGGAAAAGGAAGGAAAAATTACAAAAATTGGGCCTGAAAATCCA
TACAACACTCCAATATTTGCCATAAAAAAGAAAGACAGTACTAAGTGGAGAAAACTAGTA
GATTTCAGGGAACTCAATAAAAGAACTCAAGATTTTTGGGAAGTTCAATTAGGAATACCA
CACCCAGCAGGATTAAAAAAGAAAAAATCAGTGACAGTGCTGGATGTGGGRGATGCATAT
TTTTCAGTTCCTTTATATGAAGACTTCAGGAAATATACTGCATTCACCATACCTAGTATA
AACAATGAAACGCCAGGGATTAGGTATCAGTACAATGTACTTCCACAGGGATGGAAAGGA
TCACCAGCAATATTTCAAAGTAGCATGACAAAAATTTTAGAGCCTTTTAGAAAACARWAT
CCAGATATAGWCATCTATCAATACATGGATGATTTGTATGTAGGATCTGACYTAGAGATA
GGGCAGCATAGAAKAAAAATAGAGGAACTGAGACAACATTTGTTGAGGTGGGGATTTACC
ACGCCAGACAAAAAACATCAGAAAGAACCTCCATTTCTTTGGATGGGGTATGAACTCCAT
CCTGACAAATGGACAGTACAGCCTATACAGTTGCCAGTACAAGATAGCTGGACTGTCAAT
GATATACAAAAGTTAGTGGGAAAGTTAAAYTGGGCAAGTCAGATTTATCCTGGAATTAAA
GTAAGGCAACTTTGTAAACTCCTTAGGGGGACCAAAGCACTAACAGACATAGTACCACTA
ACTGAAGAAGCAGAGCTAGAACTGGCAGA--------------------------------
------
>111
---------------------CCCACCAGCAGAGAGCTTCAGGTTCGAGGAGACAACCCC
AGC------------TCCGAAGCAGG---------------AACCGAAAGACAGGGAA--
-------GCCTTAACTTCCCTCAGATCACTCTTTGGCARCGACCCCTTGTCTCAATAAAA
RTAGGRGGTCAGATAAAAGAGGCTCTCTTAGACACAGGAGCAGATGATACAGTATTAGAA
GAAATAAATTTGCCAGGAAAATGGAAACCAAAAATGATAGGAGGRATTGGAGGTTTTATC
AAAGTAAGACAATATGAGCAAATACCTATAGAAATTTGTGGAAAAAAGGCTATAGGTACA
GTMTTRGTGGGACCYACACCTGTCAACATAATTGGAAGRAATATGTTGACCCAGCTTGGA
TGYACWCTAAATTTTCCAATYAGTCCCATTGAAACTGTACCAGTAAAATTAAAGCCAGGA
ATGGATGGCCCAAARGTTAAACAATGGCCATTGACAGAAGARAAAATAAAAGCATTAAGA
GAAATTTGTGATGAAATGGARAAGGAAGGAAAAATYACAAAAATTGGGCCTGACAATCCA
TATAACACTCCAATATTTGCCATAAAAAAGAAAGAYAGYACTAARTGGAGAAAATTAGTA
GATTTTAGGGARCTCAATAAAAGAACTCAAGATTTTTGGGAGGTTCAAYTAGGAATACCA

CACCCAGCAGGGTTAAAAAAGAAAAAATCAGTAACAGTCCTGGATGTGGGTGATGCATAT
TTCTCAGTTCCTTTAGATAARGATTTCAGAAAGTATACTGCATTTACCATACCTAGTATA
AACAATGAGACACCAGGRATTAGATATCAGTACAATGTGCTGCCACAAGGATGGAAAGGA
TCRCCAGCAATATTTCAATGTAGCATGACAAAAATCTTAGAACCTTTTAGAAARCAAAAT
CCAGACATRGATATCTATCAATACATGGATGACTTGTATGTAGGATCTGACTTAGAAATA
GGGCAGCAYAGAGCAAAAATAGAGGAACTGAGAGAACATCTGTTAAAATGGGGATTCACC
ACACCAGACAAGAAACATCAGAAAGAACCTCCATTTCTTTGGATGGGGTATGAACTCCAT
CCTGACAAATGGACAGTACAGCCTATACAGCTGCCAGAAAARGATAGCTGGACTGTCAAT
GATATACAGAAGTTAGTGGGAAAATTAAACTGGGCAAGTCAGATTTACCCAGGAATTAAA
GTAAGGCAACTTTGTAAACTCCTTAGGGGRGCCAAAGCACTAACAGAARTAGTASCACTA
ACTGAAGAAGCAGAG---

>112
--CC
CACCAGAGGAGAGTTTCAGGTTTGGGGAA-----GAGACAACAACTCCATCTCAGAAGCA
GGA---GCCCTTAACTTCCCTCAAATCACTCTTTGGCAGCGACCCCTTGTTRCAATAAAG
RTAGGGGGGCAATCAAAGGAAGCTCTATTAGAYACAGGAGCGGATGATACAGTRTTAGAA
GACATGAATTTGCCAGGRAAATRGAAACCAAAAATGATAGGGGGAATTGGAGGTTTTATC
AAAGTAAGACAGTATGAACAGATACCCATAGAAATCTGTGGRCACAAGGCTATAGGTACA
GTATTAGTAGGACCWACACCTGTCAACATAATTGGAAGAAATCTGTTGACTCAGCTTGGT
TGCACTTTAAATTTTCCAATCAGTCCCATTGAAACTGTACCAGTAAAATTAAAGCCAGGA
ATGGATGGCCCAAAGGTTAAACAATGGCCATTGACAGAAGARAAAATAAAAGCATTAACA
GCAATTTGTGATGAAATGGAAAAGGAAGRAAAAATTACAAAAATWGGGCCTGAAAACCCA
TATAACACTCCAATATTTGCTATAAAAAAGAAGGATAGTACTAARTGGAGAAAATTAGTA
GATTTCAGGGAGCTCAATAAAAGAACTCAAGATTTTTGGGAGGTTCAATTAGGAATACCA
CACCCAGCAGGGTTAAAAAAGAAAAAATCAGTGACAGTACTGGATGTGGGGGATGCATAT
TTTTCAGTTCCTTTACATGAAGATTTCAGGAAGTAYACTGCATTCACCATACCTAGTATA
AACAATGARACACCAGGGATTAGGTATCAGTACAATGTACTTCCACAGGGATGGAAAGGA
TCACCAGCAATATTCCAAAGTAGCATGACAAAAATCTTAGAGCCTTTCAGAAAACAAAAT
CCAGAYATAGTTATCTATCAATACATGGATGATTTGTATGTAGGATCTGACTTAGAGATA
GGRSAGCATAGAACAAAAATAGAGGAACTGAGACAACATTTGTTGAGATGGGGATTYACC
ACCCCAGACAAGAAACATCMGAAAGAACCTCCATTTCTKTGGATGGGGTATGAACTCCAT
CCTGACAAGTGGACAGTGCAGCCTATACAGCTGCCAGAAAAAGAAAGCTGGACTGTCAAT
GATATACAAAAGTTAGTGGGAAAATTAAACTGGGCAAGTCAGATTTATCCTGGAATCAAG
GTAAGGCAACTTTGTAAACTCCTTAGAGGGGCCAAAGCAYTAACAGACATAGTACCACTA
ACTGGAGAAGCAGAGCT--

>113
----------------------CACCAGCAGAGAGCTTCAGGTTCGAGGAGACAACCCC
AGC------------TCCGAAGCAGG---------------ARCTGAAAGACAGGGAA--
-------RCCTTAACTTCCCTCAGATCACTCTTTGGCARCGACCCCTYGTCTCAATAAAA
GTAGGGGGTCAGATAAAAGAGGCTCTCTTAGACACAGGAGCAGATGATACAGTATTAGAA
GAARTAAATTTRCCAGGAAAATGGAAACCAAAAATGATAGGAGGAATTGGAGGKTTTATC
AAAGTAAGACAATATGAGCAAATACCTATAGAAATTTGTGGRAAAAAGGCTATAGGTACA

GTMTTRGTRGGACCYACACCTGTCAACATAATTGGRAGRAATATGTTGACYCAGCTTGGA
TGYACAYTAAATTTTCCAATMAGTCCCATTGAAACTGTACCAGTAAAAYTRAARCCAGGA
ATGGATGGSCCAAAAGTTAAACAATGGCCATTGWCMGARGARAAAATAAARGCATTAAAA
GAAATTTGTGATGAAATGGAGAAGGAAGGAAARATTACAAAAATTGGGCCTGACAATCCA
TATAACACTCCAATATTTGCCATAAAAAAGAAAGATAGTACAAAGTGGAGAAAATTAGTA
GATTTTAGGGAACTCAATAAAAGAACTCAAGATTTTTGGGAGGTTCAACTAGGAATACCA
CACCCAGCAGGGTTAAAAAAGAAAAAATCAGTAACAGTCCTGGATGTGGGTGATGCATAT
TTCTCAGTTCCTYTAGATRAAGATTTCAGAAAGTATACTGCATTYACCATACCTAGTATG
AACAATGAGACACCAGGGATTAGATAYCAGTACAATGTGCTGCCACAAGGATGGAAAGGA
TCACCAGCAATATTTCAATGTAGCATGACAAAAATCTTAGAACCTTTTAGAAAACAAAAT
CCAGAYATAGATATCTATCAATACATGGATGACTTGTATGTAGGATCTGACTTAGAAATA
GGGCAGCATAGAACAAAAATAGAGGARCTGAGAGAACATCTGTTAAAATGGGGATTCACC
ACACCAGACAAGAAACATCARAAAGAACCTCCATTTCTTTGGATGGGGTATGAACTCCAT
CCTGACAAATGGACAGTACAGCCTATACAGCTGCCAGAAAAGGATAGCTGGACTGTCAAT
GATATACAGAAGTTAGTGGGAAAATTAAACTGGGCAAGTCAGATTTACCCAGGAATTAAA
GTAARGCAACTTTGTAAACTCCTTAGGGGGGCCAAAGCACTAACAGAAATAGTAGCACTA
ACTGAAGAAGCAGAGCT---

>114

----------------------CAGG---------------AGCAGAAAGA---GGAACA
CCCTCCTCCTTCAGTTTCCCTCAGATCACTCTTTGGCAACGACCCCTTGTCACAATAAAA
ATAGGGGGACAGCTRAAAGAGGCTCTATTAGATACAGGAGCAGATGATACAGTGTTAGAA
GATATAAATTTGCCAGGAAAATGGAAACCAAAAATGATAGGGGGAATTGGAGGTTTTATY
AAGGTAAGRCAATATGATGARATACCTATAGAAATTTGTGGAAAAAAGGTTATAGGTACA
GTATTAATAGGACCTACACCTGTCAACATAATTGGACGRAATATGTTGACTCAGCTTGGT
TGTACTTTAAATTTCCCAATTAGTCCTATTGAAACTGTACCAGTAACATTAAAGCCAGGA
ATGGATGGACCAAAGGTTAAACAGTGGCCATTGACAGAAGARAAAATAAAAGCATTAACA
GAAATTTGTAGAGAAATGGAAGAAGAAGGAAAAATCTCAAAAATTGGGCCTGAAAATCCA
TACAATACTCCAATATTTGCTATAAAGAAAAAGGAYGGTACCAAATGGAGRAAATTAGTA
GATTTCAGAGAGCTTAATAAAAGAACTCAGGATTTTTGGGAAGTYCAATTRGGAATACCR
CATCCAGCAGGTTTAAAAAAGAAAAAATCAGTAACAGTAYTRGATGTGGGAGATGCATAT
TTTTCAGTTCCTTTAGATAAAAAYTTCAGAAAGTATACTGCATTCACCATACCTAGTACA
AACAATGAGACACCAGGAATCAGATATCAGTACAATGTGCTGCCACAGGGATGGAAAGGA
TCACCAGCAATATTCCAGAGTAGCATGACAAAAATCTTAGAGCCCTTTAGAACAAAGAAT
CCAGARATAGWTATCTATCAATACATGGATGACTTGTATGTAGGATCTGATYTAGAAATA
GGGCAGCACAGAGCAAAAATAGAAGAGCTGAGAGCYCATCTATTGAGCTGGGGATTAACT
ACACCAGACAAAAAGCATCAAAAGGAACCTCCATTCCTTTGGATGGGATATGAACTCCAT
CCTGATAAATGGACAGTCCAGCCTATAGAACTGCCAGAAAAAGACAGCTGGACTGTCAAT
GATATACAGAAATTAGTGGGAAAATTAAATTGGGCAAGTCAAATTTATGCAGGYATTAAG
GTAAAGCAAYTGTGTARACTCCTCAGGGGAGCTAAAGCACTAACAGACATAGTACCACTG
ACTGAAGAAGCAGAGC--

>115

----------------CAGAAGCRGG---------------AGACGATAGACAAGGARCT
ATA---TCCTTTAGCCTCCCTCAAATCACTCTTTGGCAACGACCCCTCGTCCCAATAAGG
ATAGGGGGGCAATTAAARGAAGCTCTATTAGATACAGGAGCAGATGATACAGTATTAGAA
GAMATGAATTTGCCAGGAAGATGGAAACCAAAAATGATAGGGGGAATTGGAGGTTTTATC
AAAGTAAGACARTATGATCAGATACCYATAGAAATYTGYGGACACAAGRCTGWAGGTACA
GTRTTAATAGGACCTACACCTGTCAACATAATTGGAAGAAATCTGTTGACTCAGCTTGGT
TGCACTTTAAATTTTCCCATWAGTCCTATTGAAACTGTACCAGTAAAATTAAAGCCAGGR
ATGGATGGCCCAAAAGTTAAACAATGGCCATTGACAGAAGARAAAATAAAAGCATTAGTA
GAAATTTGTACAGAAATGGAAAARGAAGGRAAAATTTCAAAAATTGGGCCTGAAAATCCA
TAYAATACTCCAGTATTTGCCATAAAGAAAAARGACAGTACTAAATGGAGAAAATTAGTA
GATTTYAGRGAACTTAATAAAAGAACTCAAGACTTCTGGGAAGTTCAATTAGGAATACCA
CATCCWGCAGGGTTAAAAAAGAAAAAATCWGTAACAGTCCTGGATGTGGGTGATGCATAY
TTCTCAGTYCCTYTASATGAAGACTTCAGGAAGTATACTGCATTTACCATACCTAGTGTA
AACAATGAGACACCAGGGATCAGRTATCAGTAYAATGTGCTTCCACAGGGATGGAAAGGA
TCACCAGCAATATTYCAATGTAGCATGACAAAAATCTTAGAGCCTTTTAGAAARCAAAAT
CCAGACATAGTTATCTATCAATACATGGATGATTTGTATGTAGGMTCTGACTTAGAAATA
GGGCARCATAGAGCAAAAATAGARGAACTGAGASARCATYTGTTGAGSTGGGGATTYACM
ACACCAGACAAAAARCATCAGAAAGAACCYCCRTTCCTTTGGATGGGTTATGAACTCCAT
CCTGATAAATGGACAGTACAGCCTATAGTGCTRCCAGARAARGAMAGCTGGACTGTCAAT
GACATACAGAAGTTAGTGGGRAAGTTRAATTGGGCAAGTCARATTTATGCAGGGATTARR
GTAAGGGAATTATGTAAACTCCTTAGGGRAACYAAAGCACTAACAGAAGTAGTACCAYTA
ACAGAAGAAGCAGAGC--

>116

------------------AAAGCAGG---------------AGCAGAAGGACAAGGAACA
------TCCCTCAGTTTCCCTCAAATCACTCTTTGGCARCGACCCTTAGTCACAATAAAA
ATAGGAGGACAGCTAAGAGAAGCTCTATTAGATACAGGAGCAGATGATACAGTATTAGAA
GATATAAATTTGCCAGGAAAATGGAAGCCAAAAATGATAGGGGGAATTGGAGGTTTTATC
AAAGTAAGACAATATGATCAGATACTTATAGAAATTTGTGGAAAAAAGGCTATAGGTACA
GTGTTAGTAGGACCTACACCTRTCAACATAATTGGACGAAATATGTTGACTCAGCTTGGT
TGTACTTTAAATTTCCCAATTAGTCCTATTGACACTGTACCAGTAACATTAAARCCAGGA
ATGGATGGACCAAAGGTTAAACAGTGGCCTTTGACAGAAGAAAAAATAAAAGCATTAACA
GAAATTTGTAAAGARATGGAAGAGGAAGGAAAAATCTCAAAAATTGGGCCTGAGAATCCA
TATAATACTCCAGTATTTGCTATAAAGAAAAAGGACAGCACCAAATGGAGGAAATTAGTA
GATTTCAGAGAGCTCAATAAAAGAACTCAGGATTTTTGGGAAGTTCAATTAGGGATACCG
CATCCAGCAGGATTAAAAAAGAAAAAATCAATGACAGTACTAGATGTGGGAGATGCCTAT
TTTTCAGTTCCTTTAGATGAAAGCTTTAGAAAGTATACTGCATTCACCATACCTAGTATA
AACAATGAGACACCAGGAATTAGATATCAGTATAATGTACTACCACAAGGATGGAAAGGA
TCTCCGGCAATATTCCAGTGTAGCATGACAAAAATCTTAGAGCCCTTTAGAATAAAAAAT
CCAGAGATAGATATCTATCAATACATGGATGACTTGTATGTAGGATCTGATTTAGAAATA
GGGCAGCACAGAACAAAAATAGAGGARCTAAGAACTCATCTATTGAGCTGGGGATTTACT
ACACCAGACAAAAAACATCAGAAGGAACCTCCATTTCTTTGGATGGGATATGARCTCCAT

CCGGACAGATGGACAGTCCAGCCTATAGAACTGCCAGAAAARGACAGCTGGACTGTCAAT
GATATACAGAAATTAGTGGGAAAATTAAATTGGGCAAGTCAAATTTATGCAGGGATTAAG
GTAAAGCAACTGTGTAGACTCCTCAGGGGAGCTAAAGCATTAACAGAAATAGTACCACTR
ACTGAAGAAGCAGAG--

>117
------------CCCACCAGCYCCACCAGAGGAGAGCTTCAGGTTTGGGGAAGAGRCAGC
AGCAGCAGCCCCACCTCAGAAGCAGG---------------AGCCGAYAGACAGGGAGCT
ATA---CCCTTTAGCTTCCCTCAAATCACTCTTTGGCAACGACCCCTTGTCACAATAAAA
ATAGGGGGGCAATTAAAGGAAGCTCTATTAGATACAGGAGCAGATGATACAGTATTAGAA
GAAATGAATTTGCCAGGAAGATGGAAACCAAAAATGATAGGGGGAATTGGAGGYTTTATC
AAAGTAAGACARTATGATCAGRTATCCATAGAAATCTGTGGACACAAAGCTGTAGGTACA
GTATTAATAGGACCTACWCCWGTCAACATAATTGGGAGAAATYTGTTGACTCAGATTGGT
TGCACTTTAAATTTTCCYATTAGTCCTATTGAAACTGTACCAGTAAAATTAAARCCAGGA
ATGGATGGCCCAAAAGTTAAACARTGGCCATTGACAGAAGARAAAATAAAAGCATTAGTA
GAAATTTGTACAGAAATGGARAAGGAAGGRAAAATTTCAAAAATTGGRCCTGAAAATCCA
TAYAATACTCCAGTATTTGCCATAAAGAAAAAAGACAGTACTAAATGGAGAAAATTAGTA
GATTTYAGGGAACTTAATAAAAGAACTCARGAYTTCTGGGAAGTYCAAYTAGGAATACCA
CATCCCGCAGGGTTAAAAAAGAAAAAATCTGTAACAGTCCTGGATGTGGGTGATGCATAT
TTCTCAGTTCCTTTAGATRAAGAATTCAGGAAGTATACTGCATTTACCATACCTAGTGTA
AACAATGAGACACCAGGRATYAGATAYCAGTACAATGTGCTKCCACAGGGATGGAAAGGA
TCACCAGCAATATTCCARTGTAGCATGACAAAAATCTTAGAGCCTTTTAGAAAACAAAAT
CCAGACATAGTKATCTATCAATAYATGGATGATTTGTATGTAGGATCTGAYTTAGAAATA
GGRCAGCAYAGAGMAAAAATAGAGGAACTGAGACAACATCTGTTGRRGTGGGGRTTTACC
ACACCAGAYAAAAAACATCAGAAAGAACCYCCATTYCTTTGGATGGGTTATGAACTCCAT
CCTGAYAAATGGACAGTACAGCCTATAGTGCTGCCAGAAAAGGACAGCTGGACTGTCAAT
GACATACAGAARTTAGTGGGAAAGTTRAATTGGGCAAGYCARATTTATSCAGGGATTAAG
GTAAAGGAATTATGTAAACTCATTAGGGGAACCAARGCACTAACAGAAGTRGTACCACTA
ACAGAAGAAGCAGAGCT--

>118

---------------GCAGGAGCAGA---------------AAGAAAAGGACCATCACCC
TCACCCTCCCTCAGTTTCCCTCAAATCACTCTTTGGCAACGACCCTTAGTCACAATAAAA
ATAGAAGGACAGCTGAAAGAAGCTCTATTAGATACAGGAGCAGATGATACAGTATTAGAA
GATATAAATTTGCCAGGAAAATGGAAACCAAAAATGATAGGGGGAATTGGAGGTTTTATC
AAAGTAAGGCAATATGATCAAATACCTATAGAAATTTGTGGAAAAAAGGCTATAGGTACA
GTGTTAGTAGGACCTACACCTGTCAACATAATTGGACGAAATATGTTGACTCAGCTTGGT
TGTACTTTAAATTTTCCAATTAGTCCTATTGACACTGTACCAGTAACATTAAAACCAGGA
ATGGATGGACCAAAGGTTAAACAGTGGCCATTGACAGAAGAAAAAATAAAAGCATTAACA
GAAATTTGTAGGGAAATGGAAGAGGAAGGAAAAATCTCAAAAATTGGGCCTGAAAATCCA
TATAATACTCCAGTATTTGCTATAAAGAAAAAGGACAGCACCAAATGGAGGAAATTRGTA
GATTTCAGAGAGCTCAATAAAAGAACTCAAGACTTTTGGGAAGTTCAATTAGGAATACCG
CATCCAGCAGGATTAAAAAAGAAAAAATCAGTAACAGTACTAGATGTGGGAGATGCATAT

TTTTCAGTCCCTTTAGATGAAAGCTTTAGAAAATATACTGCATTCACCATACCTAGTATA
AACAATGAGACACCAGGAATCAGATATCAGTACAATGTGCTACCACAGGGATGGAAAGGA
TCTCCGGCAATATTCCAGTGTAGCATGACAAAAATCTTAGAGCCCTTTAGAAGCAAAAAT
CCAGARATAGTTATCTATCAATACATGGATGACTTRTATGTAGGATCTGATCTAGAAATA
GGGCAGCACAGAATAAAAGTAGATGAGCTAAGAGCTCATCTATTAAGCTGGGGATTTACT
ACACCAGACAAAAAGCATCAGAAGGAACCRCCATTTCTTTGGATGGGATATGAACTCCAT
CCGGACAGATGGACAGTCCAGCCTATAGAACTGCCAGAAAAAGACAGCTGGACTGTCAAT
GATATACAGAAATTAGTGGGAAAACTAAATTGGGCAAGTCAAATTTATCCAGGGATTAAG
GTAAAGCAACTRTGTAAACTCCTCAGGGGAGCTAARGCATTAACAGACGTAGTACCACTG
ACTGAAGAAGCAGAGCTAGAACTGGCA--------------------------------

>119
---------------------CCCACCAGCAGAGAACTGGGGGAGGGAGGAAGAGATAAC
ATC---------CTCACTGAAGCAGG---------------AACAGAAAGACAAGGAACA
CCCTCCTCCTTTAGTTTCCCTCAAATCACTCTTTGGCAACGACCCCTTGTCACAATAAAA
ATAGAAGGACAGCTAAGAGAAGCTCTATTAGATACAGGAGCAGATGATACAGTATTAGAA
GATATAAATTTGCCAGGAAAATGGAAACCAAAAATGATAGGGGGAATTGGAGGTTTTATC
AAGGTAAGACAATATGATGAGATACTTATAGAAATTTGTGGAAAAAAGGCTATAGGTACA
GTATTARTAGGACCTACACCCGTCAACATAATTGGACGAAATATGTTGACTCAGCTTGGT
TGTACTTTAAATTTCCCAATYAGTCCTATTGACACTGTACCAGTAAAATTAAAGCCAGGA
ATGGATGGACCAAAGGTTAAACAATGGCCATTGACAGAAGAAAAAATAAAAGCATTAACA
GAAATTTGTAAAGAAATGGAAGAGGAAGGRAAAATCTCAAAAATTGGGCCTGAAAATCCA
TATAATACTCCAGTATTTGCTATMAAGAAAAAGGACAGCACCAAATGGAGRAAATTAGTR
GATTTCAGAGAGCTCAATAAAAGAACTCAGGACTTTTGGGAAGTYCAATTAGGAATACCR
CATCCAGCAGGTTTAAAAAAGAAAAAATCAGTAACAGTACTRGATGTGGGAGATGCATAT
TTTTCAGTTCCTCTAGATRAAAGCTTTRGAAAGTATACTGCATTCACCATACCTAGTAYA
AACAATGAGACACCAGGAATCAGATATCAGTACAATGTACTGCCACAGGGATGGAAAGGA
TCACCAGCAATATTCCAGAGTAGCATGACAAAAATCTTAGAGCCCTTTAGAATAAARAAT
CCAGAAATGATTATCTATCAATACGTGGATGACTTGTATGTAGCATCTGATTTAGAAATA
GGGCAGCACAGAACAAAAATAGAGGAGCTRAGAGCTCATCTATTGAGCTGGGGATTTACT
ACACCAGACAAAAAGCATCAGAAGGAACCTCCATTCCTTTGGATGGGATATGAACTCCAT
CCTGAMARATGGACAGTCCAGCCTATAGAACTGCCAGAAAAAGACAGCTGGACTGTCAAT
GATATACAGAAATTAGTGGGAAAATTAAATTGGGCAAGTCAAATTTATCCAGGGATTAAG
GTAAAGCAACTGTGCAAACTCATCAGGGGAACTAAAGCACTAACAGATRTAGTACCACTR
ACTGAAGAAGCAGA--

>120

CTC---------CTTCCTGAA-CAGG---------------AGCAGAAAGACAAGGAACC
TCCTCCTCCTGTAACTTCCCTCAAATCACTCTTTGGCAACGACCCSTTGTCACAGTAAAA
ATAGSAGGACARCTGAGAGAAGCTCTATTAGATACAGGAGCAGATGATACAGTATTAGAA
GATATAAATTTGCCAGGAAAATGGAAACCAAAAATGATAGGGGGAATTGGAGGTTTTATC
AAAGTAAGGCAATATGATGAAATACCTATAGAAATTTGTGGAAAAAAGGCTATAGGTACA
GTGTTAGTAGGACCTACCCCTGTCAACATAATTGGACGAAATATGTTGACTCAGGTGGGT

TGTACTTTAAATTTCCCGATTAGTCCTATTGACACTGTACCAGTAAAATTAAAGCCAGGT
ATGGATGGACCAAAAGTCAAACAGTGGCCATTGACAGAAGAAAAAATAAAAGCATTAACA
GAAATTTGTAAAGAGATGGAAAAGGAAGGAAAGATTTCAAAAATTGGGCCTGAAAATCCA
TACAATACTCCAGTTTTTGCTATAAAGAAAAAGGACAGCACCAAATGGAGAAAATTAGTA
GATTTCAGAGAGCTCAATAAAAGAACTCAGGACTTTTGGGAAGTTCAATTAGGAATACCG
CATCCAGCAGGTTTAAAAAAGAACAAATCAGTAACAGTACTAGATGTGGGAGATGCATAT
TTTTCAGTTCCATTAGATAAAGACTTTAGAAAGTATACTGCATTCACCATACCTAGTACA
AACAATGAGACACCAGGAATCAGATATCAGTACAATGTGTTGCCACAGGGATGGAAGGGA
TCACCAGCAATATTCCAGAGTAGTATGACAAAAATCTTAGAGCCCTTTAGAATAAAAAAT
CCAGATGTAATTATCTATCAATACGTGGATGACTTATATGTAGGATCTGATTTAGAAATA
GGACAGCATAGAATAAAAATAGAGGAGCTAAGAGCTCATCTATTGAGCTGGGGATTTACT
ACACCAGACAAAAAGCATCAGAAGGAAYCTCCATTCCTTTGGYTGGGATATGAAMTCCAT
CCAGACAAATGGACAGTCCAGCCTATAGAACTGCCAGAAAAAGACAGCTGGACTGTCAAT
GATATACAGAAATTAGTGGGAAAACTAAATTGGGCAAGTCAGATTTATTCAGGGATTAGA
ATAAAACAACTGTGTAAACTCCTCAGGGGAGCTAAAGCACTAACAGACATAGTACCATTG
ACTGCAGAAGCAGAGCTAGAA---------------------------------------

>121
-----------------------CACCAGCRGAAAACTGGGAGATGAGGGAAGARACAAC
TYC---------CCTAATGAAGCAGG---------------AGCAGAAAGACAAGGAACA
KCCTCCTCCTTTAGTTTCCCTCAAATCACTCTTTGGCAACGACCCCTTGTCACAGTAAAA
ATAGGAGGACAGCTGAARGAAGCTCTATTAGATACAGGAGCAGATGATACAGTATTAGAA
GATATAAATTTGCCAGGAAAATGGAAACCAAAATTGATAGGGGGAATTGGAGGGTTTATC
AARGTAAGGCAATATGATCARATACTTATAGAAATTTGTGGAAAAAAGGCTATAGGTACA
GTRTTAGTAGGACCTACACCTGTCAACATAATTGGACGAAATATGTTGACTCAGATTGGT
TGTACTTTAAATTTCCCAATTAGTCCTATTGACACTGTACCAGTAACATTAAAGCCAGGA
ATGGATGGACCAAAGGTTAAACAGTGGCCATTGACWGAGGAAAAAMTAAAAGCATTAACA
GAAATTTGTAAAGAAATGGAAGAGGAAGGGAAAATMTCAAGAATTGGGCCTGAAAATCCA
TACAATACTCCAGTATTTGCTATAAAGAAAAAGGAYGGCACCAAATGGAGAAAATTAGTA
GATTTCAGAGAGCTCAATAAAAGAACTCAGGATTTTTGGGAAGTTCAATTAGGAATACCA
CATCCAGCAGGTTTAAAAAAGAATAAATCAATAACAGTRCTAGATGTGGGAGATGCATAT
TTTTCAGTYCCATTAGATAAAGAGTTTAGAAAGTATACTGCATTCACCATACCTAGTATA
AACAATGAGACACCGGGAATCAGATATCAGTACAATGTGCTRCCACAGGGGTGGAAAGGA
TCACCAGCAATATTCCAGTGTAGCATGACAAAAATYTTASARCCCTTTAGAGCAAAAAAT
CCAGARCTAATTATTTATCAATACRTGGATGAYTTGTATGTAGGATCTGATTTAGAAATA
GGACAGCATAGAGCAAAAATAGAGGAGCTMAGAGCTCATCTATTGAGCTGGGGATTTACT
ACACCAGACAAAAARYACCAGAAGGAACCTCCATTCCTTTGGATGGGATATGAACTCCAT
CCTGACAGGTGGACAGTCCAGCCTATAGAACTGCCAGAAAAAGAAAGCTGGACYGTCAAT
GATATACAGAAATTAGTAGGAAAATTAAATTGGGCAAGTCAGATTTATGCAGGAATTAAA
ATAAAGCAAYTGTGTAAACTCCTCAGGGGAGCTAAAGCACTAACAGAAATAGTRCCACTG
ACTRCAGAAGCAGAGCTAGAACTGGCAGA-------------------------------

>122
----------------------CCACCAGAGGAGAGCTTCAGGTTTSGGGAGGAGACAAC

AAC------TCCCCCYCAGAAGCAGG---------------AGCCGATAGACAAGGAACT
ATA---TCCTTTAGCTTCCCTCAAATCACTCTTTGGCAACGACCCCTYGTCACAATAAAG
ATAGGGGGGCAATTAAAGGAAGCTCTATTAGATACAGGAGCAGATGATACAGTATTAGAA
GACATGAATTTGCCAGGRAGATGGAAACCAAAAATGATAGGGGGAATTGGAGGYTTTATC
AAAGTAAGACAGTATGATCAGRTACCCATAGAAATTTGTGGACACAAAGCTATAGGTACA
GTGTTAATAGGACCTACACCTGTCAACATAATTGGAAGAAATYTGTTRACTCAGCTTGGK
TGCACTTTAAATTTTCCCATTAGTCCTATTGAAACTGTACCAGTAAAATTAAAGCCAGGA
ATGGATGGCCCAAAAGTTAAACAATGGCCATTGACAGAAGAAAAAATAAAAGCATTAGTA
GAAATTTGTACAGAAATGGAAAAGGAAGGRAAAATTTCAAAAATTGGGCCTGAAAATCCA
TACAATACTCCAGTATTTGCCATAAAGAAAAAAGACAGTACTAARTGGAGAAAATTAGTA
GATTTCAGGGARCTTAATAAAAGAACTCAAGACTTCTGGGAAGTTCAATTAGGAATACCA
CATCCYGCAGGGTTAAAAAAGAAAAAATCAGTWACAGTCCTGGATGTGGGTGATGCATAT
TTCTCAGTTCCRTTAGATGAAGACTTCAGGAAGTATRCTGCATTTACCATACCTAGTGTA
AACAATGAGACACCAGGGATTAGATATCAGTACAATGTGCTCCCACAGGGATGGAAAGGA
TCACCAGCAATATTCCAATKTAGCATGACAAAAATCTTAGAGCCTTTTAGAAAACAAAAT
CCAGACATAGTWATCTATCAATACATGGATGAYTTGTATGTAGGATCTGACTTAGAAATA
GGGCAGCATAGAACAAAAATAGAGGAACTGAGAGAACATCTGTTGAGGTGGGGATTTACC
ACMCCAGACAAAAAACATCAGAAAGAACCTCCATTCCTTTGGATGGGTTATGAACTCCAT
CCTGATAAATGGRCAGTGCARCCTATARTACTGCCAGAAAARGACAGCTGGACTGTCAAT
GACATACAGAAGTTAGTGGGAAARTTGAATTGGGCAAGTCAGATTTATGCAGGRATYAAR
GTAMRGGAATTATGTAAACTCMTTAGGGGAACCAAAGCACTAACAGAAGTAGTACCACTA
ACAGAAGAAGCAGAGCTAGAA---------------------------------------

>123
--CC
CACCAATGGAGAGCTTCAGGTTTGGGGAR-----GAGACAACAACTCCAGCTCAGAAGCA
GGA---TCCTGTAACTTCCCTCAAATCACTCTTTGGCAACGACCCCTCGTCACAATAAAG
ATAGGGGGGCAATTAAAGGAAGCTCTATTAGATACAGGAGCAGATGATACAGTATTAGAA
GAAATGAATTTGCCAGGGAAATGGAAACCAAAAATGATAGGGGGAATTGGAGGTTTTATY
AAAGTAAAACAGTATGAAGARATACCCATAGAGATCAGTGGGCACAAGGCTATAGGTACA
GTATTAGTGGGACCYACACCTGTCAACATAATTGGAAGAAATYTGTTGACTCARATTGGT
TGCACTTTAAATTTTCCRATCAGTCCCATTGAAACTGTACCAGTAAAATTAAAGCCAGGA
ATGGATGGCCCAAARGTTAAACAATGGCCATTGACAGAAGAGAAAATAAAAGCATTAACA
GAAATTTGTGATGAAATGGAAAAGGAAGGAAAAATTTCAAAAATTGGGCCTGAAAATCCA
TATAACACTCCAATATTTGCCATAAAAAAGAAGRACAGTACTAGGTGGAGGAAATTAGTA
GATTTCAGGGAACTCAATAAAAGAACTCAAGATTTTTGGGAAGTTCAATTAGGAATACCA
CACCCAGCAGGGTTAAAAARGAGAAAATCAGTGACAGTACTGGATGTGGGGGATGCATAT
TTTTCAGTTCCTTTATATGAAGACTTCAGGAAGTATACTGCATTCACCATACCTAGTACA
AACAATGAAACACCAGGRATTAGGTATCAGTACAATGTACTTCCACAGGGATGGAAAGGG
TCACCAGCAATATTCCAAAGTAGCATGACAAAAATCTTAGAGCCRTTTAGAAAGCAAAAT
CCAGACATAGAKATCTRTCAATACRTGGATGATTTGTATGTAGGATCTGACTTAGAGATA
GGGCARCATAGAACAAAARTAGAGGAACTRAGACRACATTTGTTGAGGTGGGGATTTDYC
ACACCAGACRAGAAACATCAGAAAGAACCTCCATTTCTTTGGATGGGGTATGAACTCCAY
CCTGACAAATGGACAGTACAGCCTATACAGCTGCCAGAAAAAGATAGCTGGACTGTCAAT

GATATACAAAAGTTAGTGGGRAAATTAAAYTGGGCAAGTCAGATTTATCCTGGAATTAAA
GTAAGGCAACTTTGTAAACTCCTTAGGGGGGCCAAAGCACTAACAGACATAGTACCACTA
ACTGAAGAAGCAGAGCTAGAACTGGCAGA------------------------------

>124
----------------------CACCAGCAGAGAACTGGRGGAGGGARGARGAGATMAC
ATC---------CTCATTGAAACAGG---------------AGCAGAAAGACAAGGAACA
YCCTCCTCCTTTAGTTTCCCTCAAATCACTCTTTGGCAACGACCMGTTGTCACAGTAAGA
ATAGARGGACAGCTGAAGGAAGCTCTATTAGATACAGGGGCAGATGATACAGTATTAGAA
GATATAAATTTGCCAGGAAAATGGAAACCAAAAATGATAGGGGGAATTGGAGGTTTTATC
AAAGTAAGACAATATGATCAGATACTTATAGAAATTTGTGGRAAAAAGGCTATAGGTACA
GTATTAGTAGGACCTACACCTGTCAACATAATTGGGCGAAATATGTTGACTCAGATTGGT
TGTACTTTAAATTTCCCAATAAGTCCTATTGATACTGTACCAGTAAAATTAAAGCCAGGA
ATGGATGGACCAAAGGTTAAACAATGGCCATTGACAGAAGAAAAAATAAAAGCATTRACA
GAAATTTGTAAAGAAYTGGAAGAGGAMGGAAAAATCTCAAGAATTGGGCCTGAGAATCCA
TATAATACTCCAATATTTGCTATAAAGAAAAAARACAGCACCAAATGGAGGAAATTAGTA
GATTTCAGAGAGCTCAATAAAAGAACACAAGACTTTTGGGAAGTTCAATTAGGAATACCA
CATCCAGSAGGATTAAAAARGAAAAAATCAGTAACAGTACTAGATGTGGGAGATGCATAT
TTTTCAGTTCCTTTAGATGAAAGCTTTAGAAAGTATACTGCATTCACCATACCTAGTACA
AACAATGAGACACCAGGAATCAGATATCAGTACAATGTGCTACCACAGGGATGGAAAGGA
TCACCAGCAATATTCCAGARTAGCATGACAAAAATCTTAGAGCCCTTTAGACTAAAAAAT
CCAGAAATGATTATCTGTCAATAYGTGGATGATTTGTATGTAGGATCTGATTTAGAAATA
GGGCAACACAGAGCRAAAATAGAGGAGCTGAGAGCTCATCTATGGAGCTGGGGATTTTAT
ACACCAGAMAAAAAGCATCAGAAGGAACCTCCATTCCTTTGGATGGGATATGARCTCCAT
CCTGACAAATGGACAGTCCAGCCTATAGAACTGCCAGAAAAGGACAGCTGGACTGTCAAT
GATATACAGAAATTAGTGGGAAAACTAAATTGGGCAAGTCAAATTTATCCAGGGATTAAG
GTAAAACAACTATGTAARCTTATCAGGGGAACTAAAGCACTAACGGAGGTAGTACCACTG
ACTGAAGAAGCA--

>125
--------------------CCCACCAGCAGAGAGCTTCAGGTTCGAGGAGACAACCCC
AGC------------TCCAAGGCAGG---------------AGTCAAAGGACAGGGAA--
-------CCCTTAACTTCCCTCAAATCACTCTTTGGCAACGACCCCTTGTCTCAATAAAA
GTAGGGGGTCAGATAAAAGAGGCTCTCTTAGACACAGGAGCAGATGATACAGTATTAGAA
GAAATAAGTTTACCAGGAAAATGGAAACCAAAAATGATAGGAGGAATTGGAGGTTTTATC
AAAGTAAGACAATATGATCAAATACCYATAGAAATTTATGGAAAGAAGGCTATAGGTACA
GTATTAGTAGGACCTACACCTGTCAACATAATTGGAAGGAATCTGTTGACTCAGCTTGGT
TGCACTTTAAATTTTCCCATTAGTCCTATTGAAACTGTACCAGTAAAATTAAAGCCAGGA
ATGGATGGCCCAAAGGTCAAACAATGGCCATTGACAGAAGAGAAAATAAAAGCATTAGTA
GAAATTTGTACAGAAATGGAAVAGGAAGGGAAAMTTTCAAAAATTGGGCCTGAAAATCCA
TACAATACTCCAGTATTTGCCATAAAGAAAAAAGACAGTACTAAATGGAGAAAATTAGTA
GATTTCAGGGAACTTAATAAAAGGACTCAAGACTTCTGGGAAGTTCAATTAGGAATACCA
CAYCCGGCAGGATTAAAAAAGAAAAAATCAGTAACAGTACTGGATGTGGGGGATGCATAT
TTCTCAGTCCCTTTATATGAGGACTTCAGGAAGTACACTGCATTTACCATACCTAGTATA

AACAATGAGACACCAGGGATTAGGTATCAGTACAATGTACTTCCACAAGGATGGAAAGGA
TCACCAGCAATATTCCAATGTAGCATGACAAAAATCTTAGAGCCTTTTAGAAAGCAAAAT
CCAGACATAGTCATCTATCAGTATATGGATGACTTGTATGTAGGATCTGACYTAGAAATA
GGGCAACATAGAGCAAAAATAGAAGAGTTAAGAGAACACCTGTTAAAGTGGGGAYTTACC
ACACCAGACAAGAAACATCAGAAAGAACCTCCATTTCTTTGGATGGGGTATGAACTCCAT
CCTGACAAATGGACAGTACAGCCTATACAGCTGCCAGAAAAGGATAGCTGGACTGTCAAT
GATATACARAAGTTAGTGGGAAAATTAAACTGGGCAAGTCAGATTTACCCAGGAATTAAA
GTAAGGCAACTKTGTAAACTCCTTAGGGGGACTAAAGCACTAACAGAAATAGTACCACTA
ACT---

>126
---AAC
CTC---------CTTACCGAAACAGG---------------AGCAGAAAAACAAGGAACA
------TCYCTCAGTCTCCCTCAAATCACTCTTTGGCARCGACCCCTYGTCACAATAAAA
GTAGGAGGACAGCTRAGAGARGCTCTATTAGATACAGGAGCAGATGATACAGTATTAGAA
GATATAAATTTGCCAGGAAAATGGAAACCAAAAATGATAGGGGGAATTGGAGGTTTTATC
AAAGTAAGGCAATATGATCAGATAACTATAGAAATTTGTGGAAAAAAGGCTATAGGTACA
GTGTTAGTAGGACCTACACCTGTCAACATAATTGGACGRAATATGTTGACTCAGATTGGY
TGTACTTTAAATTTCCCAATTAGTCCTATTGAHACTGTACCAGTAACATTAAAGCCAGGA
ATGGATGGACCAAAGGTTAAACAGTGGCCATTRACAGAAGAAAAAATAAAAGCATTAACA
GAAATTTGTGAAGAGATGGAAAAGGAAGGAAAAATCTCAAAAATTGGGCCTGAAAATCCA
TATAATACTCCAGTATTTGCCATAAAGAAAAAGGACAGCACCAAATGGAGGAAACTAGTA
GATTTCAGAGAGCTCAATAAAAGAACTCAGGAYTTTTGGGAAGTTCAATTAGGAATACCR
CATCCWGCAGGATTAAAAAAGAAAAAATCAGTGACAGTACTAGATGTAGGGGATGCATAT
TTTTCAGTTCCTTTAGATGAAAGCTTTAGAAAGTATACTGCATTCACCATACCTAGTAGA
AACAATGAGACMCCAGGAATCAGATATCAGTACAATGTGCTACCACAGGGATGGAAAGGA
TCTCCKGCAATATTCCAGTGTAGCATGACAAAAATCTTAGAACCYTTTAGAAGYAAAAAT
CCAGAGATGGTTATCTATCAATACATGGATGACTTRTATGTAGGATCTGATTTAGAAATA
GGGCAGCATAGAACAAAAATAGAGGAGCTRAGAGCTCATCTATTGAGCTGGGGATTTACT
ACACCAGACAAAAAGCATCARAAGGAACCTCCATTTCTTTGGATGGGATATGAACTCCAT
CCRGACAGATGGACAGTCCAGCCTATAGAACTGCCAGAAAARGATAGCTGGACTGTCAAT
GATATACAGAARTTAGTGGGAAAATTAAATTGGGCAAGTCAAATTTATCCAGGGATTAGG
GTAAAGCAACTGTGTAAACTCCTCAGGGGAGCTAAAGCACTAACAGACATAGTACCACTG
ACTGWAGAAGCARAG---

>127
---------------------CCCACCAGCAGAGAACTGGGGGATGGGGGAAGA------
------------CTTACTGAAGCAGG---------------ARCAGAAAGACARGGAACA
GCCYCCACCYTCAATCTCCCTCAAATCACTCTTTGGCAACGACCCCTTGTCACAGTAAAA
ATAGGAGGACAGATAAAAGAAGCTCTATTAGATACAGGAGCAGATGATACAGTATTAGAA
GATATAAATTTGCCAGGAAAATGGAAACCAAAAATGATAGGGGGAATTGGAGGTTTTATT
AAGGTAAGGCAATATGATCAGATACTTATAGAAATTTGTGGAAAAARGGCTATAGGTACA
GTATTAGTAGGACCCACGCCTGTCAACATAATTGGACGAAATATGTTGACTCAGATTGGT
TGTACTTTAAATTTCCCAATTAGTCCTATTGACACTGTACCAGTAACATTAAAGCCAGGA

ATGGATGGACCAAAAGTTAAACARTGGCCATTGACAGAAGAAAAAATAAAAGCATTAACA
GAAATTTGTAAGGARATGGAAGARGAAGGAAAAATCTCAAAAATCGGGCCTGAAAATCCA
TACAATACTCCAGTATTTGCTATAAAGAAAAAGGACAGCACCAAATGGAGRAAATTAGTA
GATTTCAGAGAGCTTAATAAAAGAACTCAGGATTTTTSGGAARTTCAATTAGGAATACCR
CAYCCAGCRGGYTTAAAAAAGAAYAAATCAGTAACAGTACTAGATGTGGGAGATGCATAT
TTTTCAATTCCTTTAGATGAAAATTTTAGAAAGTATACTGCATTCACCATACCTAGTATA
AATAATGAGACACCAGGAATCAGATATCAGTACAAYGTGCTGCCACAGGGATGGAARGGA
TCACCAGCAATATTCCAGAGTAGCATGATAAAAATCTTAGAGCCCTTTAGAAAAAGAAAT
CCAGAAATGGTTATTTATCAATACATGGATGACYTGTATGTAGGATCTGATTTAGAAATA
GGGCAGCACAGAACAAAAATAGAGGAGCTRAGAGCTCATCTATTGAGCTGGGGAYTTACT
ACACCAGATAARAAGCATCAGAAGGAACCTCCATTCCTTTGGATGGGRTATGAACTCCAT
CCTGACACATGGACAGTCCAGCCTATAGAACTGCCAGAAAAAGACAGCTGGACTGTCAAT
GATATACAGAAATTAGTGGGAAAACTAAATTGGGCAAGTCAAATATATGCAGGAATTAGG
GTAAGRCAACTGTGTAAACTCCTCAGGGGAGCTAAAGCACTAACAGACATAGTACCATTG
ACTGAAGAAGCAGAGCTAGAA---------------------------------------

>128
---------GAGCCAAACAGCCCCACCAGAGGAGAGTCTCAGGTTTGGGGAAGAGACAAC
AAC------TCCACCTCAGAAGCAGG---------------AGCCGATAGACAAGGAACT
ATA---TCCYTTAACTTCCCTCAAATCACTCTTTGGCAGCGACCCCTTGTCACAGTAAAA
ATAGGAGGACAGCTGAAAGAAGCTCTATTRGATACAGGAGCMGATGAYACAGTATTAGAA
GACATGAGCTTGCCAGGAAAATGGAAACCAAAAATGATAGGGGGAATTGGAGGTTTTATC
AAAGTAAGACAGTATGATCARATAYCTRTAGAAATCTGYGGACACAAAGTTATAGGTACA
GTGTTAATAGGACCTACACCTGTCAACATAATTGGAAGAAATCTGTTAACTCAGCTTGGK
TGCACTTTAAATTTTCCYATTAGTCCTATTGAAACTGTACCAGTAAAATTAAAGCCAGGA
ATGGATGGCCCAAAAGTTAAACAATGGCCATTAACAGAAGAAAAAATAAAAGCATTAGTA
GAAATTTGTACAGAAATGGAAAAGGAAGGGAAAATTTCAAAAATTGGGCCTGAAAATCCT
TACAATACTCCAGTATTTGCCATAAAGAAAAAAGACAGTACTAAATGGAGAAAATTAGTA
GATTTCAGAGAACTTAATAARAGAACTCAGGACTTTTGGGAAGTCCAATTAGGAATACCA
CATCCCGCAGGGTTAAAAAAGAGGAAATCAGTGACAGTACTAGATGTAGGAGATGCATAT
TTTTCAGTTCCTCTAGATAAAAGCTTTAGAAARTATACTGCATTTACCATACCTAGTATA
AATAATGAAACACCAGGGATCAGATAYCAGTACAATGTRCTTCCACAGGGATGGAAAGGA
TCACCAGCAATATTTCAATGTAGCATGACAAAAATCTTAGAGCCTTTTAGAAAACAAAAT
CCAGAAATARTTATCTATCAATACATGGATGATTTGTATGTAGGATCTGACTTAGAAATA
GGGCAGCATAGAACAAAAATAGAGGAACTGAGAAAACAYCTGTTAAGGTGGGGAYTTACC
ACACCAGACAAAAAACATCAGAAAGAACCTCCATTYCTTTGGATGGGTTATGAACTCCAT
CCTGATAAATGGACAGTRCAGCCTATAATGCTGCCAGAAAAGGATAGCTGGACTGTCAAT
GACATACAGAAGTTAGTGGGAAAGCTAAATTGGGCAAGTCAAATTTATGGAGGAATCAAG
GTAAAGCAACTATGTAAACTCCTCAGGGGAACTAAAGCACTAACAGACATAGTACCACTG
ACTGAAGAAGCASA--

>129
---------------------CCCACCAGCAGAGAGCTTCAGGTTCGAGGAGACAACCCC
GRG------------YCCGAAGCAGG---------------AACCGAAAGACAGGGAA--

-------CCYTTAACTTCCCTCAGATCACTCTTTGGCAGCGACCCCTTGTCTCAATAAAA
GTAGRGGGTCAAATAAAAGARGCTCTTTTAGACACAGGAGCAGATGATACAGTATTAGAA
GAADTAAATTTGCCAGGAAAATGGAAACCAAAAATGATAGGAGGAATTGGMGGTTTTATC
AAAGTAAGACAATATGAGCAAATAACTATAGAAATTTGTGGAAAAAAGGCTATAGGTACA
GTATTAGTGGGACCCACACCTGTTAACATAATTGGAAGRAAYATGTTAACCCAGCTTGGA
TGCACACTAAATTTTCCAATCAGTCCCATTGAAACTGTACCAGTAAAATTAAAGCCAGGA
ATGGATGGRCCAARGGTTAAACAATGGCCATTGACAGAAGARAAAATAAAAGCATTAACA
GCAATTTGTGATGAAATGGARAAGGAAGGAAAAATTTCAAGAATTGGGCCTGACAATCCA
TATAACACTCCAATATTTGCCATAAAAAAGAAGGAYAGTACTAAGTGGAGRAAATTAGTG
GATTTCAGGGAACTCAATAAAAGAACTCAAGATTTTTGGGAAGTTCAATTAGGAATACCA
CACCCAGCAGGGTTAAAAAAGAARAAATCAGTAACAGTCCTAGATGTGGGTGATGCATAT
TTCTCAGTTCCTTTAGATAAAGACTTCAGGAAGTATACTGCATTTACCATACCTAGTGTA
AACAATGAGACACCAGGGATTAGATATCAGTACAATGTGCTCCCACAGGGATGGAAAGGA
TCACCAGCRATATTYCAATGTAGCATGACAAAAATCTTGGAGCCTTTTAGAAAACAAAAT
CCAGACATAGTTATTTATCAATACATGGATGACTTGTATGTGGGATCTGACTTAGAAATA
GGGCAGCATAGAACAAAAATAGAGGAACTGAGAGAACATCTGTTAAAGTGGGGRTTTACY
ACACCAGACAAGAAACATCAGAAAGARCCTCCATTTCTYTGGATGGGATATGAACTCCAT
CCTGACAAATGGACAGTACAGCCTATACAGCTGCCAGAAAAGGATAGCTGGACTGTCAAT
GATATACAGAAGTTAGTGGGAAAATTAAACTGGGCRAGTCAGATTTACCCAGGAATTAAA
GTAAGGCAACTGTGTAAACTCCTTAGGGGGGCCAAAGCACTAACAGAAATAGTACCACTA
ACTGAAGAAGCAGAGCTAGAA--------------------------------------
